# A Unified Platform for Direct Alkynylation from Alcohols via Deoxygenative Metallaphotoredox Catalysis

**DOI:** 10.1021/jacs.6c10795

**Published:** 2026-07-10

**Authors:** Yi-Hao Li, David W. C. MacMillan

**Affiliations:** 6740 Merck Center for Catalysis at Princeton University, Princeton, New Jersey 08544, United States

## Abstract

Alkyne motifs are privileged functional groups in medicinal chemistry and serve as key handles in bioorthogonal click chemistry. Herein, we report a general metallaphotoredox deoxygenative C­(*sp* ^*3*^)–C­(*sp*) coupling strategy that enables the direct conversion of alcohols into alkynes using alkynyl bromides as coupling partners. This protocol operates under redox-neutral, mild conditions via NHC-mediated alcohol activation and nickel catalysis, enabling the generation of alkyl radicals from alcohols and their subsequent cross-coupling with alkynyl electrophiles. The method exhibits broad substrate scope across structurally complex alcohols, including pharmaceuticals, peptides, nucleosides, and natural products, providing a practical platform for the synthesis of click-ready alkynes from readily available alcohol feedstocks. In addition, the alkynyl bromide manifold enables modular diversification and late-stage functionalization of amino acid side chains and other bioactive molecules, significantly expanding the accessible chemical space for alkyne-based medicinal and bioconjugation chemistry.

## Graphical abstract

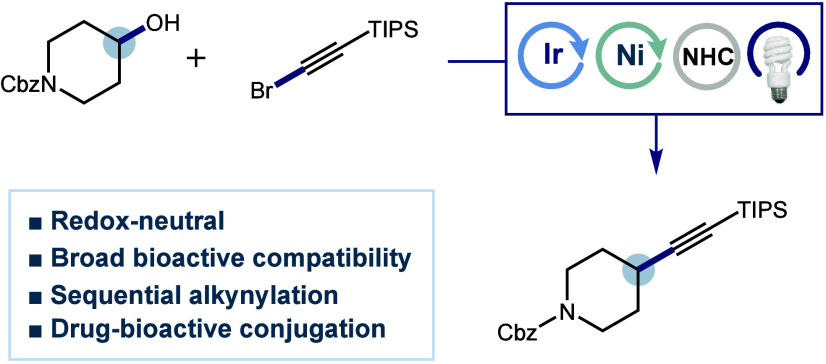

Alkynes have emerged as indispensable functional groups in modern medicinal chemistry owing to their distinctive hydrophobicity, structural rigidity, and linear geometry, which enable unique interactions with biological targets.^1–3^ Since their initial incorporation into pharmaceuticals in 1959, acetylene motifs have been widely exploited in drug discovery and development.^4–6^ Moreover, terminal alkynes are frequently introduced into bioactive molecules as versatile chemical biology handles, enabling target identification and assessment of target engagement through Cu­(I)-catalyzed azide–alkyne cycloaddition (CuAAC) as click reactions.^7–9^ One powerful strategy for accessing alkynes involves direct conversion of common functional groups into alkyne-containing building blocks.^10^ Expanding beyond traditional Sonogashira coupling of terminal alkynes with aryl or vinyl halides,^11–13^ recent advances employ *sp* ^*3*^-hybridized, bench-stable coupling partners, including redox-active esters (RAEs),^14^ sulfonyl hydrazides,^15^ xanthate esters,^16^ and others.^17,18^ While effective, these methods often require an additional isolation step for functional group activation and, in some cases, employ stoichiometric oxidants or reductants, which can limit their application to highly complex, bioactive molecules. Direct, in situ activation methods that bypass prefunctionalization and tolerate complex, bioactive substrates therefore remain an important unmet need.

Metallaphotoredox catalysis has emerged as a powerful and general platform for constructing a wide range of previously inaccessible molecular architectures under mild and efficient conditions.^19–23^ Recently, our group developed *N*-heterocyclic carbene (NHC) precursors as mild, robust activators of alcohols that enable the *in situ* generation of alkyl radicals from hydroxyl groups via metallaphotoredox catalysis.^24^ This strategy enables a broad range of deoxygenative cross-couplings,^25–31^ forming C­(*sp* ^*3*^)–C­(*sp* ^*2*^) and C­(*sp* ^*3*^)–C­(*sp* ^*3*^) bonds. However, a general platform for C­(*sp* ^*3*^)–C­(*sp*) bond formation via deoxygenative coupling of alcohols with alkynes remains underdeveloped.^32–34^ Although deoxygenative alkynylation via alkynyl sulfone addition has been reported, its distinct radical addition mechanism, limited access to dialkyl-substituted products, and requirement for excess alcohol substrate restrict its broader applicability.^35,36^ More importantly, late-stage alkynylation of complex bioactive molecules (such as peptides and nucleosides) and unified strategies for bioconjugation using alkynes remain largely unexplored. Motivated by this opportunity, we sought to develop a general and mild strategy that directly leverages alcohols for the synthesis of alkyne-containing compounds. We also aimed to establish a redox-neutral approach that avoids the use of stoichiometric oxidants or reductants and is therefore compatible with a wide range of bioactive molecules. Specifically, we envisioned a general deoxygenative alkynylation strategy designed to achieve three key objectives: (1) enable one-step conversion of complex, bioactive alcohols into click-ready alkynes; (2) establish alkynes as versatile linear linkers for connecting alcohol-derived fragments; and (3) enable conjugation of bioactive molecules with drugs and complex building blocks using alkynes as modular handles (Figure 1).

**Figure 1:**
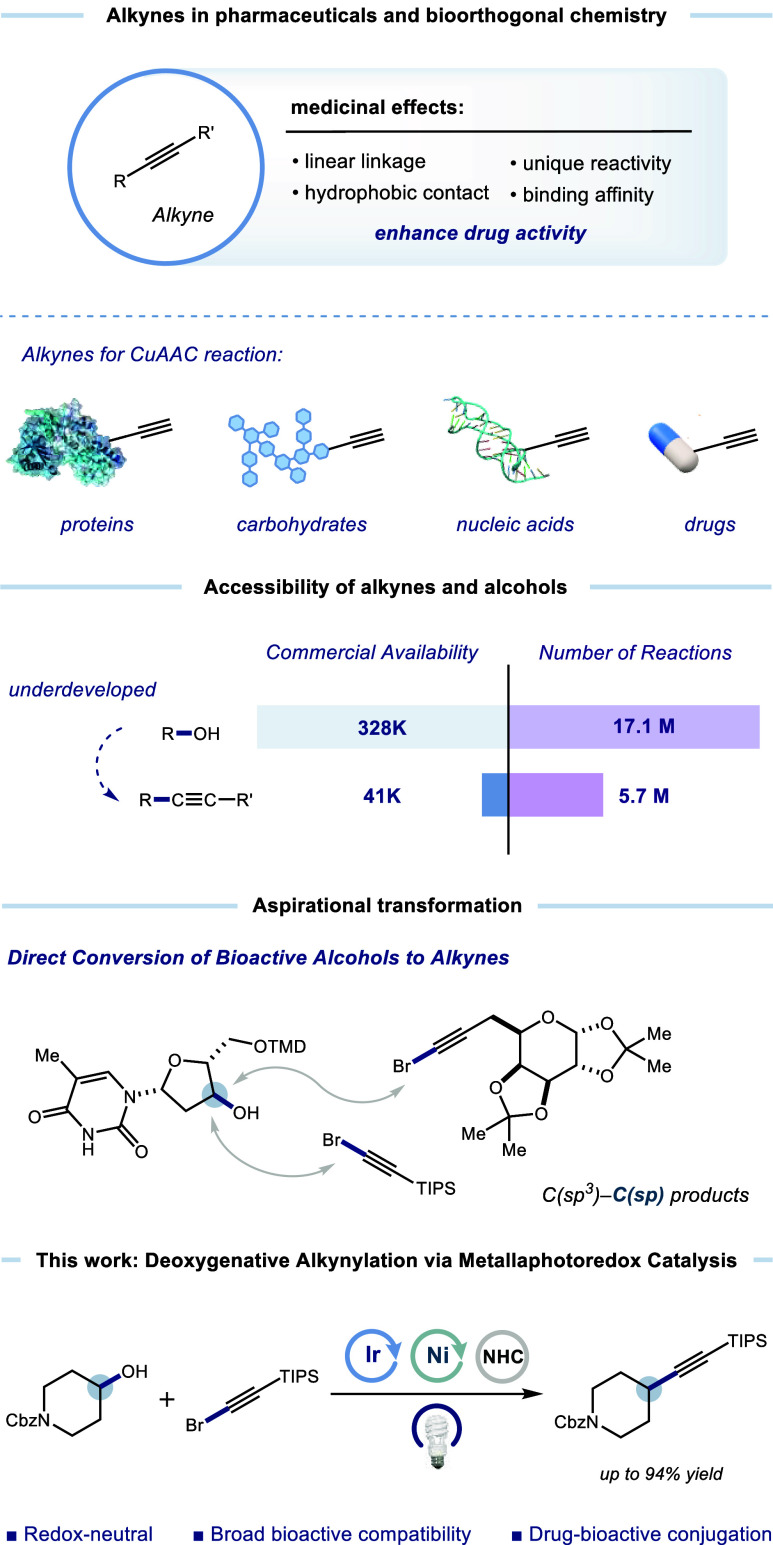
Applications of Alkynes and Deoxygenative Metallaphotoredox C­(*sp* ^*3*^)–C­(*sp*) Coupling.

We began by optimizing a deoxygenative alkynylation protocol to convert alcohols into products derived from terminal alkynes. To achieve redox-neutral conditions, we selected alkynyl bromides as coupling partners, with *N*-Cbz-piperidin-4-ol (**1**) as the limiting alcohol reagent. In a typical procedure, the alcohol was first activated with NHC-1 (1.1 equiv) in MTBE (methyl *tert*-butyl ether), and the resulting mixture was transferred to a solution containing Ni­(dtbbpy)­Br_2_ (10 mol %), quinuclidine (1.5 equiv), phthalimide (1.0 equiv), and the alkynyl bromide (**2**) in DMSO. Under metallaphotoredox conditions with 450 nm irradiation for 2 h, the desired C­(*sp* ^*3*^)–C­(*sp*) coupling product was obtained in 96% assay yield (Figure 2, entry 1). Notably, the di-*tert*-butylbipyridine (dtbbpy) nickel complex proved critical for achieving high efficiency, highlighting the essential role of the metal catalyst in facilitating oxidative addition of the alkynyl bromide and subsequent radical capture (entries 2 and 3). Control experiments further confirmed that photocatalyst, light irradiation and quinuclidine base are required for effective coupling (entries 4, 5 and 6). Moreover, phthalimide was found to be a crucial additive, suppressing decomposition of the nickel oxidative addition complex and enhancing the yield (entry 7).^37^ Notably, a 78% yield was maintained even when the amount of (bromoethynyl)­triisopropylsilane was reduced to 1.2 equiv, further highlighting the efficiency of the protocol (entry 8).

**Figure 2:**
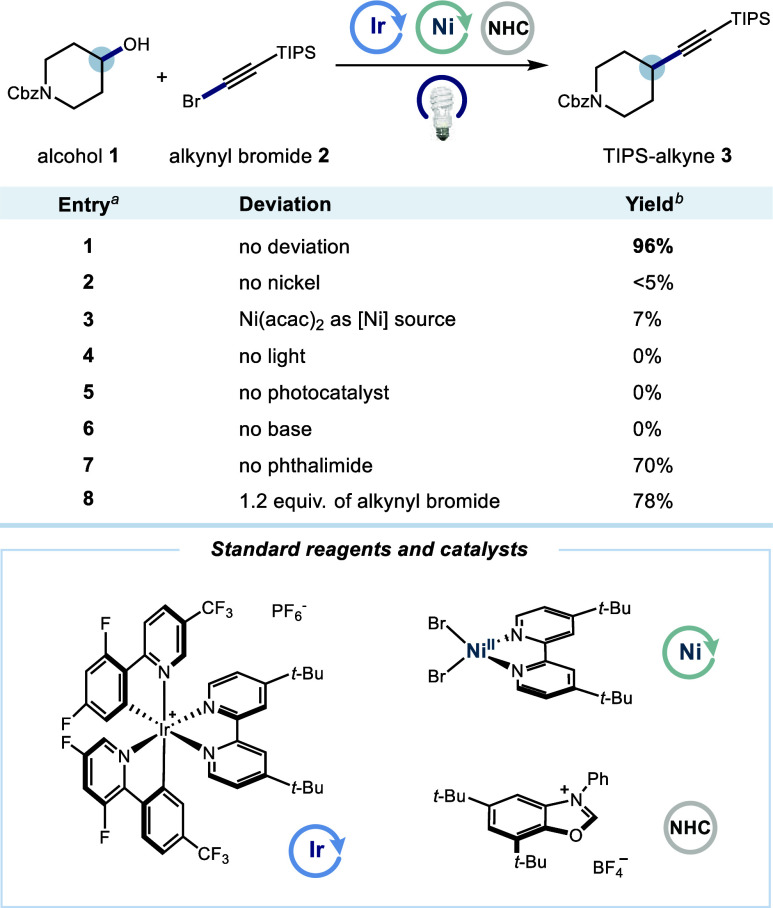
Control Experiments and Optimization for Deoxygenative metallaphotoredox C­(*sp* ^*3*^)–C­(*sp*) coupling. ^*a*^Under standard conditions, reactions performed with alcohol (1.0 equiv), (Bromoethynyl)­triisopropylsilane (alkynyl bromide, 2.5 equiv), NHC-1 (1.1 equiv), pyridine (1.1 equiv), Ni­(dtbbpy)­Br_2_ (10 mol %), (Ir­[dF­(CF_3_)­ppy]_2_(dtbpy))­PF_6_ (1 mol %), phthalimide (1.0 equiv), quinuclidine (1.5 equiv), MTBE/DMSO (1:1, 0.05 M), 450 nm for 2 h. Deviations from standard as shown in entries 2–8. 0.05 mmol scale. ^*b*^Assay yield determined by UPLC-MS analysis. See Supporting Information (SI) for further details. Triisopropyl silane (TIPS).

With the optimized conditions in hand, we next explored the substrate scope of alcohols on a 0.5 mmol scale. (Bromoethynyl)­triisopropylsilane (**2**) was selected as the alkynyl coupling partner due to its commercial availability, low cost, and facile conversion to terminal alkynes via TBAF-mediated deprotection. The reaction exhibited broad tolerance toward a variety of alcohols (Figure 3). Primary amino alcohols, including those bearing β-steric hindrance, afforded the desired products in 70–80% yield (**4**–**6**), and heterocyclic substrates were also well tolerated (**7**, 88% yield). Notably, azide-functionalized PEG linkers were compatible (**8**, 62% yield), highlighting potential bioorthogonal applications. Cyclic secondary alcohols were competent substrates: six-membered piperidine derivatives furnished para- and meta-alkynylated products in excellent yields (**3** and **9**, 71–94%), while four-, five-, and seven-membered ring systems (**10**–**12**) delivered products in 60–82% yield. However, a tertiary alcohol on a six-membered ring afforded only a moderate yield (**13**, 30%),^38^ likely owing to the reduced oxidizability of the NHC–alcohol adduct and increased competing side reactions. Activated alcohols, including those adjacent to esters or benzylic positions, underwent smooth conversion to give α-alkynyl esters (**14**) and benzylic alkynes (**15**) in 60–68% yields. Importantly, other halogen functional groups, including aryl and alkyl chlorides and aryl bromides, did not interfere with the reaction (**16**–**18**, 76–91% yield), highlighting the favorable chemoselectivity of the transformation compared to previous reports.^24,27^ Notably, 3-methylbutane-1,3-diol underwent selective alkynylation at the primary alcohol, leaving the tertiary alcohol untouched (**19**, 72% yield). Complex alcohols bearing multiple substituents were functionalized in good yields and excellent diastereoselectivity (**20**–**22**). In addition, late-stage alkynylation of *trans*-androsterone proceeded in 54% yield (**23**), underscoring the utility of this protocol for complex molecule diversification. The versatility of this reaction is further demonstrated by its tolerance of a wide range of commercially available alkyne precursors. Following efficient conversion to the corresponding alkynyl bromides (in some cases column-free; see SI for details), intermediates were coupled with alcohols to afford alkyne products bearing linear-chain, ether, hydroxyl, ester, and (hetero)­aryl substituents in consistently high yields (**24**–**29**, 65–74%). Notably, alkynyl bromides derived from ketone precursors also proved highly effective (**30**, 83% yield), enabling the merger of alcohol- and ketone-derived fragments. Finally, structurally complex and bioactive alkynes, including those derived from azabicyclo[3.1.0]­hexane (**31**) and the natural product *menthone* (**32**), were coupled in high yields (63% and 86%, respectively).

**Figure 3:**
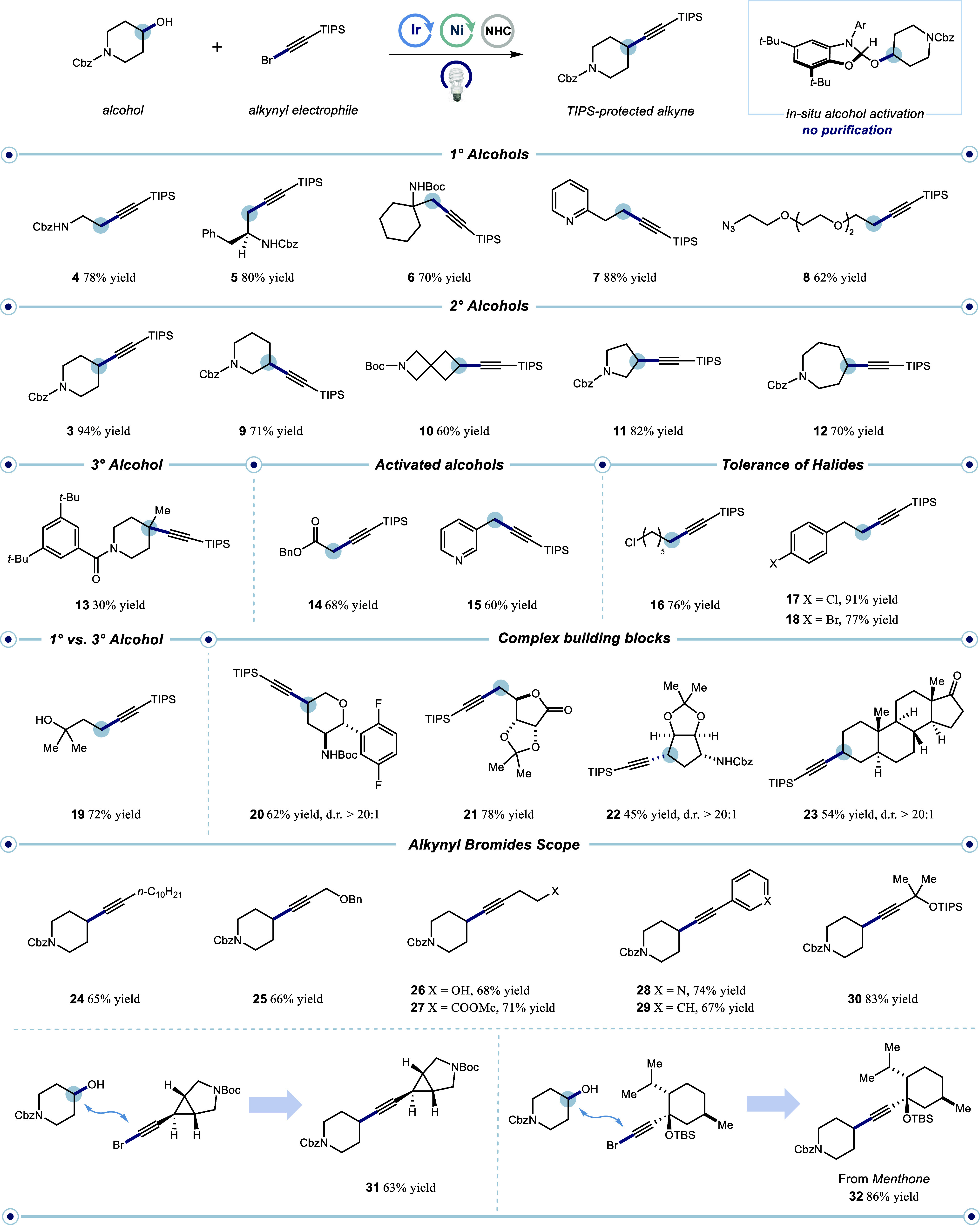
Substrate Scope for Deoxygenative C­(sp^3^)–C­(sp) Coupling. NHC-1: Ar = Ph (for 1° and 2° alcohol); NHC-2: Ar = *p*-CF_3_–Ph (for 3° alcohol). See Supporting Information for experimental details.

Encouraged by the broad tolerance for diverse substrates, we next sought to access click-active, bioactive alkynes. Among the major classes of naturally occurring biomolecules—carbohydrates, lipids, nucleic acids, and proteins—hydroxyl groups are ubiquitous in both the macromolecules and their monomeric units,^39^ making them ideal handles for late-stage functionalization. Accordingly, we investigated the direct conversion of these hydroxyl groups into TIPS-protected terminal alkynes. Carbohydrate-derived substrates, including those from galactopyranose and mannofuranose, were efficiently transformed to the corresponding alkynes in high yields and excellent diastereoselectivity (**33**–**34**, 67–82% yield, > 20:1 d.r.; **35**, 82% yield). Lipid-derived substrates, such as difatty acid-substituted glycerol, were also well tolerated, affording the desired product in 65% yield (**36**). Amino acid derivatives from serine, threonine, and proline underwent smooth alkynylation in excellent yields (**37**–**39**, 73–84%). Notably, dipeptides and tripeptides bearing diverse functional side chains, including imidazole, ester, and indole groups, were selectively functionalized at the serine hydroxyl moiety in good yields (**40**–**42**, 61–80%). Furthermore, nucleoside substrates such as thymidine underwent direct deoxygenative alkynylation to afford the desired product in 67% yield with high diastereoselectivity (**43**); subsequent deprotection delivered a synthetically valuable 3′-alkynyl nucleoside in 95% yield (**44**). Other nucleosides, including cytidine, guanosine, and uridine, were also compatible, providing moderate to good yields (**45**–**47**, 24–67%), whereas adenosine showed lower reactivity (10% yield), likely due to challenges in alcohol activation (see SI for details). RNA-derived uridine substrates were also tolerated, albeit in lower yield (**48**, 17%). Finally, the method proved effective for the late-stage functionalization of hydroxyl-containing pharmaceutical molecules. Drug-like substrates, including derivatives of zidovudine, RU-58841, abiraterone, and nadifloxacin, underwent efficient alkynylation (**49**–**52**). Cross-coupling also delivered analogues of ataluren (**53**, 63%), febuxostat (**54**, 65%), and loxoprofen (**55**, 82%). These examples demonstrate excellent tolerance toward a wide range of pharmaceutically relevant functional groups, including azides, nitriles, alkenes, and diverse heterocycles, highlighting the broad applicability of this transformation for the synthesis of structurally diverse, click-ready alkyne derivatives across complex biological and pharmaceutical contexts (Figure 4).

**Figure 4:**
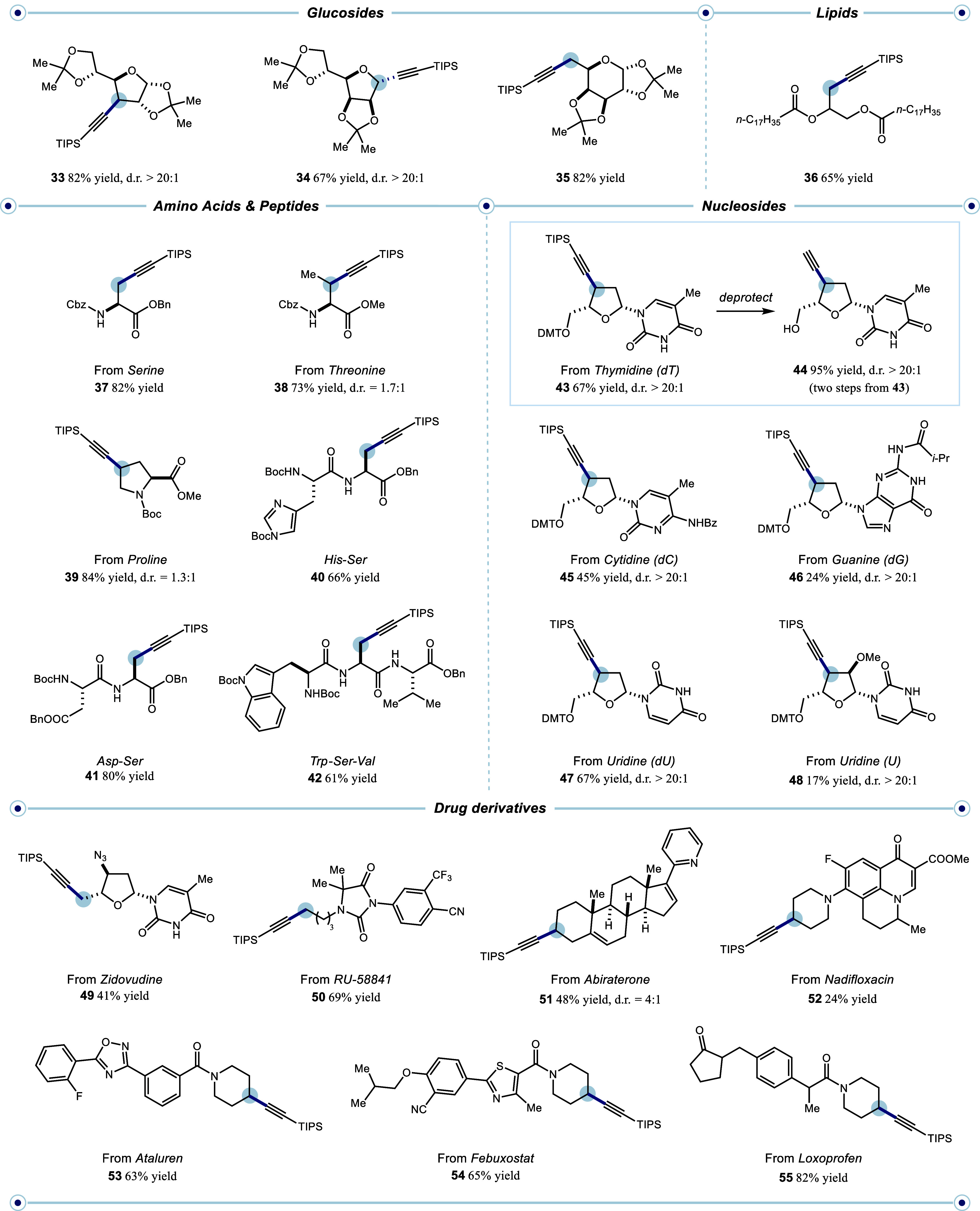
Scope of Bioactive Alcohols and Drug-related Substrates. See Supporting Information for experimental details.

Given the unique role of alkynes as linear linkers between bioactive molecules, we next explored a sequential deoxygenative coupling strategy to connect two alcohol-derived fragments. As outlined in Figure 5A, deoxygenative alkynylation of a complex alcohol first furnishes the corresponding terminal alkyne, which can be converted to an alkynyl bromide in excellent yield, enabling a second metallaphotoredox coupling. Subsequent coupling of a six-membered amino alcohol with an alkynyl bromide derived from a primary amino alcohol (**56**) afforded the corresponding internal alkyne (**57**) in 52% yield, demonstrating the feasibility of this workflow. As a natural extension, we next explored applications in bioconjugation. In an initial demonstration, a glucofuranose-derived alkynyl bromide (**58**) was successfully cross-coupled with the hydroxyl group of serine (**59**, 64%), thereby expanding the accessible chemical space for amino acid-sugar conjugation. Encouraged by this result, we conducted a large-scale experiment to access preparative-scale quantities of a sugar-derived alkyne building block. Under 2x concentration on a 3 mmol scale, a Galactopyranose derivative reacted smoothly to afford the corresponding terminal alkyne product in 68% overall yield (**60**). Importantly, TBAF deprotection could be performed directly on the crude mixture obtained from the photoredox step. Following conversion to alkynyl bromide **61** in quantitative yield, cross-coupling with *N*-Cbz-piperidin-4-ol afforded the desired product in 67% yield (**62**). Notably, serine- and thymidine-derived substrates could also be efficiently coupled with alkynyl sugars, affording the internal alkynes in 64% (**63**) and 52% (**64**) yield, respectively, thereby broadening the scope of bioconjugation applications. Finally, we demonstrated the synthesis of terminal alkynes for drug–biomolecule conjugation. Notably, zidovudine–amino acid and RU-58841–biotin conjugates were successfully prepared via click chemistry in good yields (**65**–**68**), highlighting the potential of this approach to provide versatile bioactive handles for applications in drug discovery (Figure 5B).

**Figure 5:**
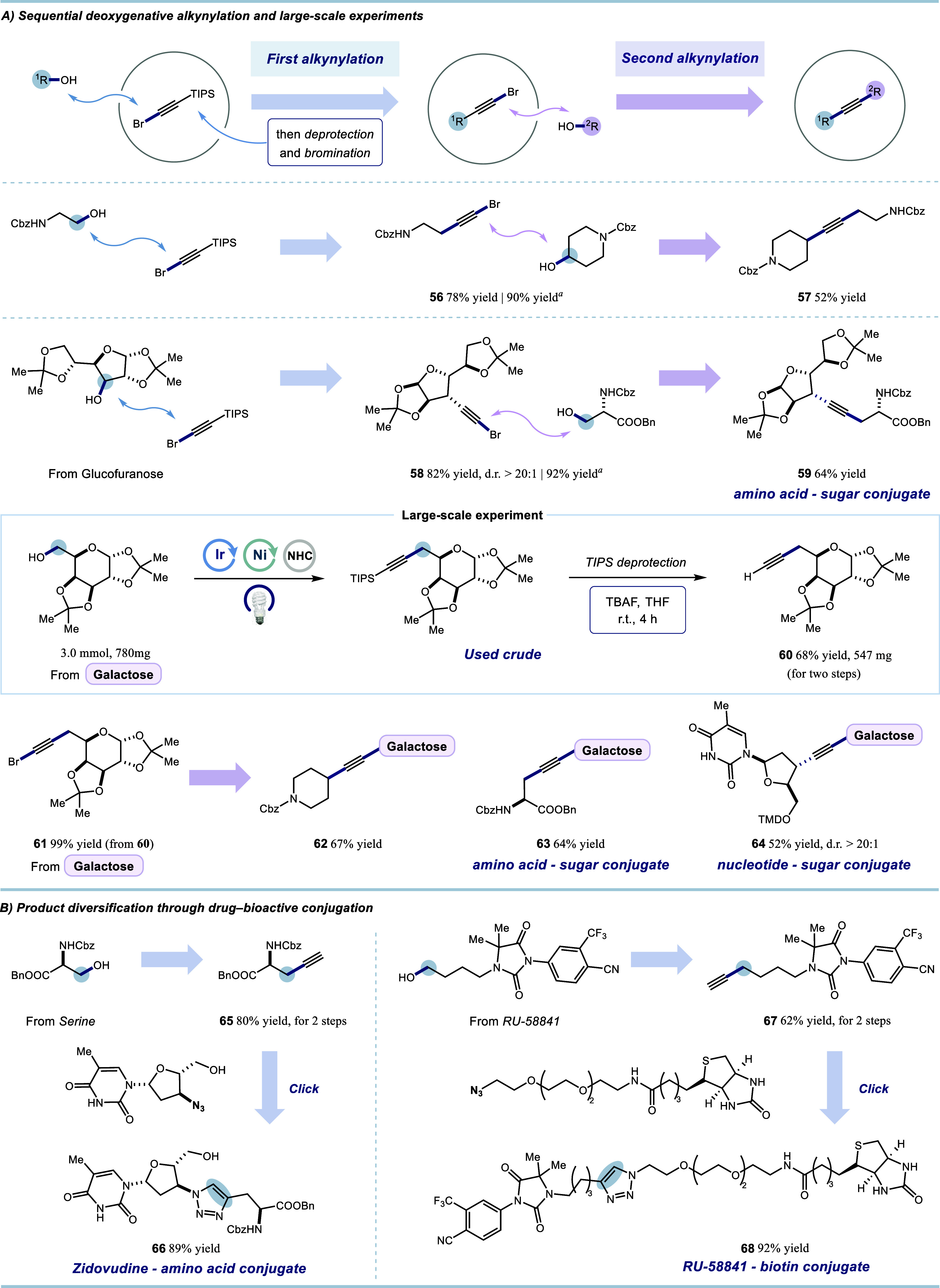
Synthetic Applications and Bioconjugation. ^*a*^Yields indicate the yield of the initial deoxygenative alkynylation step and the yield for alkynyl bromide regeneration (combination of TIPS deprotection and bromination). See Supporting Information for experimental details.

To further demonstrate the utility of this protocol for peptide-conjugate therapeutics, we explored direct installation of alkyne-containing drug moieties onto amino acid side chains via cross-coupling of serine-derived hydroxyl groups with alkynyl bromides. Remarkably, alkyne-containing drugs derived from norethindrone acetate, rasagiline, and mestranol were successfully installed onto amino acid side chains (**69**–**72**, 63–65% yield), thereby enabling streamlined amino acid–pharmacophore conjugation (Figure 6).

**Figure 6:**
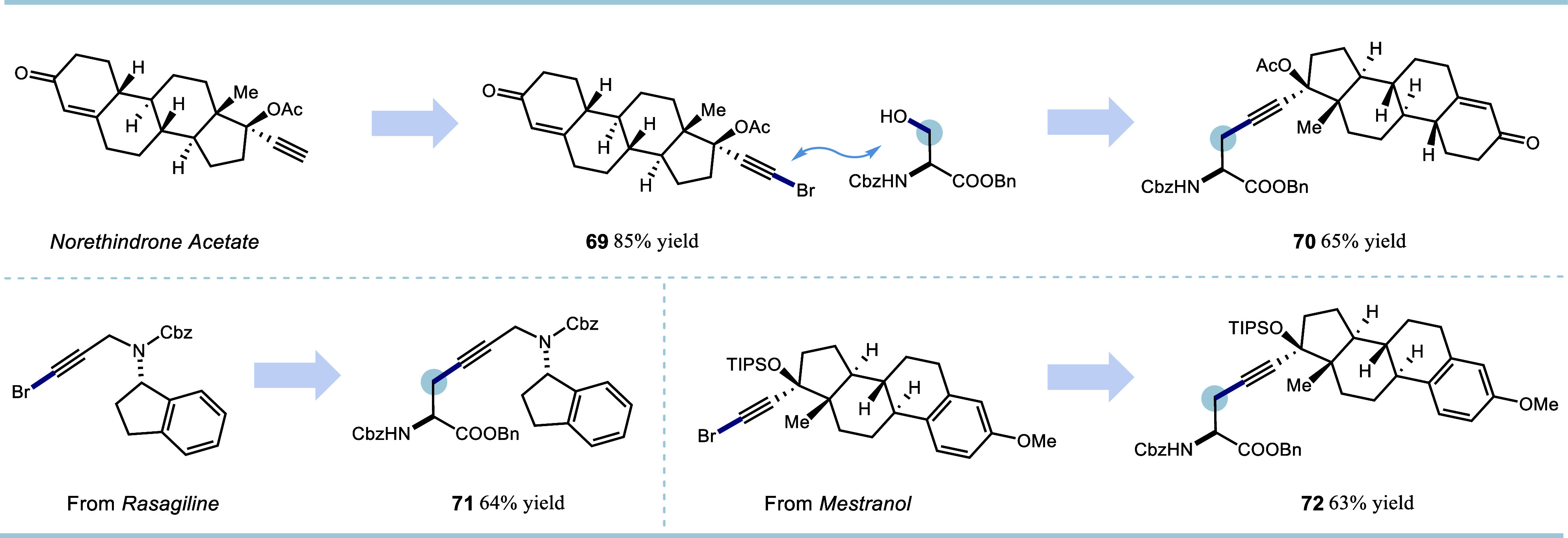
Drug-Bioactive Conjugation.

In summary, we have developed a general, mild, and redox-neutral deoxygenative alkynylation strategy that directly converts abundant alcohols into valuable alkyne-containing compounds via metallaphotoredox C­(*sp* ^*3*^)–C­(*sp*) cross-coupling. *In situ* activation of alcohols with NHC reagents avoids the need for intermediate purification. The method exhibits broad substrate scope and excellent functional group tolerance, enabling the efficient transformation of diverse alcohols, including bioactive molecules and pharmaceuticals, into click-ready terminal alkynes. Its scalability and compatibility with sequential coupling processes further highlight its synthetic utility, enabling the assembly of structurally complex architectures and bioconjugates. Overall, this work establishes alcohols as practical and general building blocks for alkyne installation and offers a unified approach to access structurally diverse, functionally rich alkyne derivatives.

## Supplementary Material



## Notes

Additional experimental details, optimization, additional substrate tables, compound characterization, and spectra (PDF)
