# Supplementary material for: A Unified Platform for Direct Alkynylation from Alcohols via Deoxygenative Metallaphotoredox Catalysis
Source: J Am Chem Soc. 2026 Jul 10;148(28):29655–62. doi: 10.1021/jacs.6c10795 (PMC13397560; doi:10.1021/jacs.6c10795)
Supplement: Supplementary file 1 [file ja6c10795_si_001.pdf]

Supplementary Information

A Unified Platform for Direct Alkynylation from Alcohols via Deoxygenative Metallaphotoredox Catalysis

Yi-Hao Li and David W. C. MacMillan†

Merck Center for Catalysis at Princeton University, Princeton, New Jersey 08544, USA

†Corresponding author. Email: dmacmill@princeton.edu

Table of Contents

1) General information 3

2) Standard reaction setup 5

3) Reaction optimization and control reactions 6

4) General procedures for deoxygenative alkynylation 14

5) Ineffective examples 19

6) Large scale experiment 20

7) Reaction time course studies 22

8) Proposed mechanism 23

9) Experimental and characterization data 24

10) Spectral data 111

11) Citations 200

1) General information

Commercial reagents were used without prior purification unless otherwise indicated. All solvents were purified according to the method of Grubbs.[1] Alcohol activation N-heterocyclic carbene reagents (**NHC)** were prepared according to literature procedures.[2] Organic solutions were concentrated under reduced pressure on a Büchi rotary evaporating using a temperature-controlled water bath.

Chromatographic purification of products was performed on an automated Teledyne ISCO Combi*Flash*® NextGen 300+ system using RediSep Rf Gold® Silica Gel Disposable Flash Columns (20–40 microns). Reverse phase chromatography was performed on a Teledyne ISCO ACCQPrep® HP150 system using Waters XBridge BEH C18 OBD Prep Column (30 mm × 150 mm, 130 Å, 5 μm) with 0.1% ammonium hydroxide buffered water and acetonitrile solutions. Thin-layer chromatography (TLC) was performed on Silicycle 0.25 mm or Supelco 0.20 mm silica gel F-254 plates. Visualization of the developed chromatogram was performed by fluorescence quenching and $KMnO_4$ stain.

^{1}H and ^{13}C NMR spectra were recorded on a Bruker Avance III NMR 500 MHz instrument, Bruker NanoBay Avance III HD NMR 400 MHz instrument, or a Bruker NanoBay Avance III NMR 300 MHz and are internally referenced to the residual proteo-solvent (CDCl3 referenced at 7.26 ppm and 77.16 ppm, respectively; Acetone-*d6* referenced at 2.05 ppm and 29.84 ppm respectively; Acetonitrile-*d3* referenced at 1.94 ppm and 118.26 ppm respectively). ^{19}F NMR spectra were recorded on a Bruker NanoBay Avance III HD NMR 400 MHz and Bruker NanoBay Avance III NMR 300 MHz are reported unreferenced. ^{1}H and ^{19}F NMR are reported as follows: chemical shift (δ ppm), multiplicity (s = singlet, d = doublet, t = triplet, q = quartet, p = pentet, h = hextet, hept = heptet, m = multiplet, b = broad), coupling constant (Hz),

and integration. Data for ^{13}C NMR are reported in terms of chemical shift; multiplicity and coupling constants are included only in the case of coupling with ^{19}F nuclei.

Liquid chromatography (LC) analysis was performed on an Agilent 1200 or Agilent 1290 Infinity II LC system. High resolution mass spectra (HRMS) were obtained from the Princeton University Mass Spectral Facility on Agilent 6220 ESI-TOF LC/MS or Agilent 7200 GC-QTOF systems.

Commercial availability data for alkenes and alkynes were obtained from Reaxys (accessed May 13th., 2026). Search criteria were defined as follows: alcohols include mono-, di- or tri- substitution at the α-position of the hydroxy group, and alkynes included mono- and di-substitution, with a unit price below USD 500 and a minimum package size of 5 mg. Under these conditions, 327,892 commercially available alcohols and 40,744 alkynes were identified. Reaction number data were collected from SciFinder (accessed May 13th, 2026). Alcohol reactions were obtained from reactions involving substrates with mono-, di- or tri- substitution at the α-position of the hydroxy group, with "Mapping Data Available" applied, yielding 17,109,553 reactions. Alkyne reactions were involving alkynes substrates included mono- and di-substitution, filtered by "Mapping Data Available", yielding 5,658,032 reactions.

Blue bold bonds indicate newly formed bonds. When attached to stereogenic centers, they also denote the relative stereochemistry. The reported d.r. values were determined either by isolating and individually weighing the separated diastereomers or, when complete separation was not practical, by collecting the combined product and determining the d.r. from the ^{1}H NMR spectrum of the purified material.

2) Standard reaction setup

All 0.2 or 0.5 mmol scale were run in a 40 mL vial and irradiated in the M2 PennOC Integrated Photoreactor[2] using a M2 450 nm LED module, 100% light intensity, 6800 rpm fans, and 600 rpm stirring. All 0.05 mmol reactions were run in an 8 mL vial and irradiated in the M2 PennOC Integrated Photoreactor using a M2 450 nm LED module, 100% light intensity, 6800 rpm fans, and 1000 rpm stirring.

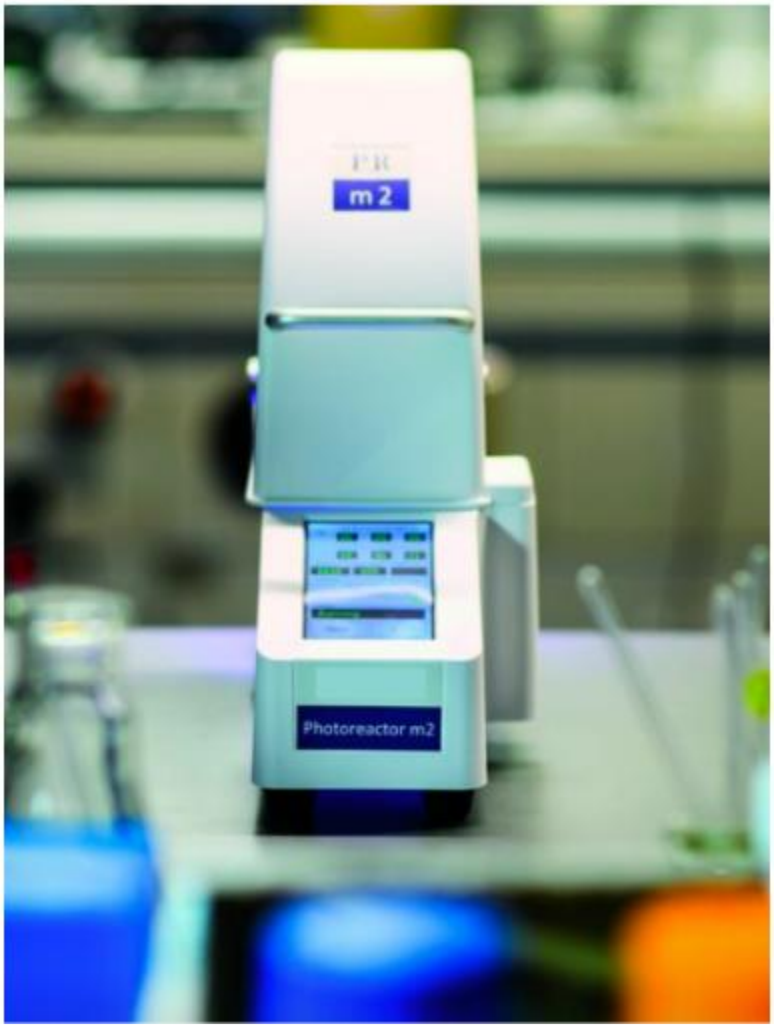

Penn PhD Photoreactor M2

3) Reaction optimization and control reactions

General Procedure for Optimization

All optimizations were performed at 0.05 mmol scale unless otherwise noted.

A 40-mL vial was charged with an adequate amount of photocatalyst, metal source, and additive. MeCN was introduced under ambient conditions, and the mixture was sonicated until all solids dissolved and distributed at this stage into 8 mL **reaction vials** for multiple reaction set-ups. The resulting cloudy solution was then concentrated *via* Genevac at 40°C until all liquid was removed. The vial was then charged with a magnetic stir bar, quinuclidine and solvent.

Stock solution A (activated alcohol): A 40-mL vial was charged with alcohol (1 equiv.), NHC (1.1 equiv.), and an X-shaped stir bar. MTBE (0.1 M) was added via syringe under air. This mixture was stirred vigorously for 1 minute before pyridine (1.1 equiv.) was added slowly via syringe. The heterogeneous mixture was vigorously stirred at room temperature for 40 minutes. During this time a white solid precipitated out, and the mixture turned from white to pale orange to pale pink. The suspension was filtered to give **Stock solution A** and distributed for multiple reaction set-ups.

The **reaction vial** was opened to air to introduce **Stock Solution A** and alkynyl bromide with a syringe. The final reaction mixture was then sparged with N_2 for ~1.5 minutes before it was irradiated with 450 nm LED modules at 100% light intensity with maximum fan speed and 1000 rpm stir rate in a PennOC or PennPhD Integrated Photoreactor.

Upon completion of the reaction, an internal standard (acetanilide) was added to the reaction mixture. An aliquot was subjected to UPLC analysis and assay yield was determined by

calculation based of response factor obtained by measurements of the pure product standard.

Table S1. Solvents screen

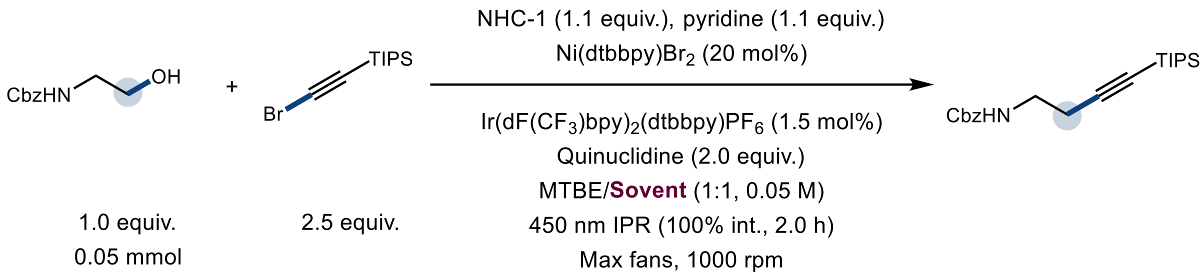

Entry	Sovent	Yield
**1**	DMSO	**70%**
**2**	DMF	59%
**3**	MeCN	40%
**4**	NMP	64%
**5**	^{t}Amyl-OH	39%
**6**	Acetone	48%

Table S2. Nickel catalysts loading screen.

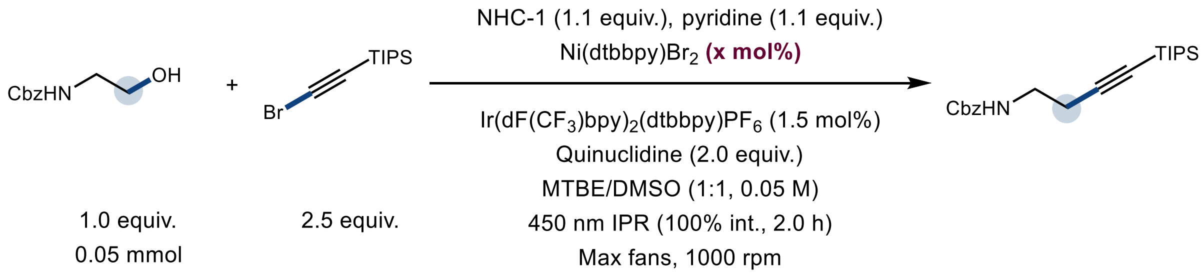

Entry	$Ni(dtbbpy)Br_2$ (x mol%)	Yield
**1**	0 mol%	<5%
**2**	5 mol%	56%
**3**	10 mol%	**63%**
**4**	15 mol%	65%
**5**	20 mol%	70%
**6**	25 mol%	65%

Since the yield remained similar at 10 mol% [Ni], we used 10 mol% [Ni] instead of 20 mol%.

Table S3. Base additive screen.

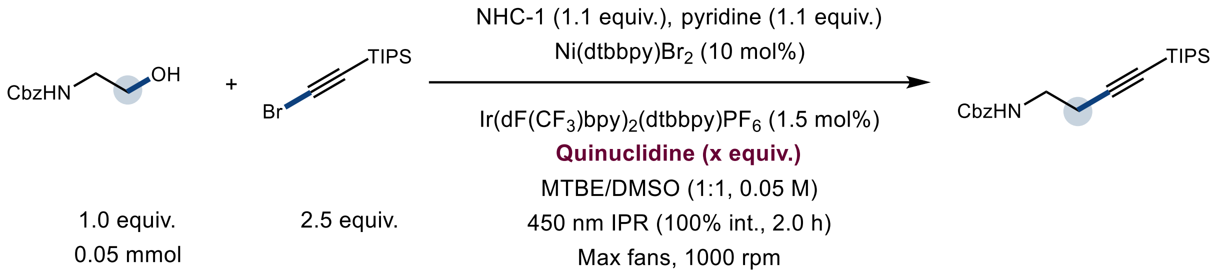

Entry	Quinuclidine (x equiv.)	Yield
**1**	0 equiv.	N.R.
**2**	1.0 equiv.	67%
**3**	1.5 equiv.	**71%**
**4**	2.0 equiv.	63%
**5**	3.0 equiv.	69%
**6**	LiOAc (2.0 equiv.) instead of Quinuclidine	N.R.
**7**	NaOAc (2.0 equiv.) instead of Quinuclidine	N.R.
**8**	KOAc (2.0 equiv.) instead of Quinuclidine	N.R.
**9**	CsOAc (2.0 equiv.) instead of Quinuclidine	<5%

Table S4. Imide additive screen.

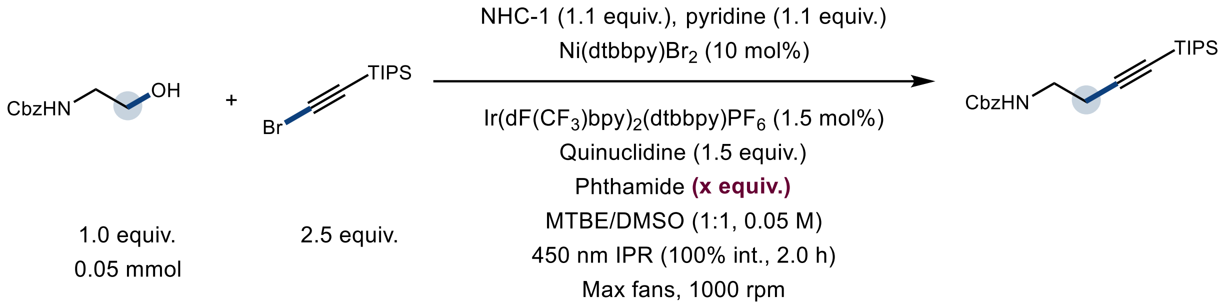

Entry	Phthamide (x equiv.)	Yield
**1**	0 equiv.	71%
**2**	0.5 equiv.	74%
**3**	1.0 equiv.	**82%**

Table S5. Photocatalysis screen.

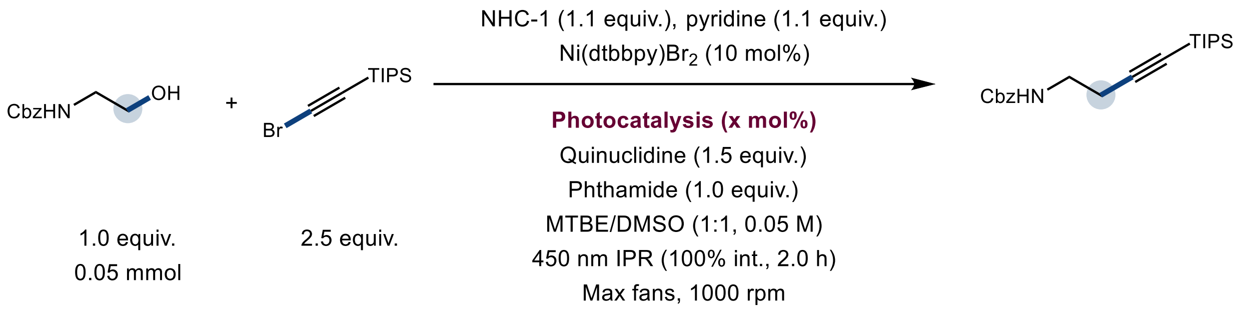

Entry	Photocatalysis (x mol%)	Yield
**1**	Ir(dF(CF$_3$)bpy)$_2$(dtbbpy)PF$_6$ (1.5 mol%)	82%
**2**	Ir(dF(CF$_3$)bpy)$_2$(dtbbpy)PF$_6$ (1.0 mol%)	**82%**
**3**	Ir(bpy)$_2$(dtbbpy)PF$_6$ (1.0 mol%)	72%
**4**	Ir(dF(Me)bpy)$_2$(dtbbpy)PF$_6$ (1.0 mol%)	79%
**5**	4-CzIPN (5.0 mol%)	75%

Table S6. Alkynyl bromide loading screening for general alkynyl bromide substrates.

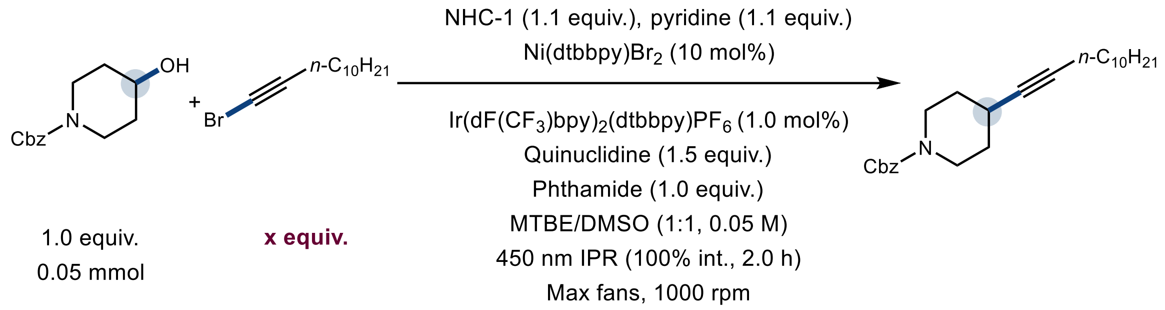

Entry	alkynyl bromide (x equiv.)	Yield
**1**	0.8 equiv.	41%
**2**	1.0 equiv.	45%
**3**	1.2 equiv.	48%
**4**	1.5 equiv.	**56%**
**5**	1.8 equiv.	56%

Table S7. Base additive screening for general alkynyl bromide substrates.

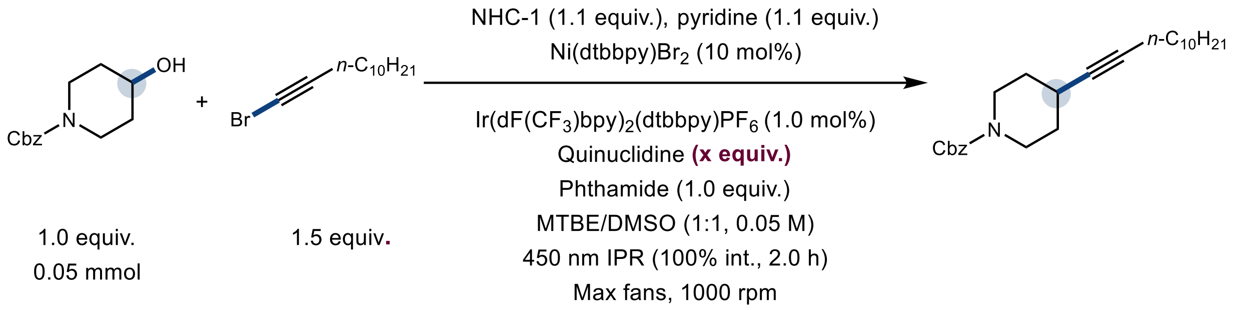

Entry	Quinuclidine (x equiv.)	Yield
**1**	1.2 equiv.	47%
**2**	1.5 equiv.	56%
**3**	2.0 equiv.	**59%**
**4**	2.5 equiv.	55%
**5**	3.0 equiv.	56%

Table S8. Imide additive screening for general alkynyl bromide substrates.

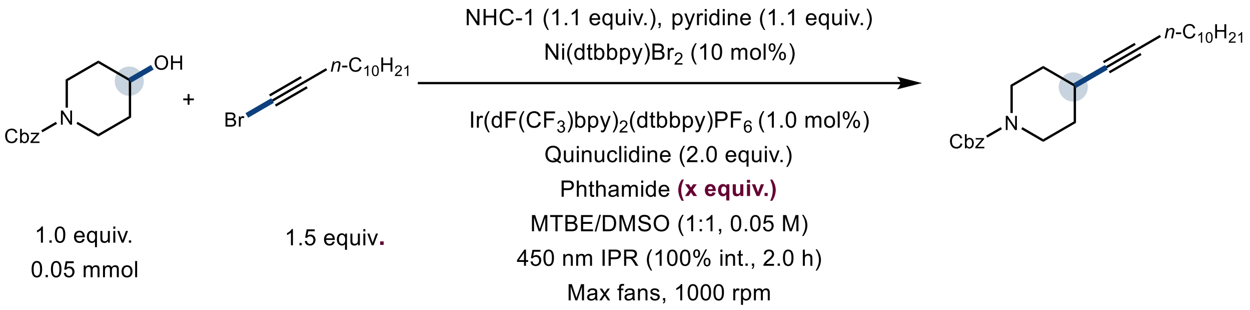

Entry	Phthamide (x equiv.)	Yield
**1**	0.8 equiv.	49%
**2**	1.0 equiv.	56%
**3**	1.5 equiv.	62%
**4**	2.0 equiv.	**71%**
**5**	2.5 equiv.	70%

Table S9. Control reactions

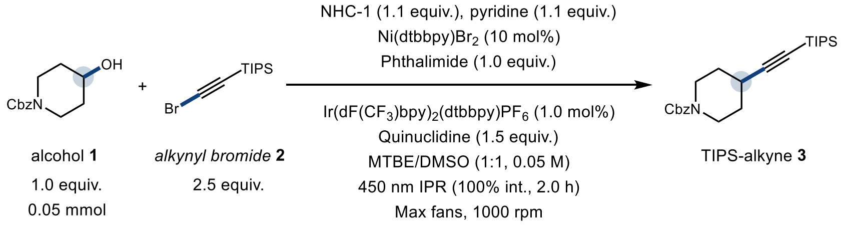

Entry	Deviation	Yield
**1**	no deviation	**96%**
**2**	no light	0%
**3**	no photocatalyst	0%
**4**	no nickel	<5%
**5**	Ni(acac)$_2$ as [Ni] source	7%
**6**	no phthalimide	70%
**7**	no base	0%

4) General procedures for deoxygenative alkynylation

General Procedure A (for (bromoethynyl)triisopropylsilane):

Stock solution A (activated alcohol): A 40-mL vial was charged with alcohol (1 equiv., *if solid*), NHC-1 (1.1 equiv.), and an X-shaped stir bar. MTBE (0.1 M) was added via syringe under air. This mixture was stirred vigorously for 1 minute before pyridine (1.1 equiv.) was added slowly via syringe. The heterogeneous mixture was vigorously stirred at room temperature for 40 minutes. During this time a white solid precipitated out, and the mixture turned from white to pale orange to pale pink. The suspension was filtered to give **Stock solution A** and distributed for multiple reaction set-ups. Such solution can be prepared at 0.2 to 3.0 mmol scale and distributed for multiple reaction set-ups.

Reaction vial (for 0.5 mmol scale, alcohol limiting): A 40-mL vial was charged with a magnetic stir bar, $Ir(dF(CF_3)ppy)_2(dtbbpy)PF_6$ (5.6 mg, 5.0 μmol, 1 mol%), $Ni(dtbbpy)Br_2$ (24.4 mg, 0.05 mmol, 10 mol%), phthalimide (73.5 mg, 0.5 mmol, 1.0 equiv.), quinuclidine (83.2 mg, 0.75 mmol, 1.5 equiv.), DMSO (5 mL). The mixture was sonicated until all solids dissolved. Such solution can be prepared at a larger scale and distributed at this stage to individual **reaction vials** for multiple reaction set-ups. The **reaction vial** was opened to air to introduce **Stock Solution A** and alkynyl bromide (2.5 equiv., 1.25 mmol, 326.3 mg, 304 μL) with a syringe. The final reaction mixture was then sparged with N_2 for ~1.5 minutes before it was irradiated with 450 nm LED modules at 100% light intensity with maximum fan speed and 600 rpm stir rate in a PennOC or PennPhD Integrated Photoreactor for 2.0 hours.

After completion, the reaction mixture was diluted with 50 mL of water and extracted with ethyl acetate (3 × 25 mL). The combined organic layers were dried over sodium sulfate, concentrated, and purified by column chromatography, followed by preparative HPLC if necessary.

Note*: If the primary or secondary alcohol is a liquid or oil, it was added as a solution in MTBE (5 mL) or introduced after MTBE and before pyridine. If alcohol contains basic hetereocycles, NHC was introduced at last (after pyridine) as a solid under air.*

General Procedure B (for other alkynyl bromides):

Stock solution A (activated alcohol): A 40-mL vial was charged with alcohol (1 equiv., *if solid*), NHC-1 (1.1 equiv.), and an X-shaped stir bar. MTBE (0.1 M) was added via syringe under air. This mixture was stirred vigorously for 1 minute before pyridine (1.1 equiv.) was added slowly via syringe. The heterogeneous mixture was vigorously stirred at room temperature for 40 minutes. During this time a white solid precipitated out, and the mixture turned from white to pale orange to pale pink. The suspension was filtered to give **Stock solution A** and distributed for multiple reaction set-ups. Such solution can be prepared at 0.2 to 1.0 mmol scale and distributed for multiple reaction set-ups.

Reaction vial (for 0.5 mmol scale, alcohol limiting): A 40-mL vial was charged with a magnetic stir bar, $Ir(dF(CF_3)ppy)_2(dtbbpy)PF_6$ (5.6 mg, 5.0 μmol, 1 mol%), $Ni(dtbbpy)Br_2$ (24.4 mg, 0.05 mmol, 10 mol%), phthalimide (147 mg, 1.0 mmol, 2.0 equiv.), quinuclidine (111 mg, 1.0 mmol, 2.0 equiv.), DMSO (5 mL). The mixture was sonicated until all solids dissolved. Such solution can be prepared at a larger scale and distributed at this stage to individual **reaction vials** for multiple reaction set-ups. The **reaction vial** was opened to air to introduce **Stock Solution A** and alkynyl

bromide (1.5 equiv., 0.75 mmol) with a syringe. The final reaction mixture was then sparged with N_2 for ~1.5 minutes before it was irradiated with 450 nm LED modules at 100% light intensity with maximum fan speed and 600 rpm stir rate in a PennOC or PennPhD Integrated Photoreactor for 2.0 hours.

After completion, the reaction mixture was diluted with 50 mL of water and extracted with ethyl acetate (3 × 25 mL). The combined organic layers were dried over sodium sulfate, concentrated, and purified by column chromatography, followed by preparative HPLC if necessary.

General Procedure C (TIPS-deprotection) To a 40 mL reaction vial, TIPS-protected alkynes (1.0 equiv., 1.0 mmol) and 1.0 M solution of TBAF in THF (1.05 mL, 1.05 mmol) were added and the mixture was diluted with THF (5.0 mL). The reaction mixture was stirred at room temperature for 2-4 h. The reaction mixture then was concentrated under vacuum and subjected to column chromatography on silica gel (eluent: hexanes/EtOAc) to afford the desired desilylated product.

General Procedure D (preparation of alkynyl bromides from alkynes): To a solution of alkyne (1 mmol) in acetone (5 mL) was added *N*-bromo succinimide (195 mg,1.1 mmol) and silver nitrate (8.5 mg, 0.05 mmol) at room temperature. The solution was stirred for 4 hours at room temperature in darkness. The reaction was quenched with water (5 mL) and extracted with DCM (10 mL) for three times. The combined organic layers were dried with sodium sulfate and concentrated. Crude residue was purified by column chromatography to give desired alkynyl bromide.

General Procedure E (for tertiary alcohols):

Stock solution A (activated alcohol): A 40-mL vial was charged with tertiary alcohol (1 equiv., *if solid*), NHC-2 (1.1 equiv.), and an X-shaped stir bar. $PhCF_3$ (0.1 M) was added via syringe under air. This mixture was stirred vigorously for 1 minute and cooled to -25˚C before pyridine (1.1 equiv.) was added slowly via syringe. The heterogeneous mixture was vigorously stirred at -25–0 ˚C for 2 hours. During this time a white solid precipitated out, and the mixture turned from white to orange. The suspension was filtered to give **Stock solution A** and distributed for multiple reaction set-ups.

Reaction vial (for 0.5 mmol scale, alcohol limiting): A 40-mL vial was charged with a magnetic stir bar, $Ir(dF(CF_3)ppy)_2(dtbbpy)PF_6$ (5.6 mg, 5.0 μmol, 1 mol%), $Ni(TMHD)_2$ (21.3 mg, 0.05 mmol, 10 mol%), quinuclidine (111 mg, 1.0 mmol, 2.0 equiv.), DMSO (5 mL). The mixture was sonicated until all solids dissolved. Such solution can be prepared at a larger scale and distributed at this stage to individual **reaction vials** for multiple reaction set-ups. The **reaction vial** was opened to air to introduce **Stock Solution A** and alkynyl bromide (2.5 equiv., 1.25 mmol) with a syringe. The final reaction mixture was then sparged with N_2 for ~1.5 minutes before it was irradiated with 450 nm LED modules at 100% light intensity with maximum fan speed and 600 rpm stir rate in a PennOC or PennPhD Integrated Photoreactor for 2.0 hours.

After completion, the reaction mixture was diluted with 50 mL of water and extracted with ethyl acetate (3 × 25 mL). The combined organic layers were dried over sodium sulfate, concentrated, and purified by column chromatography, followed by preparative HPLC if necessary.

General Procedure F (for click coupling):

Terminal alkyne (0.1 mmol) and azides (0.1 mmol) were suspended in a 1:1 mixture of water and ^{t}BuOH (0.5 mL). To the mixture, sodium ascorbate (19.8 mg, 0.1 mmol) and $CuSO_4{\cdot}5H_2O$ (2.5 mg, 0.01 mmol) were added to the solution. The heterogeneous mixture was stirred vigorously at room temperature and monitored with TLC until the reaction completion. Then, the reaction mixture was extracted with ethyl acetate, and the organic phase was concentrated under vacuum. The obtained residue was subjected to column chromatography on silica gel (eluent: hexanes/EtOAc) or preparative HPLC to afford the desired cycloaddition product.

5) Ineffective examples

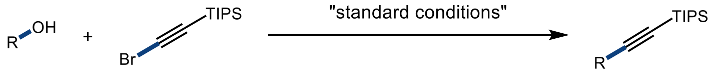

Ineffective alcohols:

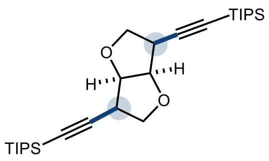

26% yield, d.r. > 20:1

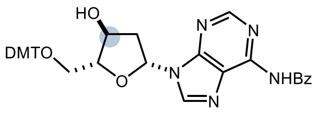

From *Adenosine (dA)*

10% yield

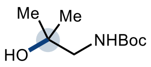

N.R.

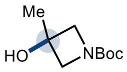

trace

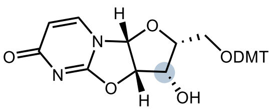

N.R.

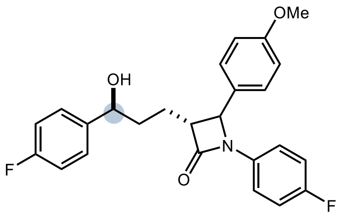

N.R.

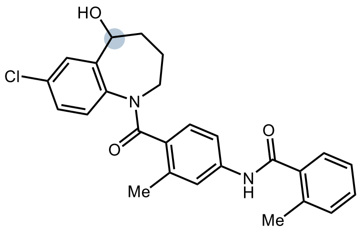

N.R.

Scheme S1. Ineffective alcohols.

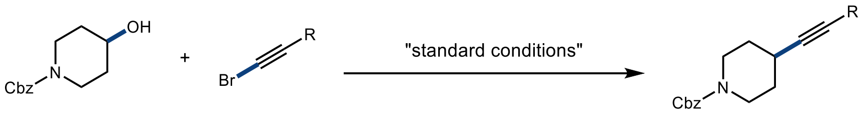

Ineffective alkynyl bromides:

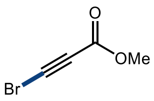

N.R.

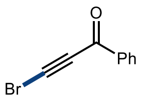

N.R.

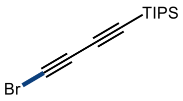

N.R.

Scheme S2. Ineffective alkynyl bromides.

6) Large scale experiment

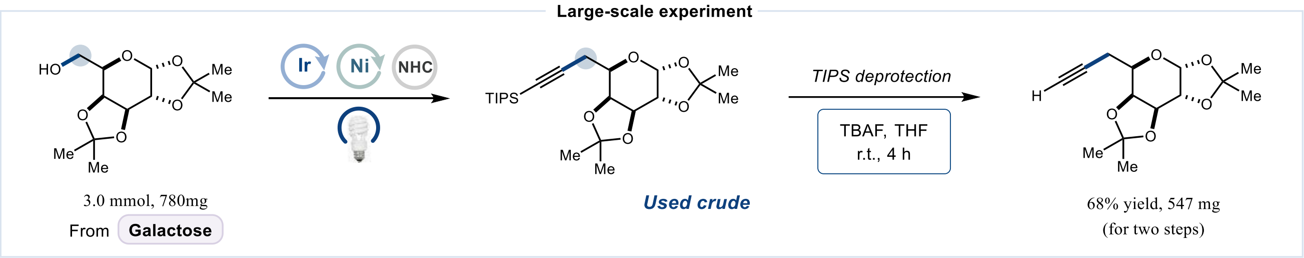

Stock solution A (activated alcohol): A 40-mL vial was charged with 1,2:3,4-Di-O-isopropylidene-D-galactopyranose (780mg, 3 mmol, 1 equiv.), NHC-1 (1.30 g, 3.3 mmol, 1.1 equiv.), and an X-shaped stir bar. MTBE (15 mL) was added via syringe under air. This mixture was stirred vigorously for 1 minute before pyridine (267 μL, 3.3 mmol, 1.1 equiv.) was added slowly via syringe. The heterogeneous mixture was vigorously stirred at room temperature for 40 minutes. During this time a white solid precipitated out, and the mixture turned from white to pale orange to pale pink. The suspension was filtered to give **Stock solution A**.

Reaction vial: A 40-mL vial was charged with a magnetic stir bar, $Ir(dF(CF_3)ppy)_2(dtbbpy)PF_6$ (33.7 mg, 30 μmol, 1 mol%), $Ni(dtbbpy)Br_2$ (146.1 mg, 0.3 mmol, 10 mol%), phthalimide (441 mg, 3.0 mmol, 1.0 equiv.), quinuclidine (499.5 mg, 4.5 mmol, 1.5 equiv.), DMSO (15 mL). The mixture was sonicated until all solids dissolved. The **reaction vial** was opened to air to introduce **Stock Solution A** and (bromoethynyl)triisopropylsilane (1.85 mL, 1.96 g, 7.5 mmol, 2.5 equiv.) with a syringe. The final reaction mixture was then sparged with N_2 for ~1.5 minutes before it was irradiated with 450 nm LED modules at 100% light intensity with maximum fan speed and 600 rpm stir rate in a PennOC or PennPhD Integrated Photoreactor for 2.0 hours.

After completion, the reaction mixture was diluted with 100 mL of water and extracted with ethyl acetate (3 × 50 mL). The combined organic layers were dried over sodium sulfate and concentrated. The crude product was passed through a short silica pad using DCM to remove very polar impurities. The eluent was collected and concentrated for further use.

To a 40 mL reaction vial, the crude product and 1.0 M solution of TBAF in THF (3 mL, 3.0 mmol) were added and the mixture was diluted with THF (20.0 mL). The reaction mixture was stirred at room temperature for 4 h. The reaction mixture then was concentrated under vacuum and subjected to column chromatography on silica gel (eluent: hexanes/EtOAc = 10:1) to afford the desired product **60** (547 mg, 68% overall yield for two steps).

7) Reaction time course studies

The time-study of difference in reaction rates between alkyl and benzylic alcohols are conducted below. We selected the structurally related substrates 2-(pyridin-2-yl)ethan-1-ol and pyridin-3-ylmethanol as model compounds. To compare their relative reactive rate, the reactions were conducted on a 0.05 mmol scale, and the assay yields were determined by HPLC at short reaction times (10–50 s). We fitted the initial assay yield data to a pseudo-first-order kinetic model, which suggests that the initial rates of product formation from the aliphatic and benzylic alcohols are comparable under the standard reaction conditions. This observation is consistent with our proposal that the single-electron transfer from the NHC–alcohol adduct to the excited-state iridium photocatalyst is the rate-determining step, and that the structure of the alcohol has only a minor influence on this process.

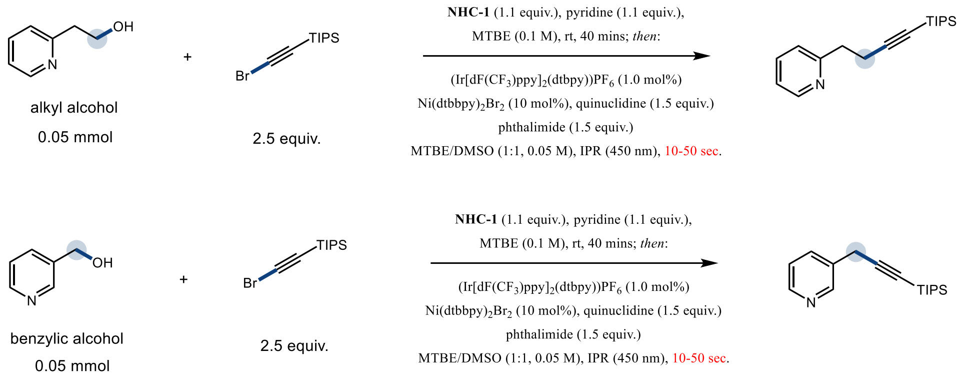

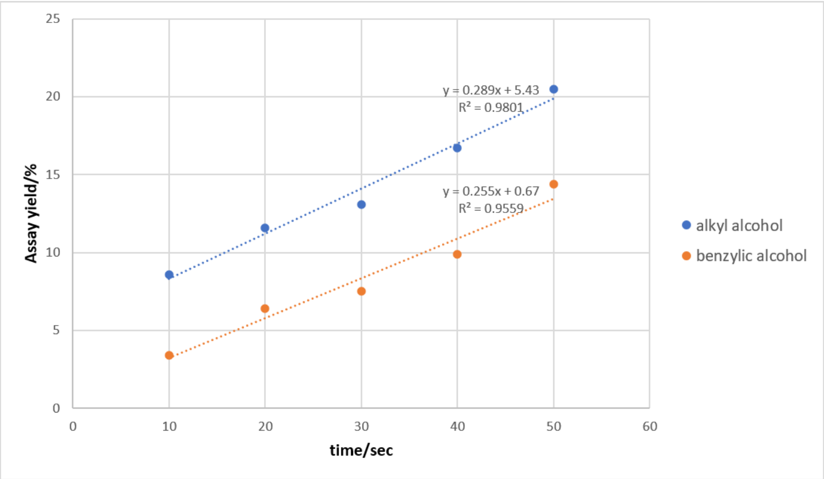

8) Proposed mechanism

A plausible mechanism for the deoxygenative alkynylation is outlined below. The alcohol substrate, **1**, first forms an NHC–alcohol adduct (**73**). Upon blue-light irradiation, the photocatalyst Ir[dF(CF_3)ppy]$_2$(dtbpy)PF_6 (**74**) is excited to its photoactive Ir^{3+*} state (**75**), which undergoes selective reductive quenching by **73** to generate the NHC–alcohol radical cation (**77**) along with the reduced photocatalyst (**76**). Subsequent fragmentation of **77** via rapid β-scission generates the deoxygenated alkyl radical (**78**). Concurrently, in the nickel catalytic cycle, a Ni(0) species (**79**), generated from the Ni(II) precatalyst, undergoes oxidative addition (OA) with the alkynyl bromide (**2**) to form an alkynyl–Ni(II) intermediate (**80**). The latter intercepts the alkyl radical (**78**) to produce a high-valent Ni(III) species (**81**), which then undergoes reductive elimination (RE) to forge the desired C(sp^3)–C(sp) bond, affording the alkynylated product (**3**). This step also generates a Ni(I) species (**82**), which is subsequently reduced to regenerate the active Ni(0) catalyst, thereby closing both the nickel and photoredox catalytic cycles.

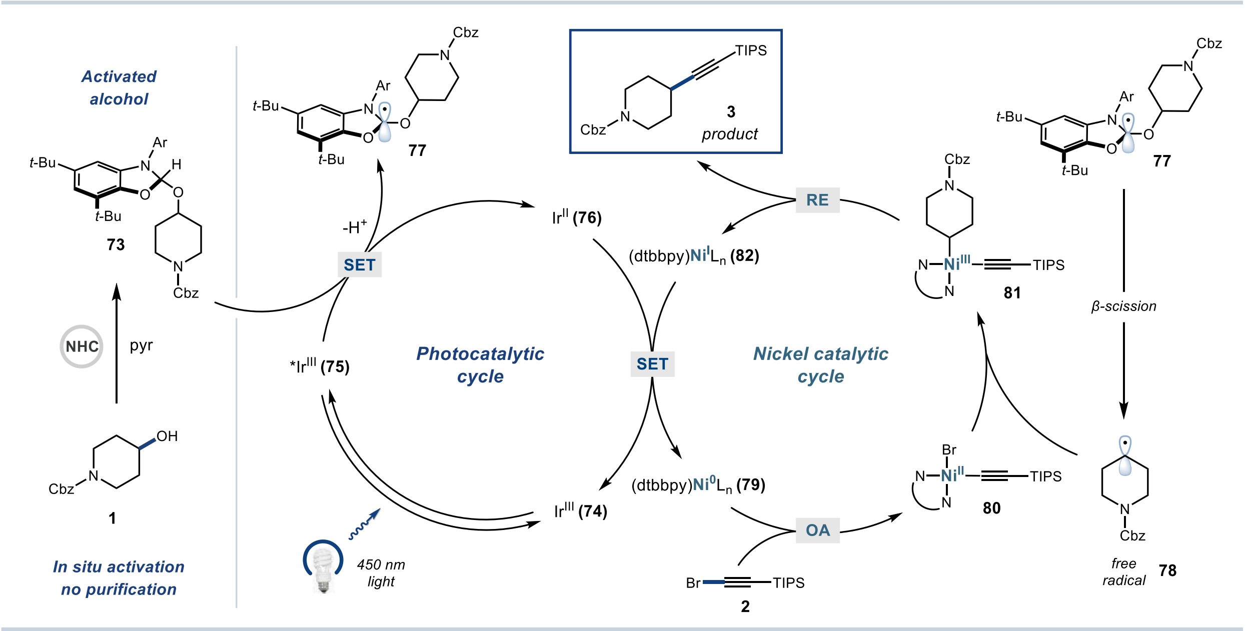

9) Experimental and characterization data

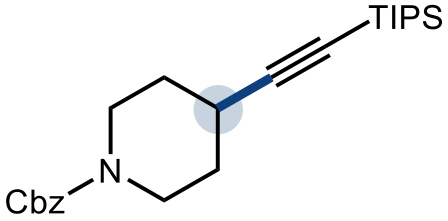

benzyl 4-((triisopropylsilyl)ethynyl)piperidine-1-carboxylate (3): The title compound was prepared according to **General Procedure A** with benzyl 4-hydroxypiperidine-1-carboxylate (alcohol) and (bromoethynyl)triisopropylsilane (alkynyl bromide). Purified by automated flash chromatography (25 g high performance silica column, 0–80% ethyl acetate/hexanes gradient) followed by preparative HPLC (XBridge BEH C18 OBD column, 30–80% MeCN/H_2O with 0.1% NH_4OH) to provide the title compound as a yellow oil. (187.3 mg, 94% yield).

^{1}H NMR (500 MHz, $CDCl_3$) δ 7.48 – 7.27 (m, 5H), 5.13 (s, 2H), 3.65 (ddd, J = 12.3, 8.2, 3.5 Hz, 2H), 3.47 (ddd, J = 13.5, 7.0, 3.7 Hz, 2H), 2.72 (tt, J = 7.1, 4.0 Hz, 1H), 1.77 (s, 2H), 1.64 (s, 2H), 1.06 (d, J = 4.8 Hz, 21H).

^{13}C NMR (126 MHz, $CDCl_3$) δ 155.37, 137.02, 128.63, 128.11, 128.01, 110.51, 82.23, 67.20, 42.07, 27.65, 18.78, 11.36.

HRMS (ESI-TOF) *m/z* calcd. For $C_{24}H_{37}NO_2SiNa^+$ ($[M+Na]^+$) 422.2486, found 422.2486.

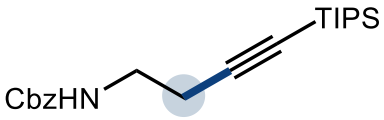

benzyl 3-((triisopropylsilyl)ethynyl)piperidine-1-carboxylate (4): The title compound was prepared according to **General Procedure A** with benzyl (2-hydroxyethyl)carbamate (alcohol) and (bromoethynyl)triisopropylsilane (alkynyl bromide). Purified by automated flash chromatography (25 g high performance silica column, 0–80% ethyl acetate/hexanes gradient) followed by preparative HPLC (XBridge BEH C18 OBD column, 30–80% MeCN/H_2O with 0.1% NH_4OH) to provide the title compound as a yellow oil. (140.9 mg, 78% yield).

^{1}H NMR (500 MHz, $CDCl_3$) δ 7.47 – 7.27 (m, 5H), 5.11 (s, 2H), 5.03 (s, 1H), 3.36 (q, *J* = 6.4 Hz, 2H), 2.48 (t, *J* = 6.5 Hz, 2H), 1.25 – 0.88 (m, 21H).

^{13}C NMR (126 MHz, $CDCl_3$) δ 156.37, 136.68, 128.65, 128.22, 128.13, 105.44, 82.73, 66.82, 40.20, 21.74, 18.74, 11.33.

HRMS (ESI-TOF) *m*/*z* calcd. For $C_{21}H_{34}NO_2Si^+$ ($[M+H]^+$) 360.2354, found 360.2357.

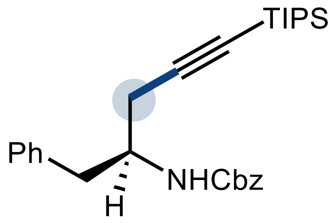

benzyl (S)-(1-phenyl-5-(triisopropylsilyl)pent-4-yn-2-yl)carbamate (5): The title compound was prepared according to **General Procedure A** with benzyl (S)-(1-hydroxy-3-phenylpropan-2-yl)carbamate (alcohol) and (bromoethynyl)triisopropylsilane (alkynyl bromide). Purified by automated flash chromatography (25 g high performance silica column, 0–80% ethyl acetate/hexanes gradient) followed by preparative HPLC (XBridge BEH C18 OBD column, 30–80% MeCN/H_2O with 0.1% NH_4OH) to provide the title compound as a yellow oil. (180.7 mg, 80% yield).

^{1}H NMR (500 MHz, $CDCl_3$) δ 7.44 – 7.26 (m, 7H), 7.26 – 7.17 (m, 3H), 5.10 (s, 2H), 4.96 (d, *J* = 9.0 Hz, 1H), 4.17 – 3.91 (m, 1H), 3.09 – 2.82 (m, 2H), 2.60 – 2.28 (m, 2H), 1.21 – 0.91 (m, 21H).

^{13}C NMR (126 MHz, $CDCl_3$) δ 155.68, 137.63, 136.70, 129.51, 128.71, 128.63, 128.19, 128.06, 126.80, 104.21, 84.26, 66.72, 50.83, 39.55, 24.56, 18.81, 11.41.

HRMS (ESI-TOF) *m*/*z* calcd. For $C_{28}H_{39}NO_2SiNa^+$ ($[M+Na]^+$) 472.2642, found 472.2642.

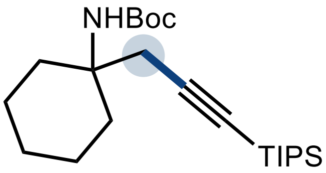

tert-butyl (1-(3-(triisopropylsilyl)prop-2-yn-1-yl)cyclohexyl)carbamate (6): The title compound was prepared according to **General Procedure A** with tert-Butyl (1-(hydroxymethyl)cyclohexyl)carbamate (alcohol) and (bromoethynyl)triisopropylsilane (alkynyl bromide). Purified by automated flash chromatography (25 g high performance silica column, 0–80% ethyl acetate/hexanes gradient) followed by preparative HPLC (XBridge BEH C18 OBD column, 30–80% MeCN/H_2O with 0.1% NH_4OH) to provide the title compound as a yellow oil. (138.1mg, 70% yield).

^{1}H NMR (500 MHz, $CDCl_3$) δ 4.40 (s, 1H), 2.68 (s, 2H), 2.15 – 1.91 (m, 2H), 1.66 – 1.44 (m, 7H), 1.42 (s, 9H), 1.30 – 1.21 (m, 1H), 1.14 – 0.90 (m, 21H).

^{13}C NMR (126 MHz, $CDCl_3$) δ 154.64, 105.54, 82.47, 78.91, 65.99, 54.20, 34.48, 30.26, 28.56, 25.75, 21.85, 18.82, 11.45.

HRMS (ESI-TOF) *m/z* calcd. For $C_{23}H_{43}NO_2Si$-$C_5H_8O_2$+H^+ ([M- $C_5H_8O_2$+H]$^+$) 294.2512, found 294.2607.

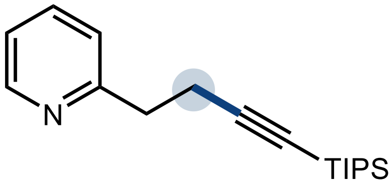

2-(4-(triisopropylsilyl)but-3-yn-1-yl)pyridine (7): The title compound was prepared according to **General Procedure A** with 2-(pyridin-2-yl)ethan-1-ol (alcohol) and (bromoethynyl)triisopropylsilane (alkynyl bromide). Purified by automated flash chromatography (25 g high performance silica column, 0–80% ethyl acetate/hexanes gradient) followed by preparative HPLC (XBridge BEH C18 OBD column, 30–80% MeCN/H_2O with 0.1% NH_4OH) to provide the title compound as a yellow oil. (126.6 mg, 88% yield).

^{1}H NMR (500 MHz, $CDCl_3$) δ 8.56 – 8.47 (m, 1H), 7.57 (td, *J* = 7.6, 1.8 Hz, 1H), 7.24 – 7.19 (m, 1H), 7.13 – 7.08 (m, 1H), 3.00 (t, *J* = 7.3 Hz, 2H), 2.70 (t, *J* = 7.3 Hz, 2H), 1.09 – 0.90 (m, 21H).

^{13}C NMR (126 MHz, $CDCl_3$) δ 160.19, 149.43, 136.29, 123.36, 121.49, 108.05, 81.18, 37.61, 20.23, 18.69, 11.36.

HRMS (ESI-TOF) *m/z* calcd. For $C_{18}H_{30}NSi^+$ ($[M+H]^+$) 288.2142, found 288.2143.

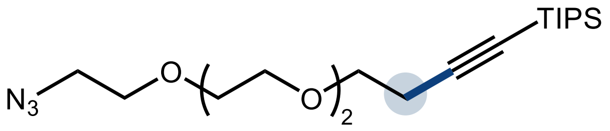

1-azido-17,17-diisopropyl-18-methyl-3,6,9,12-tetraoxa-17-silanonadec-15-yne (8): The title compound was prepared according to **General Procedure A** with 11-azido-3,6,9-trioxaundecanol (alcohol) and (bromoethynyl)triisopropylsilane (alkynyl bromide). Purified by automated flash chromatography (25 g high performance silica column, 0–80% ethyl acetate/hexanes gradient) followed by preparative HPLC (XBridge BEH C18 OBD column, 30–80% MeCN/H_2O with 0.1% NH_4OH) to provide the title compound as a yellow oil. (119.7 mg, 62% yield).

^{1}H NMR (500 MHz, $CDCl_3$) δ 3.69 – 3.59 (m, 12H), 3.38 (t, *J* = 5.1 Hz, 2H), 2.53 (t, *J* = 7.3 Hz, 2H), 1.11 – 1.00 (m, 21H).

^{13}C NMR (126 MHz, $CDCl_3$) δ 105.42, 81.66, 70.86, 70.83, 70.81, 70.44, 70.19, 70.02, 50.83, 21.41, 18.72, 11.36.

HRMS (ESI-TOF) *m/z* calcd. For $C_{19}H_{37}N_3O_3SiNa^+$ ($[M+Na]^+$) 406.2496, found 406.2492.

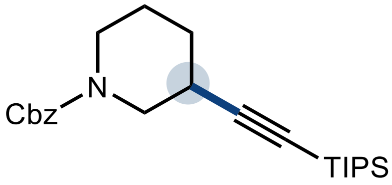

benzyl 3-((triisopropylsilyl)ethynyl)piperidine-1-carboxylate (9): The title compound was prepared according to **General Procedure A** with benzyl 3-hydroxypiperidine-1-carboxylate (alcohol) and (bromoethynyl)triisopropylsilane (alkynyl bromide). Purified by automated flash chromatography (25 g high performance silica column, 0–80% ethyl acetate/hexanes gradient) followed by preparative HPLC (XBridge BEH C18 OBD column, 30–80% MeCN/H_2O with 0.1% NH_4OH) to provide the title compound as a yellow oil. (142.4 mg, 71% yield).

^{1}H NMR (500 MHz, $CDCl_3$) δ 7.46 – 7.28 (m, 5H), 5.12 (s, 2H), 4.10 – 3.63 (m, 2H), 3.08 (s, 2H), 2.58 – 2.45 (m, 1H), 1.97 (s, 1H), 1.75 (s, 1H), 1.59 (s, 1H), 1.51 – 1.38 (m, 1H), 1.09 – 0.98 (m, 21H).

^{13}C NMR (126 MHz, $CDCl_3$) δ 155.24, 136.99, 128.58, 128.05, 127.98, 109.22, 67.20, 49.06, 44.35, 31.23, 29.54, 23.64, 18.71, 11.29.

HRMS (ESI-TOF) *m*/*z* calcd. For $C_{24}H_{37}NO_2SiNa^+$ ($[M+Na]^+$) 422.2486, found 422.2485.

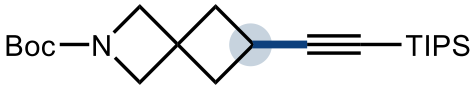

tert-butyl 6-((triisopropylsilyl)ethynyl)-2-azaspiro[3.3]heptane-2-carboxylate (10): The title compound was prepared according to **General Procedure A** with tert-butyl 6-hydroxy-2-azaspiro[3.3]heptane-2-carboxylate (alcohol) and (bromoethynyl)triisopropylsilane (alkynyl bromide). Purified by automated flash chromatography (25 g high performance silica column, 0–80% ethyl acetate/hexanes gradient) followed by preparative HPLC (XBridge BEH C18 OBD column, 30–80% MeCN/H_2O with 0.1% NH_4OH) to provide the title compound as a yellow oil. (113.4 mg, 60% yield).

^{1}H NMR (500 MHz, $CDCl_3$) δ 3.92 (s, 2H), 3.87 (s, 2H), 2.97 – 2.85 (m, 1H), 2.49 (dd, *J* = 12.0, 8.7 Hz, 2H), 2.27 – 2.19 (m, 2H), 1.42 (s, 9H), 1.10 – 0.97 (m, 21H).

^{13}C NMR (126 MHz, $CDCl_3$) δ 156.30, 111.98, 81.35, 79.43, 61.49, 40.72, 35.64, 28.51, 20.88, 18.75, 11.38.

HRMS (ESI-TOF) *m*/*z* calcd. For $C_{22}H_{39}NO_2Si$-C_4H_8+H^+ ([M-C_4H_8+H]$^+$) 322.2197, found 322.2196.

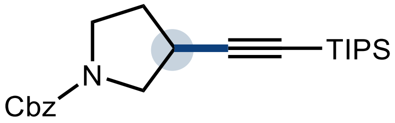

benzyl (S)-3-((triisopropylsilyl)ethynyl)pyrrolidine-1-carboxylate (11): The title compound was prepared according to **General Procedure A** with benzyl 3-hydroxypyrrolidine-1-carboxylate (alcohol) and (bromoethynyl)triisopropylsilane (alkynyl bromide). Purified by automated flash chromatography (25 g high performance silica column, 0–80% ethyl acetate/hexanes gradient) followed by preparative HPLC (XBridge BEH C18 OBD column, 30–80% MeCN/H_2O with 0.1% NH_4OH) to provide the title compound as a yellow oil. (158.3 mg, 82% yield).

^{1}H NMR (500 MHz, $CDCl_3$) δ 7.49 – 7.26 (m, 5H), 5.14 (d, J = 2.1 Hz, 2H), 3.69 (td, J = 10.9, 7.1 Hz, 1H), 3.65 – 3.54 (m, 1H), 3.49 – 3.30 (m, 2H), 3.01 (h, J = 7.2 Hz, 1H), 2.21 – 2.11 (m, 1H), 1.97 (tq, J = 12.2, 7.6 Hz, 1H), 1.20 – 0.87 (m, 21H).

^{13}C NMR (126 MHz, $CDCl_3$) δ 154.80, 154.78, 137.06, 137.04, 128.58, 128.06, 128.03, 127.95, 108.36, 108.19, 82.34, 66.92, 66.87, 52.43, 51.92, 45.57, 45.15, 33.19, 32.42, 31.15, 30.35, 18.69, 11.26. (Mixture of rotamers.)

HRMS (ESI-TOF) *m/z* calcd. For $C_{23}H_{35}NO_2SiNa^+$ ($[M+Na]^+$) 408.2329, found 408.2330.

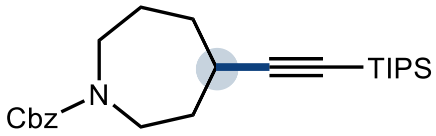

benzyl 4-((triisopropylsilyl)ethynyl)azepane-1-carboxylate (12): The title compound was prepared according to **General Procedure A** with benzyl 4-hydroxyazepane-1-carboxylate (alcohol) and (bromoethynyl)triisopropylsilane (alkynyl bromide). Purified by automated flash chromatography (25 g high performance silica column, 0–80% ethyl acetate/hexanes gradient) followed by preparative HPLC (XBridge BEH C18 OBD column, 30–80% $MeCN/H_2O$ with 0.1% NH_4OH) to provide the title compound as a yellow oil. (145.0 mg, 70% yield).

^{1}H NMR (500 MHz, $CDCl_3$) δ 7.47 – 7.27 (m, 5H), 5.26 – 5.06 (m, 2H), 3.73 – 3.55 (m, 2H), 3.49 – 3.33 (m, 2H), 2.79 (tt, *J* = 6.6, 3.5 Hz, 1H), 2.02 – 1.64 (m, 6H), 1.19 – 0.93 (m, 21H).

^{13}C NMR (126 MHz, $CDCl_3$) δ 156.22, 137.21, 128.58, 127.99, 127.87, 127.86, 111.23, 81.87, 67.04, 67.01, 46.41, 46.28, 44.50, 43.87, 35.18, 34.92, 32.25, 32.13, 31.15, 30.95, 25.00, 24.73, 18.77, 11.39.

HRMS (ESI-TOF) *m/z* calcd. For $C_{25}H_{39}NO_2SiNa^+$ ($[M+Na]^+$) 436.2642, found 436.2640.

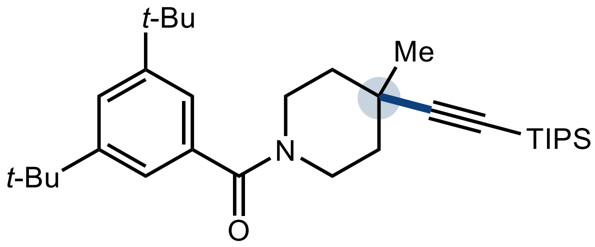

(3,5-di-tert-butylphenyl)(4-methyl-4-((triisopropylsilyl)ethynyl)piperidin-1-yl)methanone (13): The title compound was prepared according to **General Procedure E** with (3,5-di-tert-butylphenyl)(4-hydroxy-4-methylpiperidin-1-yl)methanone (alcohol) and (bromoethynyl)triisopropylsilane (alkynyl bromide). Purified by automated flash chromatography (25 g high performance silica column, 0–80% ethyl acetate/hexanes gradient) followed by preparative HPLC (XBridge BEH C18 OBD column, 30–100% MeCN/H_2O with 0.1% NH_4OH) to provide the title compound as a yellow oil. (73.5 mg, 30% yield).

^{1}H NMR (500 MHz, $CDCl_3$) δ 7.44 (t, J = 1.8 Hz, 1H), 7.21 (d, J = 1.8 Hz, 2H), 4.66 (d, J = 13.1 Hz, 1H), 3.65 (d, J = 13.5 Hz, 1H), 3.37 (t, J = 12.6 Hz, 1H), 3.16 (t, J = 12.1 Hz, 1H), 1.81 (d, J = 13.4 Hz, 1H), 1.63 (d, J = 9.3 Hz, 2H), 1.49 (d, J = 15.4 Hz, 1H), 1.32 (s, 18H), 1.30 (s, 3H), 1.12 – 0.99 (m, 21H).

^{13}C NMR (126 MHz, $CDCl_3$) δ 171.48, 151.02, 135.59, 123.64, 121.14, 113.08, 82.77, 45.59, 39.88, 39.35, 38.47, 35.05, 32.91, 31.52, 29.97, 18.81, 11.35.

HRMS (ESI-TOF) m/z calcd. For $C_{32}H_{53}NOSiNa^+$ ($[M+Na]^+$) 518.3789, found 518.3794.

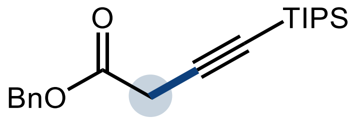

benzyl 4-(triisopropylsilyl)but-3-ynoate (14): The title compound was prepared according to **General Procedure A** with benzyl 2-hydroxyacetate (alcohol) and (bromoethynyl)triisopropylsilane (alkynyl bromide). Purified by automated flash chromatography (25 g high performance silica column, 0–80% ethyl acetate/hexanes gradient) followed by preparative HPLC (XBridge BEH C18 OBD column, 30–100% MeCN/H_2O with 0.1% NH_4OH) to provide the title compound as a yellow oil. (111.8 mg, 68% yield).

^{1}H NMR (500 MHz, $CDCl_3$) δ 7.43 – 7.29 (m, 5H), 5.18 (s, 2H), 3.40 (s, 2H), 1.12 – 1.01 (m, 21H).

^{13}C NMR (126 MHz, $CDCl_3$) δ 168.04, 135.58, 128.68, 128.47, 128.38, 98.93, 84.83, 67.28, 27.42, 18.68, 11.33.

HRMS (ESI-TOF) *m/z* calcd. For $C_{20}H_{31}O_2Si^+$ ($[M+H]^+$) 331.2088, found 331.2093.

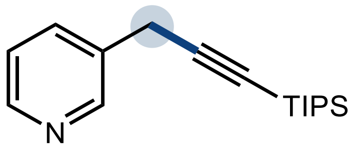

3-(3-(triisopropylsilyl)prop-2-yn-1-yl)pyridine (15): The title compound was prepared according to **General Procedure A** with pyridin-3-ylmethanol (alcohol) and (bromoethynyl)triisopropylsilane (alkynyl bromide). Purified by automated flash chromatography (25 g high performance silica column, 0–80% ethyl acetate/hexanes gradient) followed by preparative HPLC (XBridge BEH C18 OBD column, 30–80% $MeCN/H_2O$ with 0.1% NH_4OH) to provide the title compound as a yellow oil. (81.8 mg, 60% yield).

^{1}H NMR (500 MHz, $CDCl_3$) δ 8.60 (s, 1H), 8.48 (d, J = 3.1 Hz, 1H), 7.71 (dt, J = 6.6, 1.6 Hz, 1H), 7.26 – 7.22 (m, 1H), 3.69 (s, 2H), 1.08 (t, J = 2.7 Hz, 21H).

^{13}C NMR (126 MHz, $CDCl_3$) δ 149.48, 148.14, 135.48, 132.53, 123.47, 104.17, 84.13, 23.90, 18.75, 11.39.

HRMS (ESI-TOF) *m/z* calcd. For $C_{17}H_{28}NSi^+$ ($[M+H]^+$) 274.1986, found 274.1986.

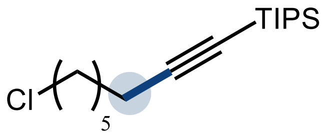

(8-chlorooct-1-yn-1-yl)triisopropylsilane (16): The title compound was prepared according to **General Procedure A** with 6-chlorohexan-1-ol (alcohol) and (bromoethynyl)triisopropylsilane (alkynyl bromide). Purified by automated flash chromatography (25 g high performance silica column, 0–80% ethyl acetate/hexanes gradient) followed by preparative HPLC (XBridge BEH C18 OBD column, 30–100% MeCN/H_2O with 0.1% NH_4OH) to provide the title compound as a yellow oil. (114.1 mg, 76% yield).

^{1}H NMR (500 MHz, $CDCl_3$) δ 3.56 (t, J = 6.7 Hz, 2H), 2.29 (t, J = 6.8 Hz, 2H), 1.86 – 1.75 (m, 2H), 1.60 – 1.54 (m, 2H), 1.49 (pd, J = 6.1, 4.0 Hz, 4H), 1.17 – 0.98 (m, 21H).

^{13}C NMR (126 MHz, $CDCl_3$) δ 109.04, 80.44, 45.14, 32.69, 28.75, 28.04, 26.49, 19.87, 18.78, 11.45.

HRMS (ESI-TOF) m/z calcd. For $C_{17}H_{33}ClSiNa^+$ ($[M+Na]^+$) 323.1932, found 323.2117.

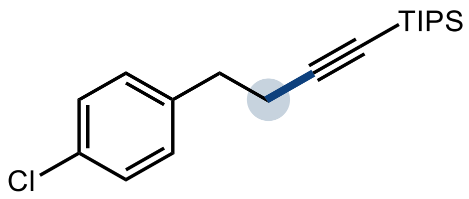

(4-(4-chlorophenyl)but-1-yn-1-yl)triisopropylsilane (17): The title compound was prepared according to **General Procedure A** with 2-(4-chlorophenyl)ethan-1-ol (alcohol) and (bromoethynyl)triisopropylsilane (alkynyl bromide). Purified by automated flash chromatography (25 g high performance silica column, 0–80% ethyl acetate/hexanes gradient) followed by preparative HPLC (XBridge BEH C18 OBD column, 30–100% $MeCN/H_2O$ with 0.1% NH_4OH) to provide the title compound as a yellow oil. (145.9 mg, 91% yield).

^{1}H NMR (500 MHz, $CDCl_3$) δ 7.29 – 7.25 (m, 2H), 7.22 – 7.18 (m, 2H), 2.83 (t, *J* = 7.2 Hz, 2H), 2.56 (t, *J* = 7.2 Hz, 2H), 1.17 – 0.95 (m, 21H).

^{13}C NMR (126 MHz, $CDCl_3$) δ 139.19, 132.13, 130.06, 128.49, 107.72, 81.62, 34.64, 22.02, 18.71, 11.40.

HRMS (EI) *m*/*z* calcd. For $C_{17}H_{29}ClSi^+$ ($[M]^+$) 320.1722, found 320.1729.

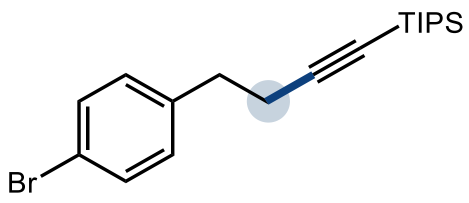

(4-(4-bromophenyl)but-1-yn-1-yl)triisopropylsilane (18): The title compound was prepared according to **General Procedure A** with 2-(4-bromophenyl)ethan-1-ol (alcohol) and (bromoethynyl)triisopropylsilane (alkynyl bromide). Purified by automated flash chromatography (25 g high performance silica column, 0–80% ethyl acetate/hexanes gradient) followed by preparative HPLC (XBridge BEH C18 OBD column, 30–100% MeCN/H_2O with 0.1% NH_4OH) to provide the title compound as a yellow oil. (140.8 mg, 77% yield).

^{1}H NMR (500 MHz, $CDCl_3$) δ 7.39 (d, J = 8.3 Hz, 2H), 7.12 (d, J = 8.3 Hz, 2H), 2.79 (t, J = 7.2 Hz, 2H), 2.53 (t, J = 7.2 Hz, 2H), 1.10 – 0.82 (m, 21H).

^{13}C NMR (126 MHz, $CDCl_3$) δ 139.72, 131.47, 130.48, 120.17, 107.68, 81.66, 34.70, 21.95, 18.72, 11.40.

HRMS (EI) *m/z* calcd. For $C_{17}H_{29}BrSi^+$ ($[M]^+$) 364.1216, found 364.1211.

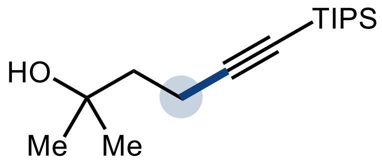

2-methyl-6-(triisopropylsilyl)hex-5-yn-2-ol (19): The title compound was prepared according to **General Procedure A** with 3-Methyl-1,3-butanediol (alcohol) and (bromoethynyl)triisopropylsilane (alkynyl bromide). Purified by automated flash chromatography (25 g high performance silica column, 0–80% ethyl acetate/hexanes gradient) followed by prep-TLC plate (Analtech Silica gel GF UV254 1000 micron) using hexanes/EtOAc = 5:1 as the eluent to provide the title compound as a yellow oil. (96.3 mg, 72% yield).

^{1}H NMR (500 MHz, CDCl$_3$) δ 2.39 (t, J = 7.4 Hz, 2H), 1.86 (s, 1H), 1.75 (t, J = 7.5 Hz, 2H), 1.25 (s, 6H), 1.14 – 0.91 (m, 21H).

^{13}C NMR (126 MHz, CDCl$_3$) δ 109.23, 81.44, 70.99, 41.97, 29.29, 18.75, 15.30, 11.42.

HRMS (ESI-TOF) m/z calcd. For $C_{16}H_{33}OSi^+$ ($[M+H]^+$) 269.2296, found 269.2299.

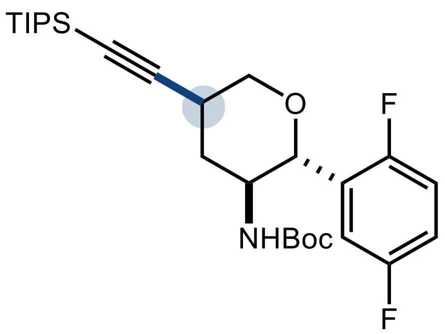

tert-butyl ((2R,3S,5S)-2-(2,5-difluorophenyl)-5-((triisopropylsilyl)ethynyl)tetrahydro-2H-pyran-3-yl)carbamate (20): The title compound was prepared according to **General Procedure A** with tert-butyl ((2R,3S)-2-(2,5-difluorophenyl) (alcohol) and (bromoethynyl)triisopropylsilane (alkynyl bromide). Purified by automated flash chromatography (25 g high performance silica column, 0–80% ethyl acetate/hexanes gradient) followed by preparative HPLC (XBridge BEH C18 OBD column, 30–80% MeCN/H_2O with 0.1% NH_4OH) to provide the title compound as a yellow oil. (153.3 mg, 62% yield, d.r. > 20:1). (relative configuration not determined)

^{1}H NMR (500 MHz, CDCl$_3$) δ 7.18 (d, J = 8.3 Hz, 1H), 7.07 – 6.82 (m, 2H), 4.42 (d, J = 9.6 Hz, 1H), 4.34 (d, J = 9.7 Hz, 1H), 4.22 – 4.10 (m, 1H), 3.63 (d, J = 11.2 Hz, 1H), 3.41 (t, J = 11.2 Hz, 1H), 2.87 (tt, J = 11.5, 4.3 Hz, 1H), 2.58 – 2.44 (m, 1H), 1.64 – 1.55 (m, 1H), 1.26 (s, 9H), 1.05 (d, J = 4.9 Hz, 21H).

^{13}C NMR (126 MHz, CDCl$_3$) δ 160.04, 158.11, 157.30, 155.39, 154.66, 128.38, 128.24, 116.21, 116.14, 116.01, 115.94, 115.26, 115.23, 115.06, 115.03, 106.19, 83.00, 79.64, 76.56, 71.79, 51.32, 38.30, 30.21, 28.22, 18.71, 11.24. (J_{CF} not determined)

^{19}F NMR (470 MHz, CDCl$_3$) δ -117.94, -118.79, -123.48, -124.47.

HRMS (ESI-TOF) *m/z* calcd. For $C_{27}H_{41}F_2NO_3Si\text{-}C_5H_8O_2+H^+$ ($[M\text{-}C_5H_8O_2+H]^+$) 394.2372, found 394.2379.

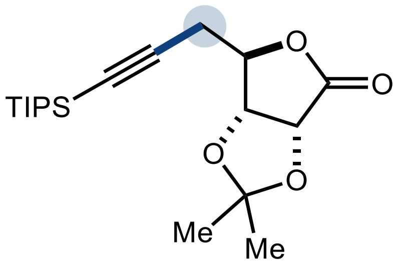

(3aR,6R,6aR)-2,2-dimethyl-6-(3-(triisopropylsilyl)prop-2-yn-1-yl)dihydrofuro[3,4-d][1,3]dioxol-4(3aH)-one (21): The title compound was prepared according to **General Procedure A** with (3aR,6R,6aR)-6-(hydroxymethyl)-2,2-dimethyldihydrofuro[3,4-d][1,3]dioxol-4(3aH)-one (alcohol) and (bromoethynyl)triisopropylsilane (alkynyl bromide). Purified by automated flash chromatography (25 g high performance silica column, 0–80% ethyl acetate/hexanes gradient) followed by preparative HPLC (XBridge BEH C18 OBD column, 30–80% MeCN/H_2O with 0.1% NH_4OH) to provide the title compound as a yellow oil. (138.1 mg, 78% yield).

^{1}H NMR (500 MHz, $CDCl_3$) δ 4.91 (d, J = 5.9 Hz, 1H), 4.75 (d, J = 5.9 Hz, 1H), 4.65 (dd, J = 5.5, 3.4 Hz, 1H), 2.82 – 2.68 (m, 2H), 1.47 (s, 3H), 1.38 (s, 3H), 1.05 (t, J = 2.4 Hz, 21H).

^{13}C NMR (126 MHz, $CDCl_3$) δ 173.49, 113.70, 100.77, 86.37, 80.44, 79.17, 75.31, 26.71, 25.53, 25.01, 18.64, 18.63, 11.29.

HRMS (ESI-TOF) *m/z* calcd. For $C_{19}H_{33}O_4Si^+$ ($[M+H]^+$) 353.2143, found 353.2143.

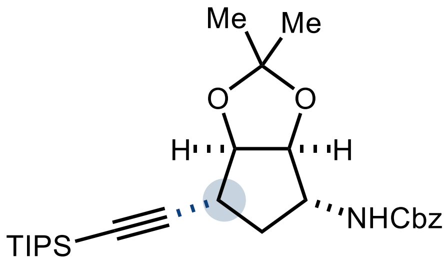

benzyl ((3aS,4R,6R,6aR)-2,2-dimethyl-6-((triisopropylsilyl)ethynyl)tetrahydro-4H-cyclopenta[d][1,3]dioxol-4-yl)carbamate (22): The title compound was prepared according to **General Procedure A** with benzyl (3aS,4R,6S,6aR)-6-hydroxy-2,2-dimethyltetrahydro-3aH-cyclopenta[d][1,3]dioxol-4-ylcarbamate (alcohol) and (bromoethynyl)triisopropylsilane (alkynyl bromide). Purified by automated flash chromatography (25 g high performance silica column, 0–80% ethyl acetate/hexanes gradient) followed by preparative HPLC (XBridge BEH C18 OBD column, 30–80% MeCN/H_2O with 0.1% NH_4OH) to provide the title compound as a yellow oil. (106.6 mg, 45% yield, d.r. > 20:1). (relative configuration is determined by NOESY analysis)

^{1}H NMR (500 MHz, $CDCl_3$) δ 7.44 – 7.26 (m, 5H), 5.76 (d, *J* = 8.7 Hz, 1H), 5.06 (s, 2H), 4.73 (d, *J* = 5.4 Hz, 1H), 4.61 (d, *J* = 5.5 Hz, 1H), 4.19 (dd, *J* = 8.7, 6.3 Hz, 1H), 3.03 (d, *J* = 7.3 Hz, 1H), 2.39 (dt, *J* = 13.8, 6.9 Hz, 1H), 1.81 (d, *J* = 13.6 Hz, 1H), 1.42 (s, 3H), 1.28 (s, 3H), 1.13 – 0.91 (m, 21H).

^{13}C NMR (126 MHz, $CDCl_3$) δ 155.62, 136.49, 128.51, 128.17, 110.63, 110.06, 86.78, 85.75, 85.30, 66.95, 58.39, 37.76, 34.55, 26.40, 24.13, 18.65, 11.16.

HRMS (ESI-TOF) *m*/*z* calcd. For $C_{27}H_{42}NO_4Si^+$ ($[M+H]^+$) 472.2878, found 472.2879.

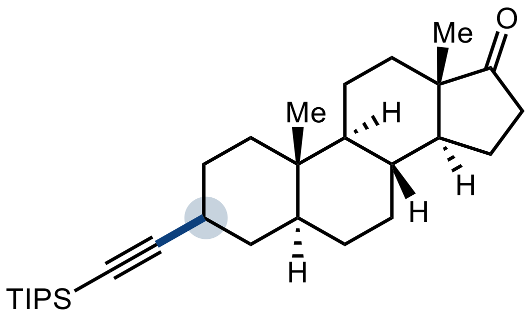

(3S,5S,8R,9S,10S,13S,14S)-10,13-dimethyl-3-((triisopropylsilyl)ethynyl)hexadecahydro-17H-cyclopenta[a]phenanthren-17-one (23): The title compound was prepared according to **General Procedure A** with Epiandrosterone (alcohol) and (bromoethynyl)triisopropylsilane (alkynyl bromide). Purified by automated flash chromatography (25 g high performance silica column, 0–80% ethyl acetate/hexanes gradient) followed by preparative HPLC (XBridge BEH C18 OBD column, 30–100% MeCN/H_2O with 0.1% NH_4OH) to provide the title compound as a yellow oil. (122.1 mg, 54% yield, d.r. > 20:1). (relative configuration not determined)

^{1}H NMR (500 MHz, $CDCl_3$) δ 2.42 (dd, J = 19.2, 8.9 Hz, 1H), 2.27 (tt, J = 12.2, 4.1 Hz, 1H), 2.05 (dt, J = 18.9, 9.1 Hz, 1H), 1.96 – 1.89 (m, 1H), 1.84 – 1.74 (m, 3H), 1.70 – 1.61 (m, 2H), 1.59 – 1.44 (m, 4H), 1.44 – 1.14 (m, 7H), 1.11 – 0.97 (m, 21H), 0.97 – 0.88 (m, 2H), 0.85 (s, 3H), 0.82 (s, 3H), 0.73 – 0.66 (m, 1H).

^{13}C NMR (126 MHz, $CDCl_3$) δ 125.90, 113.80, 78.90, 54.64, 51.63, 47.92, 46.48, 38.17, 35.96, 35.90, 35.67, 35.17, 31.69, 31.17, 31.00, 29.21, 28.39, 21.88, 20.32, 18.77, 13.95, 12.39, 11.38.

HRMS (ESI-TOF) *m/z* calcd. For $C_{30}H_{51}OSi^+$ ($[M+H]^+$) 455.3704, found 455.3701.

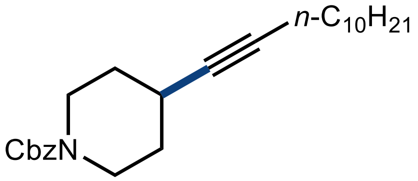

benzyl 4-(dodec-1-yn-1-yl)piperidine-1-carboxylate (24): The title compound was prepared according to **General Procedure B** with 4-hydroxypiperidine-1-carboxylate (alcohol) and 1-bromododec-1-yne (alkynyl bromide). Purified by automated flash chromatography (25 g high performance silica column, 0–80% ethyl acetate/hexanes gradient) followed by preparative HPLC (XBridge BEH C18 OBD column, 30–80% MeCN/H_2O with 0.1% NH_4OH) to provide the title compound as a yellow oil. (124.4 mg, 65% yield).

^{1}H NMR (500 MHz, CDCl$_3$) δ 7.48 – 7.27 (m, 5H), 5.13 (s, 2H), 3.73 (ddd, *J* = 13.3, 7.0, 3.6 Hz, 2H), 3.31 (ddd, *J* = 13.5, 8.1, 3.5 Hz, 2H), 2.58 (dtq, *J* = 8.0, 4.0, 2.0 Hz, 1H), 2.15 (td, *J* = 7.1, 2.2 Hz, 2H), 1.74 (d, *J* = 10.9 Hz, 2H), 1.63 – 1.51 (m, 2H), 1.51 – 1.44 (m, 2H), 1.40 – 1.22 (m, 14H), 0.88 (t, *J* = 6.9 Hz, 3H).

^{13}C NMR (126 MHz, CDCl$_3$) δ 155.37, 137.05, 128.59, 128.05, 127.95, 82.22, 81.95, 67.12, 42.43, 32.03, 31.83, 29.70, 29.67, 29.44, 29.25, 29.19, 28.96, 26.99, 22.81, 18.80, 14.23.

HRMS (ESI-TOF) *m*/*z* calcd. For $C_{25}H_{37}NO_2Na^+$ ($[M+Na]^+$) 406.2717, found 406.2717.

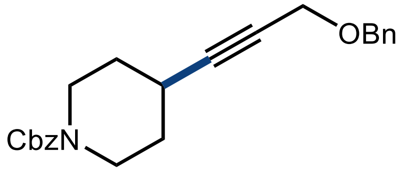

benzyl 4-(3-(benzyloxy)prop-1-yn-1-yl)piperidine-1-carboxylate (25): The title compound was prepared according to **General Procedure B** with 4-hydroxypiperidine-1-carboxylate (alcohol) and (((3-bromoprop-2-yn-1-yl)oxy)methyl)benzene (alkynyl bromide). Purified by automated flash chromatography (25 g high performance silica column, 0–80% ethyl acetate/hexanes gradient) followed by preparative HPLC (XBridge BEH C18 OBD column, 30–60% MeCN/H_2O with 0.1% NH_4OH) to provide the title compound as a yellow oil. (120.9 mg, 66% yield).

^{1}H NMR (500 MHz, $CDCl_3$) δ 7.53 – 7.27 (m, 10H), 5.14 (s, 2H), 4.59 (s, 2H), 4.18 (d, *J* = 2.0 Hz, 2H), 3.77 (ddd, *J* = 13.6, 6.6, 3.5 Hz, 2H), 3.30 (ddd, *J* = 13.5, 8.4, 3.5 Hz, 2H), 2.67 (dtt, *J* = 8.1, 4.1, 2.0 Hz, 1H), 1.82 (d, *J* = 11.6 Hz, 2H), 1.69 – 1.57 (m, 2H).

^{13}C NMR (126 MHz, $CDCl_3$) δ 155.33, 137.65, 136.96, 128.61, 128.55, 128.18, 128.10, 127.98, 127.96, 88.73, 77.61, 71.61, 67.19, 57.73, 42.48, 31.35, 27.08.

HRMS (ESI-TOF) *m*/*z* calcd. For $C_{23}H_{26}NO_3^+$ ($[M+H]^+$) 364.1907, found 364.1907.

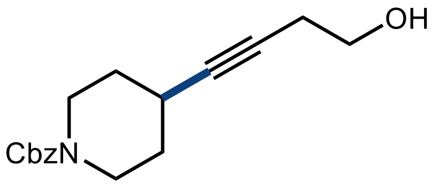

benzyl 4-(4-hydroxybut-1-yn-1-yl)piperidine-1-carboxylate (26): The title compound was prepared according to **General Procedure B** with 4-hydroxypiperidine-1-carboxylate (alcohol) and 4-bromobut-3-yn-1-ol (alkynyl bromide). Purified by automated flash chromatography (25 g high performance silica column, 0–80% ethyl acetate/hexanes gradient) followed by preparative HPLC (XBridge BEH C18 OBD column, 30–60% MeCN/H_2O with 0.1% NH_4OH) to provide the title compound as a yellow oil. (97.4 mg, 68% yield).

^{1}H NMR (500 MHz, $CDCl_3$) δ 7.38 – 7.27 (m, 5H), 5.11 (s, 2H), 3.81 – 3.70 (m, 2H), 3.66 (t, *J* = 6.5 Hz, 2H), 3.31 – 3.18 (m, 2H), 2.63 – 2.53 (m, 1H), 2.43 (td, *J* = 6.5, 2.2 Hz, 2H), 2.19 (s, 1H), 1.76 (d, *J* = 13.6 Hz, 2H), 1.55 (d, *J* = 11.7 Hz, 2H).

^{13}C NMR (126 MHz, $CDCl_3$) δ 155.32, 136.86, 128.54, 128.03, 127.90, 84.07, 78.29, 67.13, 61.37, 42.44, 26.97, 23.12.

HRMS (ESI-TOF) *m*/*z* calcd. For $C_{17}H_{22}NO_3^+$ ($[M+H]^+$) 288.1594, found 288.1587.

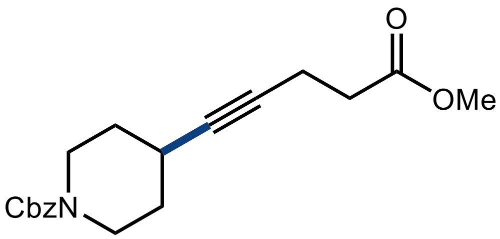

benzyl 4-(5-methoxy-5-oxopent-1-yn-1-yl)piperidine-1-carboxylate (27): The title compound was prepared according to **General Procedure B** with 4-hydroxypiperidine-1-carboxylate (alcohol) and methyl 5-bromopent-4-ynoate (alkynyl bromide, due to the low boiling point, it was used without purification after being prepared in crude form commercially available methyl pent-4-ynoate according to **General Procedure D**, followed by passage through a short silica pad to remove polar byproducts). Purified by automated flash chromatography (25 g high performance silica column, 0–80% ethyl acetate/hexanes gradient) followed by preparative HPLC (XBridge BEH C18 OBD column, 30–60% MeCN/H_2O with 0.1% NH_4OH) to provide the title compound as a yellow oil. (116.3 mg, 71% yield).

^{1}H NMR (500 MHz, $CDCl_3$) δ 7.50 – 7.27 (m, 5H), 5.12 (s, 2H), 3.68 (s, 5H), 3.30 (ddd, J = 13.5, 8.1, 3.5 Hz, 2H), 2.56 (ddh, J = 8.0, 6.4, 2.0 Hz, 1H), 2.53 – 2.42 (m, 4H), 1.73 (t, J = 9.4 Hz, 2H), 1.53 (d, J = 12.2 Hz, 2H).

^{13}C NMR (126 MHz, $CDCl_3$) δ 172.58, 155.35, 137.01, 128.59, 128.06, 127.96, 82.89, 80.04, 67.13, 51.83, 42.35, 33.99, 31.59, 26.87, 14.87.

HRMS (ESI-TOF) *m/z* calcd. For $C_{19}H_{24}NO_4^+$ ($[M+H]^+$) 330.1700, found 330.1702.

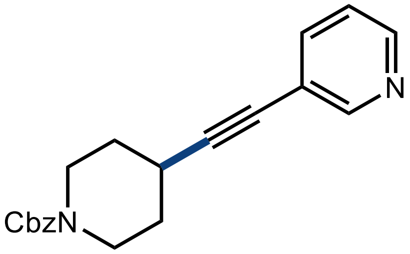

benzyl 4-(pyridin-3-ylethynyl)piperidine-1-carboxylate (28): The title compound was prepared according to **General Procedure B** with 4-hydroxypiperidine-1-carboxylate (alcohol) and 3-(bromoethynyl)pyridine (alkynyl bromide). Purified by automated flash chromatography (25 g high performance silica column, 0–80% ethyl acetate/hexanes gradient) followed by preparative HPLC (XBridge BEH C18 OBD column, 30–60% MeCN/H_2O with 0.1% NH_4OH) to provide the title compound as a yellow oil. (117.8 mg, 74% yield).

^{1}H NMR (500 MHz, $CDCl_3$) δ 8.62 (d, J = 2.1 Hz, 1H), 8.50 (dd, J = 4.9, 1.7 Hz, 1H), 7.67 (dt, J = 7.9, 2.0 Hz, 1H), 7.44 – 7.29 (m, 5H), 7.24 – 7.18 (m, 1H), 5.14 (s, 2H), 3.82 (ddd, J = 13.8, 7.1, 3.7 Hz, 2H), 3.34 (ddd, J = 13.5, 8.4, 3.5 Hz, 2H), 2.84 (tt, J = 8.1, 4.0 Hz, 1H), 1.89 (t, J = 9.5 Hz, 2H), 1.71 (d, J = 11.2 Hz, 2H).

^{13}C NMR (126 MHz, $CDCl_3$) δ 155.36, 152.48, 148.38, 138.61, 136.93, 128.63, 128.13, 128.02, 123.04, 120.71, 95.22, 79.01, 67.25, 42.54, 31.30, 27.69.

HRMS (ESI-TOF) *m/z* calcd. For $C_{22}H_{21}N_2O_2^+$ ([M+H]$^+$) 321.1598, found 321.1600.

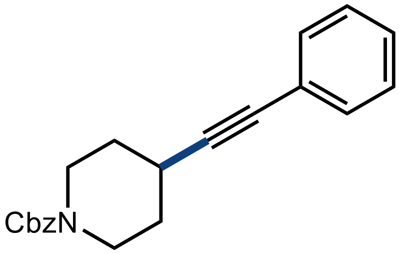

benzyl 4-(phenylethynyl)piperidine-1-carboxylate (29): The title compound was prepared according to **General Procedure B** with 4-hydroxypiperidine-1-carboxylate (alcohol) and (bromoethynyl)benzene (alkynyl bromide). Purified by automated flash chromatography (25 g high performance silica column, 0–80% ethyl acetate/hexanes gradient) followed by preparative HPLC (XBridge BEH C18 OBD column, 30–60% MeCN/H_2O with 0.1% NH_4OH) to provide the title compound as a yellow oil. (106.1 mg, 67% yield).

^{1}H NMR (500 MHz, $CDCl_3$) δ 7.47 – 7.26 (m, 10H), 5.15 (s, 2H), 3.91 – 3.73 (m, 2H), 3.38 (ddd, *J* = 13.5, 8.2, 3.5 Hz, 2H), 2.84 (tt, *J* = 7.9, 4.0 Hz, 1H), 1.88 (t, *J* = 9.9 Hz, 2H), 1.71 (d, *J* = 10.9 Hz, 2H).

^{13}C NMR (126 MHz, $CDCl_3$) δ 155.38, 137.00, 131.71, 128.61, 128.36, 128.09, 127.99, 127.95, 123.59, 91.60, 82.29, 67.19, 42.50, 31.47, 27.56.

HRMS (ESI-TOF) *m/z* calcd. For $C_{22}H_{22}NO_2^+$ ([M+H]$^+$) 320.1645, found 320.1645.

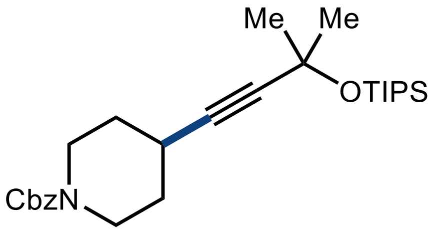

benzyl 4-(3-methyl-3-((triisopropylsilyl)oxy)but-1-yn-1-yl)piperidine-1-carboxylate (30): The title compound was prepared according to **General Procedure B** with 4-hydroxypiperidine-1-carboxylate (alcohol) and ((4-bromo-2-methylbut-3-yn-2-yl)oxy)triisopropylsilane (alkynyl bromide). Purified by automated flash chromatography (25 g high performance silica column, 0–80% ethyl acetate/hexanes gradient) followed by preparative HPLC (XBridge BEH C18 OBD column, 30–80% MeCN/H_2O with 0.1% NH_4OH) to provide the title compound as a yellow oil. (189.9 mg, 83% yield).

^{1}H NMR (500 MHz, $CDCl_3$) δ 7.41 – 7.28 (m, 5H), 5.13 (s, 2H), 3.85 – 3.71 (m, 2H), 3.22 (ddd, J = 13.5, 8.9, 3.4 Hz, 2H), 2.56 (tt, J = 8.2, 3.9 Hz, 1H), 1.84 – 1.71 (m, 2H), 1.54 (d, J = 14.6 Hz, 2H), 1.49 (s, 6H), 1.19 – 1.02 (m, 21H).

^{13}C NMR (126 MHz, $CDCl_3$) δ 155.34, 136.99, 128.61, 128.09, 127.98, 87.41, 83.73, 67.18, 66.30, 42.67, 33.69, 31.32, 27.11, 18.46, 13.14.

HRMS (ESI-TOF) *m/z* calcd. For $C_{27}H_{44}NO_3Si^+$ ($[M+H]^+$) 458.3085, found 458.3082.

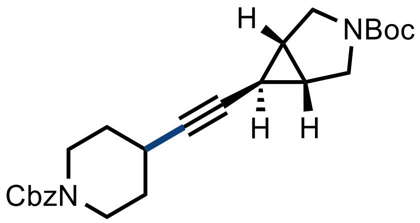

tert-butyl (1R,5S,6s)-6-((1-((benzyloxy)carbonyl)piperidin-4-yl)ethynyl)-3-azabicyclo[3.1.0]hexane-3-carboxylate (31): The title compound was prepared according to **General Procedure B** in 0.2 mmol scale with 4-hydroxypiperidine-1-carboxylate (alcohol) and tert-butyl (1R,5S,6s)-6-(bromoethynyl)-3-azabicyclo[3.1.0]hexane-3-carboxylate (alkynyl bromide). Purified by automated flash chromatography (25 g high performance silica column, 0–80% ethyl acetate/hexanes gradient) followed by preparative HPLC (XBridge BEH C18 OBD column, 30–60% MeCN/H_2O with 0.1% NH_4OH) to provide the title compound as a yellow oil. (53.3 mg, 63% yield).

^{1}H NMR (500 MHz, $CDCl_3$) δ 7.48 – 7.27 (m, 5H), 5.11 (s, 2H), 3.79 – 3.66 (m, 2H), 3.59 (dd, *J* = 42.5, 11.1 Hz, 2H), 3.32 (td, *J* = 10.6, 3.5 Hz, 2H), 3.29 – 3.18 (m, 2H), 2.66 – 2.40 (m, 1H), 1.79 – 1.66 (m, 4H), 1.52 (t, *J* = 10.6 Hz, 2H), 1.42 (s, 9H), 1.06 (td, *J* = 3.5, 1.7 Hz, 1H).

^{13}C NMR (126 MHz, $CDCl_3$) δ 155.32, 154.86, 136.96, 128.58, 128.06, 127.94, 81.70, 79.85, 79.64, 67.12, 47.97, 47.71, 42.43, 31.64, 28.54, 27.00, 26.45, 25.70, 11.30.

HRMS (ESI-TOF) *m/z* calcd. For $C_{25}H_{32}N_2O_4Na^+$ ($[M+Na]^+$) 447.2254, found 447.2258.

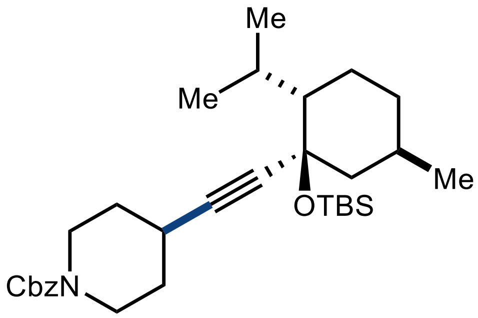

benzyl 4-(((1R,2S,5R)-1-((tert-butyldimethylsilyl)oxy)-2-isopropyl-5-methylcyclohexyl)ethynyl)piperidine-1-carboxylate (32): The title compound was prepared according to **General Procedure B** with 4-hydroxypiperidine-1-carboxylate (alcohol) and (((1R,2S,5R)-1-(bromoethynyl)-2-isopropyl-5-methylcyclohexyl)oxy)(tert-butyl)dimethylsilane (alkynyl bromide, prepared according to known procedure[4]). Purified by automated flash chromatography (25 g high performance silica column, 0–80% ethyl acetate/hexanes gradient) followed by preparative HPLC (XBridge BEH C18 OBD column, 30–60% $MeCN/H_2O$ with 0.1% NH_4OH) to provide the title compound as a yellow oil. (220.3mg, 86% yield).

^{1}H NMR (500 MHz, $CDCl_3$) δ 7.42 – 7.27 (m, 5H), 5.14 (s, 2H), 3.85 – 3.74 (m, 2H), 3.26 (ddd, J = 13.4, 8.7, 3.4 Hz, 2H), 2.63 (tt, J = 8.1, 3.9 Hz, 1H), 2.30 (pd, J = 7.0, 1.9 Hz, 1H), 1.91 (ddd, J = 13.5, 3.6, 2.3 Hz, 1H), 1.86 – 1.69 (m, 4H), 1.65 – 1.55 (m, 2H), 1.47 – 1.35 (m, 2H), 1.23 (dd, J = 13.5, 12.1 Hz, 1H), 1.14 (ddd, J = 11.8, 4.2, 1.9 Hz, 1H), 1.05 – 0.94 (m, 1H), 0.92 – 0.82 (m, 18H), 0.17 (d, J = 12.8 Hz, 6H).

^{13}C NMR (126 MHz, $CDCl_3$) δ 155.34, 136.98, 128.61, 128.10, 127.99, 87.07, 85.90, 73.17, 67.21, 52.81, 51.16, 42.65, 35.24, 31.51, 28.37, 27.40, 27.12, 26.32, 24.49, 22.15, 21.16, 18.71, 18.60, -2.51, -2.55.

HRMS (ESI-TOF) *m*/*z* calcd. For $C_{31}H_{49}NO_3SiNa^+$ ($[M+Na]^+$) 534.3374, found 534.3375.

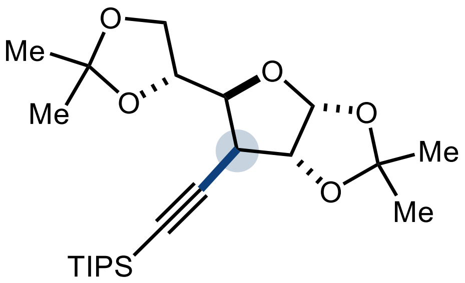

(((3aR,5S,6S,6aR)-5-((R)-2,2-dimethyl-1,3-dioxolan-4-yl)-2,2-dimethyltetrahydrofuro[2,3-d][1,3]dioxol-6-yl)ethynyl)triisopropylsilane (33): The title compound was prepared according to **General Procedure A** with 1,2:5,6-Di-O-isopropylidene-a-D-gulofuranose (alcohol) and (bromoethynyl)triisopropylsilane (alkynyl bromide). Purified by automated flash chromatography (25 g high performance silica column, 0–80% ethyl acetate/hexanes gradient) followed by preparative HPLC (XBridge BEH C18 OBD column, 30–100% MeCN/H_2O with 0.1% NH_4OH) to provide the title compound as a yellow oil. (172.9 mg, 82% yield, d.r. > 20:1). (relative configuration is determined by the NOESY analysis of deprotection-bromination product **58**)

^{1}H NMR (500 MHz, $CDCl_3$) δ 5.80 (d, J = 4.0 Hz, 1H), 4.73 (dd, J = 4.1, 3.1 Hz, 1H), 4.28 (q, J = 6.6 Hz, 1H), 4.08 (dd, J = 8.4, 6.6 Hz, 1H), 3.92 (dd, J = 8.4, 7.1 Hz, 1H), 3.86 (dd, J = 7.8, 6.2 Hz, 1H), 2.95 (dd, J = 7.7, 3.1 Hz, 1H), 1.56 (s, 3H), 1.43 (s, 3H), 1.38 (d, J = 2.4 Hz, 6H), 1.05 (t, J = 3.6 Hz, 21H).

^{13}C NMR (126 MHz, $CDCl_3$) δ 114.59, 110.12, 105.58, 104.78, 87.80, 85.39, 84.30, 76.56, 65.88, 40.52, 28.05, 27.51, 26.52, 25.65, 18.71, 11.24.

HRMS (ESI-TOF) *m/z* calcd. For $C_{23}H_{40}O_5SiNa^+$ ($[M+Na]^+$) 447.2537, found 447.2540.

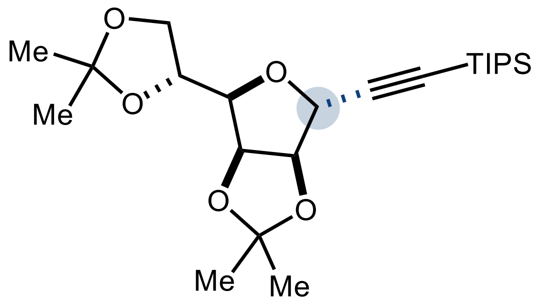

(((3aR,4R,6R,6aS)-6-((R)-2,2-dimethyl-1,3-dioxolan-4-yl)-2,2-dimethyltetrahydrofuro[3,4-d][1,3]dioxol-4-yl)ethynyl)triisopropylsilane (34): The title compound was prepared according to **General Procedure A** with (3aS,4S,6R,6aS)-6-((R)-2,2-dimethyl-1,3-dioxolan-4-yl)-2,2-dimethyltetrahydrofuro[3,4-d][1,3]dioxol-4-ol (alcohol) and (bromoethynyl)triisopropylsilane (alkynyl bromide). Purified by automated flash chromatography (25 g high performance silica column, 0–80% ethyl acetate/hexanes gradient) followed by preparative HPLC (XBridge BEH C18 OBD column, 30–100% $MeCN/H_2O$ with 0.1% NH_4OH) to provide the title compound as a yellow oil. (142.4 mg, 67% yield, d.r. >20:1). (relative configuration is determined by NOESY analysis)

^{1}H NMR (500 MHz, $CDCl_3$) δ 4.82 – 4.77 (m, 2H), 4.69 (s, 1H), 4.43 – 4.37 (m, 1H), 4.10 (dd, *J* = 8.7, 6.2 Hz, 1H), 4.03 (dd, *J* = 8.7, 4.5 Hz, 1H), 3.93 (dd, *J* = 8.2, 3.1 Hz, 1H), 1.47 (s, 3H), 1.43 (s, 3H), 1.38 (s, 3H), 1.33 (s, 3H), 1.06 (d, *J* = 2.0 Hz, 21H).

^{13}C NMR (126 MHz, $CDCl_3$) δ 113.04, 109.44, 103.15, 89.38, 86.75, 81.36, 80.47, 74.59, 73.02, 67.36, 27.02, 26.08, 25.49, 24.89, 18.68, 18.67, 11.16.

HRMS (ESI-TOF) *m/z* calcd. For $C_{23}H_{40}O_5SiNa^+$ ($[M+Na]^+$) 447.2537, found 447.2541.

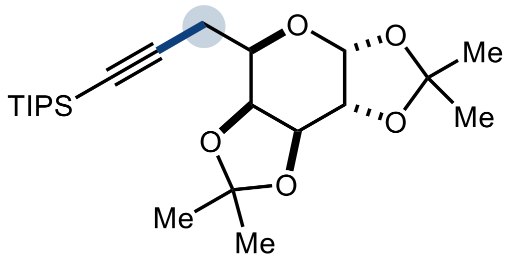

triisopropyl(3-((3aR,5R,5aS,8aS,8bR)-2,2,7,7-tetramethyltetrahydro-5H-bis([1,3]dioxolo)[4,5-b:4',5'-d]pyran-5-yl)prop-1-yn-1-yl)silane (35): The title compound was prepared according to **General Procedure A** with ((3aR,5R,5aS,8aS,8bR)-2,2,7,7-tetramethyltetrahydro-5H-bis([1,3]dioxolo)[4,5-b:4',5'-d]pyran-5-yl)methanol (alcohol) and (bromoethynyl)triisopropylsilane (alkynyl bromide). Purified by automated flash chromatography (25 g high performance silica column, 0–80% ethyl acetate/hexanes gradient) followed by preparative HPLC (XBridge BEH C18 OBD column, 30–100% MeCN/H_2O with 0.1% NH_4OH) to provide the title compound as a yellow oil. (173.5 mg, 82% yield).

^{1}H NMR (500 MHz, $CDCl_3$) δ 5.50 (d, *J* = 5.0 Hz, 1H), 4.60 (dd, *J* = 8.0, 2.3 Hz, 1H), 4.35 (dd, *J* = 8.0, 1.8 Hz, 1H), 4.28 (dd, *J* = 5.0, 2.3 Hz, 1H), 3.96 – 3.88 (m, 1H), 2.64 (dd, *J* = 16.5, 8.7 Hz, 1H), 2.53 (dd, *J* = 16.6, 6.1 Hz, 1H), 1.52 (s, 3H), 1.44 (s, 3H), 1.33 (s, 6H), 1.15 – 0.96 (m, 21H).

^{13}C NMR (126 MHz, $CDCl_3$) δ 109.21, 108.63, 104.66, 96.69, 82.27, 71.41, 70.91, 70.80, 67.20, 26.18, 26.08, 25.12, 24.28, 22.05, 18.75, 11.40.

HRMS (ESI-TOF) *m/z* calcd. For $C_{23}H_{40}O_5SiNa^+$ ($[M+Na]^+$) 447.2537, found 447.2540.

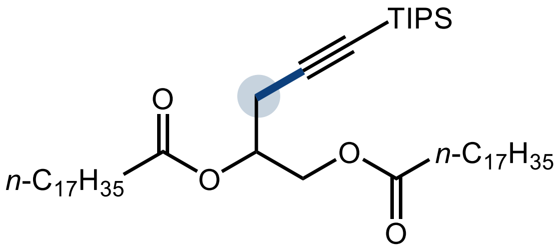

5-(triisopropylsilyl)pent-4-yne-1,2-diyl distearate (36): The title compound was prepared according to **General Procedure A** in 0.2 mmol scale with 1,2-distearoyl-sn-glycerol (alcohol) and (bromoethynyl)triisopropylsilane (alkynyl bromide). Purified by automated flash chromatography (25 g high performance silica column, 0–80% ethyl acetate/hexanes gradient) followed by preparative HPLC (XBridge BEH C18 OBD column, 30–100% MeCN/H_2O with 0.1% NH_4OH) to provide the title compound as a yellow oil. (103.2 mg, 65% yield).

^{1}H NMR (500 MHz, $CDCl_3$) δ 5.07 (qd, *J* = 6.2, 3.7 Hz, 1H), 4.29 (dd, *J* = 11.8, 3.7 Hz, 1H), 4.14 (dd, *J* = 11.8, 6.1 Hz, 1H), 2.62 – 2.48 (m, 2H), 2.23 (td, *J* = 7.6, 3.4 Hz, 4H), 1.54 (p, *J* = 7.3 Hz, 4H), 1.25 – 1.15 (m, 56H), 1.03 – 0.93 (m, 21H), 0.81 (t, *J* = 6.9 Hz, 6H).

^{13}C NMR (126 MHz, $CDCl_3$) δ 173.44, 173.07, 102.46, 83.74, 69.53, 63.84, 34.47, 34.28, 32.08, 29.86, 29.82, 29.79, 29.64, 29.52, 29.44, 29.29, 25.08, 25.06, 22.84, 22.45, 18.72, 14.26, 11.34. (aliphatic carbon resonances overlap in the ^{13}C NMR spectra)

HRMS (ESI-TOF) *m/z* calcd. For $C_{50}H_{96}O_4SiNa^+$ ($[M+Na]^+$) 811.6970, found 811.6962.

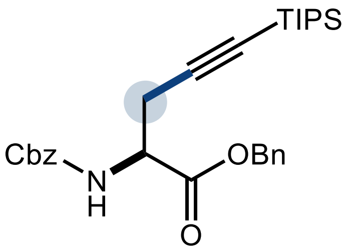

benzyl (S)-2-(((benzyloxy)carbonyl)amino)-5-(triisopropylsilyl)pent-4-ynoate (37): The title compound was prepared according to **General Procedure A** with *Z*-*L*-serine benzyl ester (alcohol) and (bromoethynyl)triisopropylsilane (alkynyl bromide). Purified by automated flash chromatography (25 g high performance silica column, 0–80% ethyl acetate/hexanes gradient) followed by preparative HPLC (XBridge BEH C18 OBD column, 30–80% MeCN/H_2O with 0.1% NH_4OH) to provide the title compound as a yellow oil. (202.3 mg, 82% yield).

^{1}H NMR (500 MHz, $CDCl_3$) δ 7.34 (q, *J* = 6.6, 5.7 Hz, 10H), 5.61 (d, *J* = 8.3 Hz, 1H), 5.18 (q, *J* = 12.2 Hz, 2H), 5.13 (s, 2H), 4.57 (dt, *J* = 8.8, 4.7 Hz, 1H), 2.87 (d, *J* = 4.7 Hz, 2H), 1.03 (d, *J* = 5.0 Hz, 21H).

^{13}C NMR (126 MHz, $CDCl_3$) δ 170.35, 155.76, 136.41, 135.28, 128.77, 128.67, 128.62, 128.36, 128.28, 128.10, 101.98, 84.97, 67.66, 67.14, 52.81, 24.27, 18.69, 11.26.

HRMS (ESI-TOF) *m*/*z* calcd. For $C_{29}H_{39}NO_4SiNa^+$ ($[M+Na]^+$) 516.2541, found 516.2536.

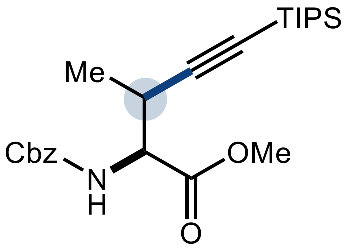

methyl (2S,3S)-2-(((benzyloxy)carbonyl)amino)-3-methyl-5-(triisopropylsilyl)pent-4-ynoate (38): The title compound was prepared according to **General Procedure A** with Z-Thr-OMe (alcohol) and (bromoethynyl)triisopropylsilane (alkynyl bromide). Purified by automated flash chromatography (25 g high performance silica column, 0–80% ethyl acetate/hexanes gradient) followed by preparative HPLC (XBridge BEH C18 OBD column, 30–80% MeCN/H_2O with 0.1% NH_4OH) to provide the title compound as a yellow oil. (157.3 mg, 73% yield, d.r. = 1.7:1).

^{1}H NMR (500 MHz, $CDCl_3$) δ 7.42 – 7.28 (m, 8H), 5.51 (d, *J* = 8.9 Hz, 0.52H), 5.42 (d, *J* = 9.7 Hz, 1H), 5.13 (d, *J* = 15.8 Hz, 3.3H), 4.48 – 4.23 (m, 1.6H), 3.74 (d, *J* = 3.2 Hz, 4.8H), 3.24 (qd, *J* = 7.1, 3.6 Hz, 1H), 3.02 (dt, *J* = 12.3, 6.0 Hz, 0.6H), 1.28 (dd, *J* = 10.0, 7.1 Hz, 5.3H), 1.15 – 0.91 (m, 34.0H). (mixture of diastereomers)

^{13}C NMR (126 MHz, $CDCl_3$) δ 170.92, 156.46, 136.37, 128.54, 128.51, 128.19, 128.10, 128.08, 127.86, 106.68, 84.33, 67.08, 67.03, 57.67, 57.61, 52.52, 52.29, 31.17, 30.75, 18.54, 18.32, 17.84, 11.13, 11.09. (mixture of diastereomers)

HRMS (ESI-TOF) *m/z* calcd. For $C_{24}H_{37}NO_4SiNa^+$ ($[M+Na]^+$) 454.2384, found 454.2382.

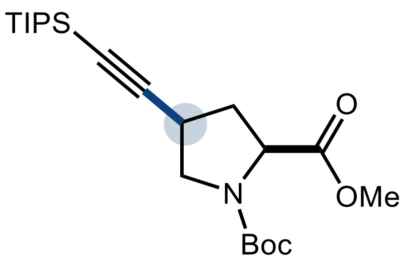

1-(tert-butyl) 2-methyl (2S,4R)-4-((triisopropylsilyl)ethynyl)pyrrolidine-1,2-dicarboxylate (39): The title compound was prepared according to **General Procedure A** with *N*-Boc-trans-4-hydroxy-*L*-proline methyl ester (alcohol) and (bromoethynyl)triisopropylsilane (alkynyl bromide). Purified by automated flash chromatography (25 g high performance silica column, 0–80% ethyl acetate/hexanes gradient) followed by preparative HPLC (XBridge BEH C18 OBD column, 30–60% MeCN/H_2O with 0.1% NH_4OH) to provide the title compound as a yellow oil. (172.1 mg, 84% yield, d.r. = 1.3:1).

^{1}H NMR (500 MHz, $CDCl_3$) δ 4.40 (dd, *J* = 7.8, 4.3 Hz, 0.85H), 4.32 (dd, *J* = 8.4, 4.4 Hz, 1H), 3.88 – 3.71 (m, 3.60H), 3.71 (d, *J* = 3.3 Hz, 5H), 3.46 (dd, *J* = 10.3, 7.0 Hz, 1H), 3.35 (dd, *J* = 10.3, 7.5 Hz, 0.85H), 3.20 – 3.08 (m, 1.8H), 2.34 – 2.17 (m, 3.8H), 1.45 (s, 8H), 1.39 (s, 9H), 1.03 (t, *J* = 4.0 Hz, 42H). (mixture of diastereomers)

^{13}C NMR (126 MHz, $CDCl_3$) δ 173.26, 173.08, 154.15, 153.51, 107.40, 107.22, 82.82, 82.72, 80.35, 80.28, 58.73, 58.36, 52.41, 52.35, 52.32, 52.18, 37.74, 36.89, 29.88, 29.23, 28.50, 28.36, 18.68, 11.23. (mixture of diastereomers)

HRMS (ESI-TOF) *m*/*z* calcd. For $C_{22}H_{39}NO_4SiNa^+$ ($[M+Na]^+$) 432.2541, found 432.2542.

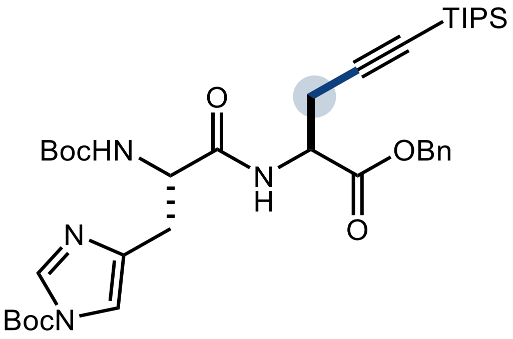

tert-butyl 4-((S)-3-(((S)-1-(benzyloxy)-1-oxo-5-(triisopropylsilyl)pent-4-yn-2-yl)amino)-2-((tert-butoxycarbonyl)amino)-3-oxopropyl)-1H-imidazole-1-carboxylate (40): The title compound was prepared according to **General Procedure A** with tert-butyl 4-((S)-3-(((S)-1-(benzyloxy)-3-hydroxy-1-oxopropan-2-yl)amino)-2-((tert-butoxycarbonyl)amino)-3-oxopropyl)-1H-imidazole-1-carboxylate (alcohol) and (bromoethynyl)triisopropylsilane (alkynyl bromide). Purified by automated flash chromatography (25 g high performance silica column, 0–80% ethyl acetate/hexanes gradient) followed by preparative HPLC (XBridge BEH C18 OBD column, 30–100% MeCN/H_2O with 0.1% NH_4OH) to provide the title compound as a yellow oil. (231.7 mg, 66% yield).

^{1}H NMR (500 MHz, $CDCl_3$) δ 7.95 (d, J = 1.4 Hz, 1H), 7.55 – 7.38 (m, 1H), 7.38 – 7.28 (m, 5H), 7.15 (s, 1H), 5.99 (s, 1H), 5.14 (d, J = 2.1 Hz, 2H), 4.76 (dt, J = 8.7, 4.6 Hz, 1H), 4.56 – 4.24 (m, 1H), 3.23 – 3.01 (m, 1H), 2.93 (dd, J = 14.9, 5.5 Hz, 1H), 2.81 (dd, J = 17.0, 4.3 Hz, 1H), 2.60 (dd, J = 16.9, 5.0 Hz, 1H), 1.59 (s, 9H), 1.44 (s, 9H), 1.09 – 0.97 (m, 21H).

^{13}C NMR (126 MHz, $CDCl_3$) δ 171.13, 169.82, 155.66, 146.99, 139.19, 136.89, 135.28, 128.67, 128.49, 128.25, 114.79, 102.18, 85.60, 84.19, 67.44, 54.38, 51.17, 30.32, 28.39, 27.97, 24.14, 18.70, 18.68, 11.22.

HRMS (ESI-TOF) *m*/*z* calcd. For $C_{37}H_{56}N_4O_7SiNa^+$ ($[M+Na]^+$) 719.3811, found 719.3819.

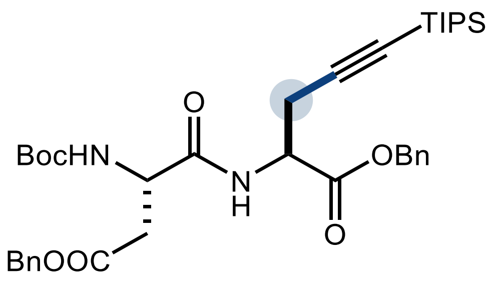

benzyl (S)-2-((S)-4-(benzyloxy)-2-((tert-butoxycarbonyl)amino)-4-oxobutanamido)-5-(triisopropylsilyl)pent-4-ynoate (41): The title compound was prepared according to **General Procedure A** with benzyl (S)-4-(((S)-1-(benzyloxy)-3-hydroxy-1-oxopropan-2-yl)amino)-3-((tert-butoxycarbonyl)amino)-4-oxobutanoate (alcohol) and (bromoethynyl)triisopropylsilane (alkynyl bromide). Purified by automated flash chromatography (25 g high performance silica column, 0–80% ethyl acetate/hexanes gradient) followed by preparative HPLC (XBridge BEH C18 OBD column, 30–100% MeCN/H_2O with 0.1% NH_4OH) to provide the title compound as a yellow oil. (265.7 mg, 80% yield).

^{1}H NMR (500 MHz, Acetone) δ 7.59 (d, J = 8.1 Hz, 1H), 7.48 – 7.28 (m, 10H), 6.31 (d, J = 8.4 Hz, 1H), 5.26 – 5.16 (m, 2H), 5.13 (s, 2H), 4.72 (dt, J = 7.9, 5.3 Hz, 1H), 4.59 (t, J = 7.2 Hz, 1H), 2.91 – 2.78 (m, 4H), 1.43 (s, 9H), 1.16 – 0.94 (m, 21H).

^{13}C NMR (126 MHz, Acetone) δ 171.40, 171.36, 170.65, 156.32, 137.21, 136.76, 129.32, 129.25, 128.98, 128.87, 128.83, 128.77, 104.12, 83.99, 79.99, 67.62, 66.85, 52.42, 52.33, 52.05, 37.07, 28.52, 23.90, 19.01, 11.90.

HRMS (ESI-TOF) *m/z* calcd. For $C_{37}H_{53}N_2O_7Si^+$ ([M+H]$^+$) 665.3617, found 665.3617.

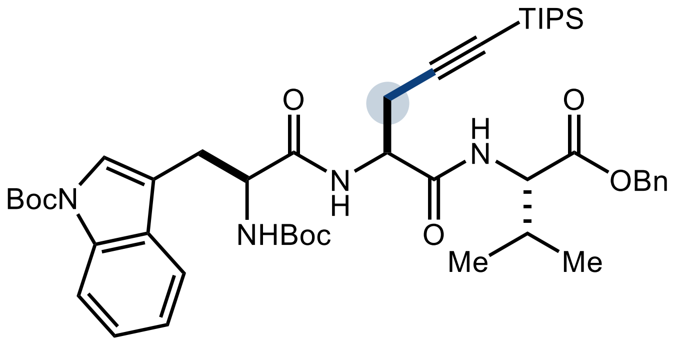

tert-butyl 3-((S)-3-(((S)-1-(((S)-1-(benzyloxy)-3-methyl-1-oxobutan-2-yl)amino)-1-oxo-5-(triisopropylsilyl)pent-4-yn-2-yl)amino)-2-((tert-butoxycarbonyl)amino)-3-oxopropyl)-1H-indole-1-carboxylate (42): The title compound was prepared according to **General Procedure** in 0.2 mmol scale with tert-butyl 3-((S)-3-(((S)-1-(((S)-1-(benzyloxy)-3-methyl-1-oxobutan-2-yl)amino)-3-hydroxy-1-oxopropan-2-yl)amino)-2-((tert-butoxycarbonyl)amino)-3-oxopropyl)-1H-indole-1-carboxylate (alcohol) and (bromoethynyl)triisopropylsilane (alkynyl bromide). Purified by automated flash chromatography (25 g high performance silica column, 0–80% ethyl acetate/hexanes gradient) followed by preparative HPLC (XBridge BEH C18 OBD column, 30–100% MeCN/H_2O with 0.1% NH_4OH) to provide the title compound as a yellow solid. (103.5 mg, 61% yield).

^{1}H NMR (500 MHz, $CDCl_3$) δ 8.12 (d, J = 8.2 Hz, 1H), 7.56 (dt, J = 7.8, 0.9 Hz, 1H), 7.44 (s, 1H), 7.39 – 7.28 (m, 6H), 7.23 (td, J = 7.5, 1.0 Hz, 1H), 6.88 (d, J = 7.5 Hz, 1H), 6.74 (d, J = 8.5 Hz, 1H), 5.20 (d, J = 12.2 Hz, 1H), 5.10 (d, J = 12.2 Hz, 1H), 4.90 (s, 1H), 4.55 (td, J = 7.0, 5.1 Hz, 1H), 4.50 (dt, J = 10.0, 5.1 Hz, 2H), 3.23 (p, J = 6.5, 6.0 Hz, 2H), 2.79 (dd, J = 17.1, 5.1 Hz, 1H), 2.55 (dd, J = 17.1, 6.7 Hz, 1H), 2.14 (pd, J = 6.9, 5.1 Hz, 1H), 1.66 (s, 9H), 1.38 (s, 9H), 0.99 (q, J = 3.9 Hz, 21H), 0.86 (dd, J = 14.3, 6.9 Hz, 6H).

^{13}C NMR (126 MHz, CDCl$_3$) δ 171.63, 171.19, 169.41, 155.56, 149.62, 135.70, 135.48, 130.38, 128.70, 128.55, 124.83, 124.39, 122.81, 119.06, 115.50, 115.26, 102.91, 84.36, 83.80, 80.65, 67.13, 57.74, 54.90, 51.90, 31.30, 28.34, 28.30, 27.87, 23.19, 19.05, 18.71, 18.03, 11.23.

HRMS (ESI-TOF) *m/z* calcd. For $C_{47}H_{68}N_4O_8SiNa^+$ ([M+Na]$^+$) 867.4699, found 867.4696.

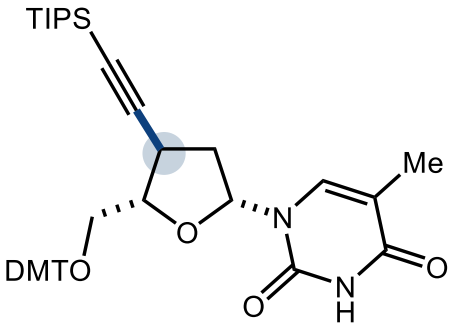

1-((2R,4R,5S)-5-((bis(4-methoxyphenyl)(phenyl)methoxy)methyl)-4-((triisopropylsilyl)ethynyl)tetrahydrofuran-2-yl)-5-methylpyrimidine-2,4(1H,3H)-dione (43): The title compound was prepared according to **General Procedure A** with 5'-O-(4,4'-Dimethoxytrityl)thymidine (alcohol) and (bromoethynyl)triisopropylsilane (alkynyl bromide). Purified by automated flash chromatography (25 g high performance silica column, 0–80% ethyl acetate/hexanes gradient) followed by preparative HPLC (XBridge BEH C18 OBD column, 30–100% MeCN/H_2O with 0.1% NH_4OH) to provide the title compound as a yellow solid. (235.9 mg, 67% yield, d.r. >20:1). (relative configuration is determined by NOESY analysis of deprotection product **43**)

^{1}H NMR (500 MHz, Acetone) δ 10.07 (s, 1H), 7.68 (d, *J* = 1.6 Hz, 1H), 7.52 (dd, *J* = 7.5, 1.7 Hz, 2H), 7.42 – 7.36 (m, 4H), 7.31 (t, *J* = 7.7 Hz, 2H), 7.26 – 7.21 (m, 1H), 7.00 – 6.80 (m, 4H), 6.22 (dd, *J* = 7.3, 3.1 Hz, 1H), 4.11 (ddd, *J* = 9.6, 4.1, 2.3 Hz, 1H), 3.78 (s, 6H), 3.59 – 3.48 (m, 2H), 3.42 (dd, *J* = 10.7, 4.0 Hz, 1H), 2.67 – 2.51 (m, 2H), 1.57 (s, 3H), 1.10 – 0.93 (m, 21H).

^{13}C NMR (126 MHz, Acetone) δ 159.69, 159.66, 151.22, 151.14, 145.99, 136.57, 136.52, 130.96, 128.93, 128.69, 127.65, 113.97, 110.58, 107.31, 87.12, 85.72, 85.61, 83.40, 63.04, 55.49, 40.15, 31.46, 18.95, 12.41, 11.83.

HRMS (ESI-TOF) *m/z* calcd. For $C_{42}H_{52}N_2O_6SiNa^+$ ([M+Na]$^+$) 731.3487, found 731.3488.

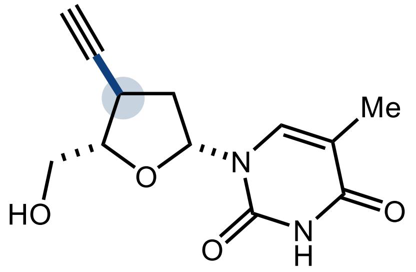

1-((2R,4R,5S)-4-ethynyl-5-(hydroxymethyl)tetrahydrofuran-2-yl)-5-methylpyrimidine-2,4(1H,3H)-dione (44): The title compound was prepared by deprotection of **43** according to **General Procedure C** (no purification needed) followed by reported procedure of deprotection of dimethoxytriyl (DMT) group.[3] Purified by automated flash chromatography (25 g high performance silica column, 0–100% ethyl acetate/hexanes gradient) followed by preparative HPLC (XBridge BEH C18 OBD column, 20–50% MeCN/H_2O with 0.1% NH_4OH) to provide the title compound as a yellow oil with total yield of 95% for two steps. (relative configuration is determined by NOESY analysis)

^{1}H NMR (500 MHz, $CDCl_3$) δ 8.61 (s, 1H), 7.49 (d, J = 1.3 Hz, 1H), 6.14 (dd, J = 7.3, 3.5 Hz, 1H), 4.07 (dd, J = 12.2, 2.3 Hz, 1H), 4.03 (dt, J = 8.9, 2.5 Hz, 1H), 3.86 (dd, J = 12.2, 2.7 Hz, 1H), 3.25 (dtd, J = 11.0, 8.7, 2.4 Hz, 1H), 2.56 – 2.43 (m, 2H), 2.18 (d, J = 2.4 Hz, 1H), 1.90 (d, J = 1.2 Hz, 3H).

^{13}C NMR (126 MHz, Acetone) δ 164.37, 151.26, 137.09, 110.17, 86.98, 85.53, 83.15, 72.24, 61.11, 39.86, 29.26, 12.54.

HRMS (ESI-TOF) *m/z* calcd. For $C_{12}H_{14}N_2O_4SiNa^+$ ($[M+Na]^+$) 273.0846, found 273.0844.

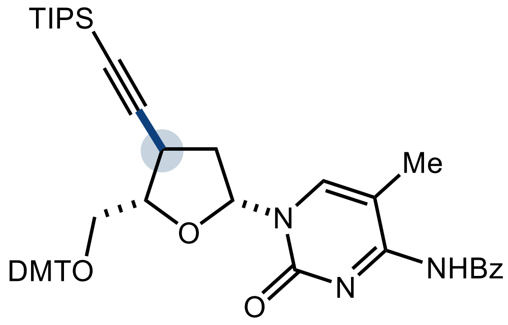

N-(1-((2R,4R,5S)-5-((bis(4-methoxyphenyl)(phenyl)methoxy)methyl)-4-((triisopropylsilyl)ethynyl)tetrahydrofuran-2-yl)-5-methyl-2-oxo-1,2-dihydropyrimidin-4-yl)benzamide (45): The title compound was prepared according to **General Procedure A** with 5'-O-(4,4'-Dimethoxytrityl)-n4-benzoyl-5-methyl-2'-deoxycytidine (alcohol) and (bromoethynyl)triisopropylsilane (alkynyl bromide). Purified by automated flash chromatography (25 g high performance silica column, 0–80% ethyl acetate/hexanes gradient) followed by preparative HPLC (XBridge BEH C18 OBD column, 30–100% $MeCN/H_2O$ with 0.1% NH_4OH) to provide the title compound as a yellow solid. (183.7 mg, 45% yield, d.r. >20:1). (relative configuration is determined by analysis of the ^3J coupling constants in comparison with **43**)

^{1}H NMR (500 MHz, Acetone) δ 13.45 (s, 1H), 8.34 – 8.26 (m, 2H), 8.00 (d, J = 1.4 Hz, 1H), 7.56 – 7.52 (m, 3H), 7.46 (dd, J = 8.3, 7.0 Hz, 2H), 7.43 – 7.37 (m, 4H), 7.34 (dd, J = 8.5, 7.0 Hz, 2H), 7.27 – 7.23 (m, 1H), 6.94 – 6.88 (m, 4H), 6.21 (dd, J = 7.2, 2.2 Hz, 1H), 4.19 (ddd, J = 9.9, 3.7, 2.0 Hz, 1H), 3.79 (s, 6H), 3.63 – 3.54 (m, 2H), 3.50 (dd, J = 10.8, 3.8 Hz, 1H), 2.75 – 2.67 (m, 1H), 2.66 – 2.58 (m, 1H), 1.75 (d, J = 1.1 Hz, 3H), 1.04 (t, J = 2.7 Hz, 21H).

^{13}C NMR (126 MHz, Acetone) δ 159.75, 159.72, 148.35, 145.91, 139.15, 138.38, 136.48, 136.43, 133.10, 131.01, 130.98, 130.52, 128.97, 128.91, 128.77, 127.74, 114.04, 111.24, 106.73, 87.27, 86.75, 86.36, 83.69, 62.67, 55.52, 40.52, 31.02, 18.97, 11.83.

HRMS (ESI-TOF) *m*/*z* calcd. For $C_{49}H_{57}N_3O_6SiNa^+$ ($[M+Na]^+$) 834.3909, found 834.3913.

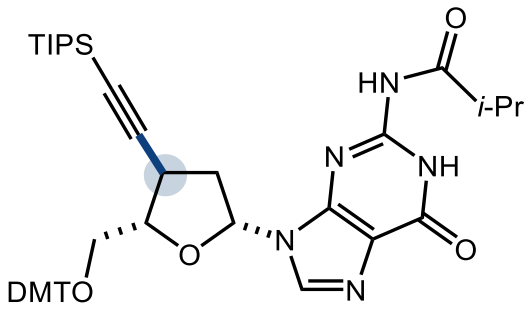

N-(9-((2R,4R,5S)-5-((bis(4-methoxyphenyl)(phenyl)methoxy)methyl)-4-((triisopropylsilyl)ethynyl)tetrahydrofuran-2-yl)-6-oxo-6,9-dihydro-1H-purin-2-yl)isobutyramide (46): The title compound was prepared according to **General Procedure A** with N2-Isobutyryl-5'-O-(4,4'-dimethoxytrityl)-2'-deoxyguanosine (alcohol) and (bromoethynyl)triisopropylsilane (alkynyl bromide). Purified by automated flash chromatography (25 g high performance silica column, 0–80% ethyl acetate/hexanes gradient) followed by preparative HPLC (XBridge BEH C18 OBD column, 30–100% MeCN/H_2O with 0.1% NH_4OH) to provide the title compound as a yellow solid. (94.7 mg, 24% yield, d.r. >20:1). (relative configuration is determined by analysis of the ^3J coupling constants in comparison with **43**)

^{1}H NMR (500 MHz, Acetone) δ 8.05 (s, 1H), 7.45 – 7.38 (m, 2H), 7.30 – 7.26 (m, 4H), 7.23 (td, *J* = 7.2, 6.3, 1.3 Hz, 2H), 7.20 – 7.14 (m, 1H), 6.80 (dd, *J* = 8.9, 6.2 Hz, 4H), 6.31 (dd, *J* = 7.7, 1.9 Hz, 1H), 4.20 (ddd, *J* = 9.7, 5.0, 2.7 Hz, 1H), 3.75 (d, *J* = 1.8 Hz, 7H), 3.64 (t, *J* = 5.3 Hz, 1H), 3.43 – 3.36 (m, 2H), 3.03 – 2.97 (m, 1H), 2.93 (p, *J* = 6.7 Hz, 1H), 2.72 – 2.64 (m, 1H), 2.04 (s, 1H), 1.20 (dd, *J* = 6.8, 2.1 Hz, 6H), 1.08 – 0.98 (m, 21H).

^{13}C NMR (126 MHz, Acetone) δ 180.43, 159.55, 159.53, 149.57, 149.36, 146.05, 137.96, 136.70, 136.68, 130.89, 130.85, 128.93, 128.52, 127.45, 113.82, 107.70, 86.86, 86.53, 85.15, 83.28, 63.94, 55.46, 39.63, 36.44, 32.27, 18.98, 11.87.

HRMS (ESI-TOF) *m*/*z* calcd. For $C_{46}H_{58}N_5O_6Si^+$ ([M+H]$^+$) 804.4151, found 804.4152.

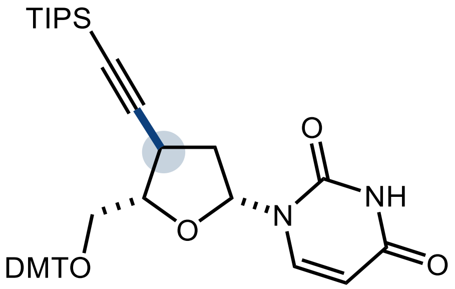

1-((2R,4R,5S)-5-((bis(4-methoxyphenyl)(phenyl)methoxy)methyl)-4-((triisopropylsilyl)ethynyl)tetrahydrofuran-2-yl)pyrimidine-2,4(1H,3H)-dione (47): The title compound was prepared according to **General Procedure A** with 5'-O-(4,4'-Dimethoxytrityl)-2'-deoxyuridine (alcohol) and (bromoethynyl)triisopropylsilane (alkynyl bromide). Purified by automated flash chromatography (25 g high performance silica column, 0–80% ethyl acetate/hexanes gradient) followed by preparative HPLC (XBridge BEH C18 OBD column, 30–100% MeCN/H_2O with 0.1% NH_4OH) to provide the title compound as a yellow solid. (231.9 mg, 67% yield, d.r. > 20:1). (relative configuration is determined by analysis of the ^3J coupling constants in comparison with **43**)

^{1}H NMR (500 MHz, Acetone) δ 10.05 (s, 1H), 7.98 (dd, J = 8.2, 2.0 Hz, 1H), 7.50 (d, J = 7.8 Hz, 2H), 7.44 – 7.20 (m, 7H), 6.90 (d, J = 8.5 Hz, 4H), 6.17 (dd, J = 7.0, 2.5 Hz, 1H), 5.31 (d, J = 8.1 Hz, 1H), 4.12 (dt, J = 10.1, 2.9 Hz, 1H), 3.79 (s, 6H), 3.65 – 3.40 (m, 3H), 2.71 – 2.46 (m, 2H), 1.05 (t, J = 2.8 Hz, 21H).

^{13}C NMR (126 MHz, Acetone) δ 162.84, 158.85, 150.30, 145.05, 140.31, 135.63, 135.46, 130.14, 130.09, 128.07, 127.86, 126.83, 113.15, 106.04, 101.21, 86.49, 85.21, 85.14, 82.72, 61.65, 54.65, 39.69, 30.23, 18.11, 10.98.

HRMS (ESI-TOF) *m/z* calcd. For $C_{41}H_{50}N_2O_6SiNa^+$ ($[M+Na]^+$) 717.3330, found 717.3335.

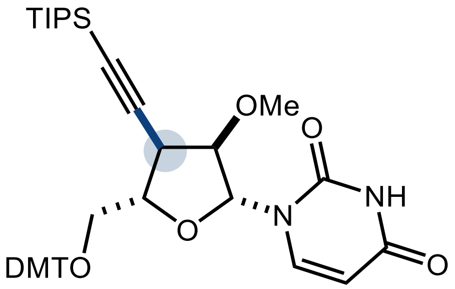

1-((2R,3R,4R,5S)-5-((bis(4-methoxyphenyl)(phenyl)methoxy)methyl)-3-methoxy-4-((triisopropylsilyl)ethynyl)tetrahydrofuran-2-yl)pyrimidine-2,4(1H,3H)-dione (48): The title compound was prepared according to **General Procedure A** with 1-((2R,3R,4R,5R)-5-((Bis(4-methoxyphenyl)(phenyl)methoxy)methyl)-4-hydroxy-3-methoxytetrahydrofuran-2-yl)pyrimidine-2,4(1H,3H)-dione (alcohol) and (bromoethynyl)triisopropylsilane (alkynyl bromide). Purified by automated flash chromatography (25 g high performance silica column, 0–80% ethyl acetate/hexanes gradient) followed by preparative HPLC (XBridge BEH C18 OBD column, 30–100% MeCN/H_2O with 0.1% NH_4OH) to provide the title compound as a yellow solid. (61.0 mg, 17% yield, d.r. > 20:1). (relative configuration is determined by analysis of the ^3J coupling constants in comparison with **43**)

^{1}H NMR (500 MHz, Acetone) δ 10.06 (s, 1H), 8.02 (d, *J* = 8.1 Hz, 1H), 7.51 – 7.47 (m, 2H), 7.38 – 7.31 (m, 6H), 7.28 – 7.24 (m, 1H), 6.92 – 6.88 (m, 4H), 5.85 (s, 1H), 5.18 (d, *J* = 8.2 Hz, 1H), 4.31 (dt, *J* = 10.8, 2.7 Hz, 1H), 4.14 (d, *J* = 4.8 Hz, 1H), 3.80 (d, *J* = 1.1 Hz, 6H), 3.62 (s, 3H), 3.60 – 3.52 (m, 3H), 1.06 (td, *J* = 2.3, 1.9, 1.0 Hz, 21H).

^{13}C NMR (126 MHz, Acetone) δ 163.63, 159.77, 159.74, 151.10, 145.85, 140.68, 136.40, 136.18, 131.06, 130.99, 128.96, 128.77, 127.77, 114.06, 102.78, 101.96, 90.98, 87.47, 86.92, 85.97, 84.63, 62.00, 59.24, 55.53, 36.47, 18.96, 11.90.

HRMS (ESI-TOF) *m*/*z* calcd. For $C_{42}H_{52}N_2O_7SiNa^+$ ($[M+Na]^+$) 747.3460, found 747.3461.

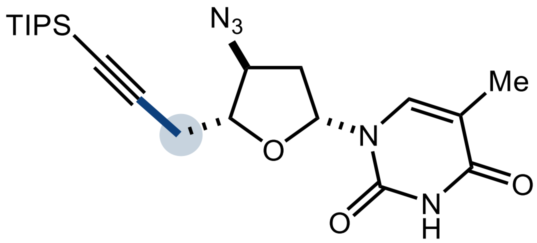

1-((2R,4S,5R)-4-azido-5-(3-(triisopropylsilyl)prop-2-yn-1-yl)tetrahydrofuran-2-yl)-5-methylpyrimidine-2,4(1H,3H)-dione (49): The title compound was prepared according to **General Procedure A** with Zidovudine (alcohol) and (bromoethynyl)triisopropylsilane (alkynyl bromide). Purified by automated flash chromatography (25 g high performance silica column, 0–80% ethyl acetate/hexanes gradient) followed by preparative HPLC (XBridge BEH C18 OBD column, 30–100% MeCN/H_2O with 0.1% NH_4OH) to provide the title compound as a yellow solid. (89.4 mg, 41% yield).

^{1}H NMR (500 MHz, Acetone) δ 10.08 (s, 1H), 7.48 (q, J = 1.2 Hz, 1H), 6.19 (dd, J = 7.5, 6.4 Hz, 1H), 4.44 (dt, J = 7.4, 3.8 Hz, 1H), 4.05 (td, J = 6.3, 3.9 Hz, 1H), 2.84 (d, J = 6.3 Hz, 2H), 2.59 (dt, J = 14.6, 7.4 Hz, 1H), 2.44 (ddd, J = 14.0, 6.4, 3.8 Hz, 1H), 1.84 (d, J = 1.3 Hz, 3H), 1.20 – 0.97 (m, 21H).

^{13}C NMR (126 MHz, Acetone) δ 164.19, 151.23, 136.39, 111.24, 104.77, 85.56, 83.61, 82.71, 64.52, 36.77, 25.34, 18.97, 12.57, 11.98.

HRMS (ESI-TOF) *m/z* calcd. For $C_{21}H_{34}N_5O_3Si^+$ ($[M+H]^+$) 432.2425, found 432.2426.

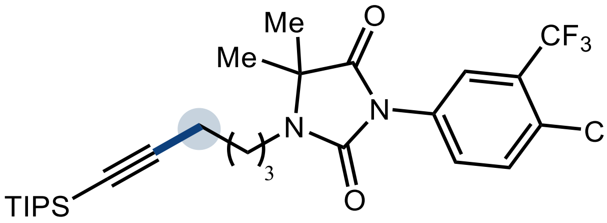

4-(4,4-dimethyl-2,5-dioxo-3-(6-(triisopropylsilyl)hex-5-yn-1-yl)imidazolidin-1-yl)-2-(trifluoromethyl)benzonitrile (50): The title compound was prepared according to **General Procedure A** with RU-58841 (alcohol) and (bromoethynyl)triisopropylsilane (alkynyl bromide). Purified by automated flash chromatography (25 g high performance silica column, 0–80% ethyl acetate/hexanes gradient) followed by preparative HPLC (XBridge BEH C18 OBD column, 30–100% MeCN/H_2O with 0.1% NH_4OH) to provide the title compound as a yellow oil. (184.2 mg, 69% yield).

^{1}H NMR (500 MHz, $CDCl_3$) δ 8.16 (d, J = 2.0 Hz, 1H), 8.01 (dd, J = 8.4, 2.1 Hz, 1H), 7.90 (d, J = 8.4 Hz, 1H), 3.43 – 3.34 (m, 2H), 2.34 (t, J = 6.7 Hz, 2H), 1.92 – 1.81 (m, 2H), 1.61 (p, J = 6.9 Hz, 2H), 1.53 (s, 6H), 1.14 – 0.94 (m, 21H).

^{13}C NMR (126 MHz, $CDCl_3$) δ 174.76, 152.87, 136.67, 135.35, 133.82, 133.56, 127.92, 123.19, 123.10, 123.06, 123.02, 122.98, 121.01, 115.16, 108.29, 108.03, 81.15, 61.94, 40.08, 28.73, 26.33, 23.63, 19.56, 18.74, 11.37. (J_{CF} coupling constants remain unidentified in the data.)

^{19}F NMR (471 MHz, $CDCl_3$) δ -62.03.

HRMS (ESI-TOF) *m/z* calcd. For $C_{28}H_{39}F_3N_3O_2Si^+$ ($[M+H]^+$) 534.2758, found 534.2757.

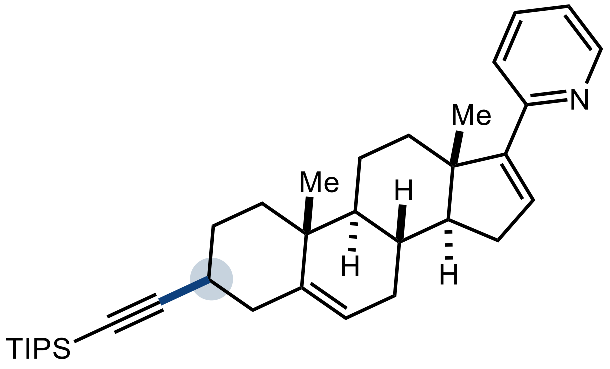

2-((3S,8R,9S,10R,13S,14S)-10,13-dimethyl-3-((triisopropylsilyl)ethynyl)-2,3,4,7,8,9,10,11,12,13,14,15-dodecahydro-1H-cyclopenta[a]phenanthren-17-yl)pyridine (51): The title compound was prepared according to **General Procedure A** with Abiraterone (alcohol) and (bromoethynyl)triisopropylsilane (alkynyl bromide). Purified by automated flash chromatography (25 g high performance silica column, 0–80% ethyl acetate/hexanes gradient) followed by preparative HPLC (XBridge BEH C18 OBD column, 30–100% MeCN/H_2O with 0.1% NH_4OH) to provide the title compound as a yellow oil. (122.6 mg, 48% yield, d.r. = 4:1).

Major product:

^{1}H NMR (500 MHz, $CDCl_3$) δ 8.56 (d, J = 85.8 Hz, 2H), 7.65 (d, J = 7.9 Hz, 1H), 7.22 (dd, J = 8.0, 4.7 Hz, 1H), 6.00 (s, 1H), 5.36 (d, J = 4.8 Hz, 1H), 2.86 (dt, J = 4.0, 2.3 Hz, 1H), 2.61 – 2.41 (m, 1H), 2.28 (ddd, J = 15.8, 6.6, 3.3 Hz, 1H), 2.13 – 2.01 (m, 4H), 1.76 – 1.46 (m, 10H), 1.16 (td, J = 6.6, 3.4 Hz, 1H), 1.08 – 0.91 (m, 27H).

^{13}C NMR (126 MHz, $CDCl_3$) δ 151.95, 148.05, 147.92, 139.82, 133.84, 129.44, 122.00, 111.76, 81.78, 57.90, 50.66, 47.49, 37.98, 37.51, 35.47, 35.11, 31.95, 31.74, 30.48, 29.97, 27.26, 20.72, 19.40, 18.83, 18.81, 16.75, 11.44.

Minor product:

^{1}H NMR (500 MHz, $CDCl_3$) δ 8.62 (d, *J* = 2.2 Hz, 1H), 8.45 (dd, *J* = 4.8, 1.6 Hz, 1H), 7.65 (dd, *J* = 7.9, 2.0 Hz, 1H), 7.21 (dd, *J* = 7.9, 4.8 Hz, 1H), 5.99 (d, *J* = 1.5 Hz, 1H), 5.36 (d, *J* = 4.9 Hz, 1H), 2.38 – 2.20 (m, 4H), 2.05 (dddd, *J* = 13.5, 10.0, 6.2, 1.8 Hz, 3H), 1.85 (ddt, *J* = 27.3, 13.2, 3.2 Hz, 2H), 1.77 – 1.54 (m, 6H), 1.47 (td, *J* = 12.3, 4.7 Hz, 1H), 1.15 – 0.96 (m, 29H).

^{13}C NMR (126 MHz, $CDCl_3$) δ 151.83, 148.00, 147.90, 141.97, 133.88, 133.16, 129.43, 123.17, 120.45, 113.38, 79.59, 57.72, 50.62, 47.47, 39.53, 38.99, 37.09, 35.40, 32.34, 31.93, 31.60, 30.48, 29.70, 20.78, 19.47, 18.79, 16.72, 11.39.

HRMS (ESI-TOF) *m*/*z* calcd. For $C_{35}H_{52}NSi^+$ ($[M+H]^+$) 514.3864, found 514.3865.

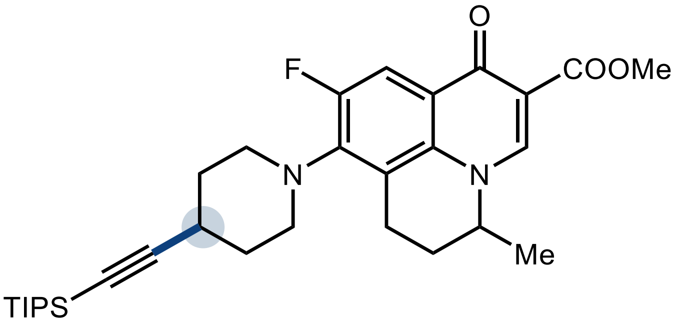

methyl 9-fluoro-5-methyl-1-oxo-8-(4-((triisopropylsilyl)ethynyl)piperidin-1-yl)-6,7-dihydro-1H,5H-pyrido[3,2,1-ij]quinoline-2-carboxylate (52): The title compound was prepared according to **General Procedure A** with methyl 9-fluoro-8-(4-hydroxypiperidin-1-yl)-5-methyl-1-oxo-6,7-dihydro-1H,5H-pyrido[3,2,1-ij]quinoline-2-carboxylate (alcohol) and (bromoethynyl)triisopropylsilane (alkynyl bromide). Purified by automated flash chromatography (25 g high performance silica column, 0–80% ethyl acetate/hexanes gradient) followed by preparative HPLC (XBridge BEH C18 OBD column, 30–80% MeCN/H_2O with 0.1% NH_4OH) to provide the title compound as a yellow oil. (63.6 mg, 24% yield).

^{1}H NMR (500 MHz, $CDCl_3$) δ 8.45 (s, 1H), 8.01 (d, J = 12.6 Hz, 1H), 4.39 (tt, J = 6.9, 3.3 Hz, 1H), 3.92 (s, 3H), 3.85 – 2.22 (m, 7H), 2.13 (dt, J = 9.4, 3.9 Hz, 2H), 2.09 – 1.86 (m, 2H), 1.80 (dt, J = 31.5, 7.3 Hz, 2H), 1.46 (d, J = 6.8 Hz, 3H), 1.09 (d, J = 5.1 Hz, 21H).

^{13}C NMR (126 MHz, $CDCl_3$) δ 173.55, 167.05, 157.30, 147.57, 142.14, 132.86, 127.02, 126.97, 125.44, 111.89, 111.70, 109.45, 57.13, 52.22, 26.29, 20.19, 18.79, 18.68, 11.40. (J_{CF} coupling constants remain unidentified in the data.)

^{19}F NMR (471 MHz, $CDCl_3$) δ -121.52, -122.07.

HRMS (ESI-TOF) *m/z* calcd. For $C_{31}H_{43}FN_2O_3SiNa^+$ ($[M+Na]^+$) 561.2919, found 561.2925.

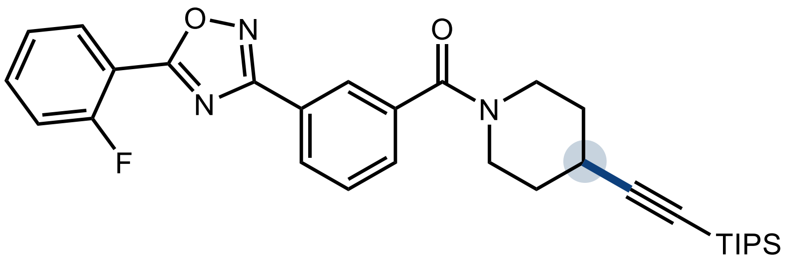

(3-(5-(2-fluorophenyl)-1,2,4-oxadiazol-3-yl)phenyl)(4-((triisopropylsilyl)ethynyl)piperidin-1-yl)methanone (53): The title compound was prepared according to **General Procedure A** with benzyl (3-(5-(2-fluorophenyl)-1,2,4-oxadiazol-3-yl)phenyl)(4-hydroxypiperidin-1-yl)methanone (alcohol) and (bromoethynyl)triisopropylsilane (alkynyl bromide). Purified by automated flash chromatography (25 g high performance silica column, 0–80% ethyl acetate/hexanes gradient) followed by preparative HPLC (XBridge BEH C18 OBD column, 30–80% MeCN/H_2O with 0.1% NH_4OH) to provide the title compound as a yellow oil. (168.7 mg, 63% yield).

^{1}H NMR (500 MHz, CDCl$_3$) δ 8.29 – 8.15 (m, 3H), 7.65 – 7.52 (m, 3H), 7.33 (td, J = 7.6, 1.0 Hz, 1H), 7.31 – 7.26 (m, 1H), 3.98 – 3.31 (m, 4H), 2.84 (tt, J = 6.6, 4.1 Hz, 1H), 2.00 – 1.56 (m, 4H), 1.16 – 0.93 (m, 21H).

^{13}C NMR (126 MHz, CDCl$_3$) δ 173.09, 173.05, 169.47, 168.25, 159.87, 137.10, 134.86, 134.79, 131.04, 129.77, 129.31, 128.66, 127.27, 126.13, 124.86, 124.83, 117.39, 117.22, 112.89, 112.80, 110.03, 82.63, 45.95, 40.33, 32.21, 31.35, 27.84, 18.76, 11.32. (J_{CF} coupling constants remain unidentified in the data.)

^{19}F NMR (471 MHz, CDCl$_3$) δ -108.18.

HRMS (ESI-TOF) *m/z* calcd. For $C_{31}H_{38}FN_3O_2SiNa^+$ ($[M+Na]^+$) 554.2610, found 554.2613.

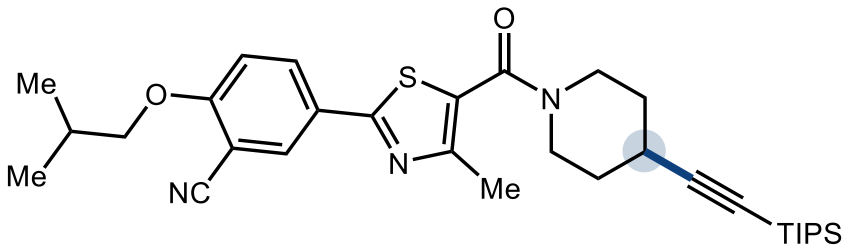

2-isobutoxy-5-(4-methyl-5-(4-((triisopropylsilyl)ethynyl)piperidine-1-carbonyl)thiazol-2-yl)benzonitrile (54): The title compound was prepared according to **General Procedure A** with benzyl 5-(5-(4-hydroxypiperidine-1-carbonyl)-4-methylthiazol-2-yl)-2-isobutoxybenzonitrile (alcohol) and (bromoethynyl)triisopropylsilane (alkynyl bromide). Purified by automated flash chromatography (25 g high performance silica column, 0–80% ethyl acetate/hexanes gradient) followed by preparative HPLC (XBridge BEH C18 OBD column, 30–80% MeCN/H_2O with 0.1% NH_4OH) to provide the title compound as a yellow oil. (183.3 mg, 65% yield).

^{1}H NMR (500 MHz, $CDCl_3$) δ 8.10 (d, *J* = 2.3 Hz, 1H), 8.02 (dd, *J* = 8.8, 2.3 Hz, 1H), 6.99 (d, *J* = 8.9 Hz, 1H), 3.88 (d, *J* = 6.5 Hz, 2H), 3.87 – 3.13 (m, 4H), 2.85 (tt, *J* = 6.4, 4.0 Hz, 1H), 2.46 (s, 3H), 2.19 (hept, *J* = 6.7 Hz, 1H), 1.94 – 1.79 (m, 2H), 1.72 (q, *J* = 5.9 Hz, 2H), 1.20 – 0.91 (m, 27H).

^{13}C NMR (126 MHz, $CDCl_3$) δ 164.93, 162.30, 162.24, 152.81, 132.42, 131.89, 126.24, 124.76, 115.60, 112.74, 109.65, 103.03, 82.96, 75.76, 43.13, 31.79, 28.28, 27.69, 19.17, 18.75, 16.55, 11.31.

HRMS (ESI-TOF) *m/z* calcd. For $C_{32}H_{46}N_3O_2SSi^+$ ($[M+H]^+$) 564.3075, found 564.3082.

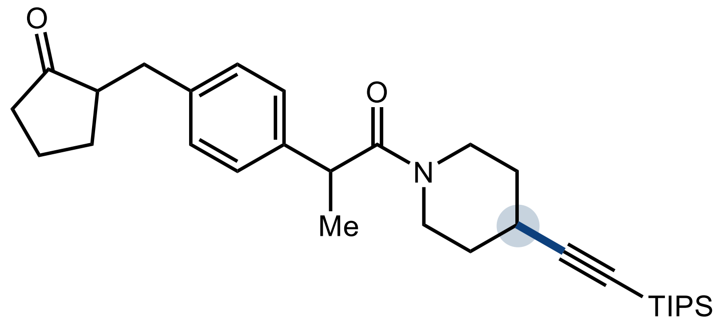

2-(4-(1-oxo-1-(4-((triisopropylsilyl)ethynyl)piperidin-1-yl)propan-2-yl)benzyl)cyclopentan-1-one (55): The title compound was prepared according to **General Procedure A** with 2-(4-(1-(4-hydroxypiperidin-1-yl)-1-oxopropan-2-yl)benzyl)cyclopentan-1-one (alcohol) and (bromoethynyl)triisopropylsilane (alkynyl bromide). Purified by automated flash chromatography (25 g high performance silica column, 0–80% ethyl acetate/hexanes gradient) followed by preparative HPLC (XBridge BEH C18 OBD column, 30–100% MeCN/H_2O with 0.1% NH_4OH) to provide the title compound as a yellow oil. (202.2 mg, 82% yield).

^{1}H NMR (500 MHz, $CDCl_3$) δ 7.24 – 6.97 (m, 4H), 3.94 – 3.76 (m, 2H), 3.60 – 3.39 (m, 2H), 3.38 – 3.22 (m, 1H), 3.14 – 3.04 (m, 1H), 2.63 (h, J = 4.8, 4.2 Hz, 1H), 2.53 – 2.43 (m, 1H), 2.37 – 2.24 (m, 2H), 2.14 – 2.00 (m, 2H), 1.97 – 1.89 (m, 1H), 1.81 – 1.67 (m, 1.5H), 1.64 – 1.47 (m, 3H), 1.41 (d, J = 6.8 Hz, 3.5H), 1.21 – 0.88 (m, 22H).

^{13}C NMR (126 MHz, $CDCl_3$) δ 172.16, 171.90, 140.29, 140.02, 138.49, 129.46, 129.42, 127.38, 127.36, 110.30, 82.47, 51.06, 51.03, 51.00, 43.90, 43.88, 43.34, 42.96, 42.91, 40.41, 40.37, 39.77, 38.29, 35.31, 35.28, 31.70, 31.42, 31.39, 31.06, 29.29, 29.27, 27.93, 27.43, 20.85, 20.83, 20.80, 20.65, 18.74, 18.68, 11.31, 11.25. (Mixture of rotamers.)

HRMS (ESI-TOF) *m/z* calcd. For $C_{31}H_{48}NO_2Si^+$ ($[M+H]^+$) 494.3449, found 494.3457.

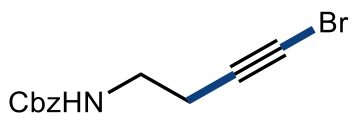

benzyl (4-bromobut-3-yn-1-yl)carbamate (56): The title compound was prepared according to **General Procedure C** followed by **General Procedure D** from **4**. Purified by automated flash chromatography (25 g high performance silica column, 0–10% ethyl acetate/hexanes gradient) to provide the title compound as a yellow oil. (two steps, 90% yield).

^{1}H NMR (500 MHz, $CDCl_3$) δ 7.42 – 7.28 (m, 5H), 5.11 (s, 2H), 5.04 (s, 1H), 3.35 (q, J = 6.4 Hz, 2H), 2.44 (t, J = 6.4 Hz, 2H).

^{13}C NMR (126 MHz, $CDCl_3$) δ 156.31, 136.51, 128.69, 128.32, 128.29, 77.48, 66.98, 40.12, 39.74, 21.27.

HRMS (ESI-TOF) *m/z* calcd. For $C_{12}H_{13}BrNO_2^+$ ($[M+H]^+$) 282.0124, found 282.0126.

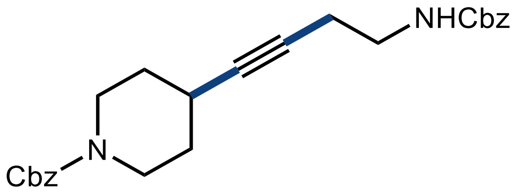

benzyl 4-(4-(((benzyloxy)carbonyl)amino)but-1-yn-1-yl)piperidine-1-carboxylate (57): The title compound was prepared according to **General Procedure B** with 4-hydroxypiperidine-1-carboxylate (alcohol) and **56** (alkynyl bromide). Purified by automated flash chromatography (25 g high performance silica column, 0–80% ethyl acetate/hexanes gradient) followed by preparative HPLC (XBridge BEH C18 OBD column, 30–60% MeCN/H_2O with 0.1% NH_4OH) to provide the title compound as a yellow oil. (108.6 mg, 52% yield, d.r. > 20:1).

^{1}H NMR (500 MHz, $CDCl_3$) δ 7.50 – 7.26 (m, 10H), 5.11 (d, *J* = 10.5 Hz, 4H), 5.01 (s, 1H), 3.84 – 3.65 (m, 2H), 3.37 – 3.11 (m, 4H), 2.63 – 2.50 (m, 1H), 2.38 (td, *J* = 6.6, 2.0 Hz, 2H), 1.75 (d, *J* = 13.6 Hz, 2H), 1.53 (q, *J* = 9.8, 9.2 Hz, 2H).

^{13}C NMR (126 MHz, $CDCl_3$) δ 156.32, 155.33, 136.98, 136.60, 128.66, 128.60, 128.29, 128.08, 127.97, 83.99, 78.59, 67.16, 66.87, 42.49, 40.30, 31.64, 27.01, 20.27.

HRMS (ESI-TOF) *m*/*z* calcd. For $C_{25}H_{28}N_2O_4Na^+$ ($[M+Na]^+$) 443.1941, found 443.1938.

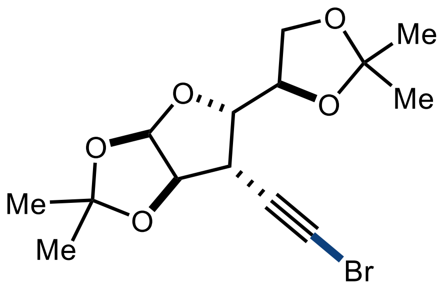

(3aR,5S,6S,6aR)-6-(bromoethynyl)-5-((R)-2,2-dimethyl-1,3-dioxolan-4-yl)-2,2-dimethyltetrahydrofuro[2,3-d][1,3]dioxole (58): The title compound was prepared according to **General Procedure C** followed by **General Procedure D** from **33**. Purified by automated flash chromatography (25 g high performance silica column, 0–10% ethyl acetate/hexanes gradient) to provide the title compound as a yellow oil. (two steps, 92% yield, d.r. >20:1). (relative configuration is determined by NOESY analysis)

^{1}H NMR (500 MHz, CDCl$_3$) δ 5.79 (d, J = 4.0 Hz, 1H), 4.75 (dd, J = 4.1, 3.1 Hz, 1H), 4.30 (td, J = 6.7, 5.7 Hz, 1H), 4.07 (dd, J = 8.4, 6.7 Hz, 1H), 3.92 – 3.87 (m, 2H), 2.97 (dd, J = 7.5, 3.1 Hz, 1H), 1.56 (s, 3H), 1.44 (s, 3H), 1.37 (d, J = 7.3 Hz, 6H).

^{13}C NMR (126 MHz, CDCl$_3$) δ 114.65, 110.22, 105.55, 86.76, 83.42, 75.88, 65.79, 43.77, 40.20, 27.94, 27.40, 26.50, 25.60.

HRMS (ESI-TOF) *m/z* calcd. For $C_{14}H_{19}BrO_5Na^+$ ([M+Na]$^+$) 369.0308, found 369.0310.

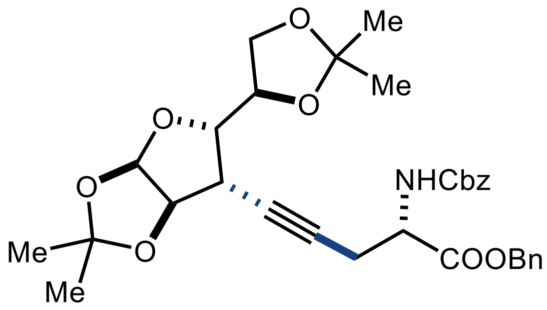

benzyl (S)-2-(((benzyloxy)carbonyl)amino)-5-((3aR,5S,6S,6aR)-5-((R)-2,2-dimethyl-1,3-dioxolan-4-yl)-2,2-dimethyltetrahydrofuro[2,3-d][1,3]dioxol-6-yl)pent-4-ynoate (58): The title compound was prepared according to **General Procedure B** with *Z*-*L*-serine benzyl ester (alcohol) and **58** (alkynyl bromide). Purified by automated flash chromatography (25 g high performance silica column, 0–80% ethyl acetate/hexanes gradient) followed by preparative HPLC (XBridge BEH C18 OBD column, 30–60% MeCN/H_2O with 0.1% NH_4OH) to provide the title compound as a yellow oil. (184.9 mg, 64% yield).

^{1}H NMR (500 MHz, $CDCl_3$) δ 7.46 – 7.28 (m, 10H), 5.70 (d, *J* = 4.0 Hz, 1H), 5.57 (d, *J* = 8.2 Hz, 1H), 5.26 – 5.14 (m, 2H), 5.12 (s, 2H), 4.63 – 4.39 (m, 2H), 4.22 (q, *J* = 6.5 Hz, 1H), 3.99 (dd, *J* = 8.4, 6.6 Hz, 1H), 3.83 (dd, *J* = 8.4, 6.8 Hz, 1H), 3.70 (dd, *J* = 7.5, 5.9 Hz, 1H), 2.79 (ddd, *J* = 27.0, 6.5, 2.6 Hz, 3H), 1.55 (s, 3H), 1.42 (s, 3H), 1.35 (s, 6H).

^{13}C NMR (126 MHz, $CDCl_3$) δ 170.34, 155.70, 136.18, 135.26, 128.78, 128.70, 128.42, 128.39, 128.33, 114.43, 110.09, 105.44, 87.18, 83.91, 80.97, 78.49, 76.04, 67.62, 67.32, 65.72, 52.80, 39.16, 27.95, 27.41, 26.49, 25.54, 23.34.

HRMS (ESI-TOF) *m*/*z* calcd. For $C_{32}H_{37}NO_9Na^+$ ($[M+Na]^+$) 602.2361, found 602.2362.

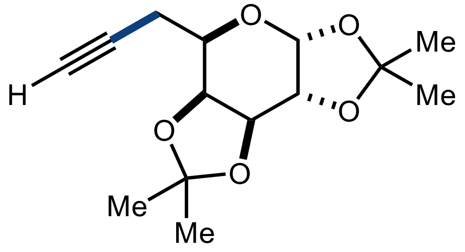

(3aR,5R,5aS,8aS,8bR)-2,2,7,7-tetramethyl-5-(prop-2-yn-1-yl)tetrahydro-5H-bis([1,3]dioxolo)[4,5-b:4',5'-d]pyran (60): The title compound was prepared by deprotection of a crude mixture of **35** according to **General Procedure C** in a 3 mmol scale (see large scale experiment for details). Purified by automated flash chromatography (25 g high performance silica column, 0–20% ethyl acetate/hexanes gradient) as a colorless oil. (547 mg, a total of 68% yield for two steps).

^{1}H NMR (500 MHz, $CDCl_3$) δ 5.51 (d, J = 5.0 Hz, 1H), 4.63 (dd, J = 7.9, 2.4 Hz, 1H), 4.36 – 4.29 (m, 2H), 3.92 (ddd, J = 8.4, 6.4, 1.9 Hz, 1H), 2.61 – 2.48 (m, 2H), 2.01 (t, J = 2.7 Hz, 1H), 1.55 (s, 3H), 1.45 (s, 3H), 1.36 (s, 3H), 1.33 (s, 3H).

^{13}C NMR (126 MHz, $CDCl_3$) δ 109.49, 108.80, 96.70, 80.43, 71.49, 70.95, 70.64, 69.98, 66.76, 26.20, 26.12, 25.05, 24.65, 20.47.

HRMS (ESI-TOF) *m*/*z* calcd. For $C_{14}H_{20}O_5Na^+$ ($[M+Na]^+$) 291.1203, found 291.1201.

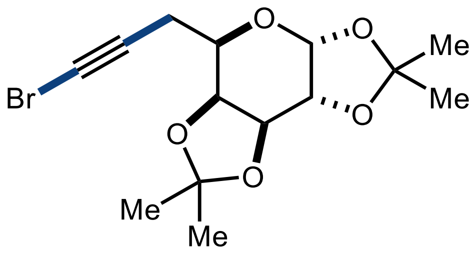

(3aR,5R,5aS,8aS,8bR)-5-(3-bromoprop-2-yn-1-yl)-2,2,7,7-tetramethyltetrahydro-5H-bis([1,3]dioxolo)[4,5-b:4',5'-d]pyran (61): The title compound was prepared according to **General Procedure D** from **60**. Purified by automated flash chromatography (25 g high performance silica column, 0–10% ethyl acetate/hexanes gradient) to provide the title compound as a yellow oil. (99% yield).

^{1}H NMR (500 MHz, CDCl$_3$) δ 5.50 (d, J = 5.0 Hz, 1H), 4.63 (dd, J = 7.9, 2.4 Hz, 1H), 4.31 (dt, J = 7.5, 2.1 Hz, 2H), 3.90 (ddd, J = 8.3, 5.9, 1.8 Hz, 1H), 2.62 (dd, J = 16.4, 8.9 Hz, 1H), 2.52 (dd, J = 16.4, 6.0 Hz, 1H), 1.55 (s, 3H), 1.45 (s, 3H), 1.37 (s, 3H), 1.33 (s, 3H).

^{13}C NMR (126 MHz, CDCl$_3$) δ 109.51, 108.84, 96.68, 71.43, 70.93, 70.65, 66.55, 39.85, 26.23, 26.11, 25.05, 24.65, 21.63.

HRMS (ESI-TOF) *m/z* calcd. For $C_{14}H_{19}BrO_5Na^+$ ($[M+Na]^+$) 369.0308, found 369.0307.

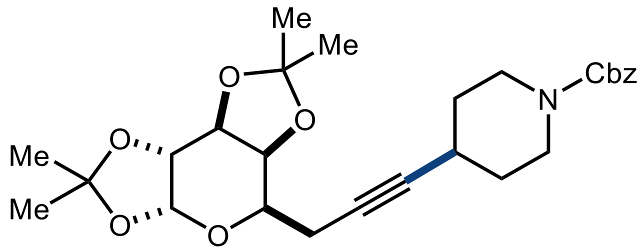

benzyl 4-(3-((3aR,5R,5aS,8aS,8bR)-2,2,7,7-tetramethyltetrahydro-5H-bis([1,3]dioxolo)[4,5-b:4',5'-d]pyran-5-yl)prop-1-yn-1-yl)piperidine-1-carboxylate (62): The title compound was prepared according to **General Procedure B** with benzyl 4-hydroxypiperidine-1-carboxylate (alcohol) and **61** (alkynyl bromide). Purified by automated flash chromatography (25 g high performance silica column, 0–80% ethyl acetate/hexanes gradient) followed by preparative HPLC (XBridge BEH C18 OBD column, 30–60% MeCN/H_2O with 0.1% NH_4OH) to provide the title compound as a yellow oil. (163.6 mg, 67% yield).

^{1}H NMR (500 MHz, $CDCl_3$) δ 7.42 – 7.27 (m, 5H), 5.50 (d, *J* = 5.1 Hz, 1H), 5.12 (s, 2H), 4.60 (dd, *J* = 8.0, 2.4 Hz, 1H), 4.32 – 4.25 (m, 2H), 3.88 – 3.81 (m, 1H), 3.78 – 3.60 (m, 2H), 3.42 – 3.22 (m, 2H), 2.64 – 2.56 (m, 1H), 2.56 – 2.43 (m, 2H), 1.74 (d, *J* = 8.3 Hz, 2H), 1.65 – 1.54 (m, 2H), 1.52 (s, 3H), 1.44 (s, 3H), 1.33 (d, *J* = 10.5 Hz, 6H).

^{13}C NMR (126 MHz, $CDCl_3$) δ 155.34, 137.02, 128.57, 128.04, 127.93, 109.32, 108.65, 96.65, 83.44, 78.01, 71.63, 70.92, 70.68, 67.24, 67.08, 42.29, 31.56, 26.88, 26.18, 26.07, 25.03, 24.51, 20.77.

HRMS (ESI-TOF) *m/z* calcd. For $C_{27}H_{35}NO_7Na^+$ ($[M+Na]^+$) 508.2306, found 508.2301.

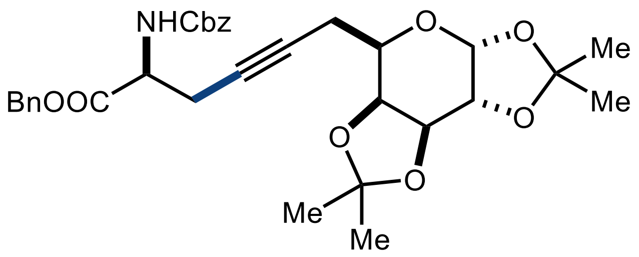

benzyl (S)-2-(((benzyloxy)carbonyl)amino)-6-((3aR,5R,5aS,8aS,8bR)-2,2,7,7-tetramethyltetrahydro-5H-bis([1,3]dioxolo)[4,5-b:4',5'-d]pyran-5-yl)hex-4-ynoate (63): The title compound was prepared according to **General Procedure B** with *Z*-*L*-serine benzyl ester (alcohol) and **61** (alkynyl bromide). Purified by automated flash chromatography (25 g high performance silica column, 0–80% ethyl acetate/hexanes gradient) followed by preparative HPLC (XBridge BEH C18 OBD column, 30–60% MeCN/H_2O with 0.1% NH_4OH) to provide the title compound as a yellow oil. (184.1 mg, 64% yield).

^{1}H NMR (500 MHz, $CDCl_3$) δ 7.56 – 7.26 (m, 10H), 5.75 (d, *J* = 8.7 Hz, 1H), 5.48 (d, *J* = 5.0 Hz, 1H), 5.26 – 5.08 (m, 4H), 4.63 – 4.39 (m, 2H), 4.27 (td, *J* = 8.0, 7.3, 2.2 Hz, 2H), 3.86 – 3.75 (m, 1H), 2.86 – 2.65 (m, 2H), 2.52 – 2.37 (m, 2H), 1.50 (s, 3H), 1.40 (s, 3H), 1.30 (d, *J* = 10.7 Hz, 6H).

^{13}C NMR (126 MHz, $CDCl_3$) δ 170.63, 155.91, 136.28, 135.42, 128.73, 128.64, 128.54, 128.35, 128.33, 128.25, 109.39, 108.68, 96.65, 80.22, 75.69, 71.45, 70.91, 70.56, 67.45, 67.23, 66.75, 52.94, 26.14, 26.04, 24.99, 24.50, 23.29, 20.68.

HRMS (ESI-TOF) *m*/*z* calcd. For $C_{32}H_{38}NO_9^+$ ($[M+H]^+$) 580.2541, found 580.2553.

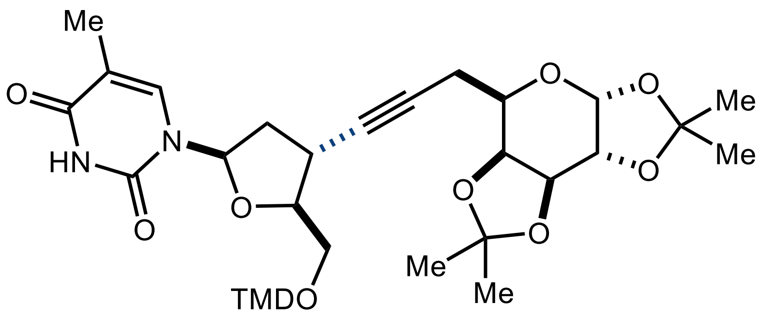

1-((2R,4R,5S)-5-((bis(4-methoxyphenyl)(phenyl)methoxy)methyl)-4-(3-((3aR,5R,5aS,8aS,8bR)-2,2,7,7-tetramethyltetrahydro-5H-bis([1,3]dioxolo)[4,5-b:4',5'-d]pyran-5-yl)prop-1-yn-1-yl)tetrahydrofuran-2-yl)-5-methylpyrimidine-2,4(1H,3H)-dione (64): The title compound was prepared according to **General Procedure B** with 5'-O-(4,4'-Dimethoxytrityl)thymidine (alcohol) and **61** (alkynyl bromide). Purified by automated flash chromatography (25 g high performance silica column, 0–80% ethyl acetate/hexanes gradient) followed by preparative HPLC (XBridge BEH C18 OBD column, 30–60% MeCN/H_2O with 0.1% NH_4OH) to provide the title compound as a yellow oil. (205.6 mg, 52% yield, d.r. > 20:1).

^{1}H NMR (500 MHz, Acetone) δ 10.00 (s, 1H), 7.63 (d, J = 1.5 Hz, 1H), 7.52 (dd, J = 8.5, 1.3 Hz, 2H), 7.39 (d, J = 8.9 Hz, 4H), 7.32 (dd, J = 8.6, 7.0 Hz, 2H), 7.27 – 7.22 (m, 1H), 6.90 (dd, J = 9.0, 1.5 Hz, 4H), 6.15 (dd, J = 7.3, 3.2 Hz, 1H), 5.44 (d, J = 5.0 Hz, 1H), 4.62 (dd, J = 7.9, 2.4 Hz, 1H), 4.33 (dd, J = 5.0, 2.4 Hz, 1H), 4.28 (dd, J = 7.9, 1.8 Hz, 1H), 4.04 (dt, J = 9.3, 3.1 Hz, 1H), 3.84 (ddd, J = 8.1, 6.6, 1.9 Hz, 1H), 3.79 (s, 6H), 3.50 – 3.38 (m, 3H), 2.57 – 2.42 (m, 3H), 2.41 – 2.34 (m, 1H), 1.56 (d, J = 1.3 Hz, 3H), 1.41 (s, 3H), 1.37 (s, 3H), 1.30 (d, J = 10.0 Hz, 6H).

^{13}C NMR (126 MHz, Acetone) δ 164.39, 159.68, 151.22, 145.98, 136.75, 136.60, 136.58, 131.00, 130.97, 129.00, 128.71, 127.66, 114.02, 114.00, 110.51, 109.60, 109.00, 97.33, 87.21, 85.60, 85.57, 80.10, 79.88, 72.28, 71.72, 71.35, 67.79, 63.25, 55.54, 40.05, 30.66, 26.39, 26.35, 25.13, 24.67, 21.26, 12.45.

HRMS (ESI-TOF) *m*/*z* calcd. For $C_{45}H_{50}N_2O_{11}Na^+$ ($[M+Na]^+$) 817.3307, found 817.3310.

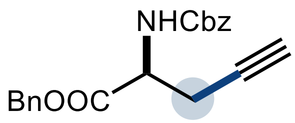

benzyl (S)-2-(((benzyloxy)carbonyl)amino)pent-4-ynoate (65): The title compound was prepared from **37** according to **General Procedure C** with yield of 98%, total yield of 80% from *Z*-*L*-serine benzyl ester. Purified by automated flash chromatography (25 g high performance silica column, 20–50% ethyl acetate/hexanes gradient) to provide the title compound as a yellow oil.

^{1}H NMR (500 MHz, $CDCl_3$) δ 7.48 – 7.27 (m, 10H), 5.64 (d, *J* = 8.5 Hz, 1H), 5.22 (d, *J* = 4.3 Hz, 2H), 5.13 (d, *J* = 1.4 Hz, 2H), 4.59 (dt, *J* = 9.1, 4.8 Hz, 1H), 2.87 – 2.63 (m, 2H), 2.00 (s, 1H).

^{13}C NMR (126 MHz, $CDCl_3$) δ 170.30, 155.78, 136.24, 135.22, 128.76, 128.71, 128.68, 128.47, 128.40, 128.29, 78.32, 72.09, 67.76, 67.32, 52.57, 22.97.

HRMS (ESI-TOF) *m*/*z* calcd. For $C_{20}H_{19}NO_4Na^+$ ($[M+Na]^+$) 360.1206, found 360.1211.

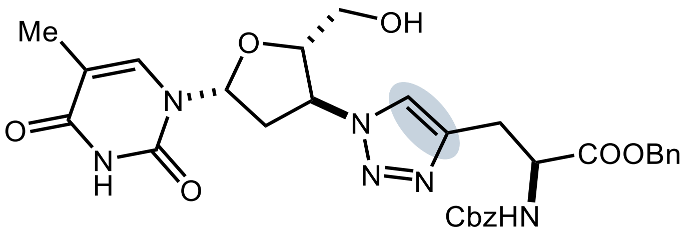

benzyl (S)-2-(((benzyloxy)carbonyl)amino)-3-(1-((2S,3S,5R)-2-(hydroxymethyl)-5-(5-methyl-2,4-dioxo-3,4-dihydropyrimidin-1(2H)-yl)tetrahydrofuran-3-yl)-1H-1,2,3-triazol-4-yl)propanoate (66): The title compound was prepared according to **General Procedure F** with **65** (alkyne) and Zidovudine (azide) in a 0.1 mmol scale. Purified by preparative HPLC (XBridge BEH C18 OBD column, 30–60% MeCN/H_2O with 0.1% NH_4OH) to provide the title compound as a yellow solid. (53.8 mg, 89% yield).

^{1}H NMR (500 MHz, CD_3CN) δ 9.04 (s, 1H), 7.61 (q, *J* = 1.3 Hz, 1H), 7.54 (d, *J* = 10.9 Hz, 1H), 7.38 – 7.26 (m, 9H), 6.35 (t, *J* = 6.5 Hz, 1H), 6.15 (d, *J* = 8.2 Hz, 1H), 5.20 (dt, *J* = 8.5, 5.4 Hz, 1H), 5.10 (d, *J* = 2.1 Hz, 2H), 5.03 (d, *J* = 3.5 Hz, 2H), 4.55 (q, *J* = 7.1 Hz, 1H), 4.19 (dq, *J* = 5.5, 2.8 Hz, 1H), 3.81 – 3.73 (m, 1H), 3.63 (dd, *J* = 12.3, 3.2 Hz, 1H), 3.16 (qd, *J* = 15.1, 6.3 Hz, 2H), 2.74 – 2.64 (m, 1H), 2.62 – 2.53 (m, 1H), 1.92 (p, *J* = 2.5 Hz, 2H), 1.83 (d, *J* = 1.3 Hz, 3H).

^{13}C NMR (126 MHz, CD_3CN) δ 172.13, 164.57, 156.95, 151.45, 143.87, 137.99, 137.31, 136.93, 129.50, 129.44, 129.21, 129.10, 129.07, 128.93, 128.71, 128.69, 123.57, 111.25, 86.20, 86.16, 85.66, 85.62, 67.71, 67.21, 61.94, 60.24, 54.86, 38.45, 28.47, 12.55.

HRMS (ESI-TOF) *m*/*z* calcd. For $C_{30}H_{33}N_6O_8^+$ ($[M+H]^+$) 605.2354, found 605.2354.

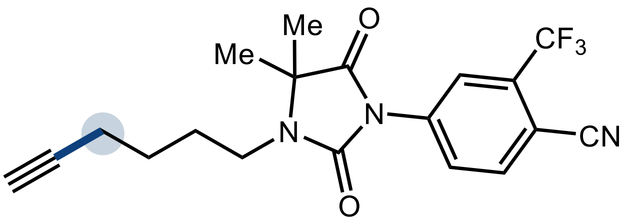

4-(3-(hex-5-yn-1-yl)-4,4-dimethyl-2,5-dioxoimidazolidin-1-yl)-2-(trifluoromethyl)benzonitrile (67): The title compound was prepared from **50** according to **General Procedure C** with yield of 90%, total yield of 62% from RU-58841. Purified by automated flash chromatography (25 g high performance silica column, 20–50% ethyl acetate/hexanes gradient) to provide the title compound as a yellow oil.

^{1}H NMR (500 MHz, CDCl$_3$) δ 8.16 (d, J = 2.0 Hz, 1H), 8.01 (dd, J = 8.4, 2.1 Hz, 1H), 7.91 (d, J = 8.4 Hz, 1H), 3.43 – 3.35 (m, 2H), 2.28 (td, J = 6.9, 2.6 Hz, 2H), 1.98 (t, J = 2.6 Hz, 1H), 1.89 – 1.81 (m, 2H), 1.65 – 1.59 (m, 2H), 1.55 (s, 6H).

^{13}C NMR (126 MHz, CDCl$_3$) δ 174.63, 152.83, 136.50, 135.26, 133.63 (q, J_{CF} = 33.1 Hz), 127.86, 122.98 (q, J_{CF} = 4.8 Hz), 121.97 (q, J_{CF} = 274.3 Hz), 115.03, 108.25 (q, J_{CF} = 2.2 Hz), 83.52, 69.07, 61.87, 39.87, 28.47, 25.63, 23.54, 18.01.

^{19}F NMR (471 MHz, CDCl$_3$) δ -62.03.

HRMS (ESI-TOF) *m/z* calcd. For $C_{19}H_{19}F_3N_3O_2^+$ ([M+H]$^+$) 378.1424, found 378.1421.

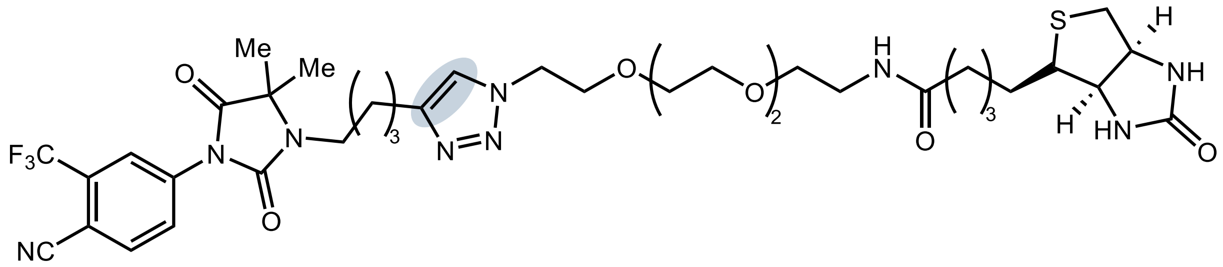

N-(2-(2-(2-(2-(4-(4-(3-(4-cyano-3-(trifluoromethyl)phenyl)-5,5-dimethyl-2,4-dioxoimidazolidin-1-yl)butyl)-1H-1,2,3-triazol-1-yl)ethoxy)ethoxy)ethoxy)ethyl)-5-((3aR,4S,6aS)-2-oxohexahydro-1H-thieno[3,4-d]imidazol-4-yl)pentanamide (68): The title compound was prepared according to **General Procedure F** with **67** (alkyne) and Biotin-PEG3-azide (azide) in a 0.1 mmol scale. Purified by preparative HPLC (XBridge BEH C18 OBD column, 30–60% MeCN/H_2O with 0.1% NH_4OH) to provide the title compound as a yellow solid. (76.0 mg, 92% yield).

^{1}H NMR (500 MHz, $CDCl_3$) δ 8.13 (d, J = 2.0 Hz, 1H), 7.99 (dd, J = 8.4, 2.1 Hz, 1H), 7.90 (d, J = 8.4 Hz, 1H), 7.50 (s, 1H), 6.72 (t, J = 5.6 Hz, 1H), 6.55 (s, 1H), 5.67 (s, 1H), 4.48 (dt, J = 15.7, 5.0 Hz, 3H), 4.28 (dd, J = 8.0, 4.5 Hz, 1H), 3.86 (t, J = 5.1 Hz, 2H), 3.65 – 3.50 (m, 11H), 3.44 – 3.35 (m, 4H), 3.12 (dt, J = 12.0, 5.4 Hz, 1H), 2.87 (dd, J = 12.8, 4.8 Hz, 1H), 2.74 (q, J = 13.0, 10.0 Hz, 3H), 2.34 (s, 1H), 2.18 (t, J = 7.5 Hz, 2H), 1.76 (d, J = 5.5 Hz, 4H), 1.66 – 1.60 (m, 2H), 1.50 (s, 6H), 1.40 (p, J = 6.9 Hz, 2H).

^{13}C NMR (126 MHz, $CDCl_3$) δ 174.69, 173.34, 164.07, 152.81, 136.55, 135.29, 133.47 (q, J_{CF} = 33.3 Hz), 127.99, 123.01 (q, J_{CF} = 4.8 Hz), 122.13, 122.00 (q, J_{CF} = 274.3 Hz), 115.07, 108.11 (d, J_{CF} = 2.4 Hz), 70.47, 70.36, 70.34, 70.04, 69.95, 69.54, 61.93, 61.79, 60.19, 55.70, 50.14, 40.57, 40.09, 39.12, 35.92, 28.93, 28.23, 28.09, 26.87, 25.59, 25.08, 23.47.

^{19}F NMR (471 MHz, $CDCl_3$) δ -61.97.

HRMS (ESI-TOF) *m/z* calcd. For $C_{37}H_{50}F_3N_9O_7SNa^+$ ($[M+Na]^+$) 844.3398, found 844.3397.

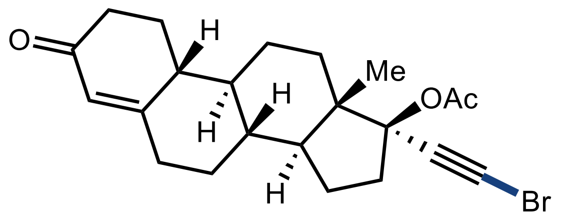

(8R,9S,10R,13S,14S,17S)-17-(bromoethynyl)-13-methyl-3-oxo-2,3,6,7,8,9,10,11,12,13,14,15,16,17-tetradecahydro-1H-cyclopenta[a]phenanthren-17-yl acetate (69): The title compound was prepared according to **General Procedure D** from *Norethindrone Acetate*. Purified by automated flash chromatography (25 g high performance silica column, 0–10% ethyl acetate/hexanes gradient) to provide the title compound as a yellow solid. (85% yield).

^{1}H NMR (500 MHz, CDCl$_3$) δ 5.84 (s, 1H), 2.73 (ddd, *J* = 14.7, 9.8, 5.9 Hz, 1H), 2.49 (dt, *J* = 14.9, 3.5 Hz, 1H), 2.43 (dt, *J* = 16.0, 4.6 Hz, 1H), 2.33 – 2.23 (m, 3H), 2.12 – 2.06 (m, 1H), 2.05 – 1.98 (m, 4H), 1.93 (dt, *J* = 13.7, 3.9 Hz, 1H), 1.84 (ddd, *J* = 10.1, 5.5, 2.8 Hz, 1H), 1.80 – 1.71 (m, 3H), 1.65 – 1.57 (m, 1H), 1.50 (td, *J* = 11.6, 7.3 Hz, 1H), 1.39 – 1.24 (m, 3H), 1.14 (td, *J* = 12.6, 12.1, 4.1 Hz, 1H), 0.91 (s, 4H).

^{13}C NMR (126 MHz, CDCl$_3$) δ 199.97, 169.54, 166.37, 124.84, 85.33, 79.58, 49.04, 48.22, 47.89, 46.83, 42.68, 40.91, 37.19, 36.67, 35.57, 33.19, 30.79, 26.70, 26.29, 23.56, 21.53, 13.59.

HRMS (ESI-TOF) *m/z* calcd. For $C_{22}H_{28}BrO_3^+$ ([M+H]$^+$) 419.1216, found 419.1207.

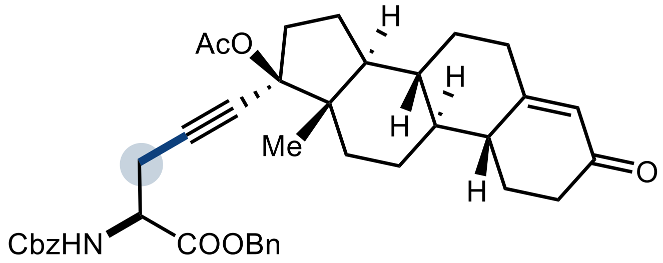

benzyl (S)-5-((8R,9S,10R,13S,14S,17S)-17-acetoxy-13-methyl-3-oxo-2,3,6,7,8,9,10,11,12,13,14,15,16,17-tetradecahydro-1H-cyclopenta[a]phenanthren-17-yl)-2-(((benzyloxy)carbonyl)amino)pent-4-ynoate (70): The title compound was prepared according to **General Procedure B** in 0.2 mmol scale with with *Z*-*L*-serine benzyl ester (alcohol) and **69** (alkynyl bromide). Purified by automated flash chromatography (25 g high performance silica column, 0–80% ethyl acetate/hexanes gradient) followed by preparative HPLC (XBridge BEH C18 OBD column, 30–60% MeCN/H_2O with 0.1% NH_4OH) to provide the title compound as a yellow solid. (85.0 mg, 65% yield).

^{1}H NMR (500 MHz, $CDCl_3$) δ 7.51 – 7.26 (m, 10H), 5.97 – 5.65 (m, 2H), 5.23 (d, *J* = 12.5 Hz, 1H), 5.17 – 5.04 (m, 3H), 4.59 (dt, *J* = 8.8, 4.5 Hz, 1H), 2.91 – 2.76 (m, 2H), 2.67 – 2.59 (m, 1H), 2.50 – 2.42 (m, 1H), 2.37 (dt, *J* = 17.6, 4.7 Hz, 1H), 2.30 – 2.11 (m, 3H), 2.08 – 1.98 (m, 2H), 1.97 (s, 3H), 1.87 – 1.76 (m, 2H), 1.73 – 1.61 (m, 3H), 1.54 – 1.37 (m, 2H), 1.38 – 1.27 (m, 2H), 1.25 – 1.17 (m, 1H), 1.10 – 1.00 (m, 1H), 0.88 (s, 3H), 0.87 – 0.80 (m, 1H).

^{13}C NMR (126 MHz, $CDCl_3$) δ 199.90, 170.40, 170.04, 166.38, 156.00, 136.55, 135.40, 128.78, 128.61, 128.54, 128.19, 127.99, 127.96, 124.80, 84.75, 83.70, 81.77, 67.38, 67.02, 52.92, 49.08,

47.81, 47.60, 42.62, 40.85, 37.44, 36.64, 35.54, 33.15, 30.75, 26.63, 26.28, 23.41, 23.34, 21.57, 13.47.

HRMS (ESI-TOF) *m*/*z* calcd. For $C_{40}H_{45}NO_7Na^+$ ($[M+Na]^+$) 674.3088, found 674.3088.

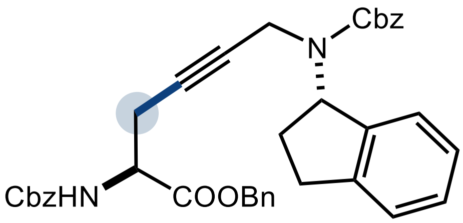

benzyl (S)-6-(((benzyloxy)carbonyl)((R)-2,3-dihydro-1H-inden-1-yl)amino)-2-(((benzyloxy)carbonyl)amino)hex-4-ynoate (71): The title compound was prepared according to **General Procedure B** in 0.2 mmol scale with with *Z-L*-serine benzyl ester (alcohol) and benzyl (S)-(3-bromoprop-2-yn-1-yl)(2,3-dihydro-1H-inden-1-yl)carbamate (alkynyl bromide). Purified by automated flash chromatography (25 g high performance silica column, 0–80% ethyl acetate/hexanes gradient) followed by preparative HPLC (XBridge BEH C18 OBD column, 30–60% MeCN/H_2O with 0.1% NH_4OH) to provide the title compound as a yellow solid. (78.4 mg, 64% yield).

^{1}H NMR (500 MHz, $CDCl_3$) δ 7.49 – 7.27 (m, 14H), 7.27 – 7.08 (m, 5H), 5.95 – 5.67 (m, 1H), 5.67 – 5.35 (m, 1H), 5.26 – 5.08 (m, 6H), 4.52 (s, 1H), 3.95 (dd, *J* = 54.9, 17.9 Hz, 1H), 3.48 (dd, *J* = 63.3, 17.9 Hz, 1H), 2.97 (d, *J* = 14.1 Hz, 1H), 2.88 – 2.66 (m, 3H), 2.38 (s, 1H), 2.13 (t, *J* = 11.2 Hz, 1H).

^{13}C NMR (126 MHz, $CDCl_3$) δ 170.41, 156.16, 155.80, 143.98, 143.50, 141.18, 141.17, 136.85, 136.27, 136.25, 135.28, 128.71, 128.66, 128.58, 128.32, 128.14, 127.77, 126.77, 125.14, 124.48, 67.51, 67.25, 62.01, 61.99, 52.72, 34.09, 32.89, 30.32, 30.01, 23.13.

HRMS (ESI-TOF) *m/z* calcd. For $C_{38}H_{36}N_2O_6Na^+$ ($[M+Na]^+$) 639.2466, found 639.2469.

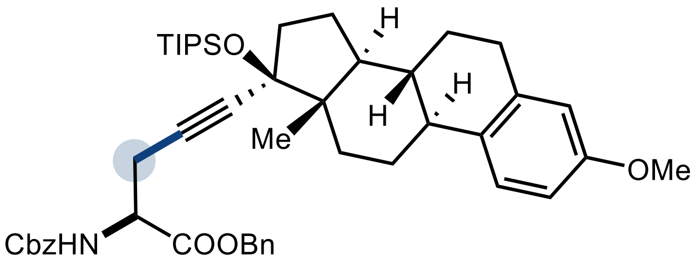

benzyl (S)-2-(((benzyloxy)carbonyl)amino)-5-((8R,9S,13S,14S,17S)-3-methoxy-13-methyl-17-((triisopropylsilyl)oxy)-7,8,9,11,12,13,14,15,16,17-decahydro-6H-cyclopenta[a]phenanthren-17-yl)pent-4-ynoate (72): The title compound was prepared according to **General Procedure B** with *Z-L*-serine benzyl ester (alcohol) and (((8R,9S,13S,14S,17S)-17-(bromoethynyl)-3-methoxy-13-methyl-7,8,9,11,12,13,14,15,16,17-decahydro-6H-cyclopenta[a]phenanthren-17-yl)oxy)triisopropylsilane (alkynyl bromide, prepared according to known procedure[4]). Purified by automated flash chromatography (25 g high performance silica column, 0–80% ethyl acetate/hexanes gradient) followed by preparative HPLC (XBridge BEH C18 OBD column, 30–60% $MeCN/H_2O$ with 0.1% NH_4OH) to provide the title compound as a yellow oil. (244.6 mg, 63% yield).

^{1}H NMR (500 MHz, $CDCl_3$) δ 7.43 – 7.26 (m, 10H), 7.16 (d, J = 8.6 Hz, 1H), 6.72 (dd, J = 8.6, 2.8 Hz, 1H), 6.63 (d, J = 2.7 Hz, 1H), 5.58 (d, J = 8.0 Hz, 1H), 5.19 (d, J = 2.3 Hz, 2H), 5.14 – 5.01 (m, 2H), 4.55 (dt, J = 8.5, 4.5 Hz, 1H), 3.79 (s, 3H), 2.97 – 2.65 (m, 4H), 2.39 – 2.25 (m, 1H), 2.25 – 2.13 (m, 2H), 1.96 (td, J = 12.5, 3.9 Hz, 1H), 1.88 – 1.76 (m, 2H), 1.75 – 1.57 (m, 3H), 1.50 – 1.25 (m, 4H), 1.19 – 1.12 (m, 3H), 1.08 (dd, J = 12.7, 7.0 Hz, 18H), 0.85 (s, 3H).

^{13}C NMR (126 MHz, $CDCl_3$) δ 170.43, 157.51, 155.68, 138.13, 136.20, 135.19, 133.01, 128.78, 128.63, 128.61, 128.27, 128.20, 128.14, 126.56, 113.89, 111.54, 88.48, 80.76, 67.59, 67.23,

55.34, 52.89, 48.92, 48.69, 43.74, 41.06, 39.63, 33.36, 29.97, 27.39, 26.67, 23.30, 23.09, 18.51, 18.47, 13.45, 13.12.

HRMS (ESI-TOF) *m*/*z* calcd. For $C_{48}H_{63}NO_6SiNa^+$ ($[M+Na]^+$) 800.4317, found 800.4327.

Additional Previously Unreported Compounds

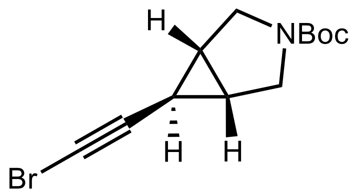

tert-butyl (1R,5S,6s)-6-(bromoethynyl)-3-azabicyclo[3.1.0]hexane-3-carboxylate: The title compound was prepared according to **General Procedure D** from commercially available starting material tert-butyl (1R,5S,6S)-6-ethynyl-3-azabicyclo[3.1.0]hexane-3-carboxylate (CAS: 414574-91-5). Purified by automated flash chromatography (25 g high performance silica column, 0–20% ethyl acetate/hexanes gradient) to provide the title compound as a yellow solid. (99% yield).

^{1}H NMR (500 MHz, CDCl$_3$) δ 3.69 – 3.52 (m, 2H), 3.34 (d, J = 11.1 Hz, 2H), 1.85 – 1.78 (m, 2H), 1.43 (s, 9H), 1.11 (t, J = 3.4 Hz, 1H).

^{13}C NMR (126 MHz, CDCl$_3$) δ 154.88, 80.04, 79.86, 47.90, 47.66, 36.26, 28.58, 26.18, 25.45, 12.11.

HRMS (ESI-TOF) *m*/*z* calcd. For $C_8H_9BrNO_2^+$ ([M-C_4H_8+H]$^+$) 229.9811, found 229.2810.

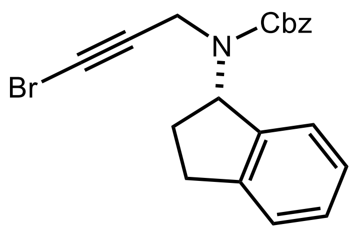

benzyl (S)-(3-bromoprop-2-yn-1-yl)(2,3-dihydro-1H-inden-1-yl)carbamate: The title compound was prepared according to **General Procedure D** from *Cbz*-protected commercially available Rasagiline (*Cbz*-protection procedure can be found)[5]. Purified by automated flash chromatography (25 g high performance silica column, 0–20% ethyl acetate/hexanes gradient) to provide the title compound as a colorless oil. (75% yield).

^{1}H NMR (500 MHz, $CDCl_3$) δ 7.65 – 7.06 (m, 9H), 6.15 – 5.56 (m, 1H), 5.42 – 5.08 (m, 2H), 4.13 (dd, *J* = 89.2, 17.8 Hz, 1H), 3.62 (dd, *J* = 57.8, 17.9 Hz, 1H), 3.06 (d, *J* = 11.2 Hz, 1H), 2.89 (d, *J* = 7.0 Hz, 1H), 2.50 (d, *J* = 10.1 Hz, 1H), 2.33 – 2.05 (m, 1H).

^{13}C NMR (126 MHz, $CDCl_3$) δ 156.11, 144.03, 143.61, 140.85, 140.69, 136.68, 136.48, 128.59, 128.21, 128.06, 127.94, 127.82, 126.79, 125.15, 124.56, 124.37, 67.60, 67.59, 62.16, 61.98, 42.01, 34.81, 33.74, 30.35, 30.04.

HRMS (ESI-TOF) *m/z* calcd. For $C_{20}H_{19}BrNO_2^+$ ($[M+H]^+$) 384.0594, found 384.0587.

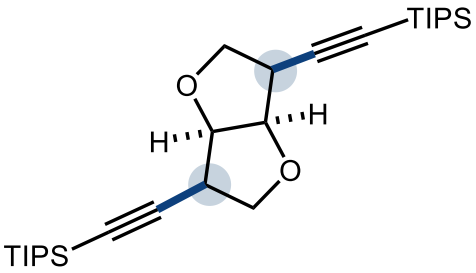

(3R,3aR,6R,6aR)-3,6-bis((triisopropylsilyl)ethynyl)hexahydrofuro[3,2-b]furan: The title compound was prepared according to **General Procedure A** with Isosorbide (alcohol) and (bromoethynyl)triisopropylsilane (alkynyl bromide). Purified by automated flash chromatography (25 g high performance silica column, 0–80% ethyl acetate/hexanes gradient) followed by preparative HPLC (XBridge BEH C18 OBD column, 30–100% MeCN/H_2O with 0.1% NH_4OH) to provide the title compound as a yellow oil. (61.8 mg, 26% yield, d.r. > 20:1). (relative configuration is determined by NOESY analysis)

^{1}H NMR (500 MHz, $CDCl_3$) δ 4.74 (s, 2H), 4.00 (dd, J = 8.3, 6.0 Hz, 2H), 3.89 (dd, J = 8.3, 3.4 Hz, 2H), 3.09 (dd, J = 5.9, 3.5 Hz, 2H), 1.15 – 0.96 (m, J = 4.1 Hz, 42H).

^{13}C NMR (126 MHz, $CDCl_3$) δ 106.41, 89.07, 84.12, 73.86, 39.59, 18.71, 11.28.

HRMS (ESI-TOF) *m/z* calcd. For $C_{28}H_{51}O_2Si_2^+$ ($[M+H]^+$) 475.3422, found 475.3420.

10) Spectral data

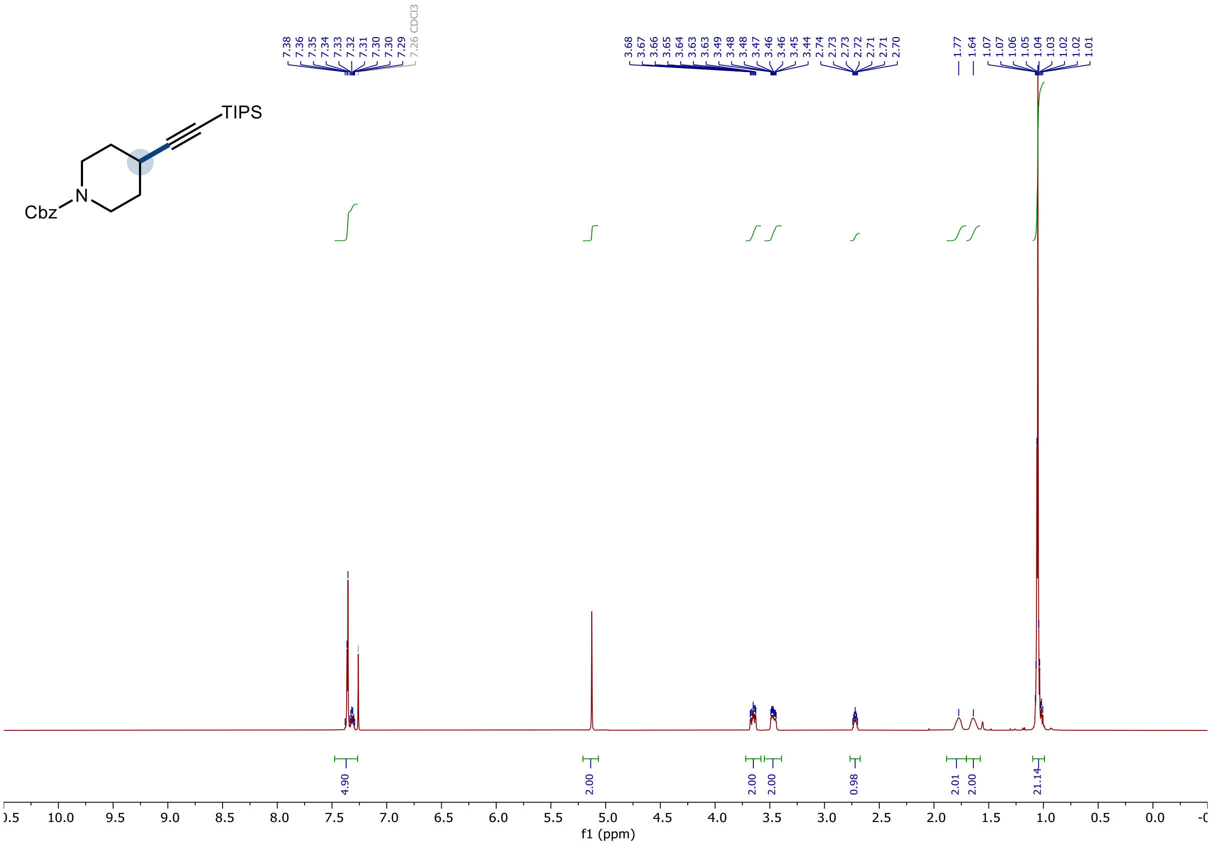

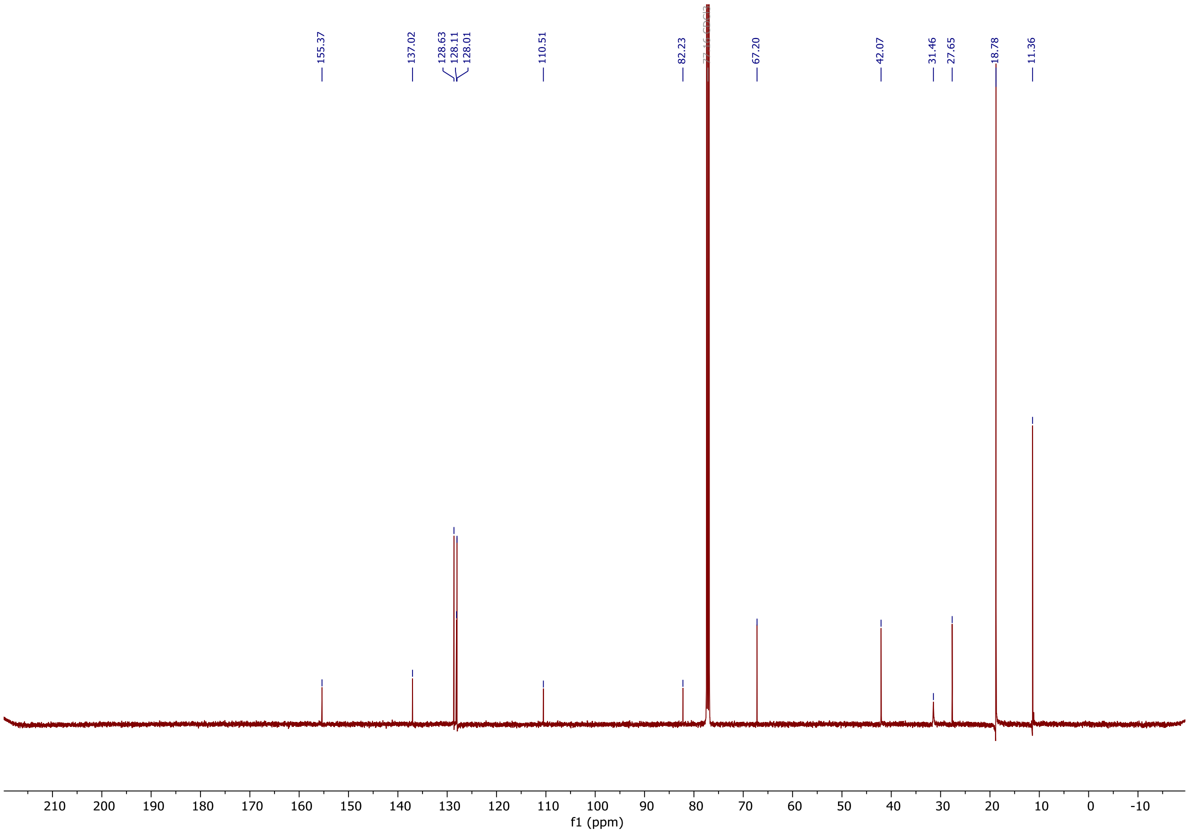

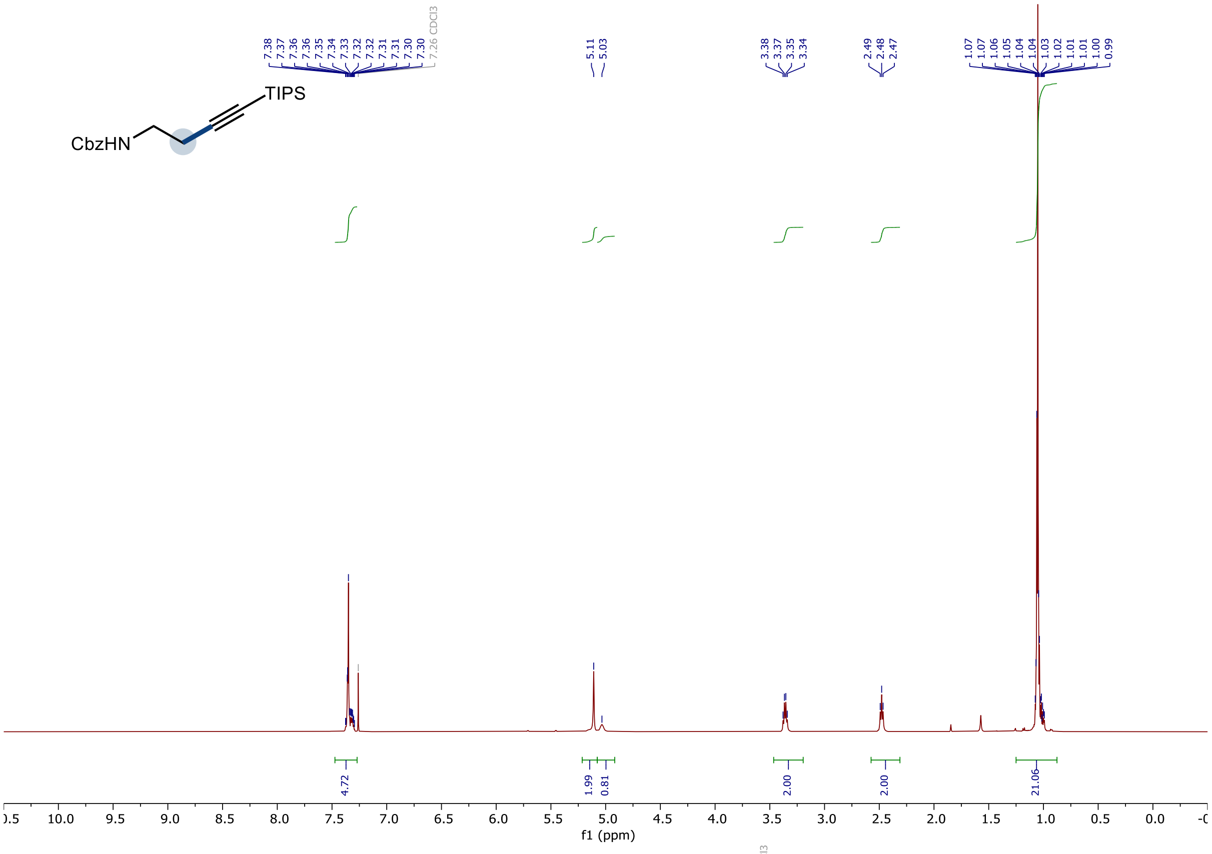
TIPS
CbzHN
7.38
7.37
7.36
7.36
7.35
7.34
7.33
7.32
7.32
7.31
7.31
7.30
7.30
7.26 CDCl3
5.11
5.03
3.38
3.37
3.35
3.34
2.49
2.48
2.47
1.07
1.07
1.06
1.05
1.04
1.04
1.03
1.02
1.01
1.01
1.00
0.99
4.72
1.99
0.81
2.00
2.00
21.06
f1 (ppm)

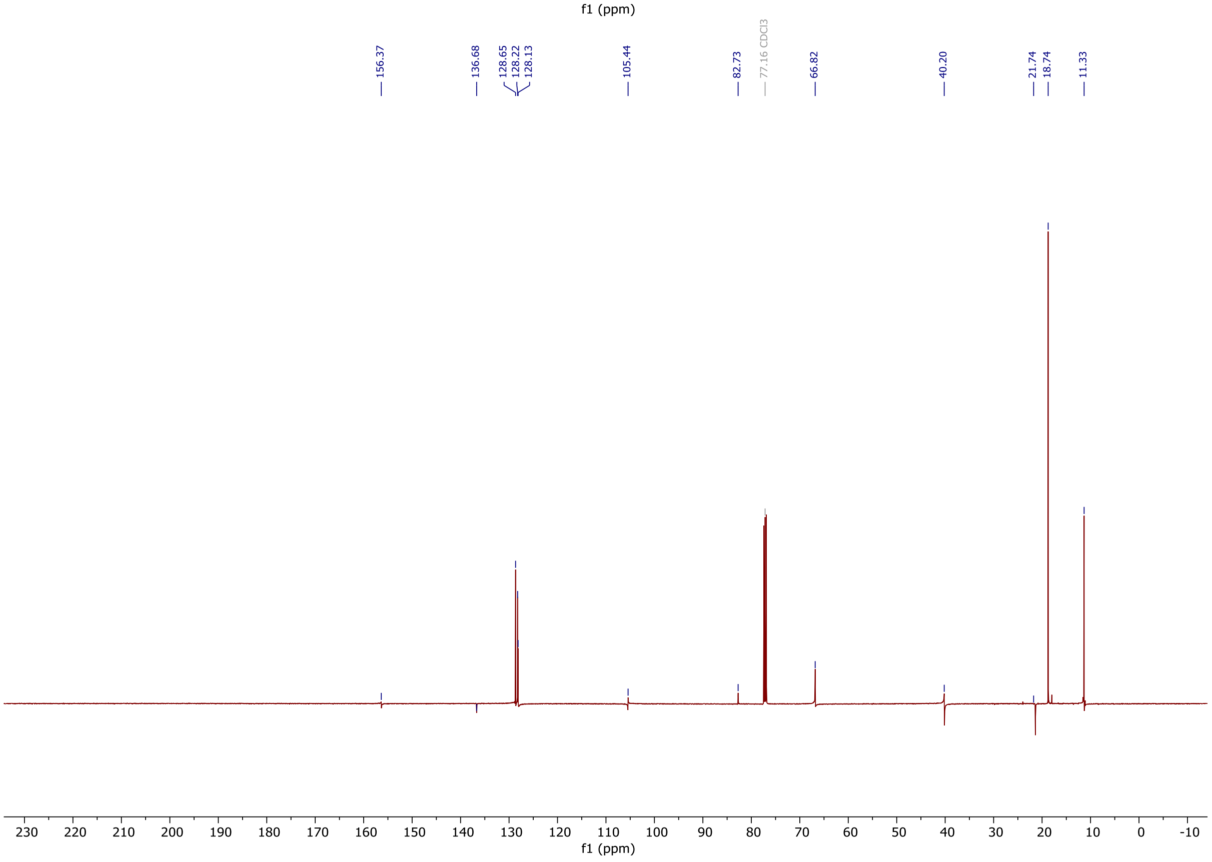
156.37
136.68
128.65
128.22
128.13
105.44
82.73
77.16 CDCl3
66.82
40.20
21.74
18.74
11.33
f1 (ppm)

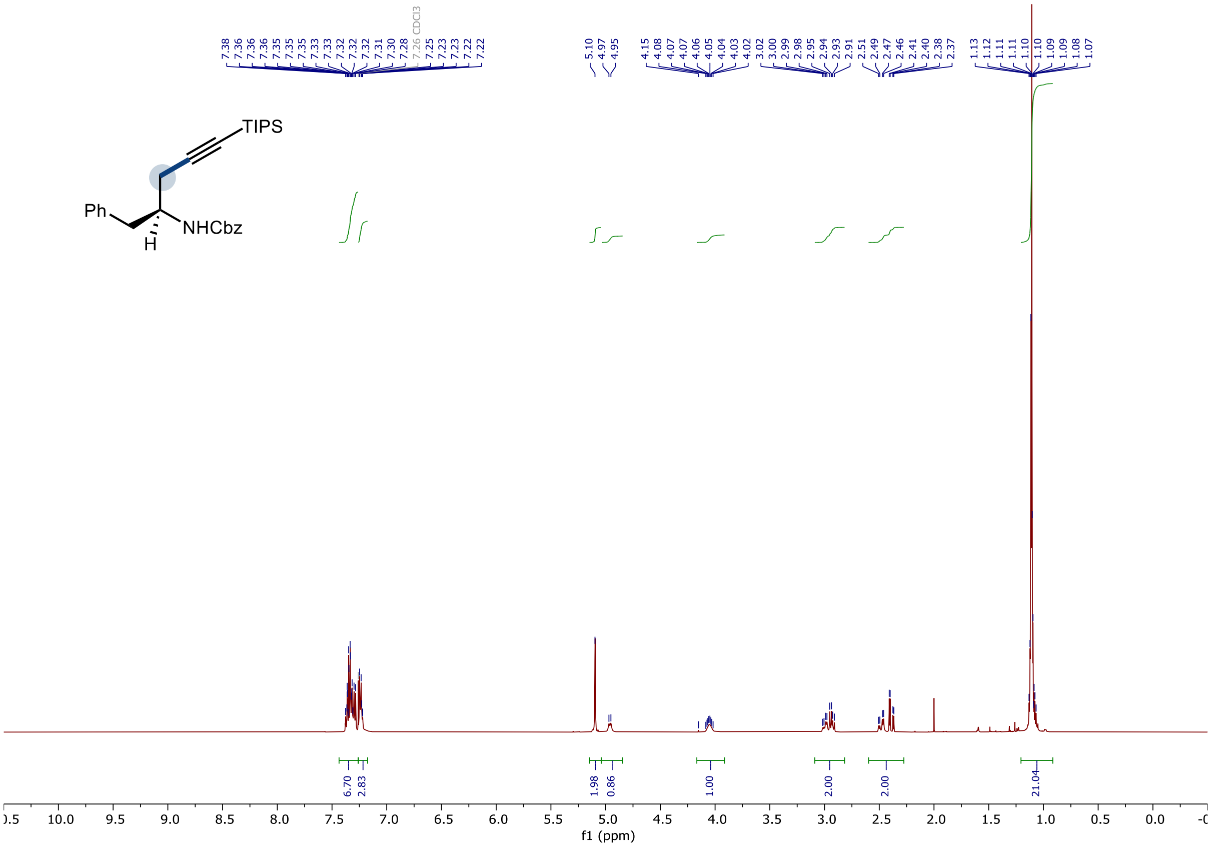
TIPS
Ph
NHCbz
H
7.38 7.36 7.36 7.36 7.35 7.35 7.35 7.33 7.33 7.32 7.32 7.32 7.31 7.30 7.28 7.26 CDCl3 7.25 7.23 7.23 7.22 7.22
5.10 4.97 4.95
4.15 4.08 4.07 4.06 4.05 4.04 4.03 4.02 3.02 3.00 2.99 2.98 2.95 2.94 2.93 2.91 2.51 2.49 2.47 2.46 2.41 2.40 2.38 2.37
1.13 1.12 1.11 1.11 1.10 1.09 1.09 1.08 1.07
6.70 2.83 1.98 0.86 1.00 2.00 2.00 21.04
f1 (ppm)

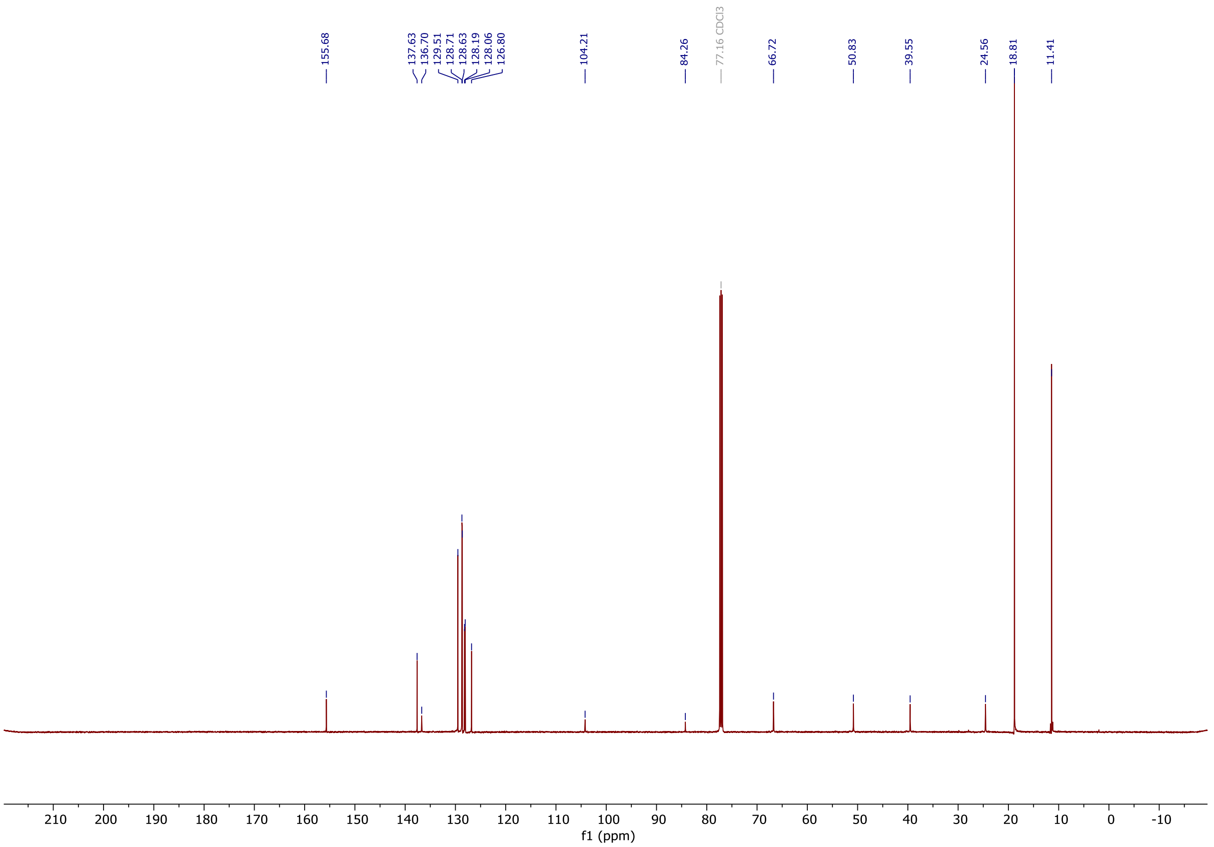
155.68
137.63 136.70 129.51 128.71 128.63 128.19 128.06 126.80
104.21
84.26
77.16 CDCl3
66.72
50.83
39.55
24.56
18.81
11.41
f1 (ppm)

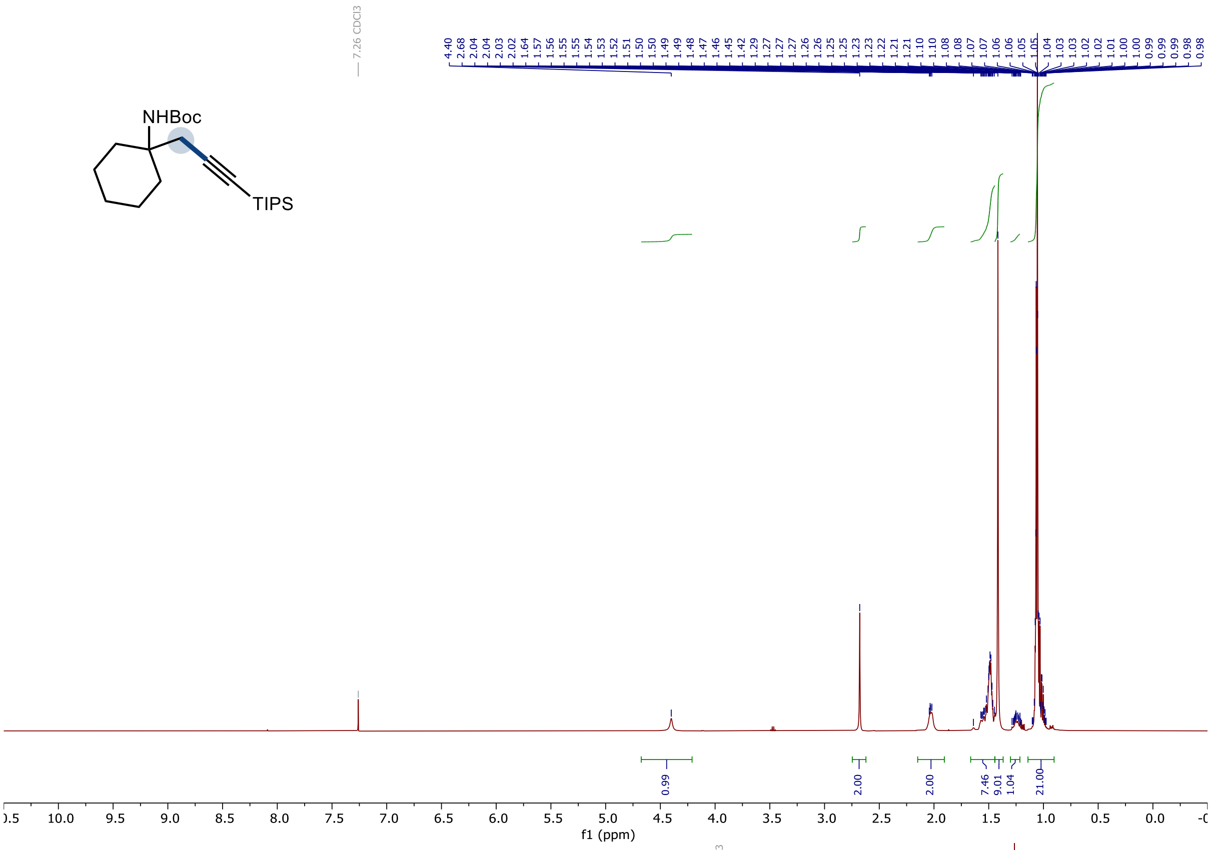
NHBoc
TIPS

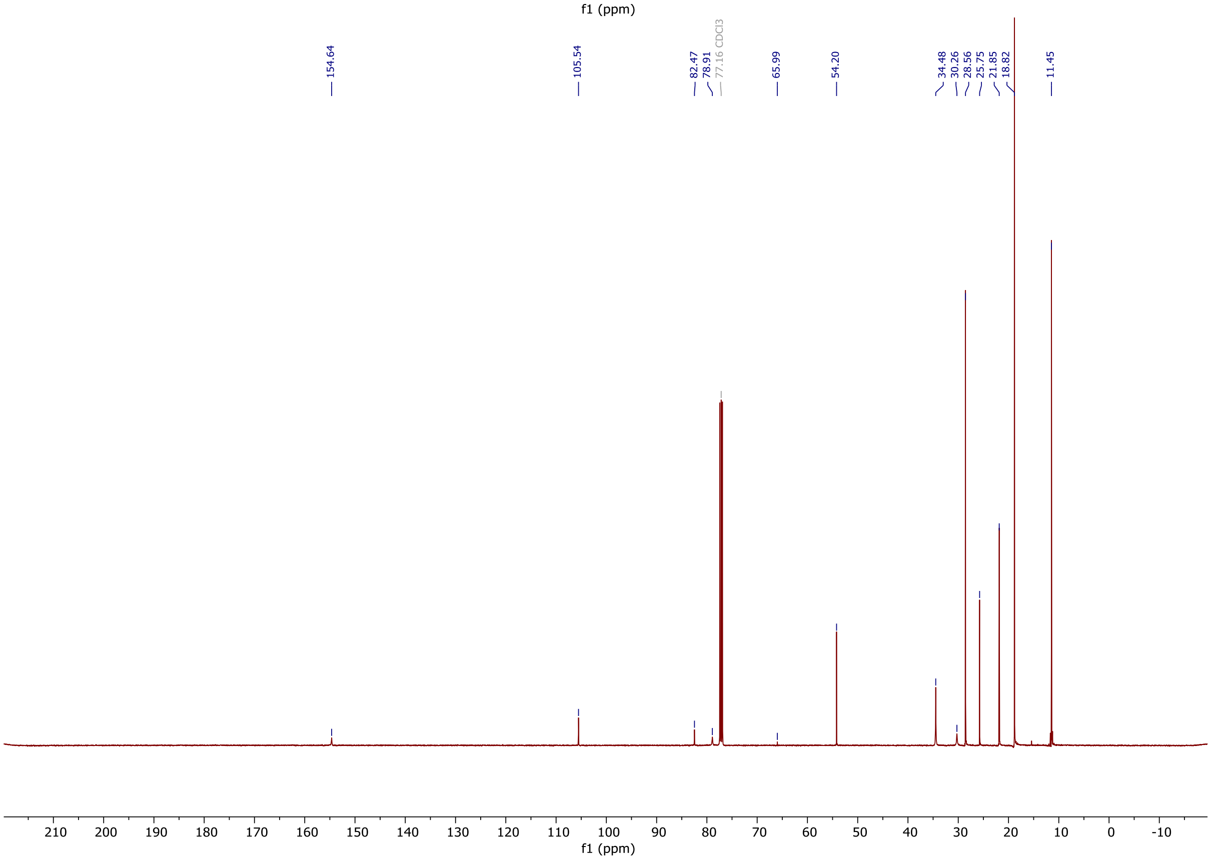

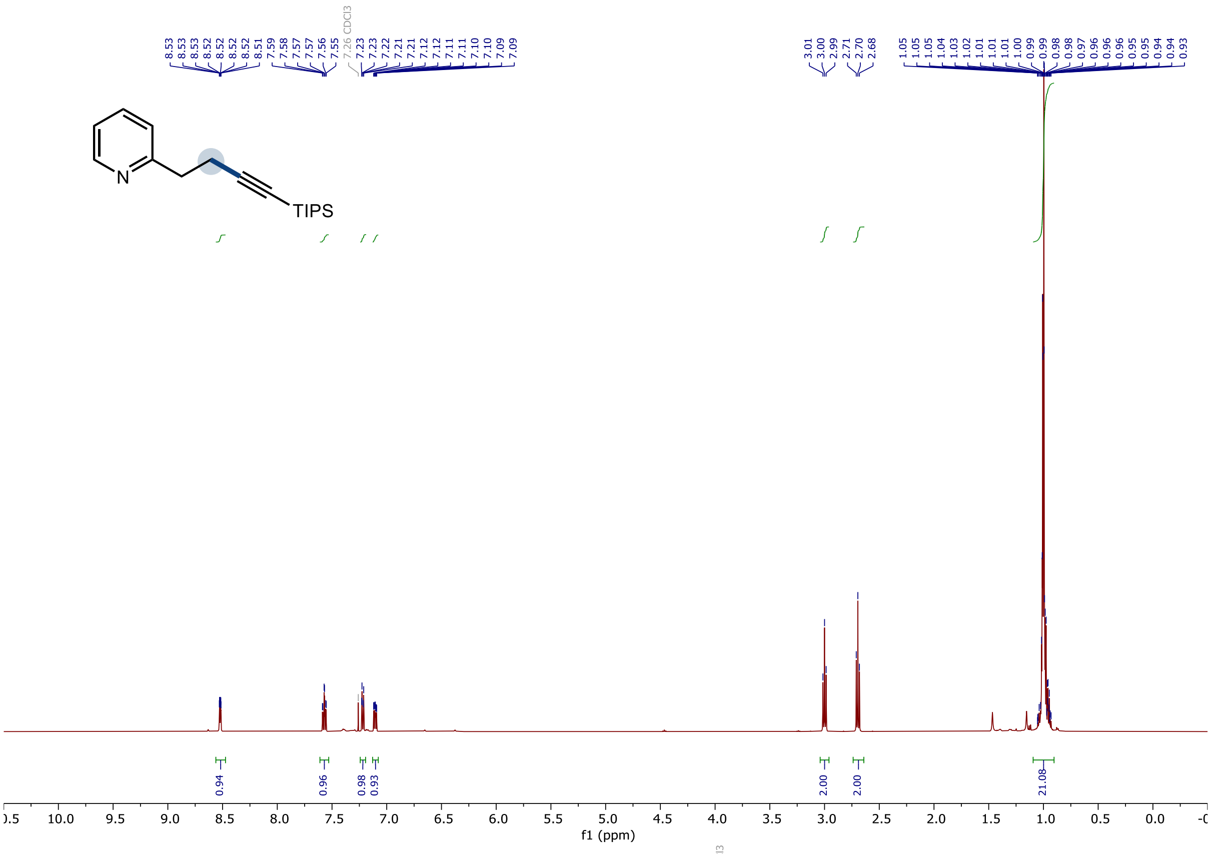
8.53
8.53
8.53
8.52
8.52
8.52
8.52
8.51
7.59
7.58
7.57
7.57
7.56
7.55
7.26 CDCl3
7.23
7.23
7.22
7.21
7.21
7.12
7.12
7.11
7.11
7.10
7.09
7.09
3.01
3.00
2.99
2.71
2.70
2.68
1.05
1.05
1.05
1.04
1.03
1.02
1.01
1.01
1.01
1.00
0.99
0.99
0.98
0.98
0.97
0.96
0.96
0.96
0.95
0.95
0.94
0.94
0.93
N
TIPS
0.94
0.96
0.98
0.93
2.00
2.00
21.08
f1 (ppm)

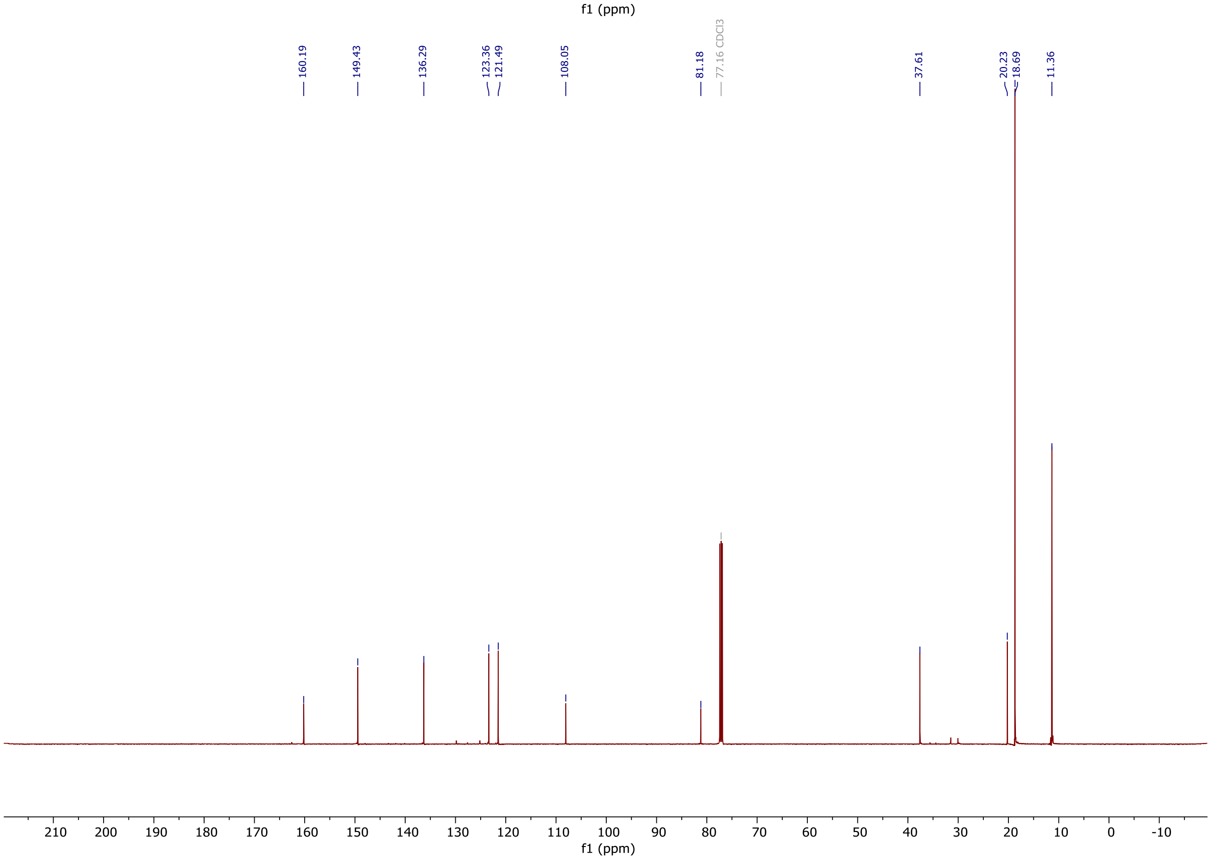
160.19
149.43
136.29
123.36
121.49
108.05
81.18
77.16 CDCl3
37.61
20.23
18.69
11.36
f1 (ppm)

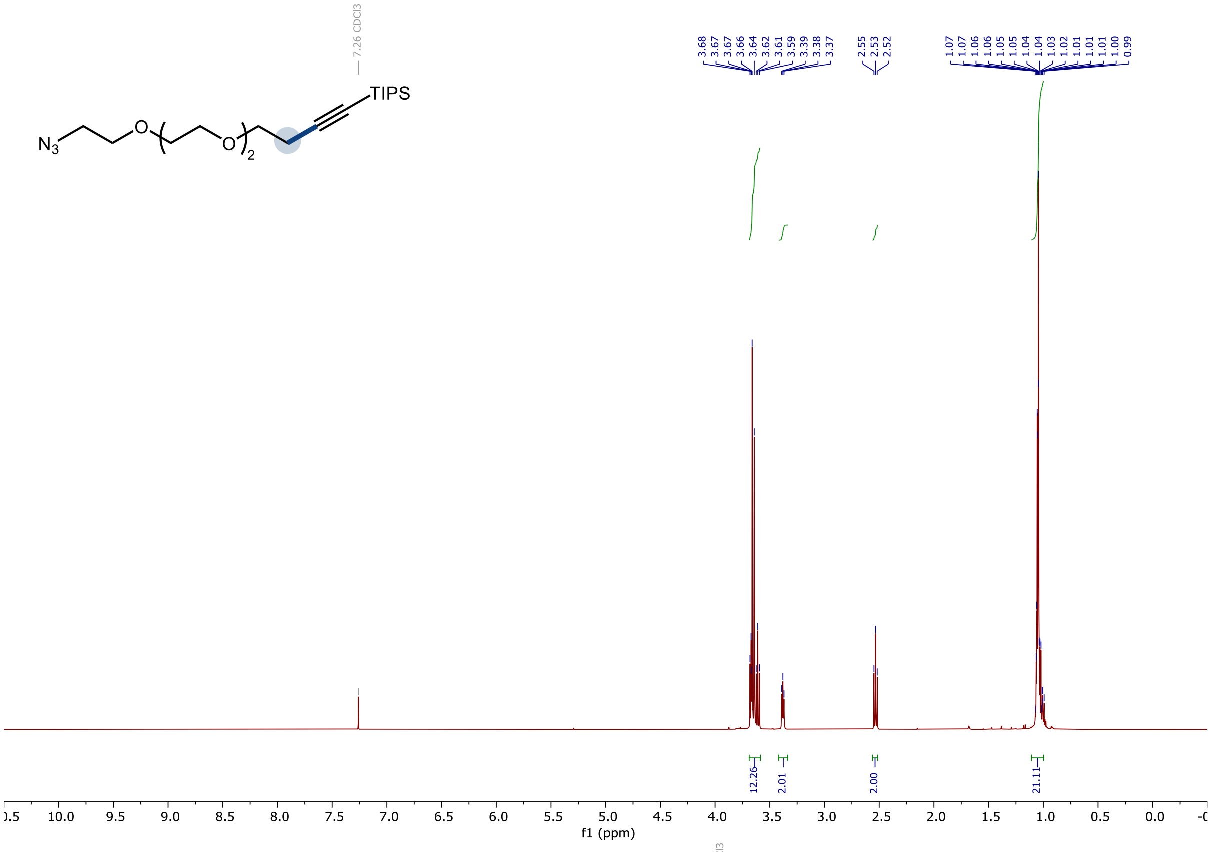

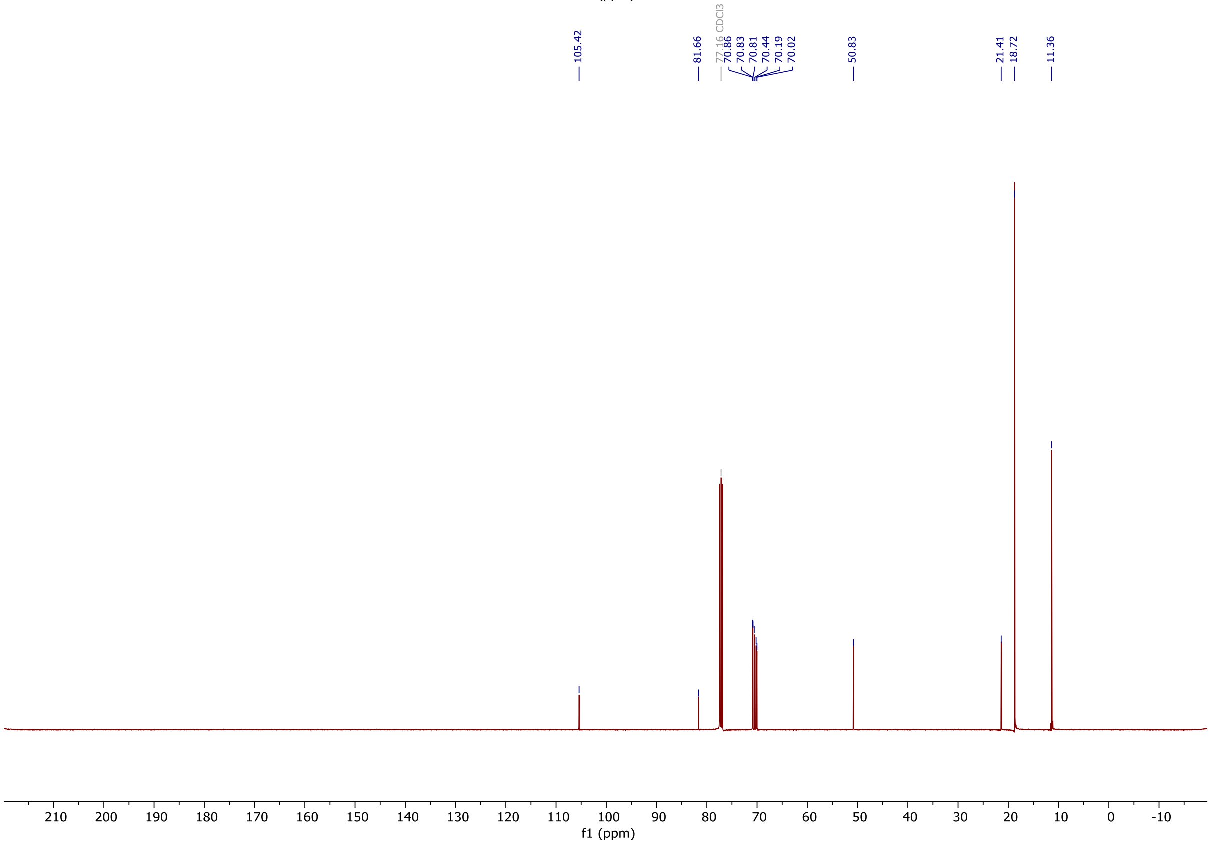

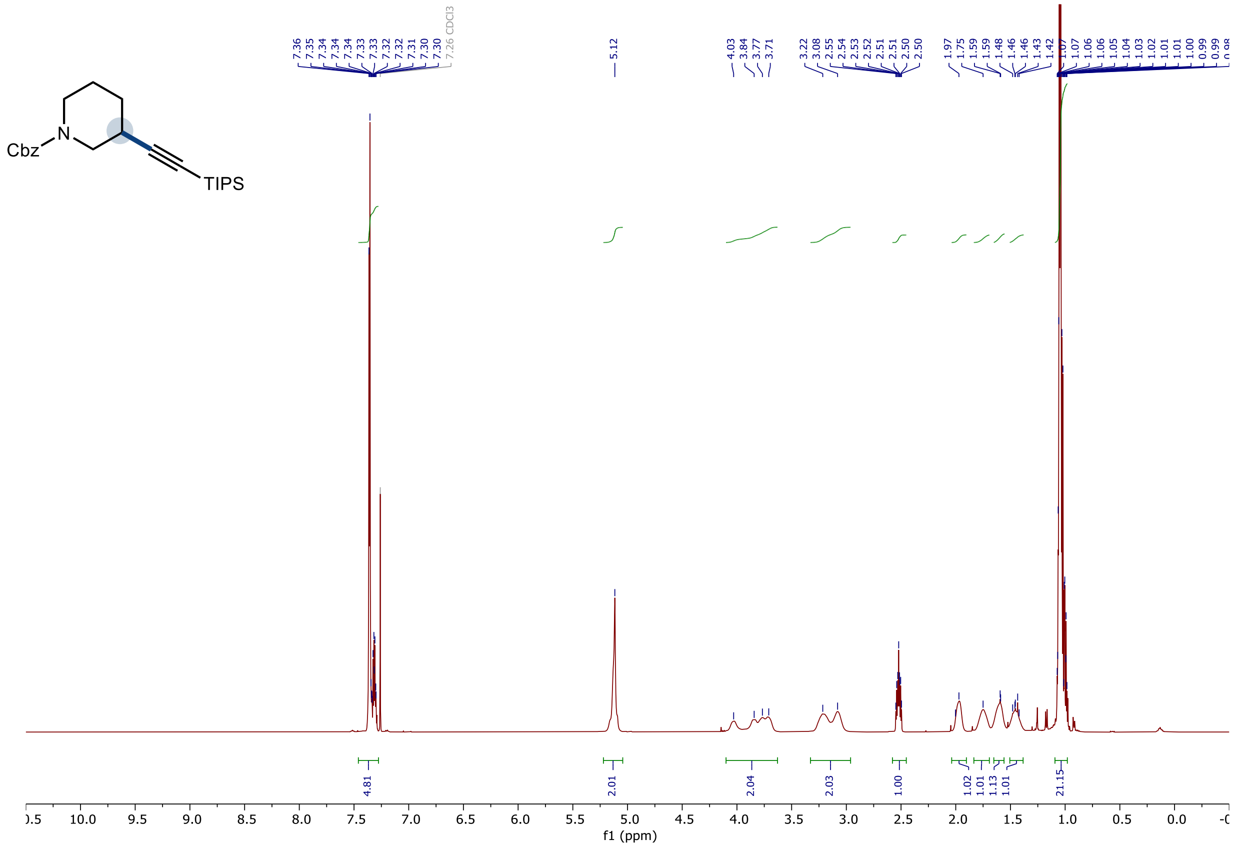
Cbz
N
TIPS
7.36 7.35 7.34 7.34 7.34 7.33 7.33 7.32 7.32 7.31 7.30 7.30 7.26 CDCl3
5.12
4.03 3.84 3.77 3.71
3.22 3.08 2.55 2.54 2.53 2.52 2.51 2.51 2.50 2.50
1.97 1.75 1.59 1.59 1.48 1.46 1.46 1.43 1.42
1.07 1.07 1.06 1.06 1.05 1.04 1.03 1.02 1.01 1.01 1.00 0.99 0.99 [illegible]
4.81 2.01 2.04 2.03 1.00 1.02 1.01 1.13 1.01 21.15
f1 (ppm)

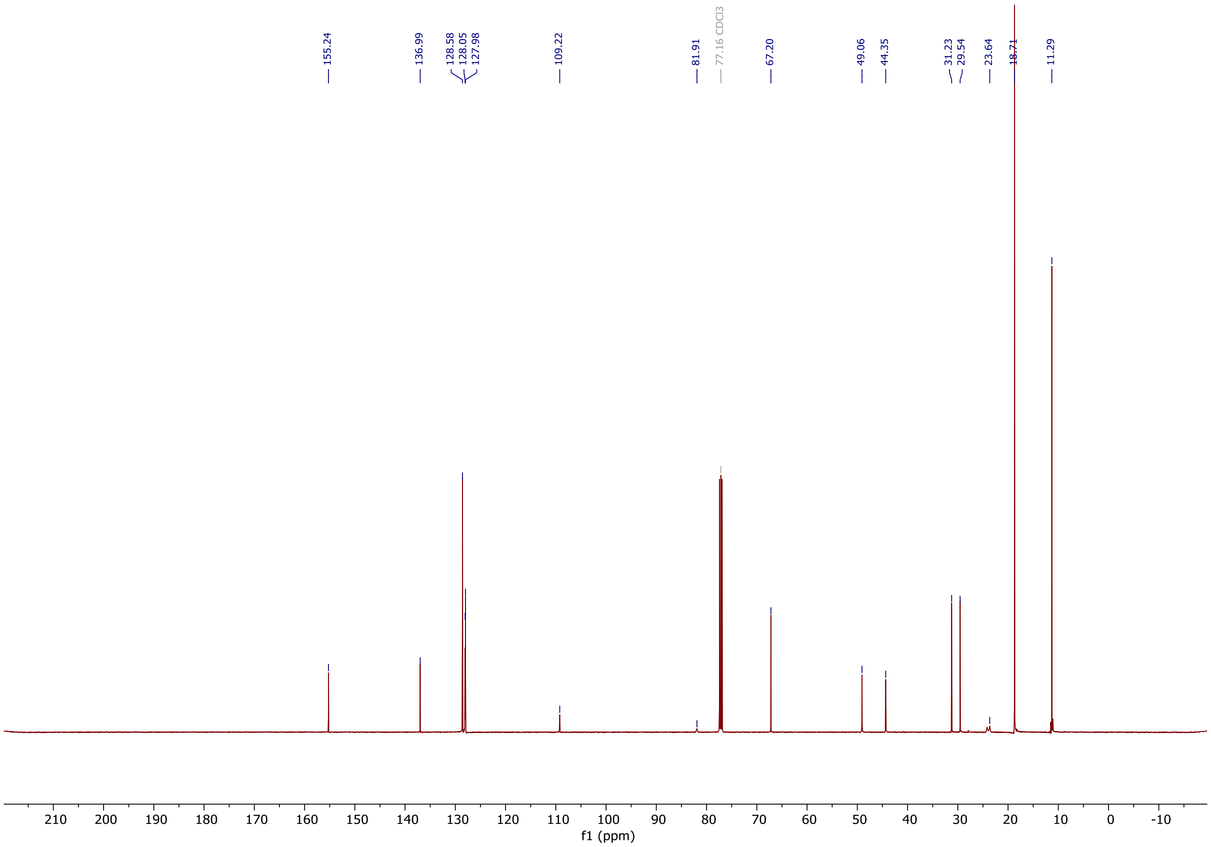
155.24
136.99
128.58 128.05 127.98
109.22
81.91
77.16 CDCl3
67.20
49.06
44.35
31.23 29.54
23.64
[illegible]
11.29
f1 (ppm)

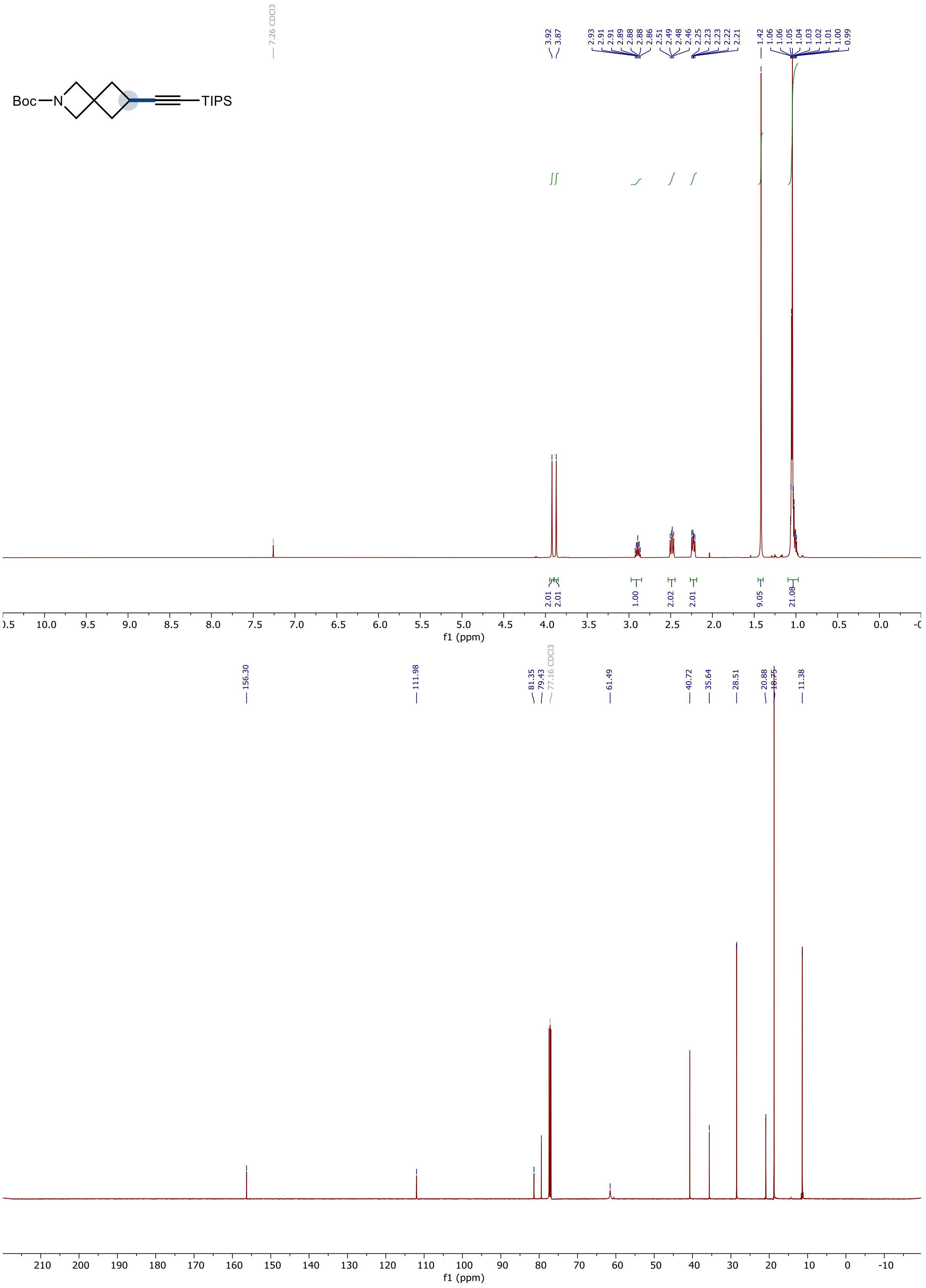
Boc
N
TIPS
7.26 CDCl3
3.92
3.87
2.93
2.91
2.91
2.89
2.88
2.88
2.86
2.51
2.49
2.48
2.46
2.25
2.23
2.23
2.22
2.21
1.42
1.06
1.06
1.05
1.04
1.03
1.02
1.01
1.00
0.99
2.01
2.01
1.00
2.02
2.01
9.05
21.08
f1 (ppm)
156.30
111.98
81.35
79.43
77.16 CDCl3
61.49
40.72
35.64
28.51
20.88
18.75
11.38
f1 (ppm)

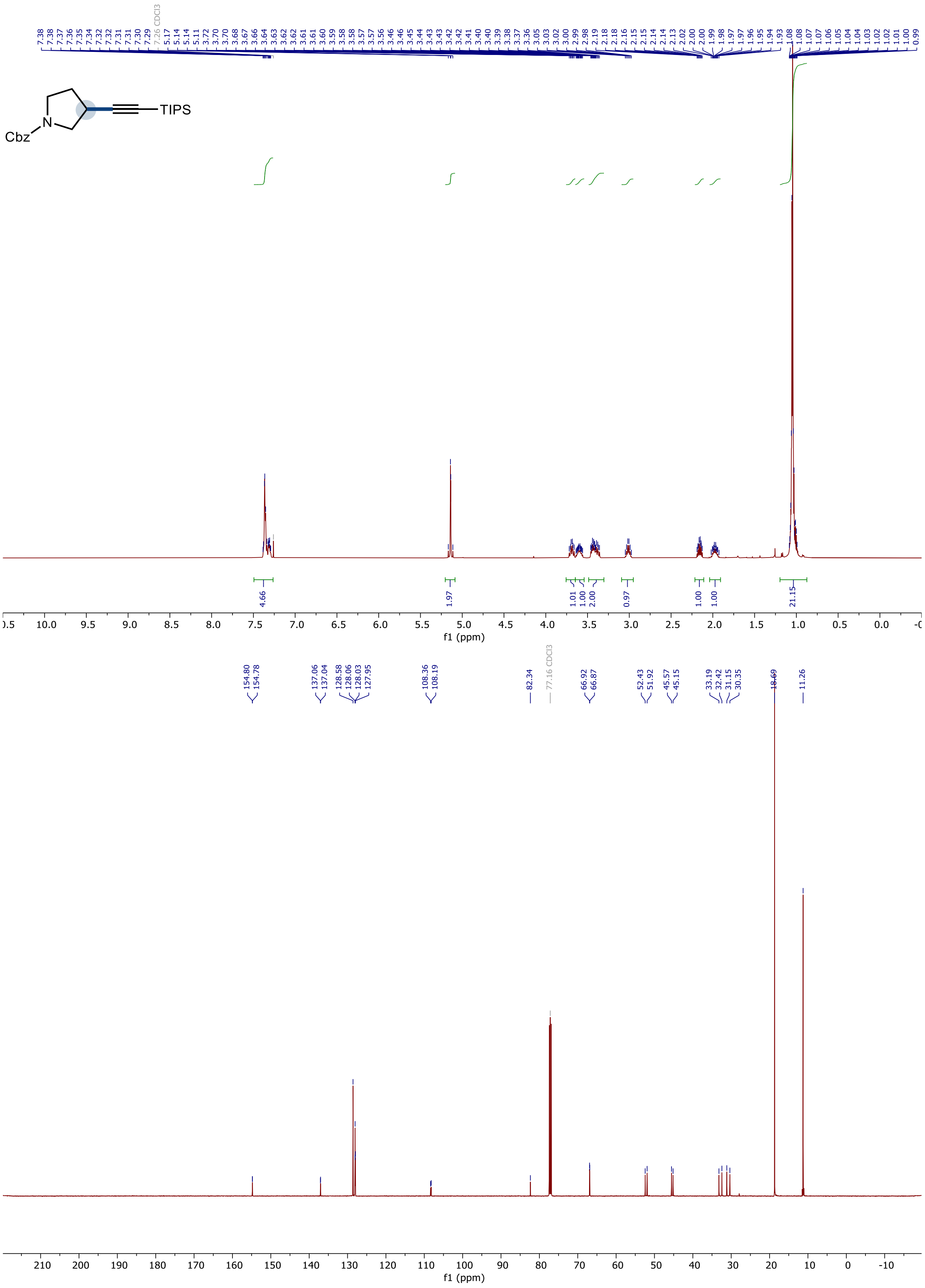

Cbz
N
TIPS
f1 (ppm)
f1 (ppm)

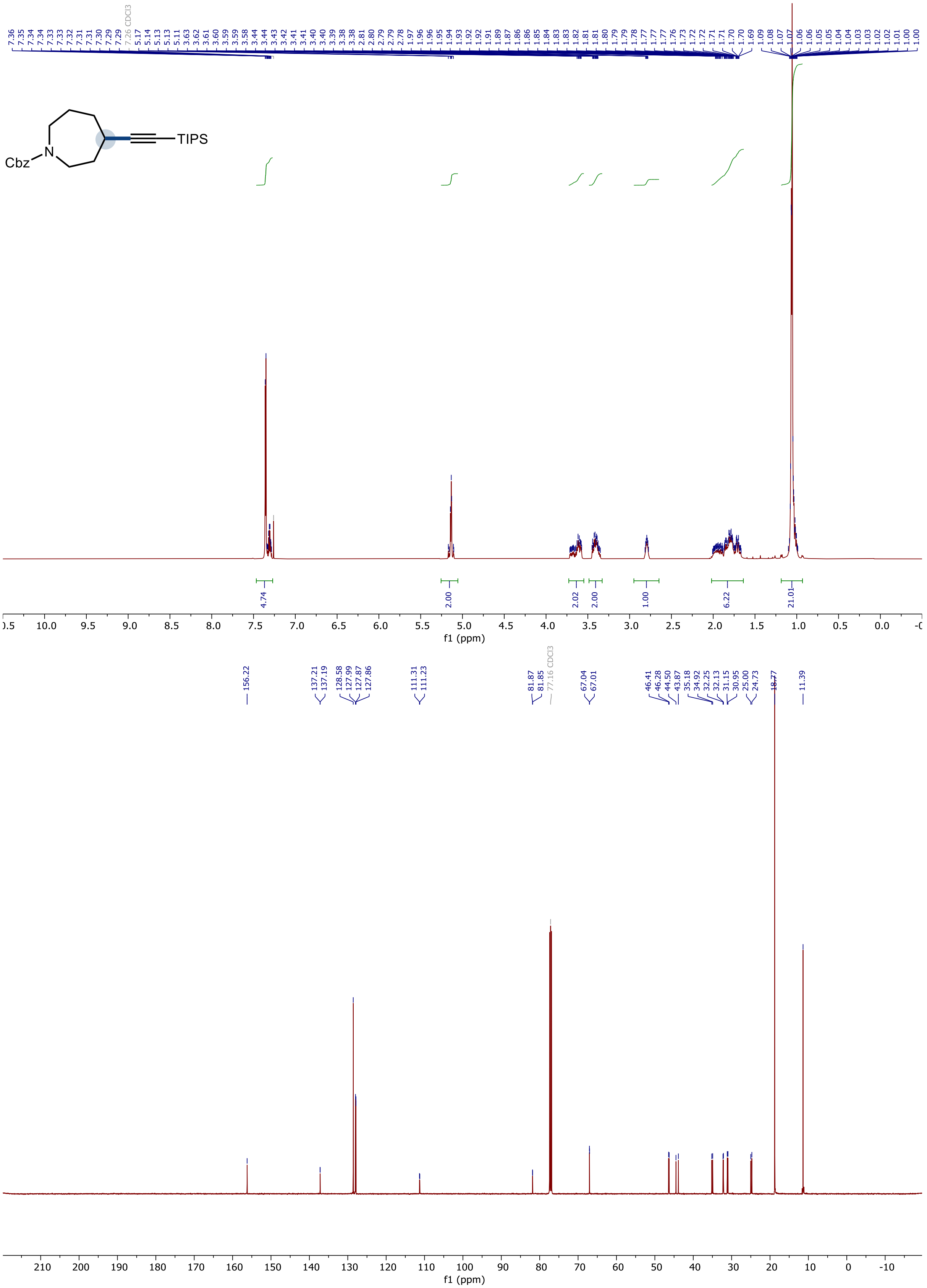
TIPS
Cbz
N
f1 (ppm)
f1 (ppm)

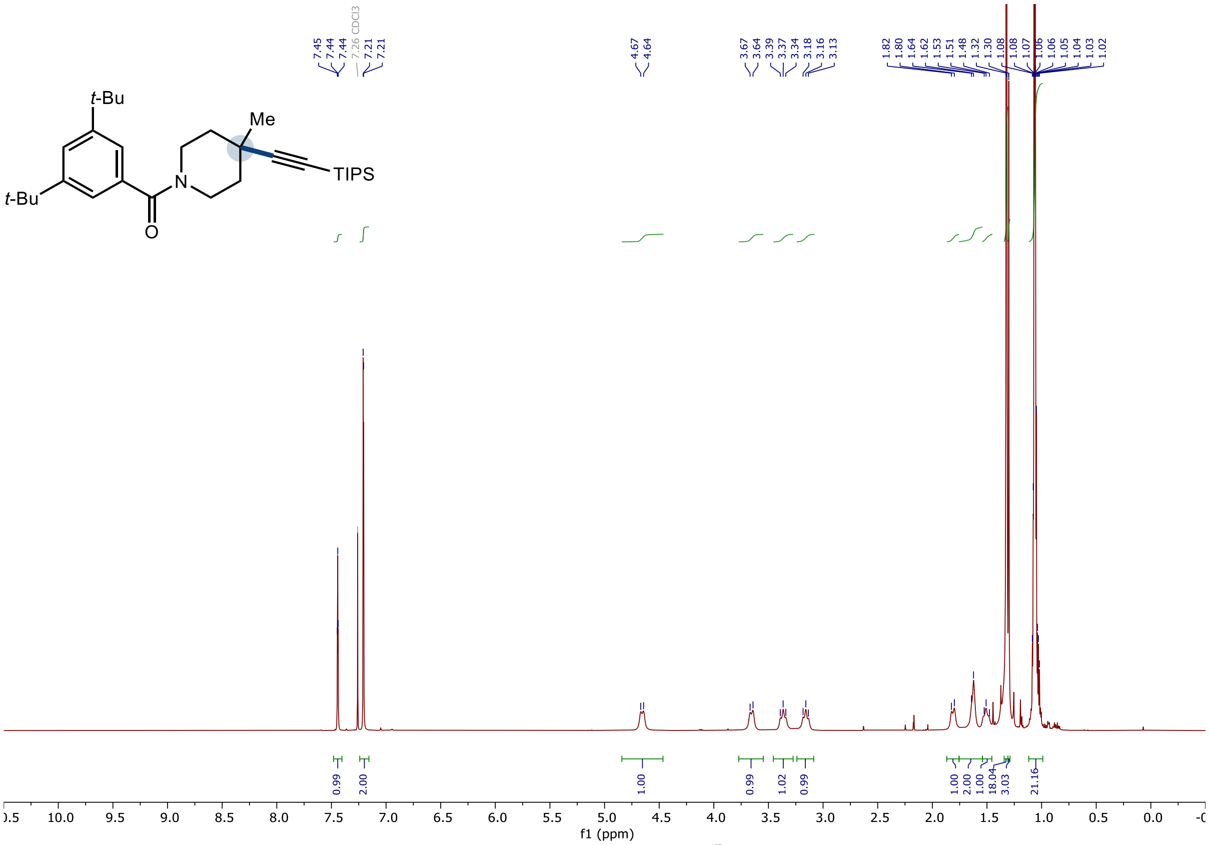
t-Bu
Me
TIPS
N
O
t-Bu
7.45
7.44
7.44
7.26 CDCl3
7.21
7.21
4.67
4.64
3.67
3.64
3.39
3.37
3.34
3.18
3.16
3.13
1.82
1.80
1.64
1.62
1.53
1.51
1.48
1.32
1.30
1.08
1.08
1.07
1.06
1.05
1.04
1.03
1.02
0.99
2.00
1.00
0.99
1.02
0.99
1.00
2.00
1.00
18.04
3.03
21.16
f1 (ppm)

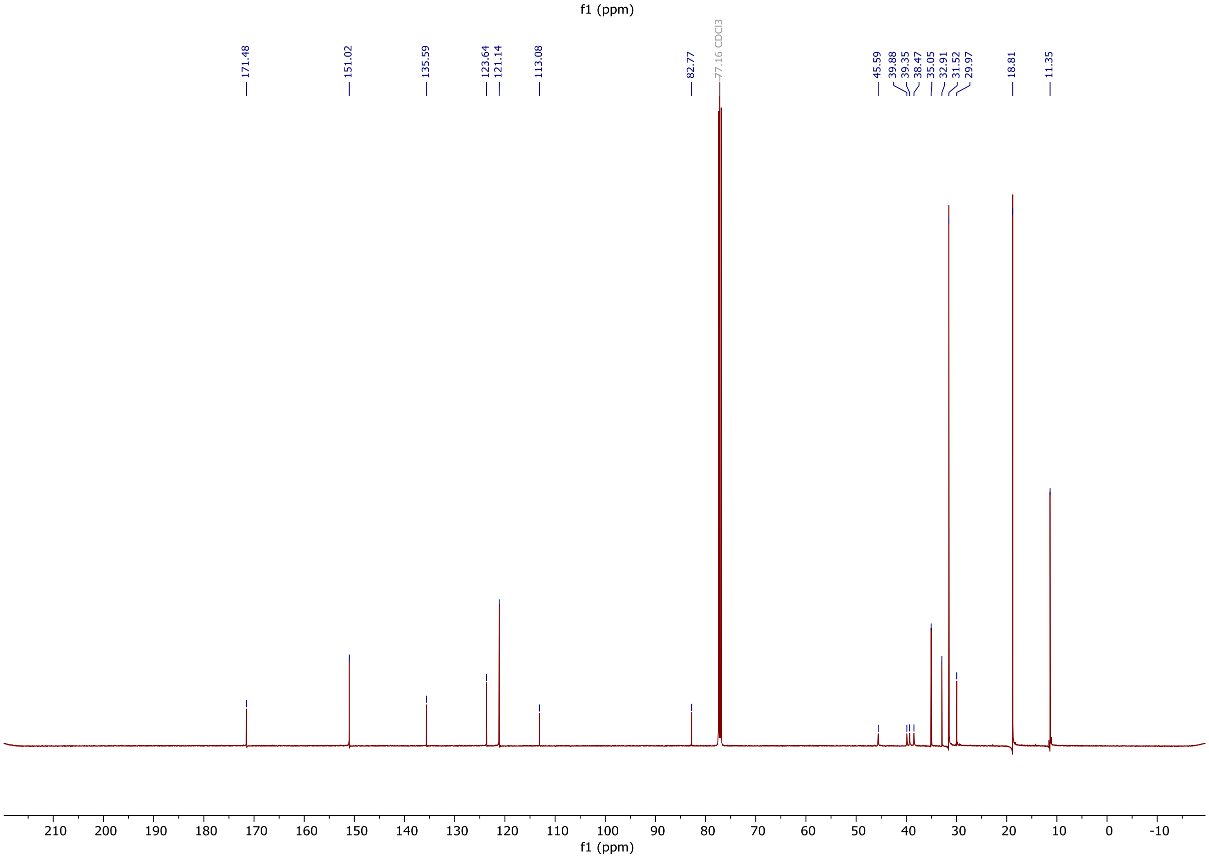
171.48
151.02
135.59
123.64
121.14
113.08
82.77
77.16 CDCl3
45.59
39.88
39.35
38.47
35.05
32.91
31.52
29.97
18.81
11.35
f1 (ppm)

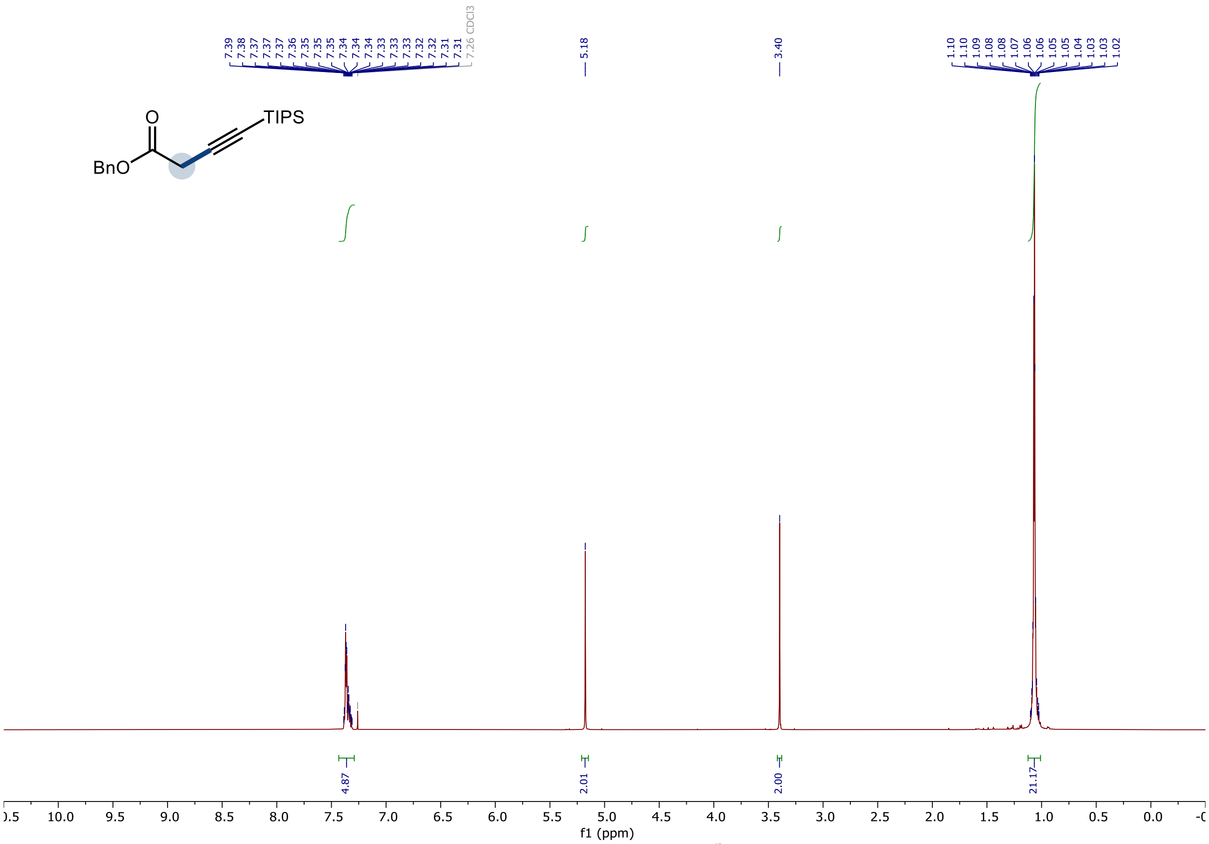
O
TIPS
BnO

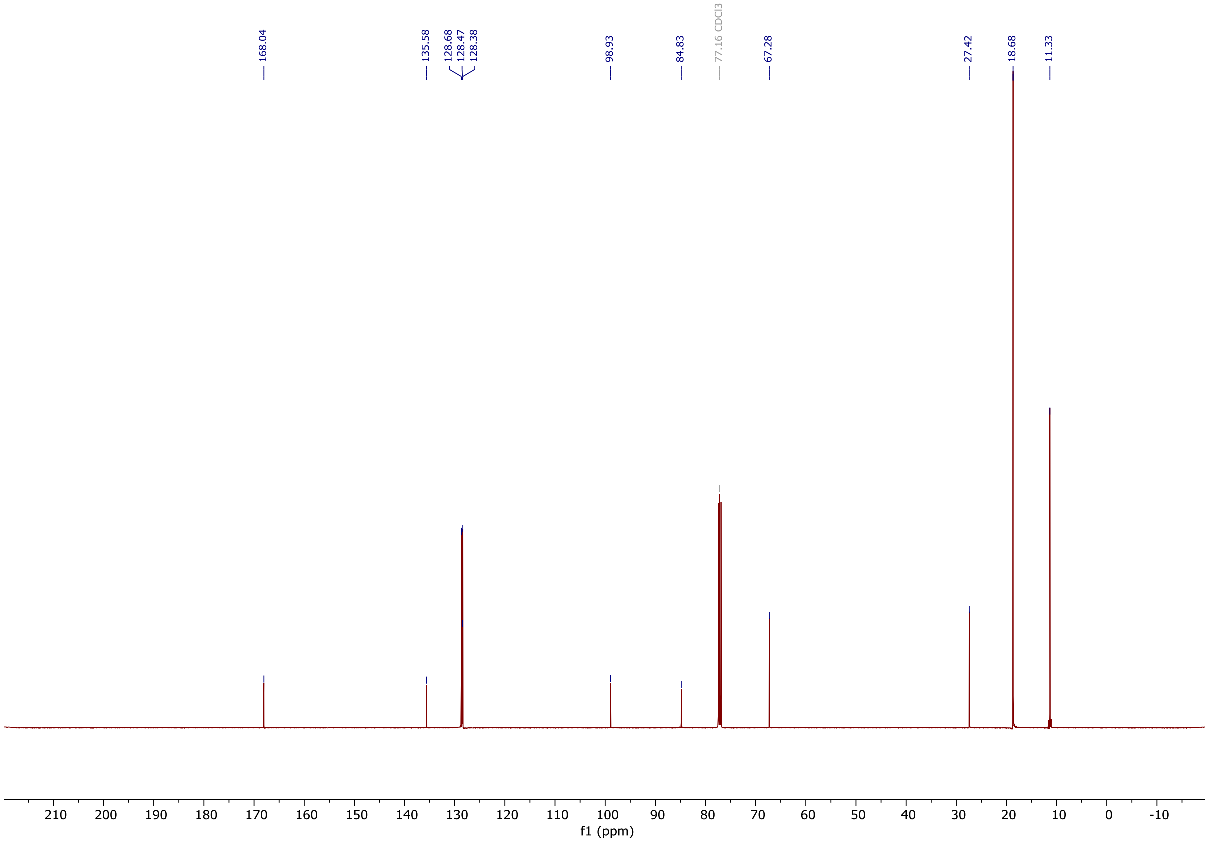

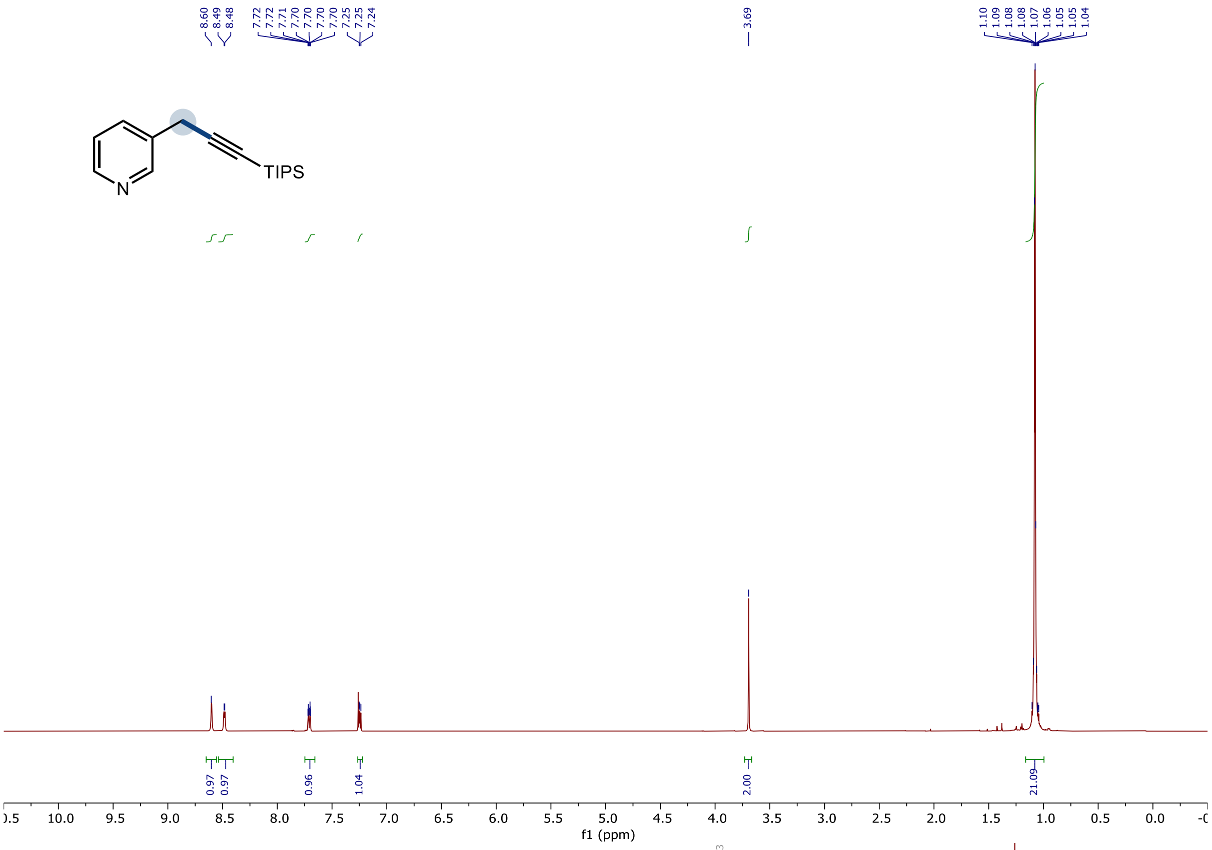
TIPS
N
8.60
8.49
8.48
7.72
7.72
7.71
7.70
7.70
7.70
7.25
7.25
7.24
3.69
1.10
1.09
1.08
1.08
1.07
1.06
1.05
1.05
1.04
0.97
0.97
0.96
1.04
2.00
21.09
f1 (ppm)

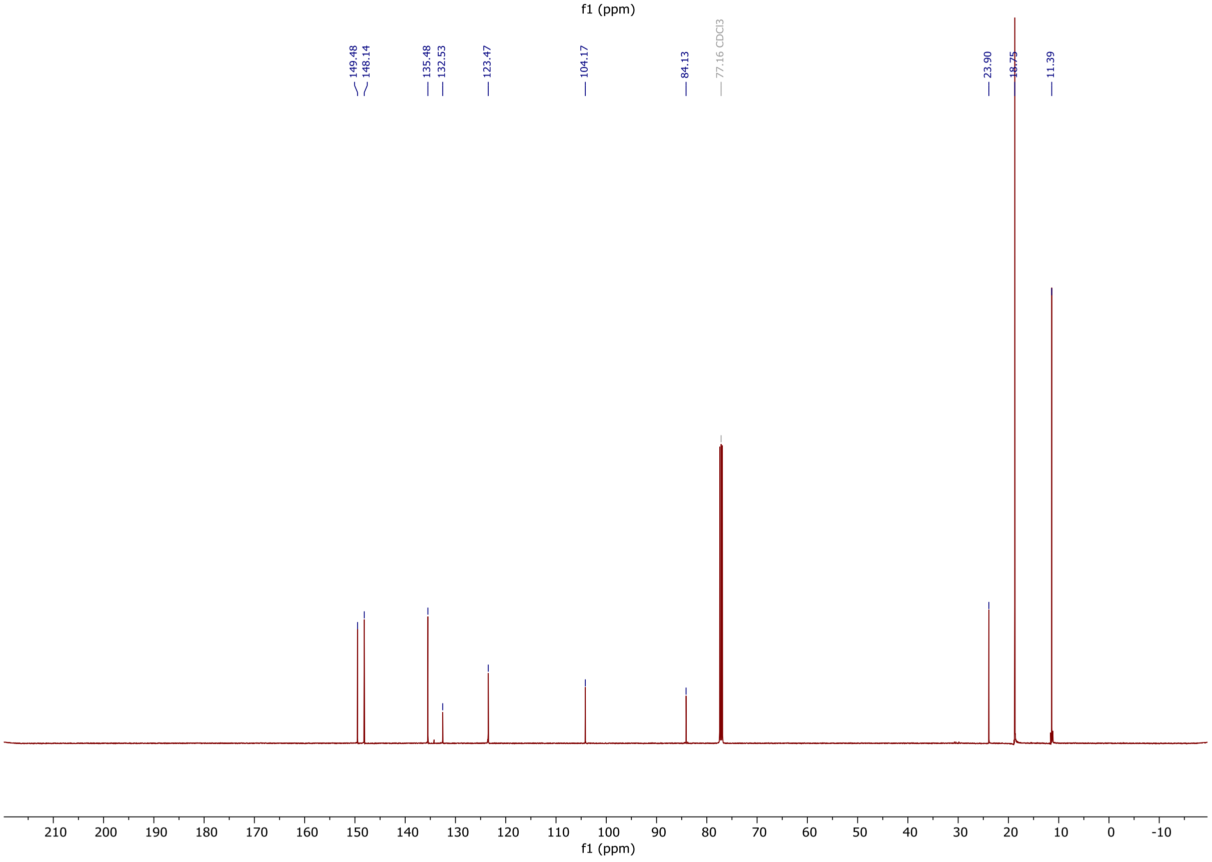
149.48
148.14
135.48
132.53
123.47
104.17
84.13
77.16 CDCl3
23.90
18.75
11.39
f1 (ppm)

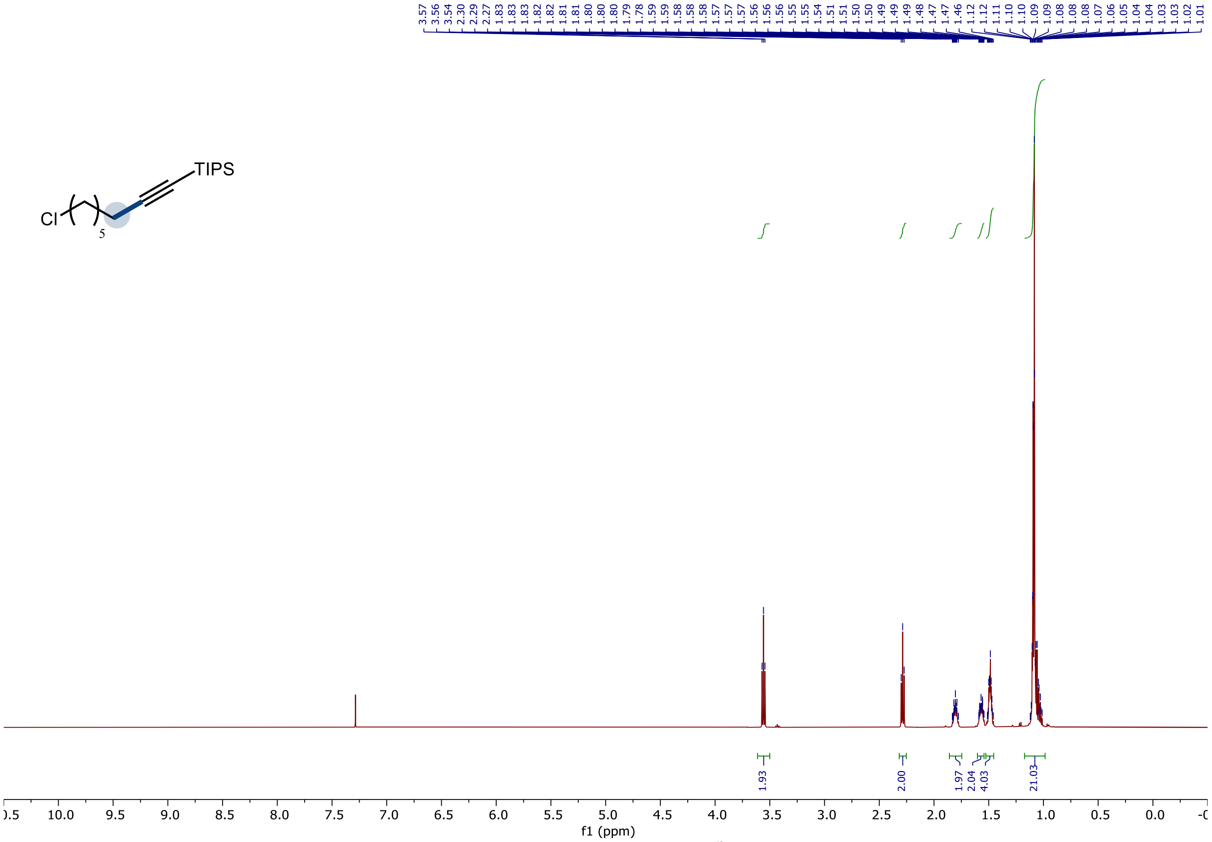
TIPS
Cl
5
3.57 3.56 3.54 2.30 2.29 2.27 1.83 1.83 1.83 1.82 1.82 1.81 1.81 1.80 1.80 1.80 1.79 1.78 1.59 1.59 1.58 1.58 1.58 1.57 1.57 1.57 1.56 1.56 1.55 1.55 1.54 1.51 1.51 1.50 1.50 1.49 1.49 1.49 1.48 1.47 1.46 1.12 1.12 1.11 1.10 1.10 1.09 1.09 1.08 1.08 1.08 1.07 1.06 1.05 1.04 1.04 1.03 1.03 1.02 1.01
1.93
2.00
1.97
2.04
4.03
21.03
f1 (ppm)

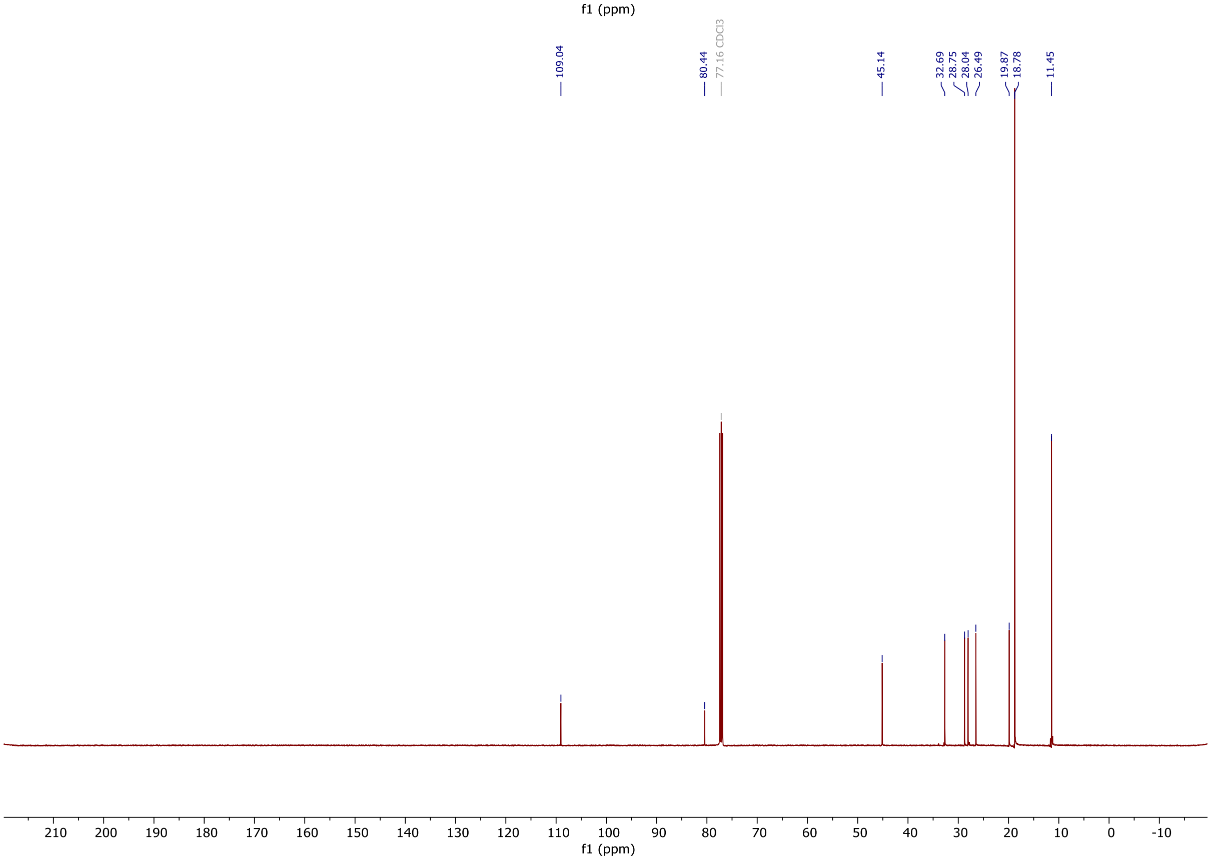
109.04
80.44
77.16 CDCl3
45.14
32.69
28.75
28.04
26.49
19.87
18.78
11.45
f1 (ppm)

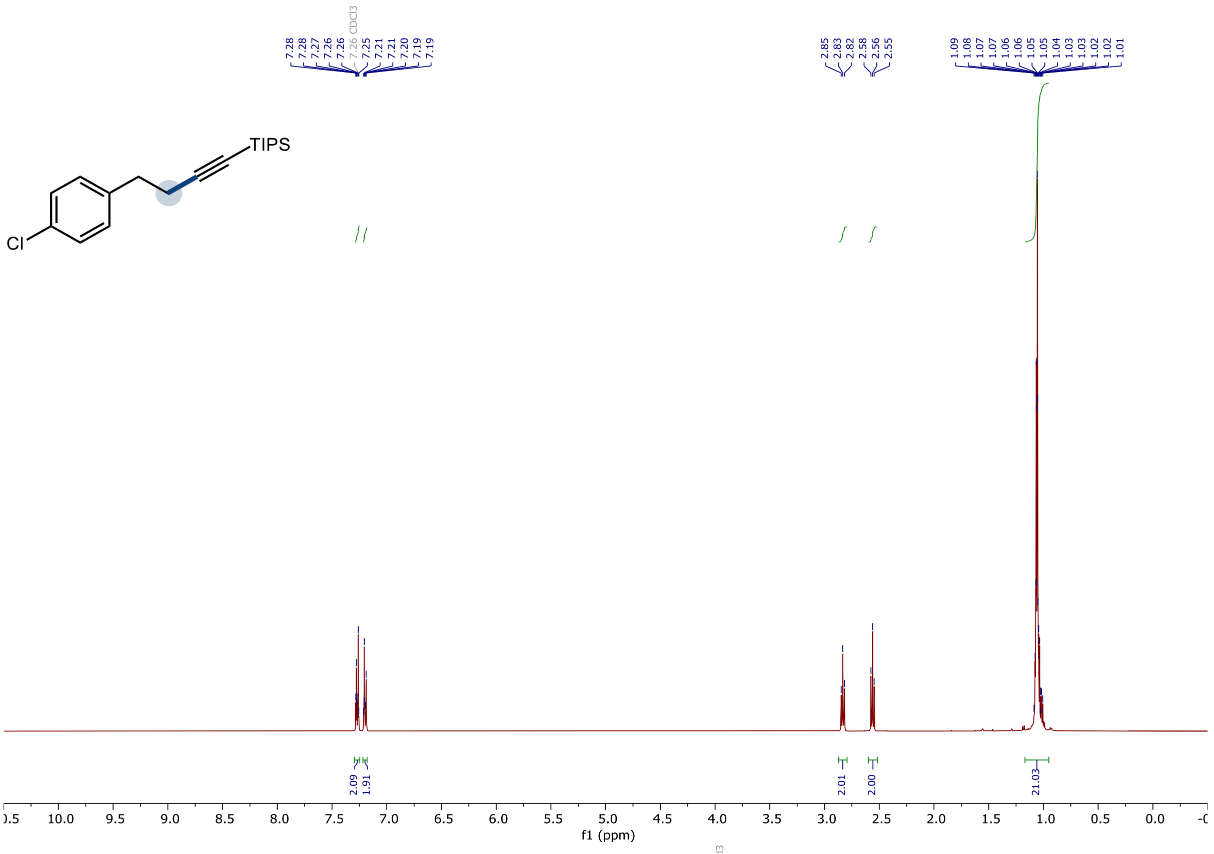
TIPS
Cl
7.28
7.28
7.27
7.26
7.26
7.26 CDCl3
7.25
7.21
7.21
7.20
7.19
7.19
2.85
2.83
2.82
2.58
2.56
2.55
1.09
1.08
1.07
1.07
1.06
1.06
1.05
1.05
1.04
1.03
1.03
1.02
1.02
1.01
2.09
1.91
2.01
2.00
21.03
f1 (ppm)

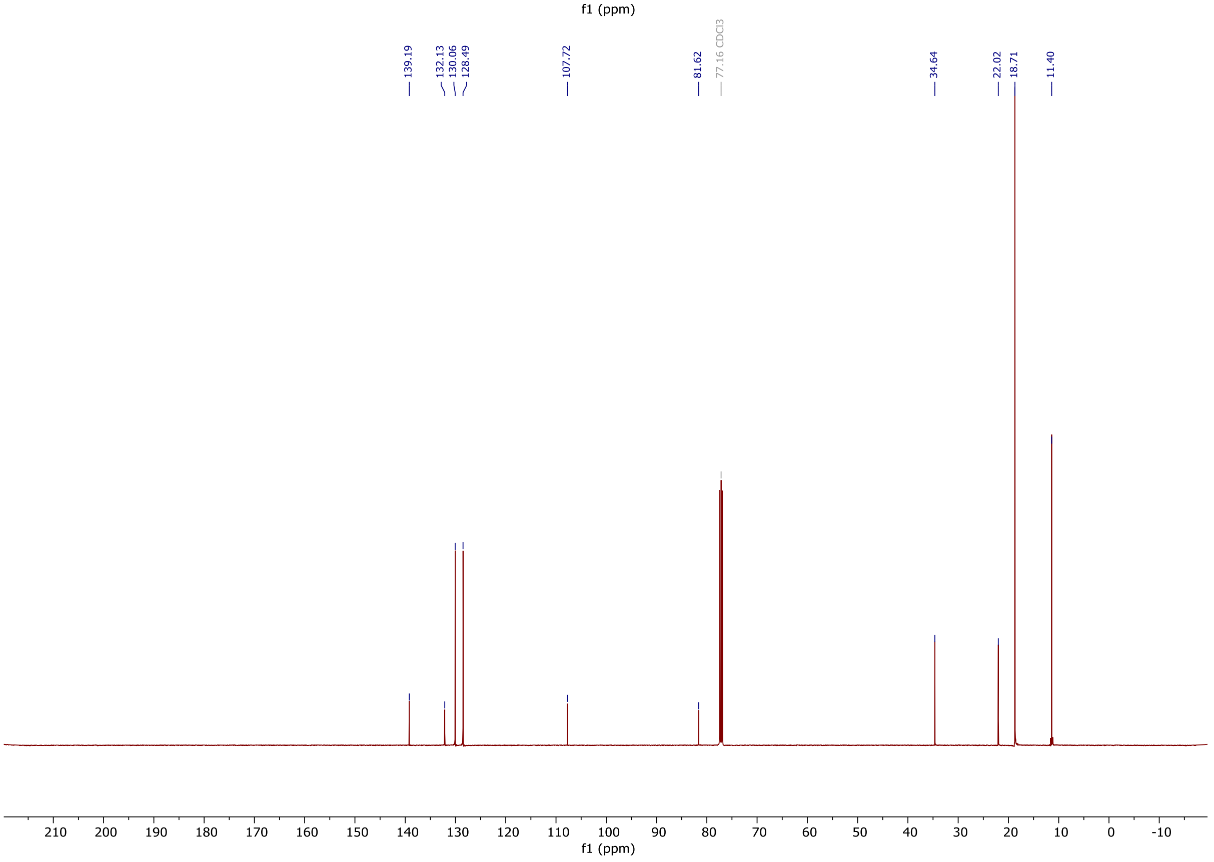
139.19
132.13
130.06
128.49
107.72
81.62
77.16 CDCl3
34.64
22.02
18.71
11.40
f1 (ppm)

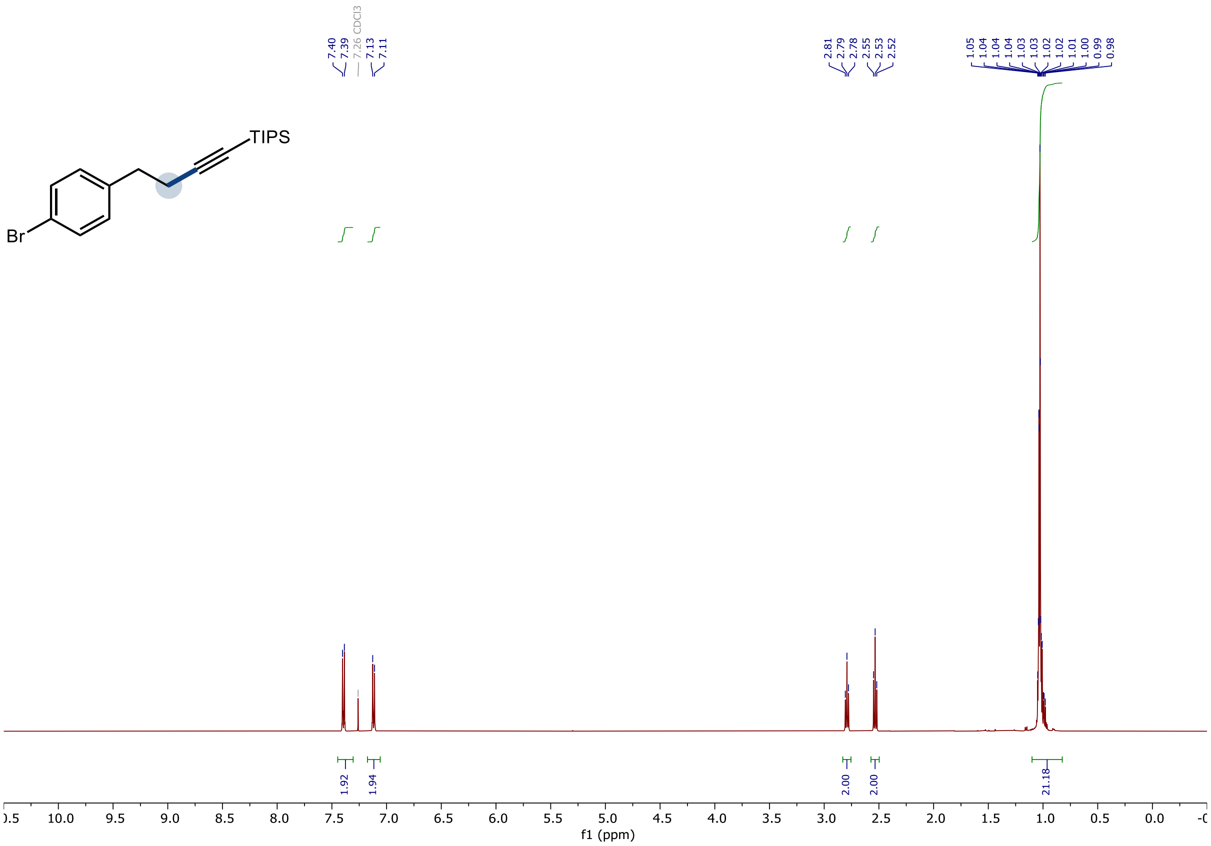

TIPS
Br
7.40
7.39
7.26 CDCl3
7.13
7.11
2.81
2.79
2.78
2.55
2.53
2.52
1.05
1.04
1.04
1.04
1.03
1.03
1.02
1.02
1.01
1.00
0.99
0.98
1.92
1.94
2.00
2.00
21.18
f1 (ppm)

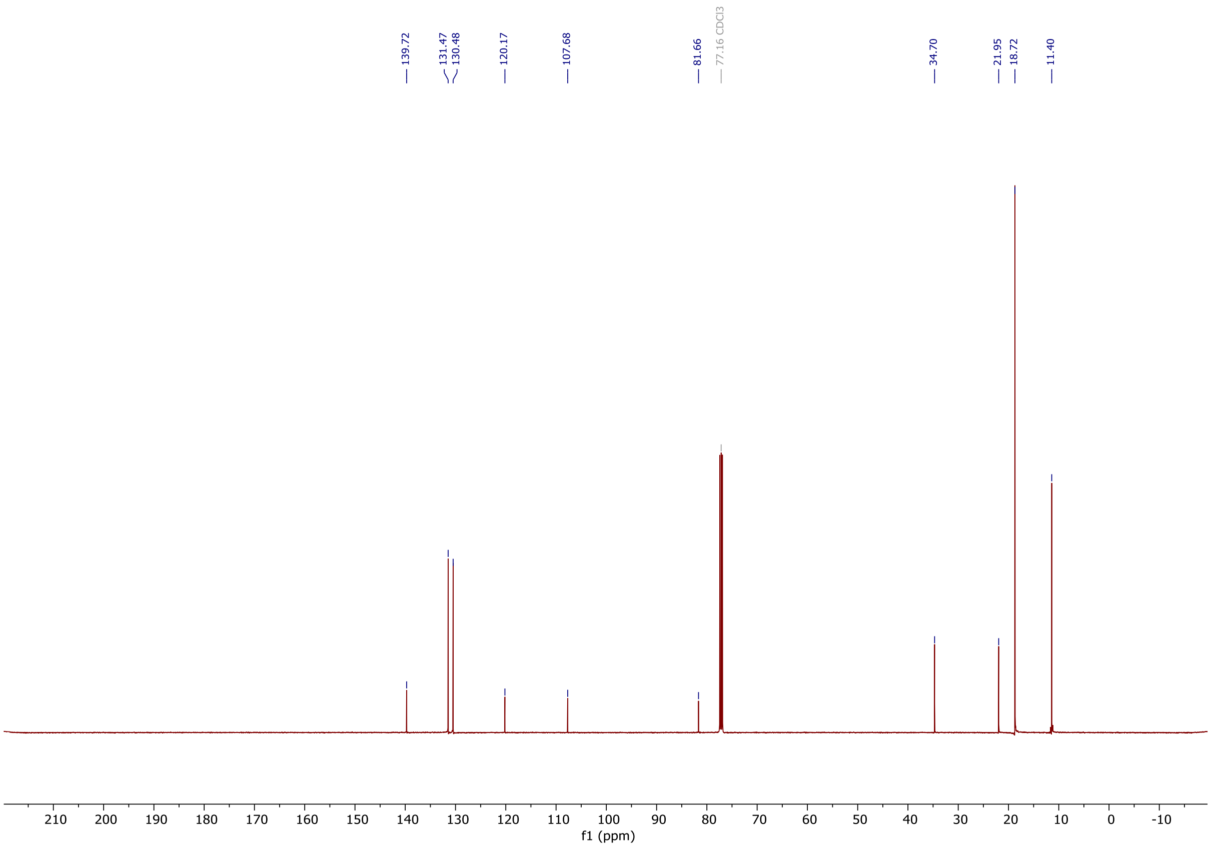

139.72
131.47
130.48
120.17
107.68
81.66
77.16 CDCl3
34.70
21.95
18.72
11.40
f1 (ppm)

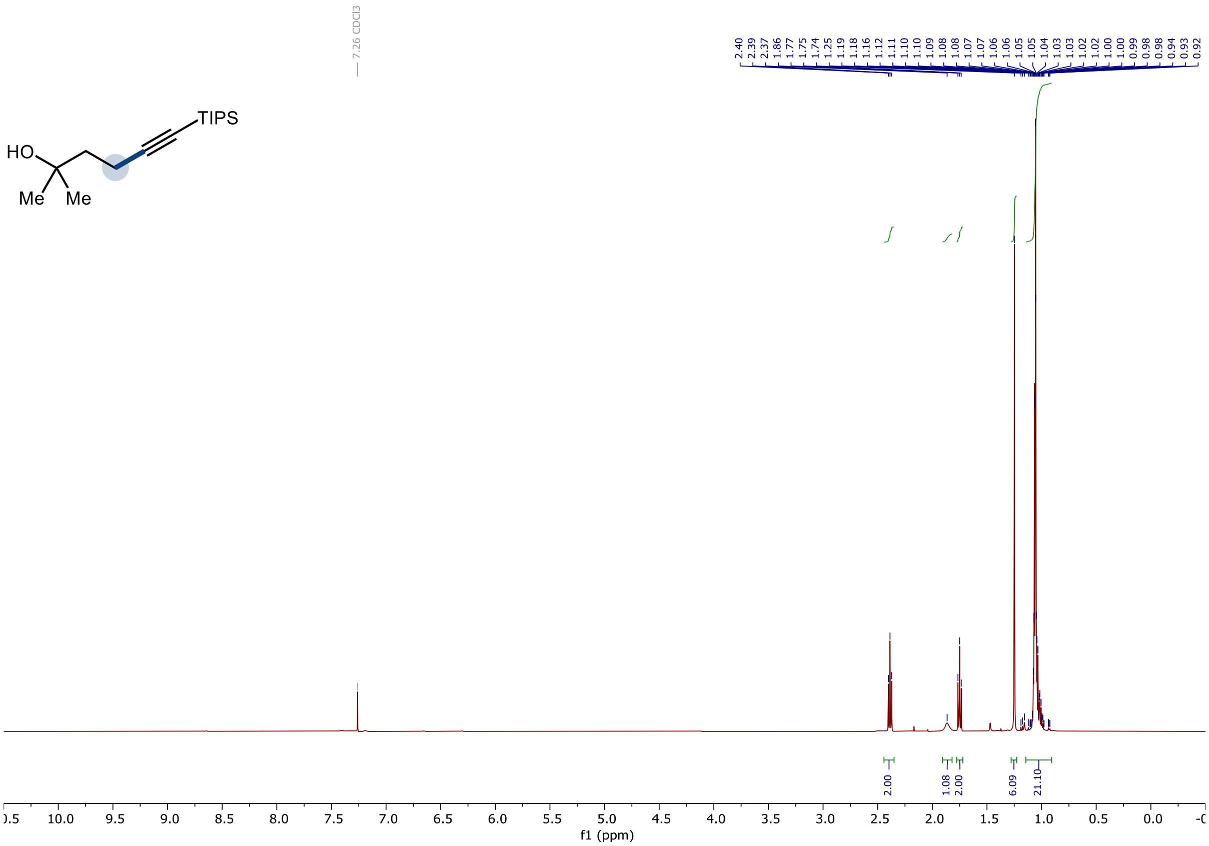
TIPS
HO
Me Me
7.26 CDCl3
2.40
2.39
2.37
1.86
1.77
1.75
1.74
1.25
1.19
1.18
1.16
1.12
1.11
1.10
1.10
1.09
1.08
1.08
1.07
1.07
1.06
1.06
1.05
1.05
1.04
1.03
1.03
1.02
1.02
1.00
1.00
0.99
0.98
0.98
0.94
0.93
0.92
2.00
1.08
2.00
6.09
21.10
f1 (ppm)

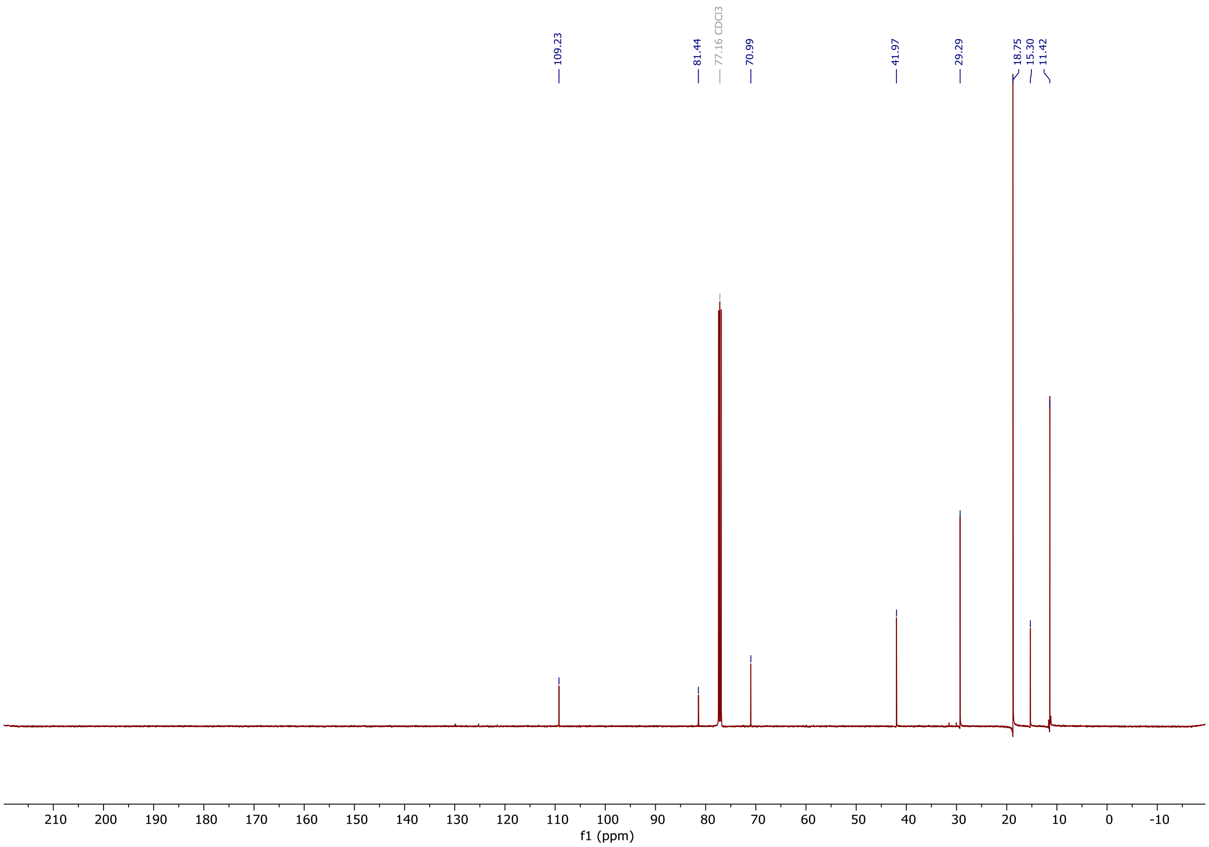
109.23
81.44
77.16 CDCl3
70.99
41.97
29.29
18.75
15.30
11.42
f1 (ppm)

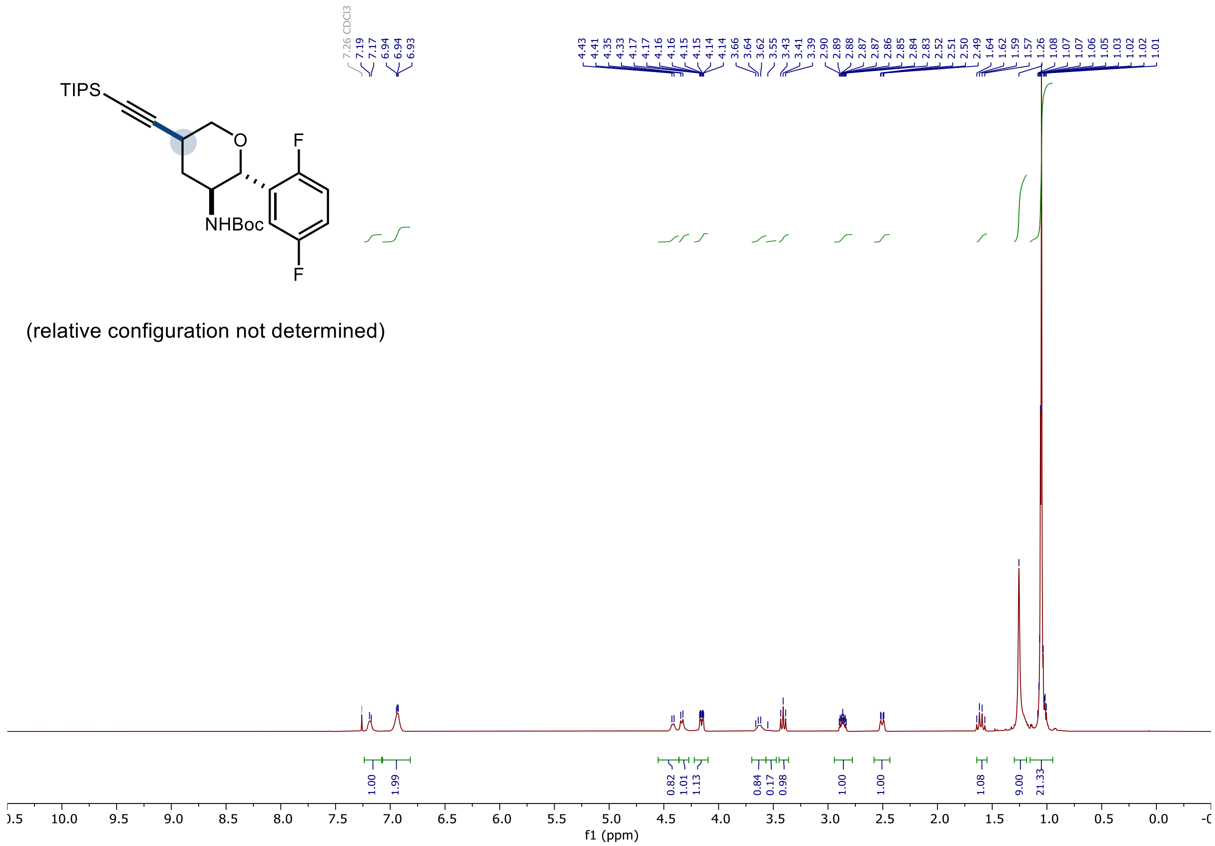

(relative configuration not determined)

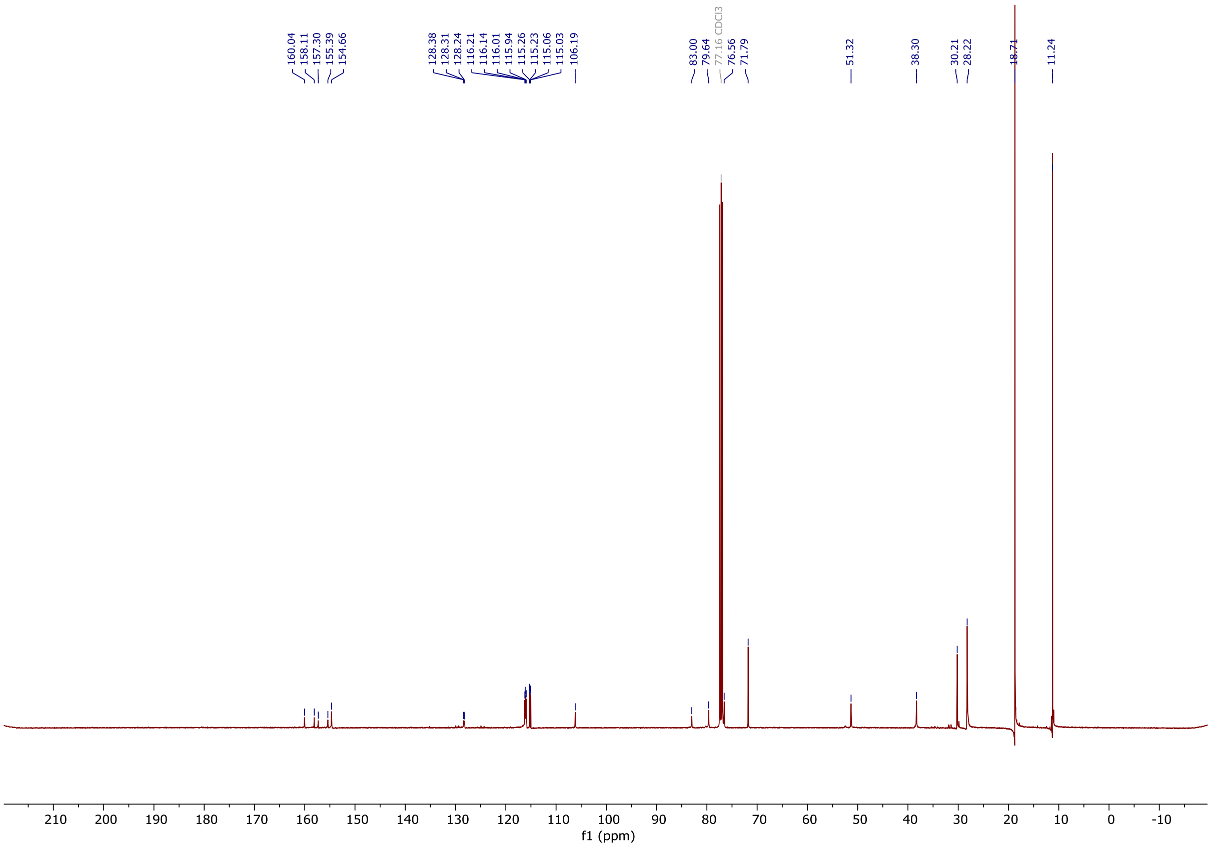

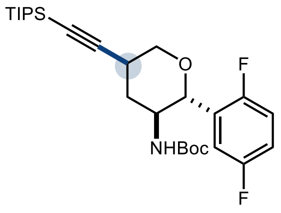

(relative configuration not determined)

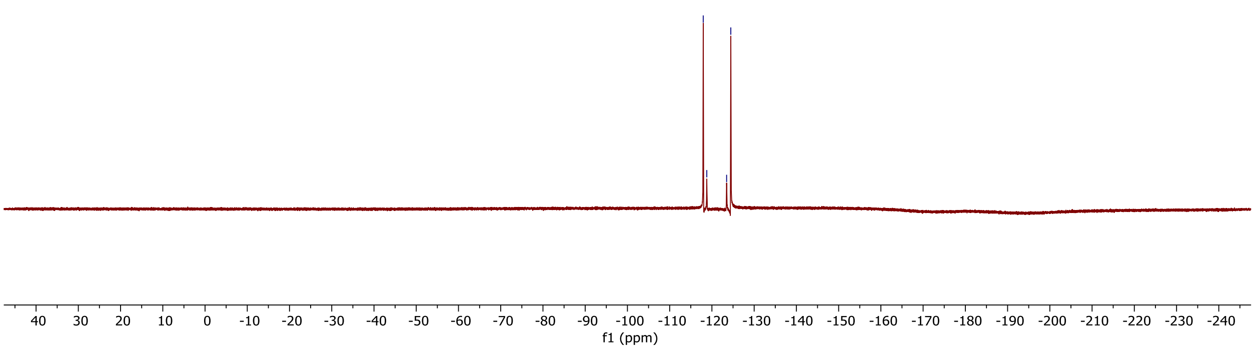

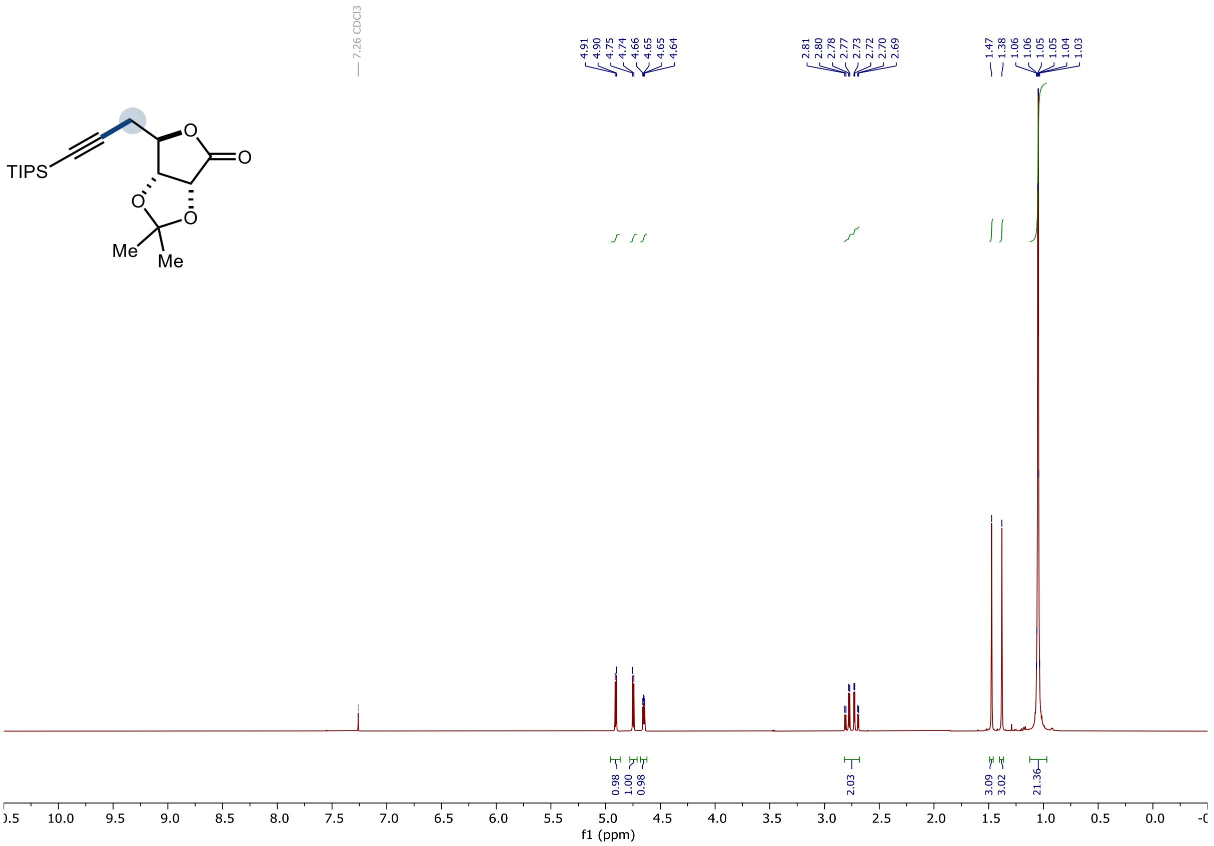
TIPS
O
O
O
O
Me
Me
7.26 CDCl3
4.91
4.90
4.75
4.74
4.66
4.65
4.65
4.64
2.81
2.80
2.78
2.77
2.73
2.72
2.70
2.69
1.47
1.38
1.06
1.06
1.05
1.05
1.04
1.03
0.98
1.00
0.98
2.03
3.09
3.02
21.36
f1 (ppm)

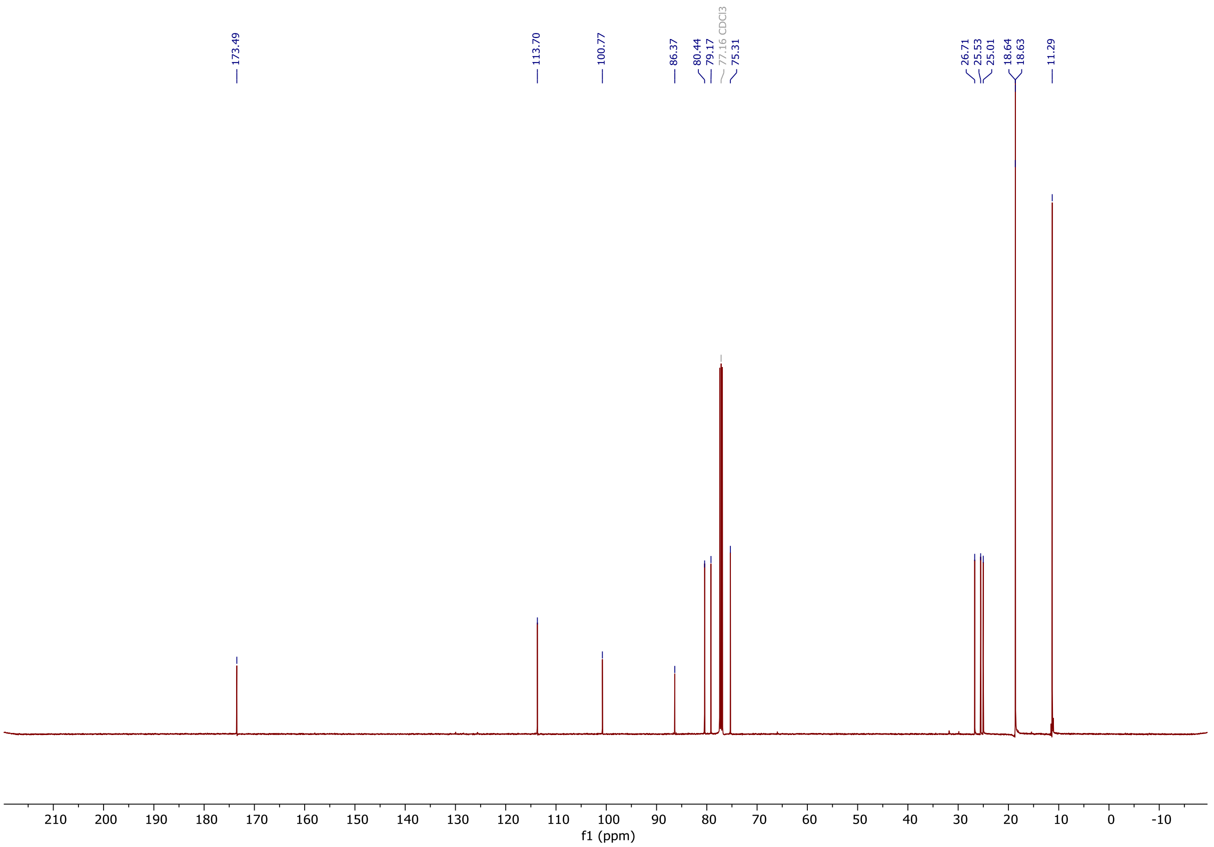
173.49
113.70
100.77
86.37
80.44
79.17
77.16 CDCl3
75.31
26.71
25.53
25.01
18.64
18.63
11.29
f1 (ppm)

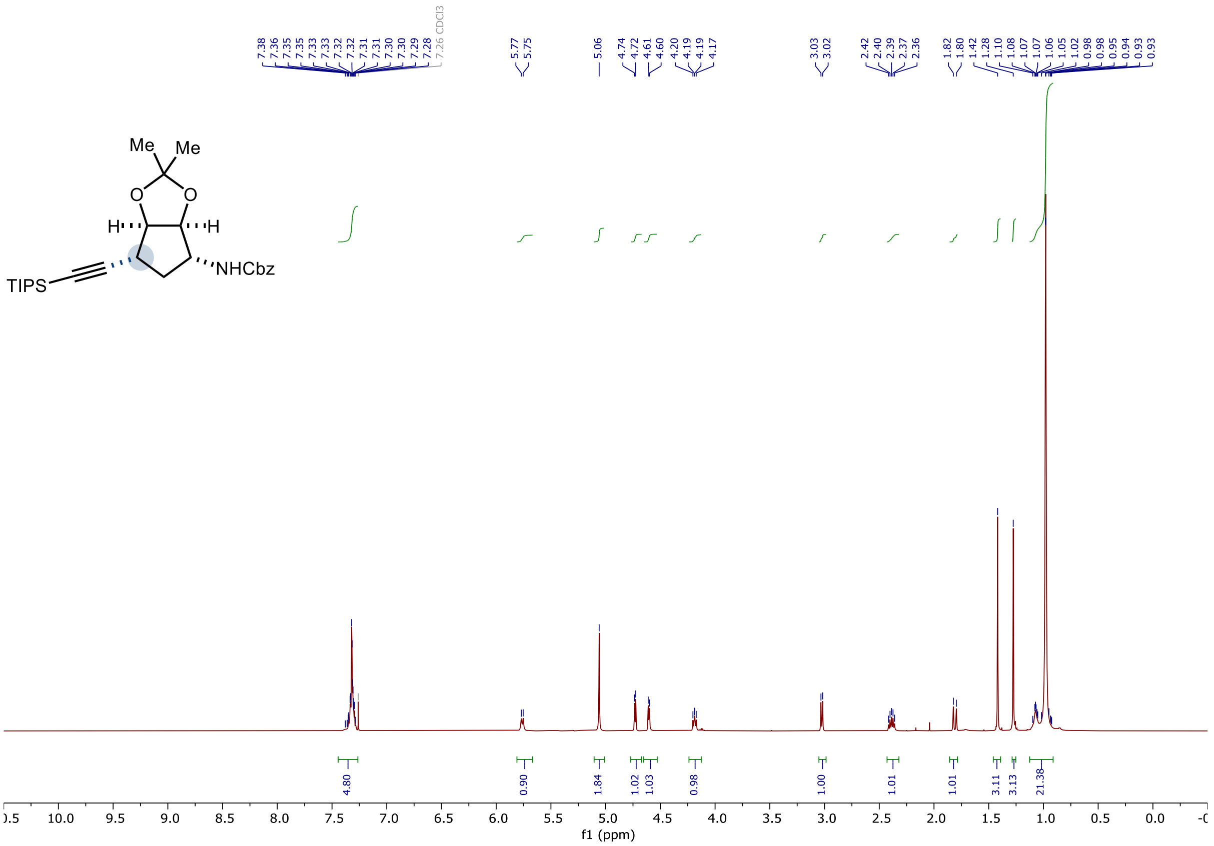

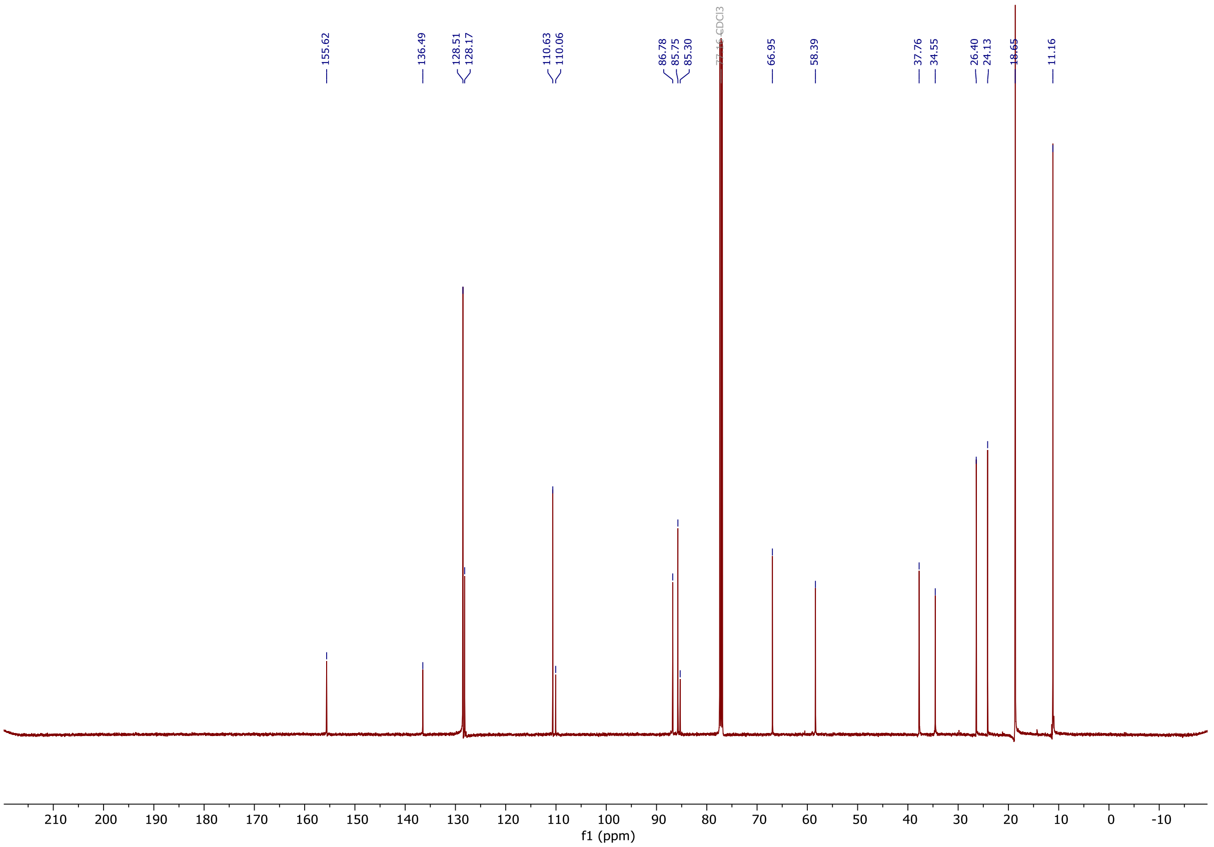

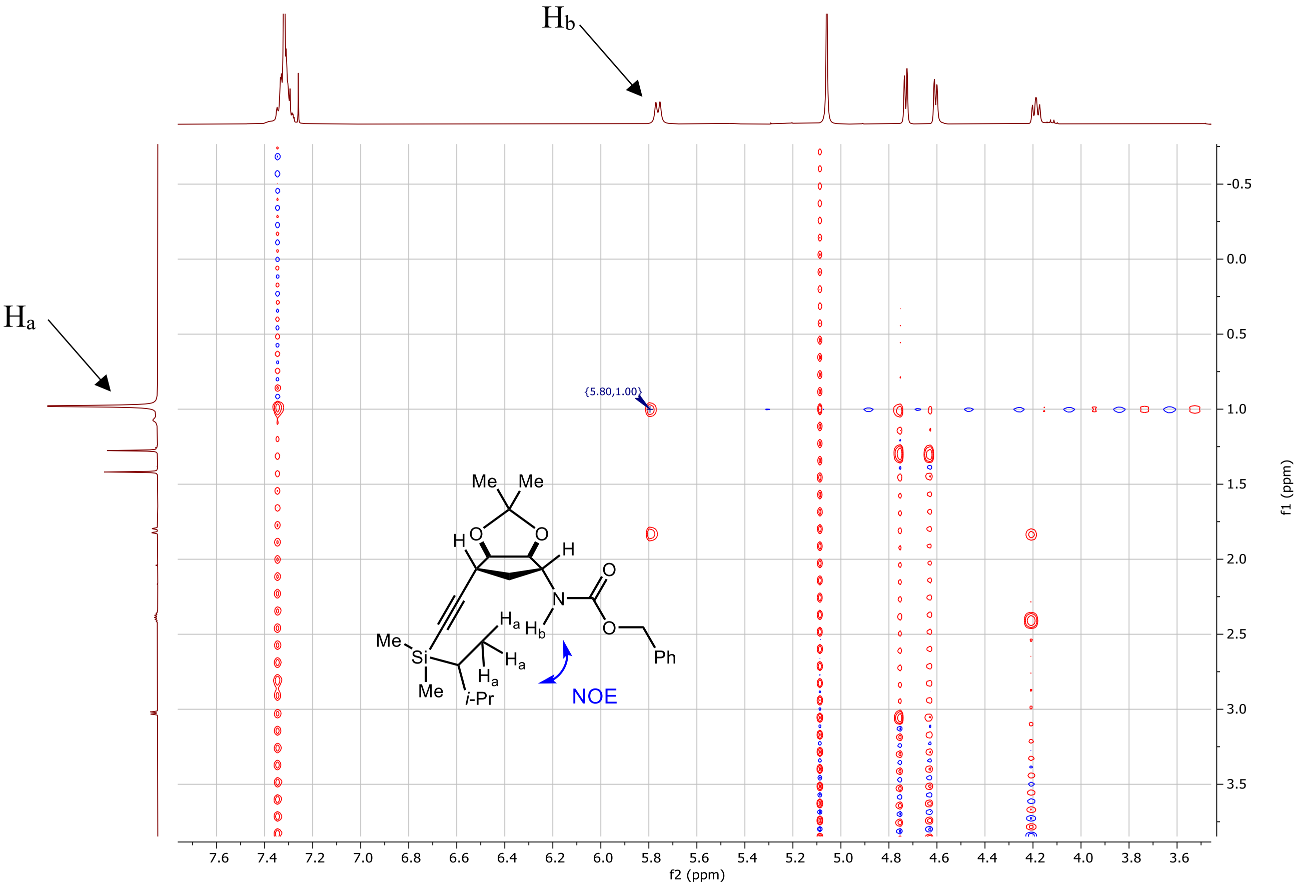

H_a-H_b with NOESY

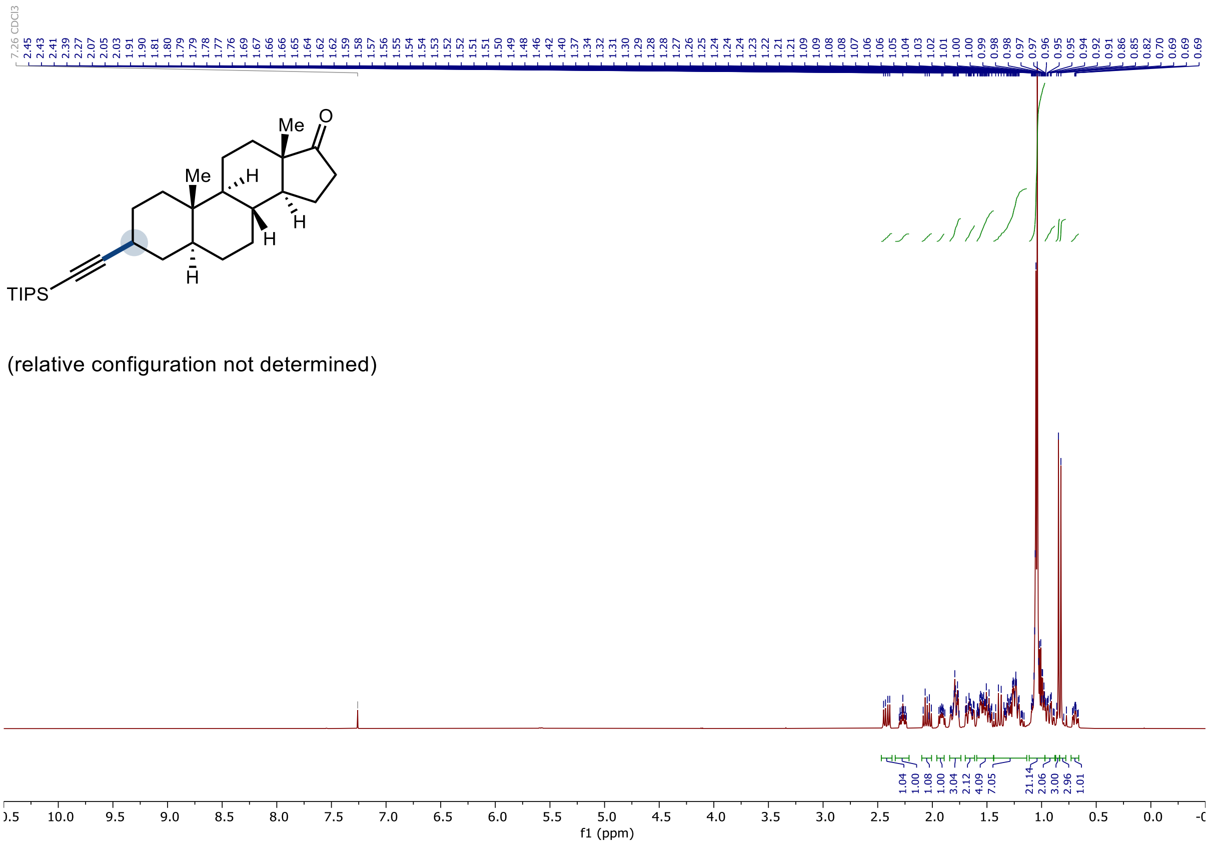

(relative configuration not determined)

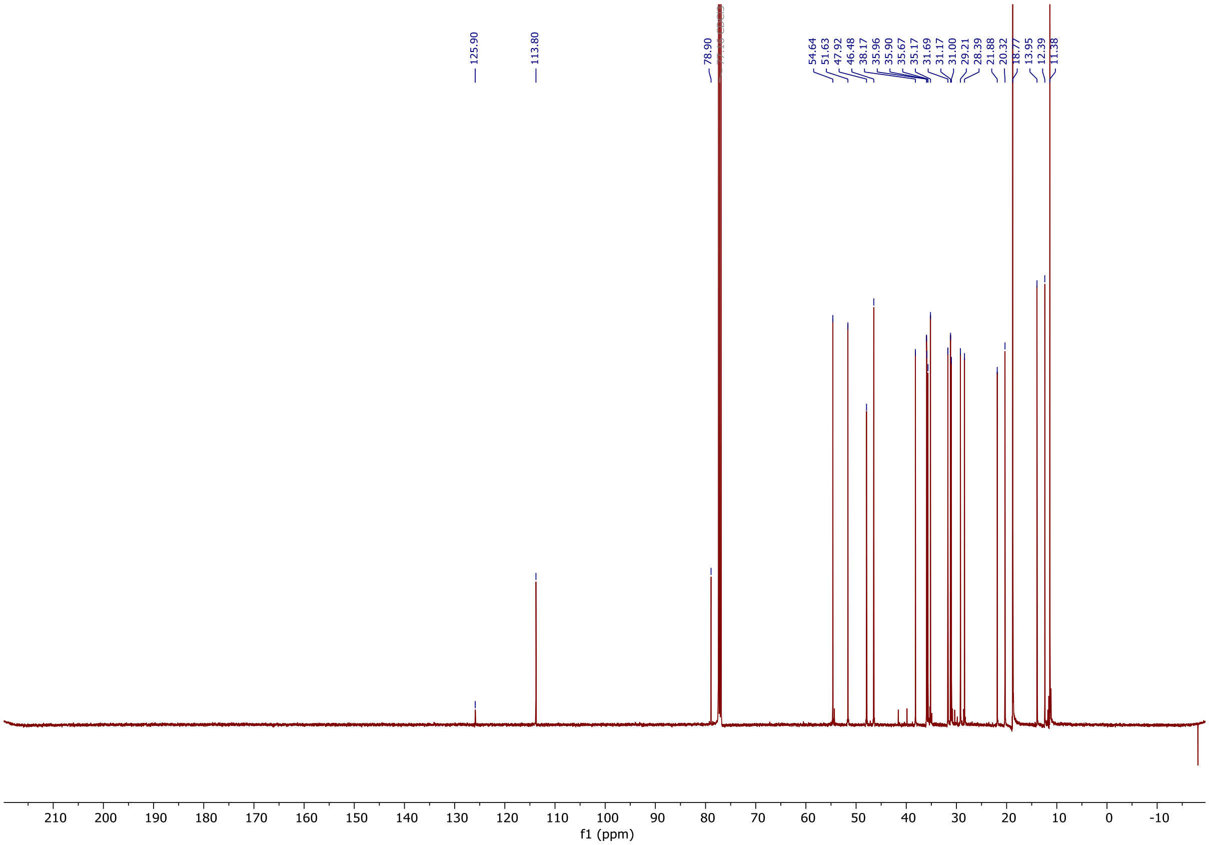

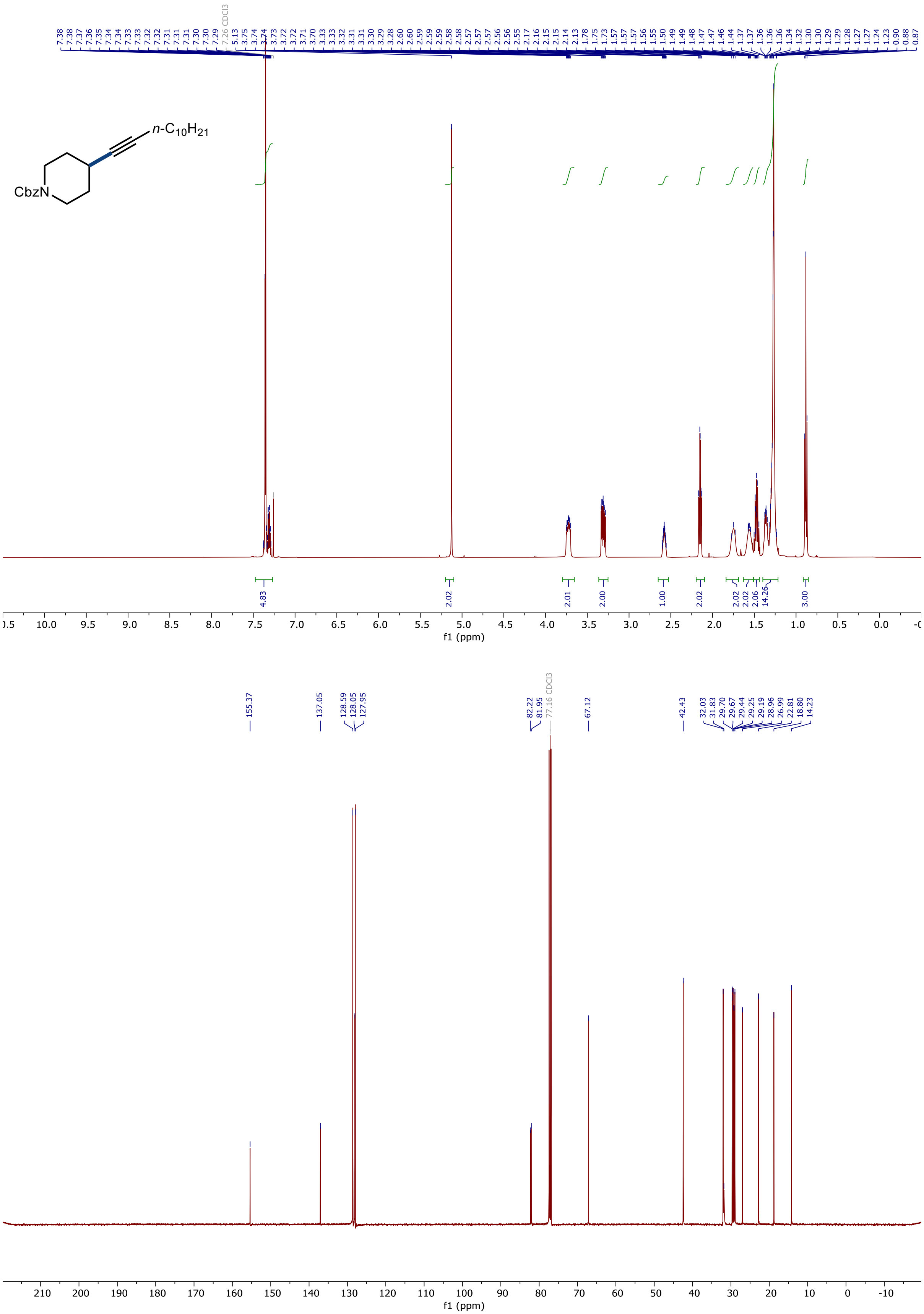

n-C10H21
CbzN
f1 (ppm)
f1 (ppm)

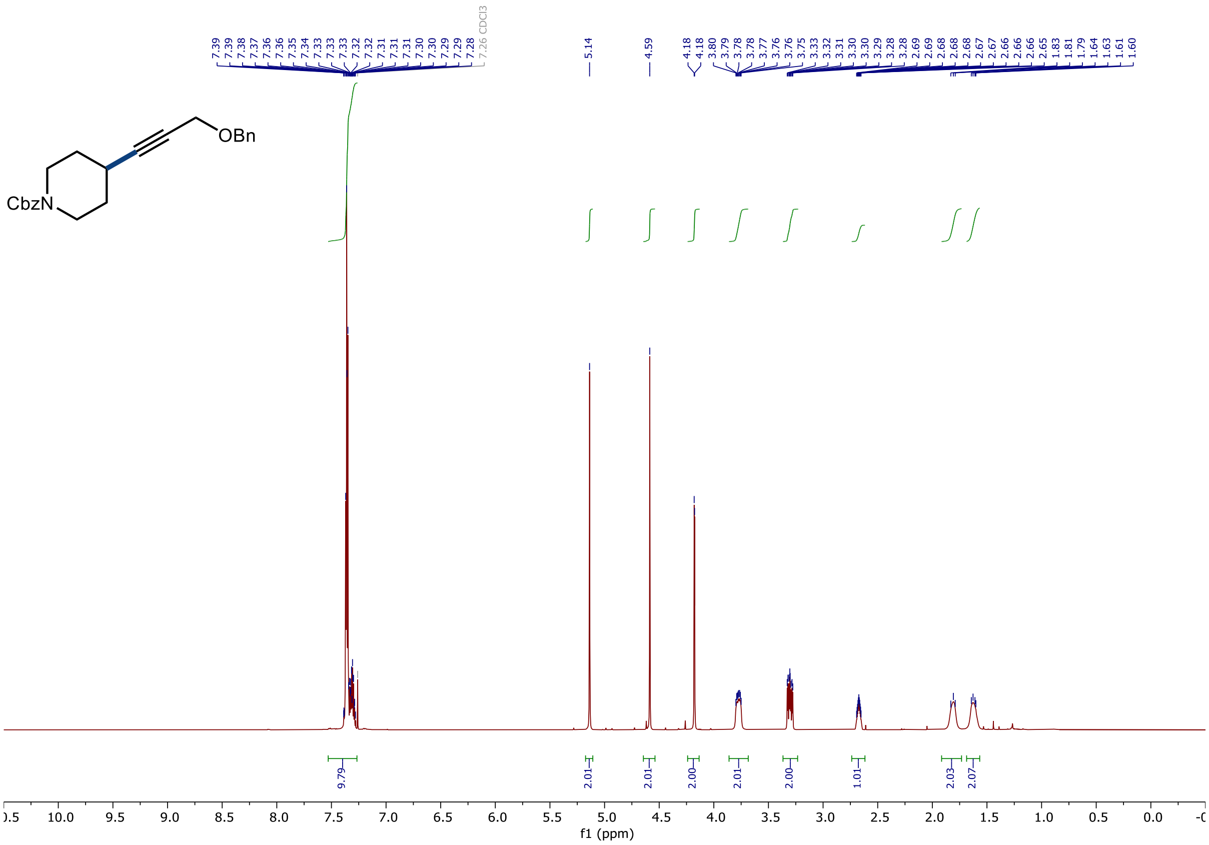
OBn
CbzN
7.39 7.39 7.38 7.37 7.36 7.36 7.35 7.34 7.33 7.33 7.33 7.32 7.32 7.31 7.31 7.30 7.30 7.29 7.29 7.28
7.26 CDCl3
5.14
4.59
4.18 4.18 3.80 3.79 3.78 3.78 3.77 3.76 3.76 3.75 3.33 3.32 3.31 3.30 3.30 3.29 3.28 3.28 2.69 2.69 2.68 2.68 2.67 2.67 2.66 2.66 2.65 1.83 1.81 1.79 1.64 1.63 1.61 1.60
9.79
2.01
2.01
2.00
2.01
2.00
1.01
2.03
2.07
f1 (ppm)

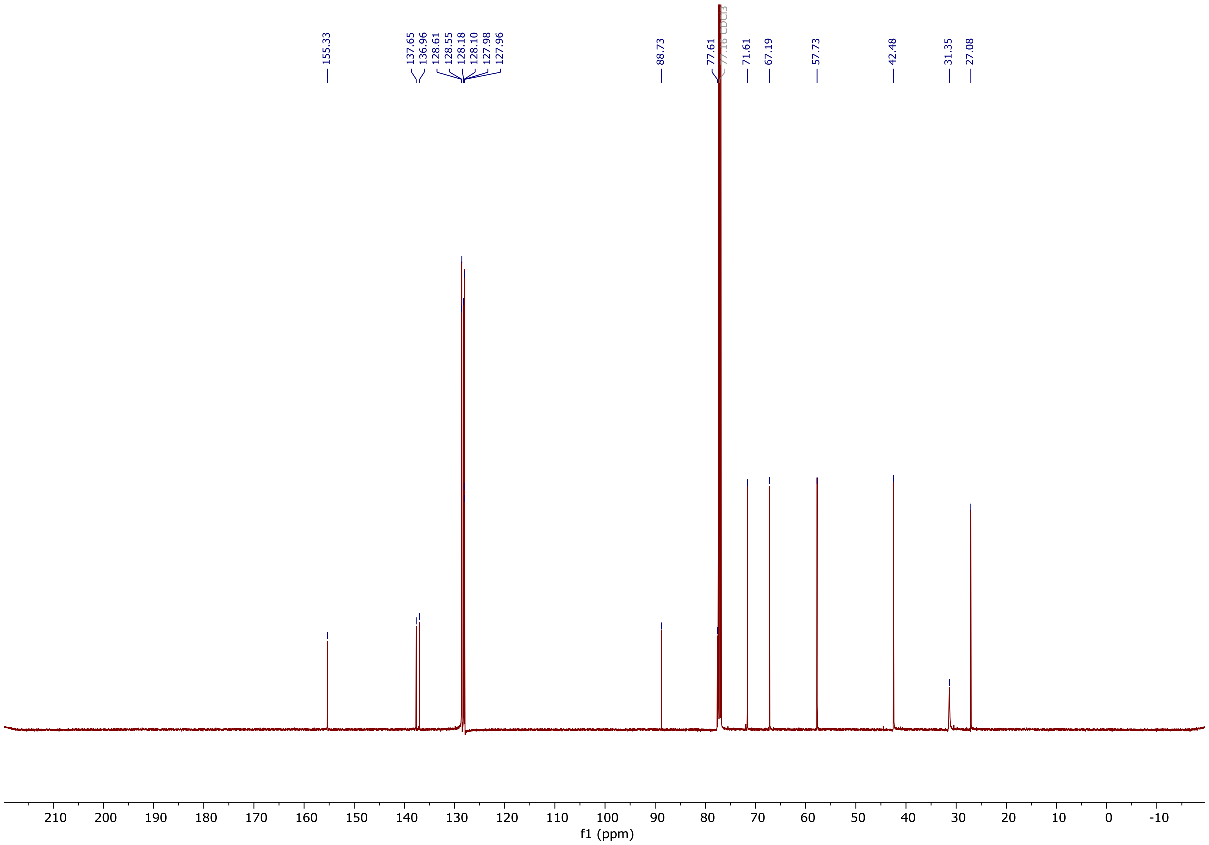
155.33
137.65 136.96 128.61 128.55 128.18 128.10 127.98 127.96
88.73
77.61
77.16 CDCl3
71.61
67.19
57.73
42.48
31.35
27.08
f1 (ppm)

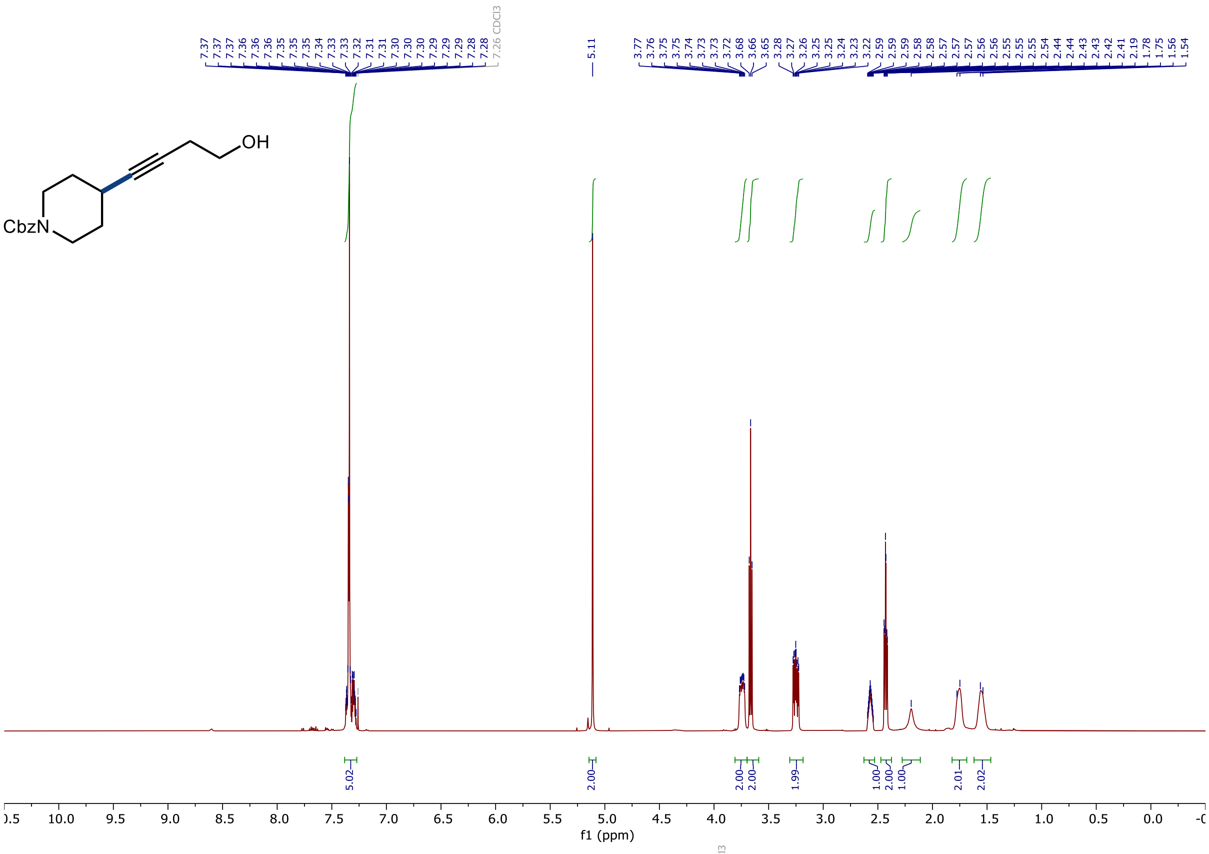
OH
CbzN
7.37 7.37 7.37 7.36 7.36 7.35 7.35 7.35 7.34 7.33 7.33 7.32 7.31 7.31 7.30 7.30 7.29 7.29 7.29 7.28 7.28 7.26 CDCl3
5.11
3.77 3.76 3.75 3.75 3.74 3.73 3.73 3.72 3.68 3.66 3.65 3.28 3.27 3.26 3.25 3.25 3.24 3.23 3.22 2.59 2.59 2.59 2.59 2.58 2.58 2.57 2.57 2.57 2.56 2.56 2.55 2.55 2.55 2.54 2.44 2.44 2.43 2.42 2.41 2.19 1.78 1.56 1.54
5.02
2.00
2.00
2.00
1.99
1.00
2.00
1.00
2.01
2.02
f1 (ppm)

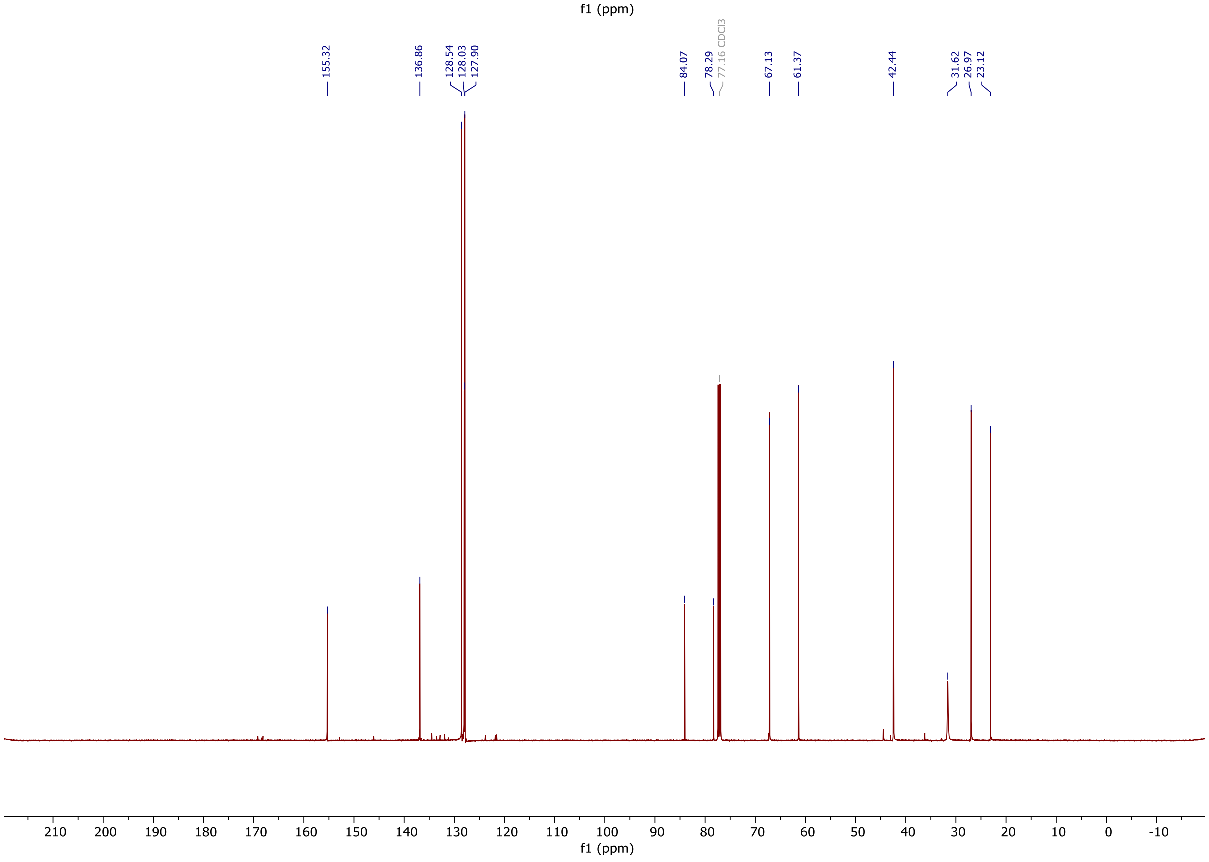
155.32
136.86
128.54
128.03
127.90
84.07
78.29
77.16 CDCl3
67.13
61.37
42.44
31.62
26.97
23.12
f1 (ppm)

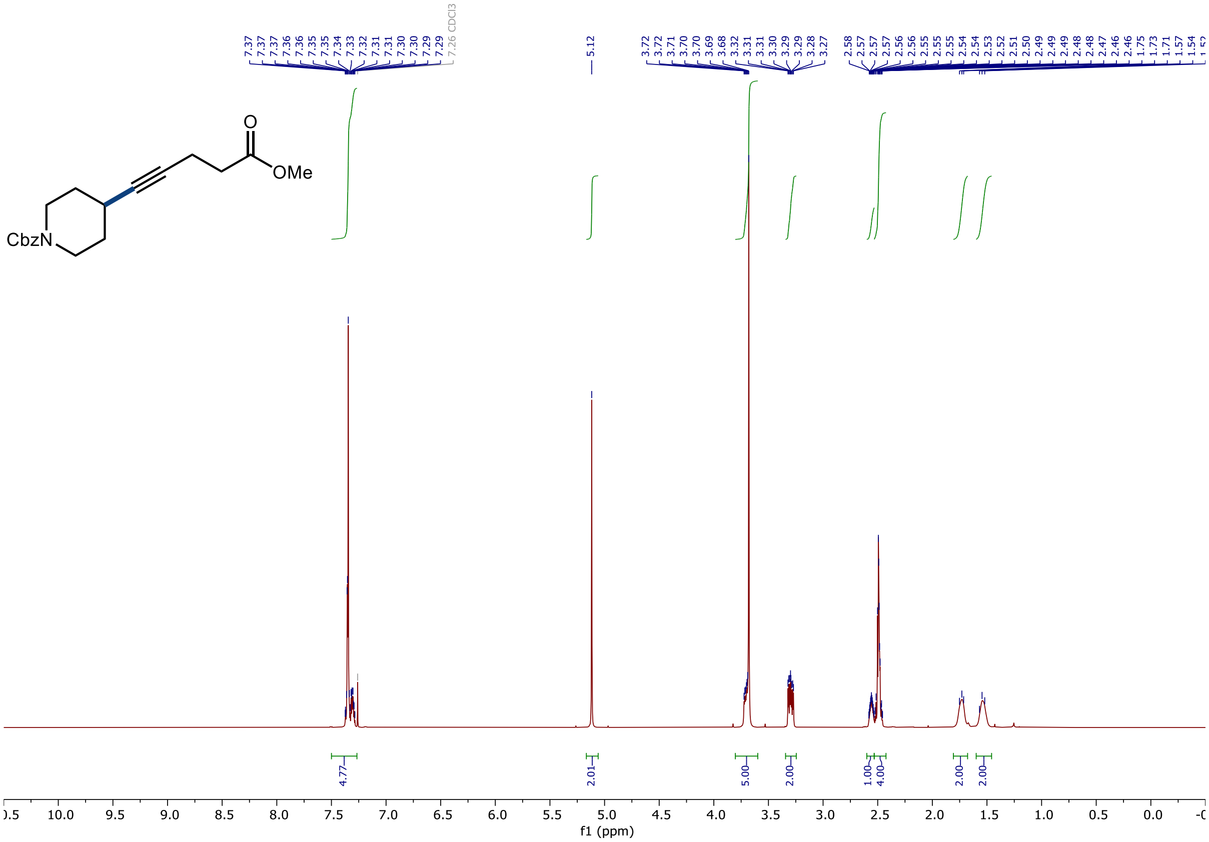
O
OMe
CbzN
7.26 CDCl3
5.12
4.77
2.01
5.00
2.00
1.00
4.00
2.00
2.00
f1 (ppm)

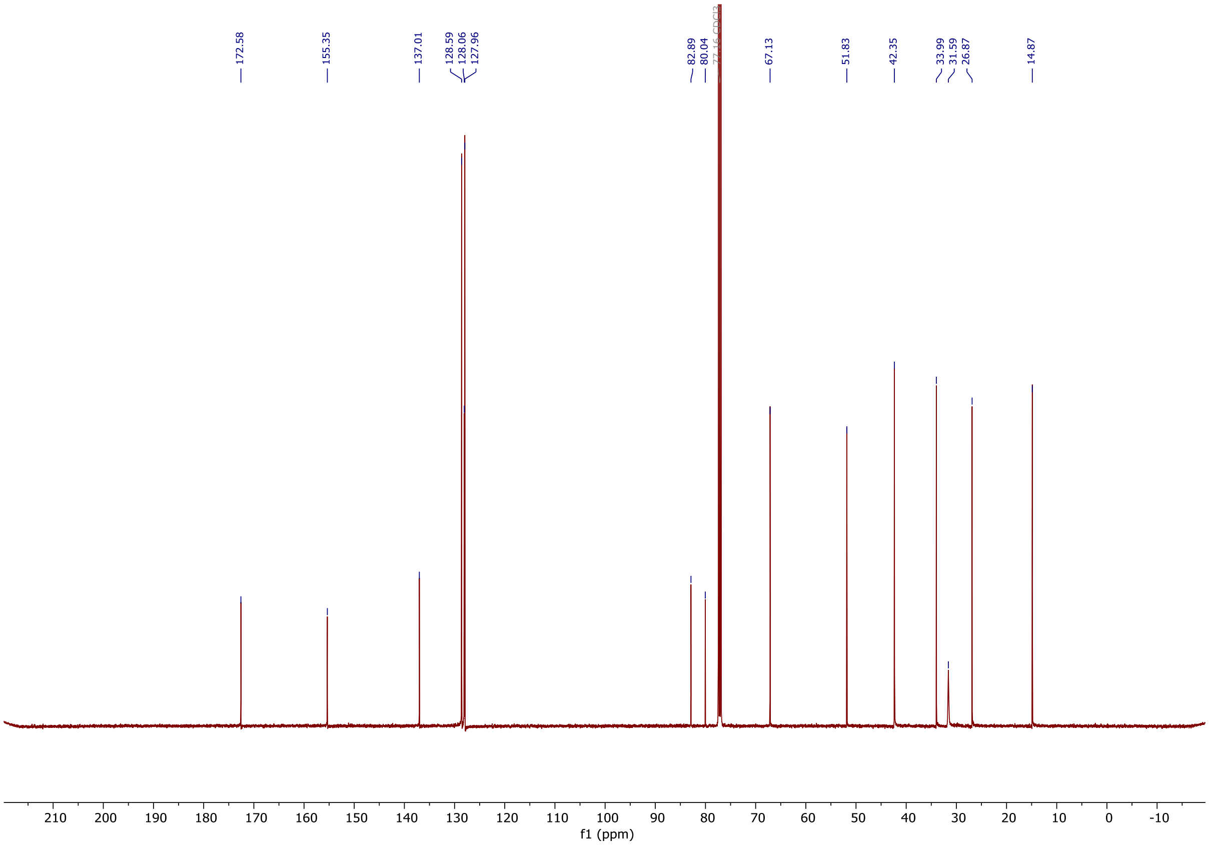
172.58
155.35
137.01
128.59
128.06
127.96
82.89
80.04
67.13
51.83
42.35
33.99
31.59
26.87
14.87
f1 (ppm)

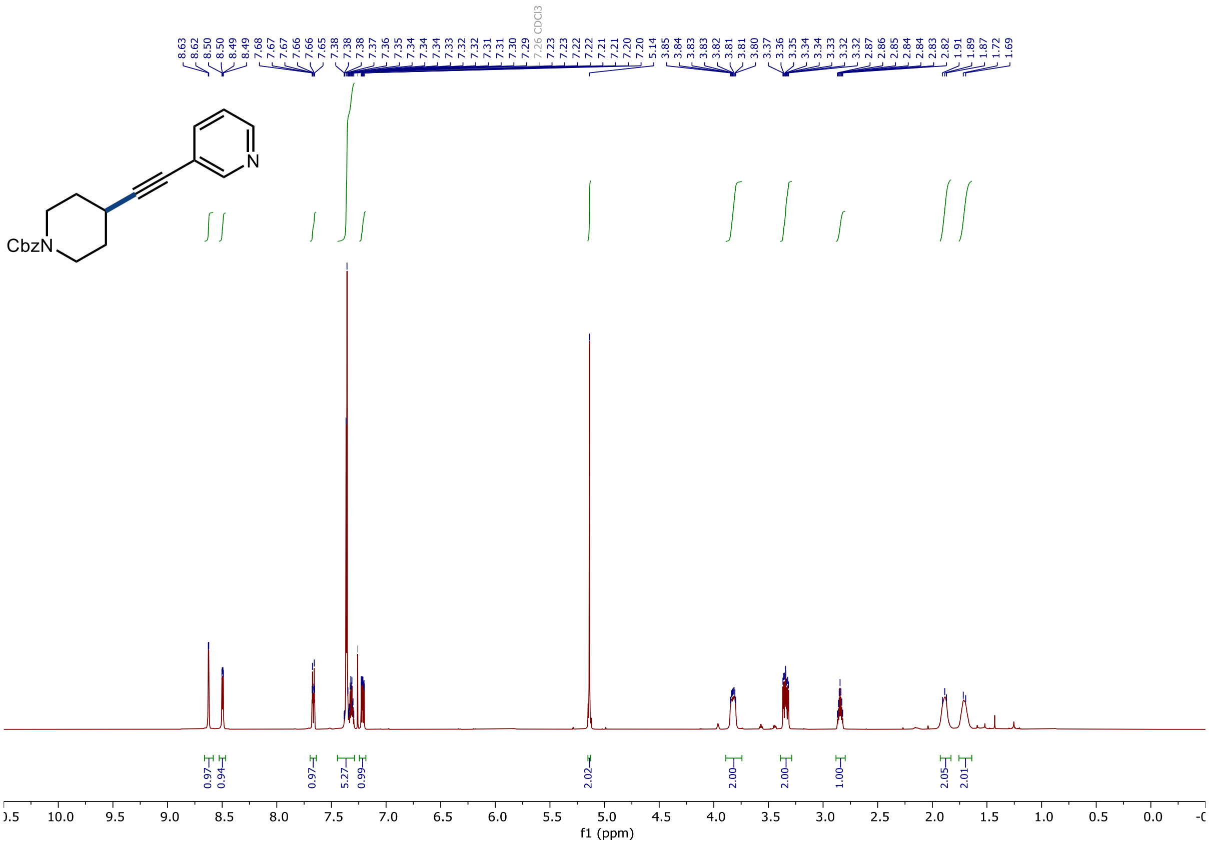

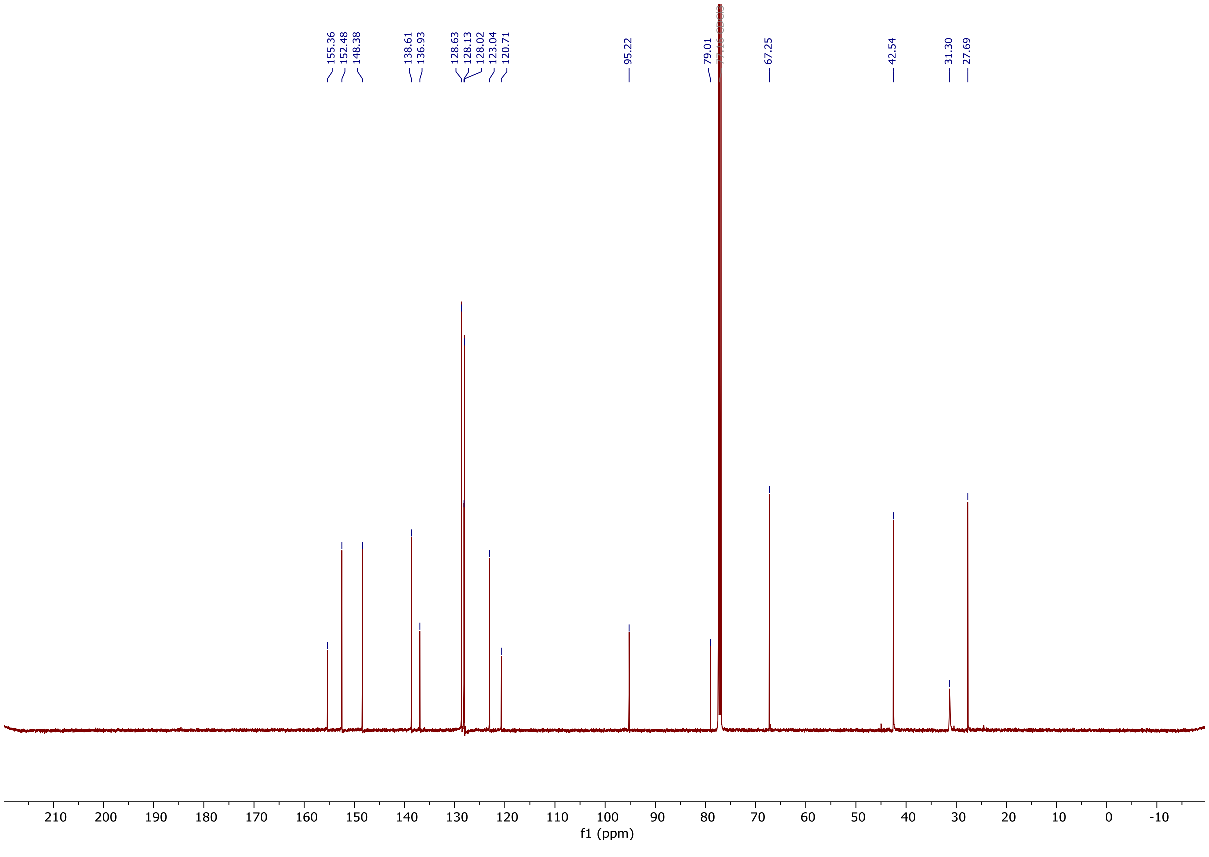

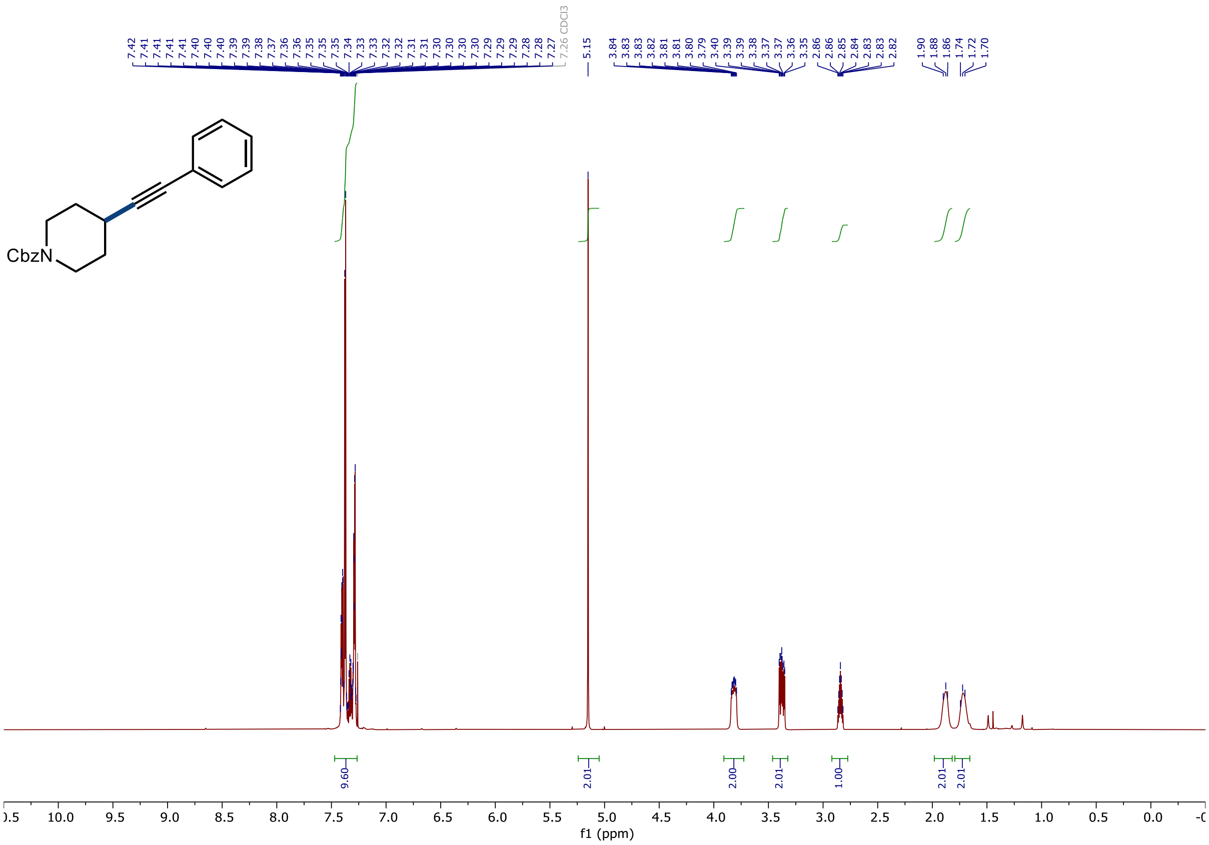
7.42 7.41 7.41 7.41 7.40 7.40 7.39 7.39 7.38 7.37 7.36 7.35 7.35 7.34 7.33 7.33 7.32 7.32 7.31 7.31 7.30 7.30 7.29 7.29 7.28 7.28 7.27
7.26 CDCl3
5.15
3.84 3.83 3.83 3.82 3.81 3.80 3.79 3.40 3.39 3.39 3.38 3.37 3.37 3.36 3.35
2.86 2.86 2.85 2.84 2.83 2.83 2.82
1.90 1.88 1.86 1.74 1.72 1.70
CbzN
9.60
2.01
2.00
2.01
1.00
2.01
2.01
f1 (ppm)

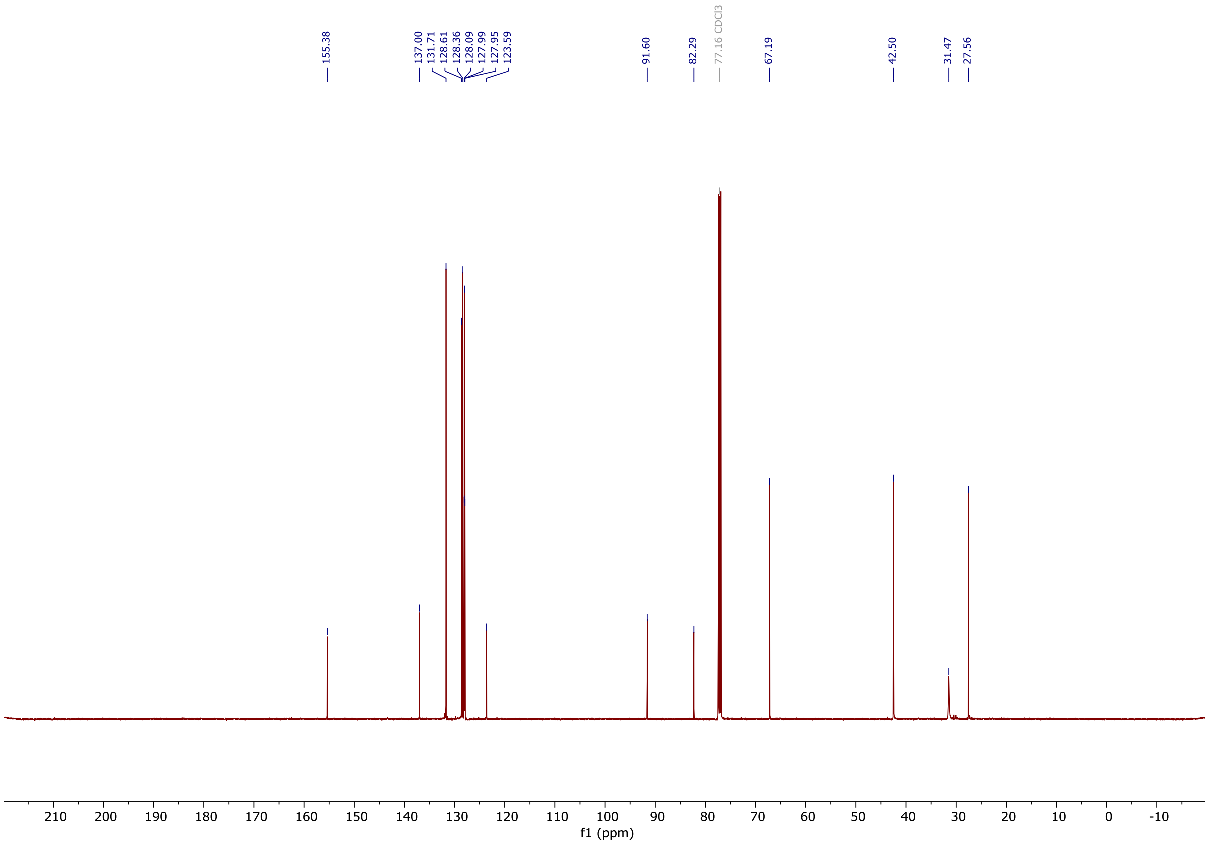
155.38
137.00 131.71 128.61 128.36 128.09 127.99 127.95 123.59
91.60
82.29
77.16 CDCl3
67.19
42.50
31.47
27.56
f1 (ppm)

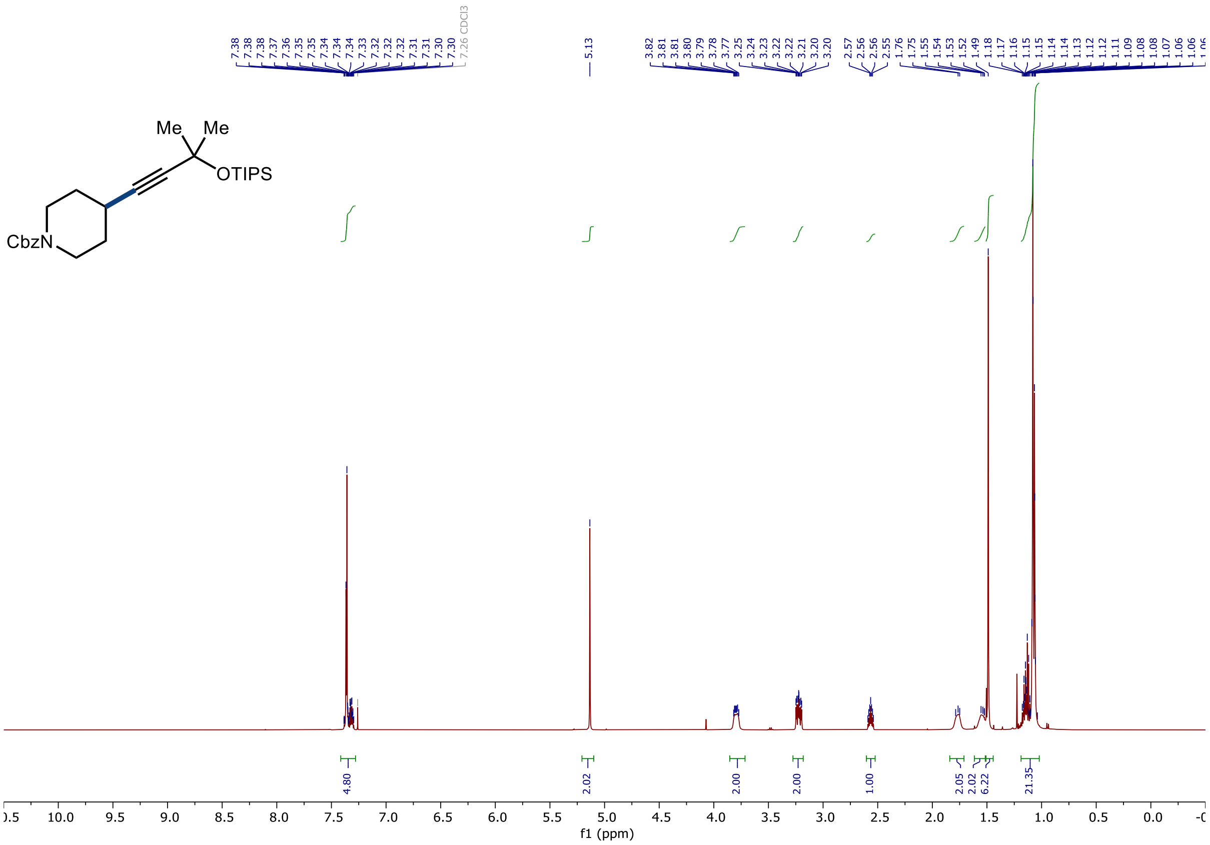
Me
Me
OTIPS
CbzN
f1 (ppm)

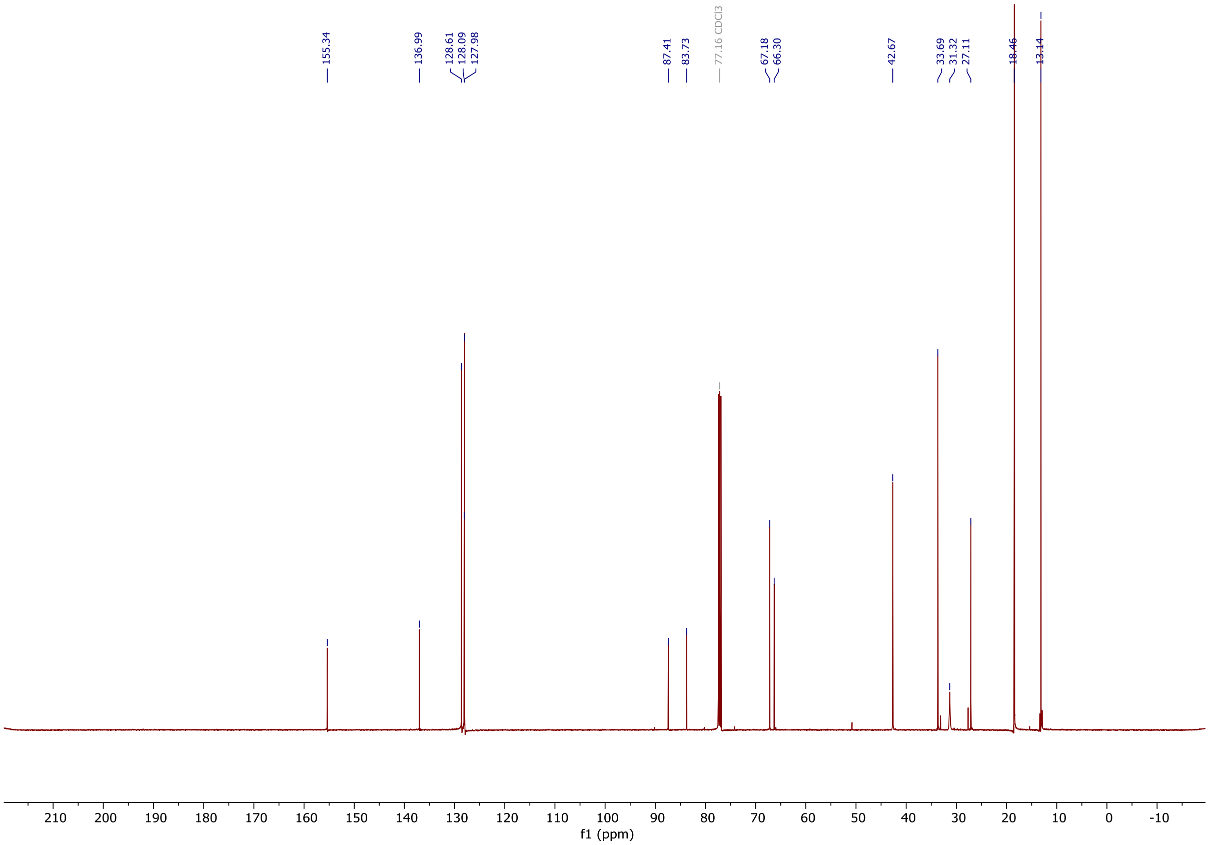
f1 (ppm)

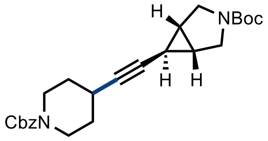
H
NBoc
H
H
CbzN

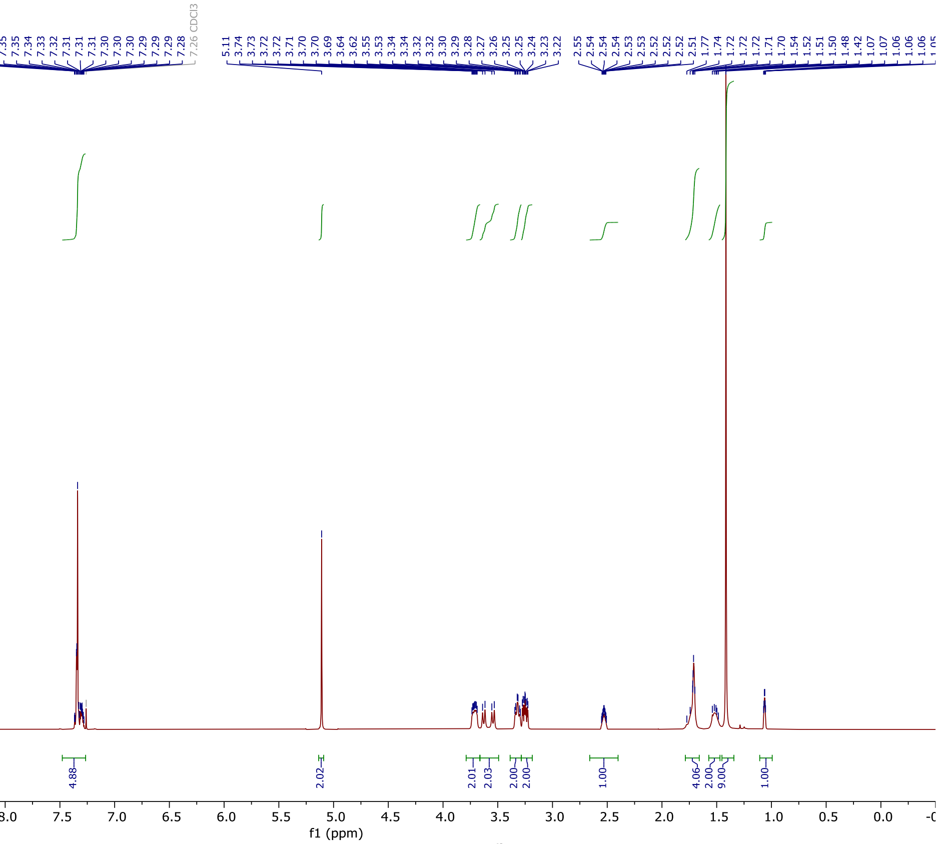
f1 (ppm)

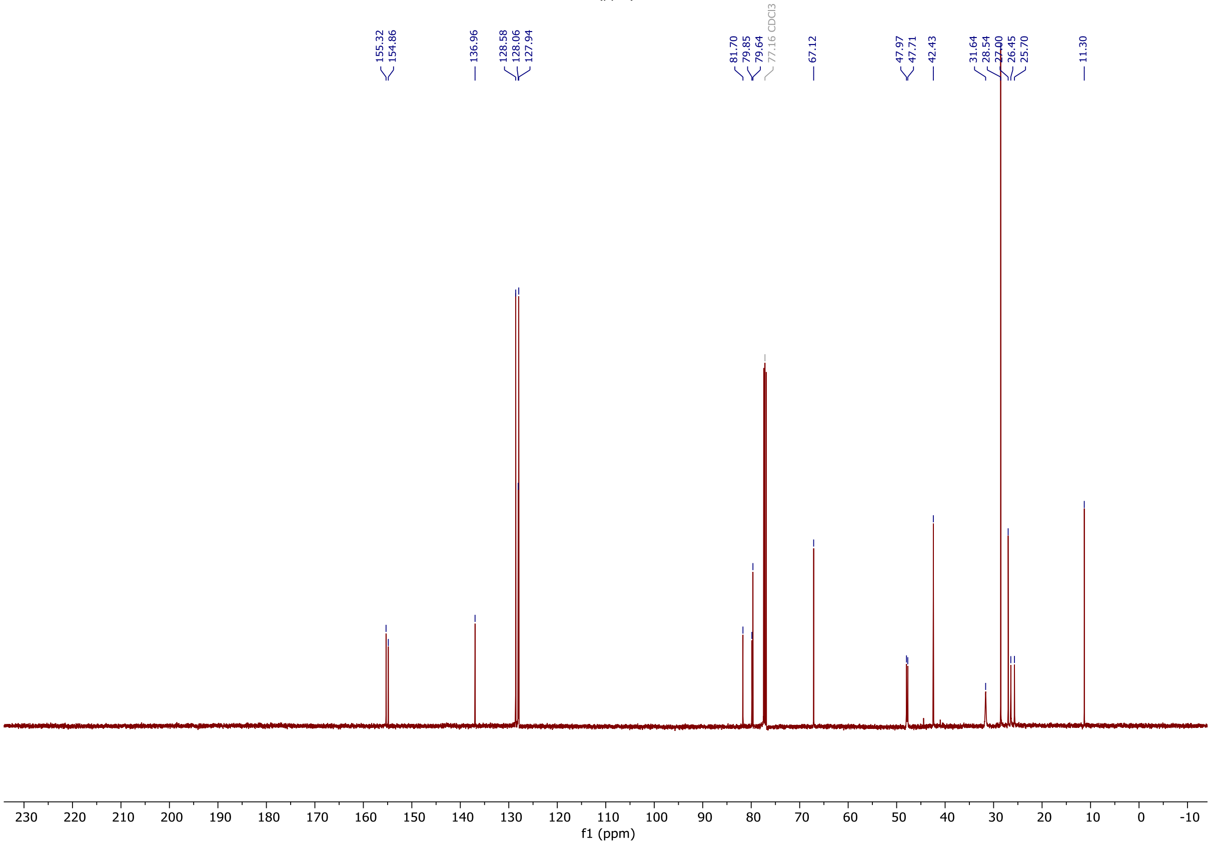
f1 (ppm)

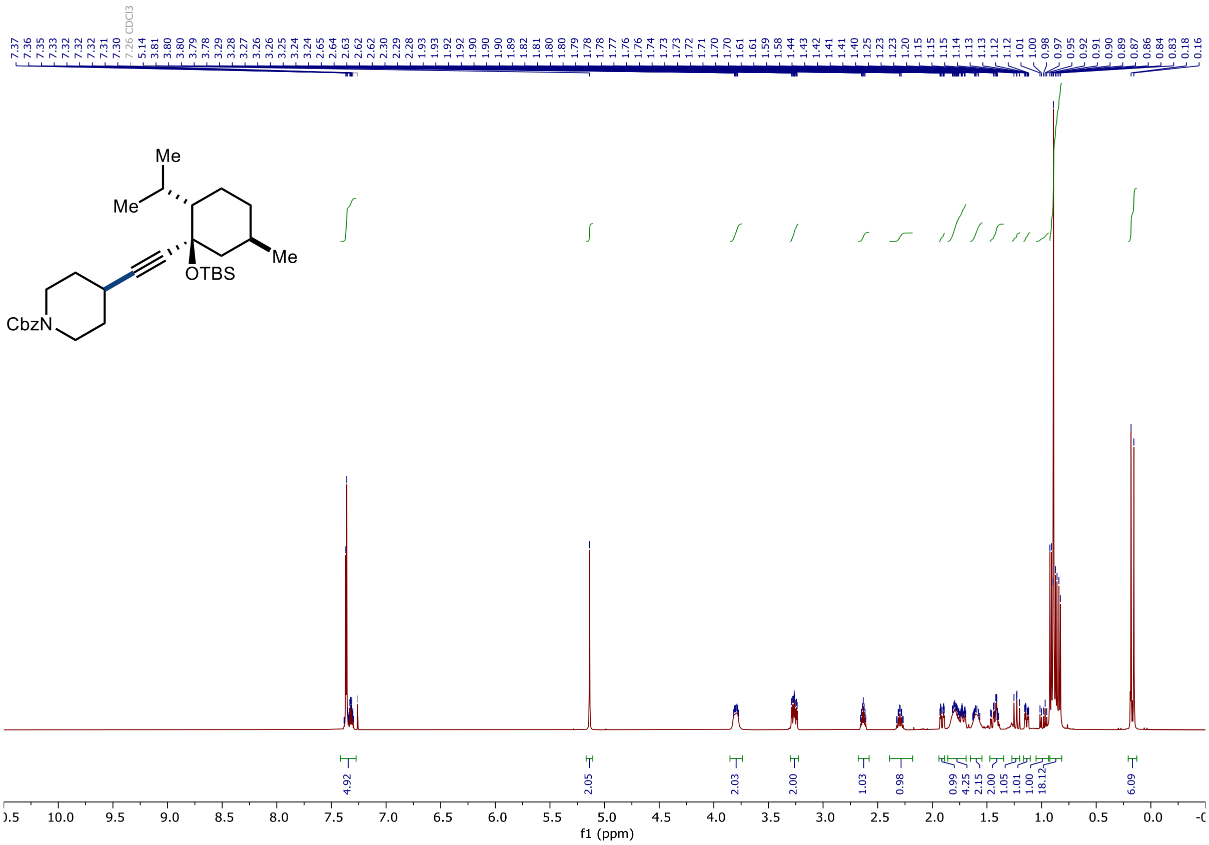
7.37 7.36 7.35 7.33 7.32 7.32 7.31 7.30 7.26 CDCl3 5.14 3.81 3.80 3.80 3.79 3.78 3.29 3.28 3.27 3.26 3.25 3.24 2.65 2.64 2.63 2.62 2.62 2.30 2.29 2.28 1.93 1.93 1.93 1.92 1.90 1.90 1.90 1.89 1.82 1.81 1.80 1.80 1.79 1.78 1.78 1.77 1.76 1.76 1.74 1.73 1.73 1.72 1.71 1.70 1.61 1.61 1.59 1.58 1.44 1.43 1.42 1.41 1.41 1.40 1.25 1.23 1.23 1.20 1.15 1.15 1.14 1.13 1.13 1.12 1.12 1.01 1.00 0.98 0.97 0.95 0.92 0.91 0.90 0.89 0.87 0.86 0.84 0.83 0.18 0.16
Me
Me
OTBS
Me
CbzN
4.92
2.05
2.03
2.00
1.03
0.98
0.99
4.25
2.15
2.00
1.05
1.01
1.00
18.12
6.09
f1 (ppm)

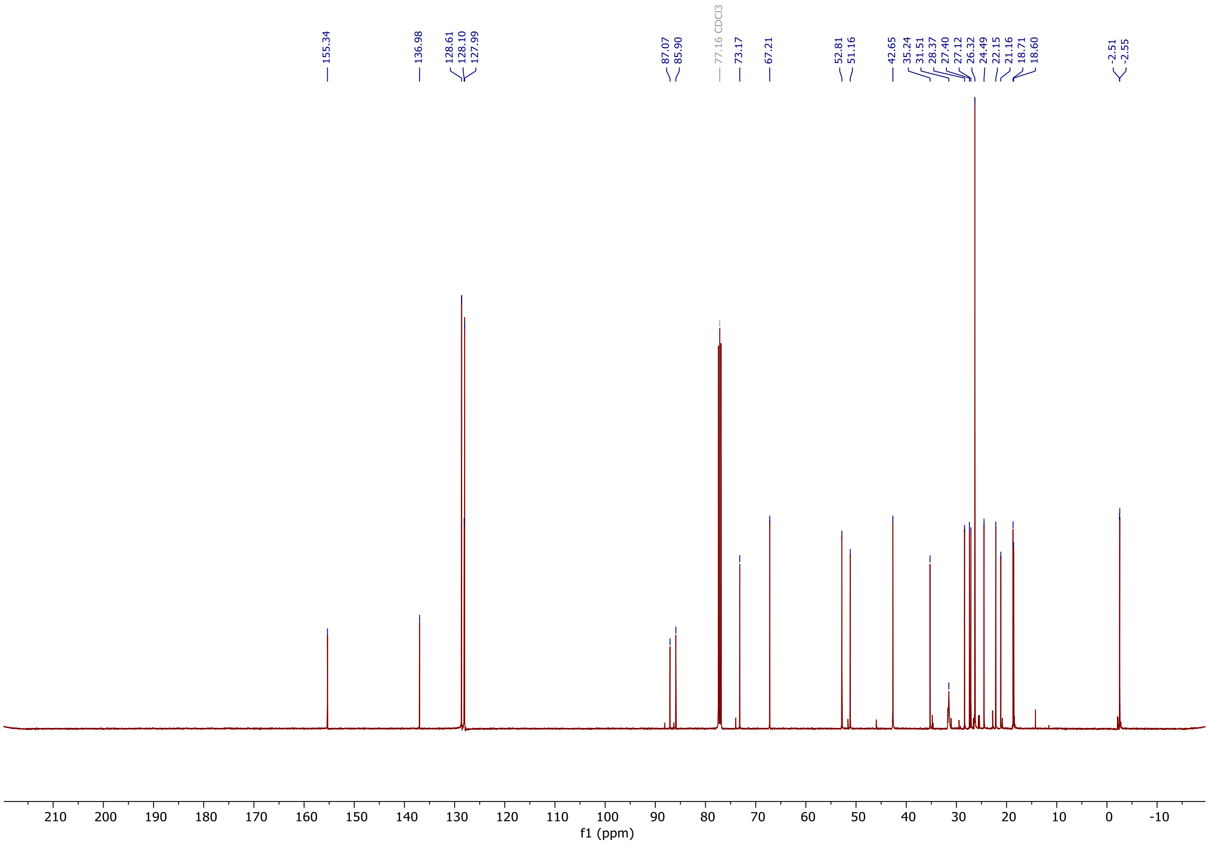
155.34
136.98
128.61 128.10 127.99
87.07 85.90
77.16 CDCl3
73.17
67.21
52.81 51.16
42.65 35.24 31.51 28.37 27.40 27.12 26.32 24.49 22.15 21.16 18.71 18.60
-2.51 -2.55
f1 (ppm)

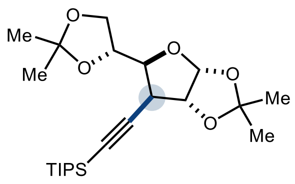
O
Me
Me
O
O
O
O
Me
Me
TIPS

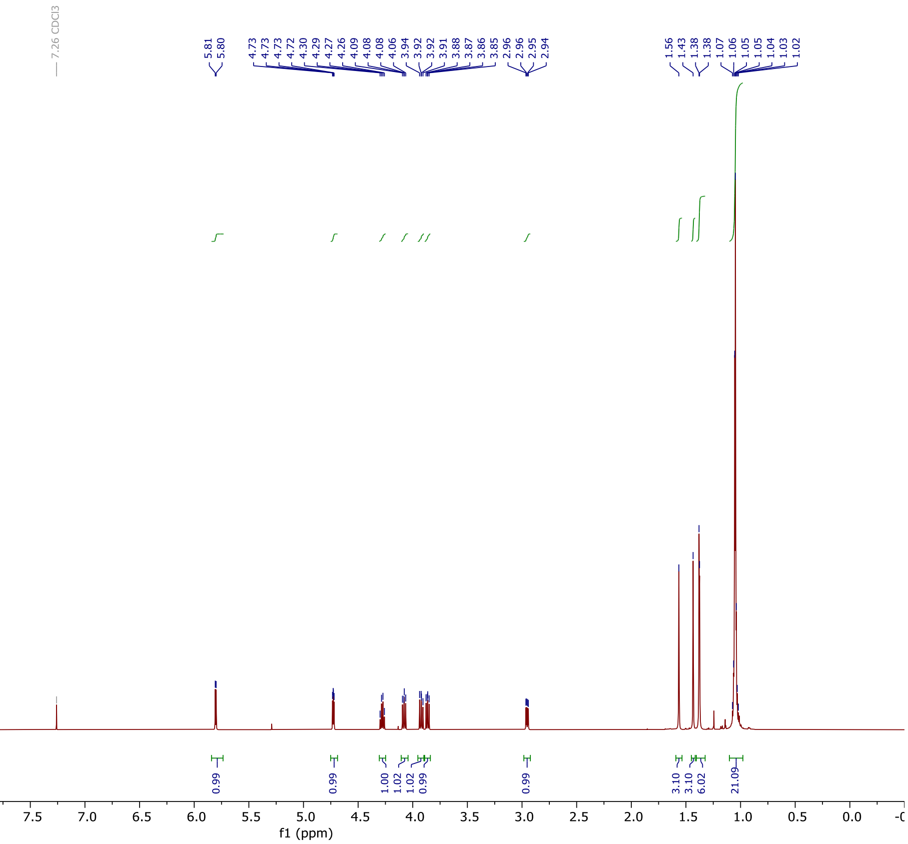
7.26 CDCl3
5.81
5.80
4.73
4.73
4.73
4.72
4.30
4.29
4.27
4.26
4.09
4.08
4.08
4.06
3.94
3.92
3.92
3.91
3.88
3.87
3.86
3.85
2.96
2.96
2.95
2.94
1.56
1.43
1.38
1.38
1.07
1.06
1.05
1.05
1.04
1.03
1.02
0.99
0.99
1.00
1.02
1.02
0.99
0.99
3.10
3.10
6.02
21.09
f1 (ppm)

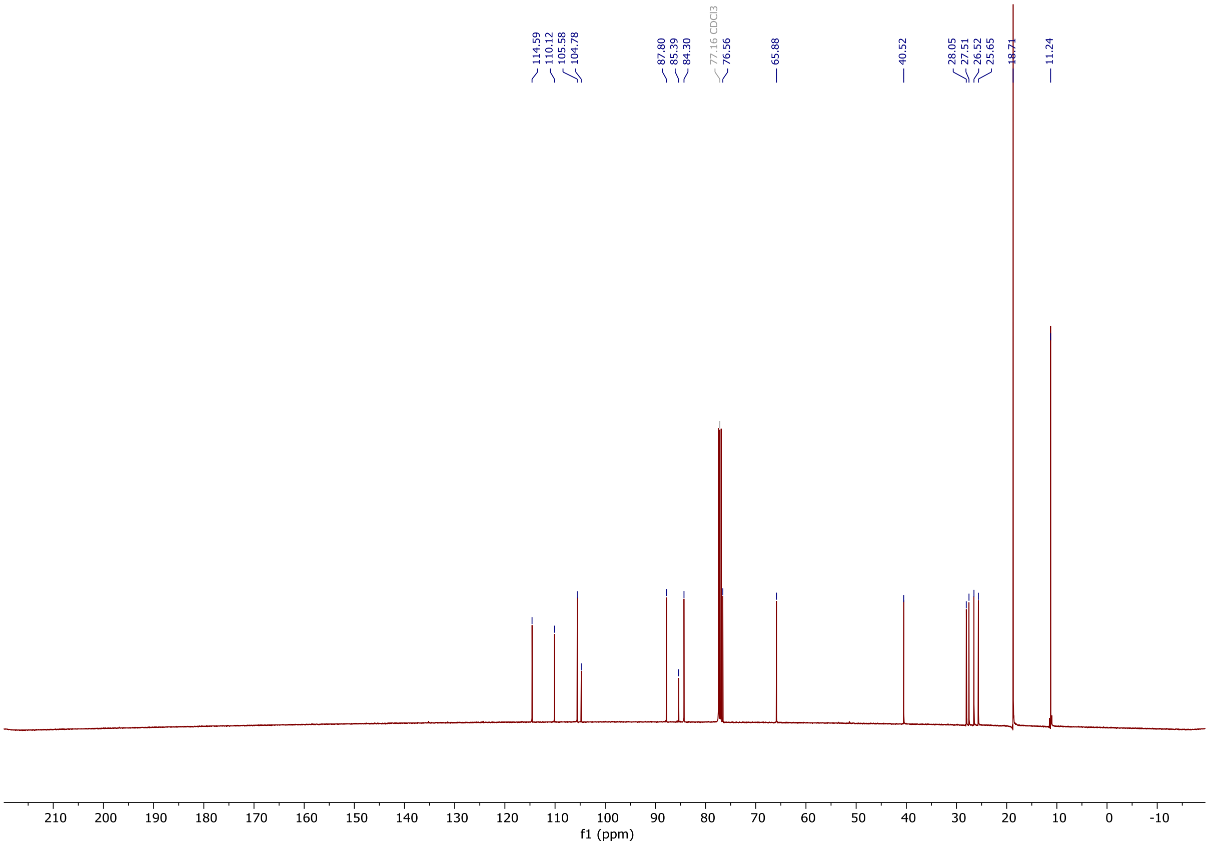
114.59
110.12
105.58
104.78
87.80
85.39
84.30
77.16 CDCl3
76.56
65.88
40.52
28.05
27.51
26.52
25.65
18.71
11.24
f1 (ppm)

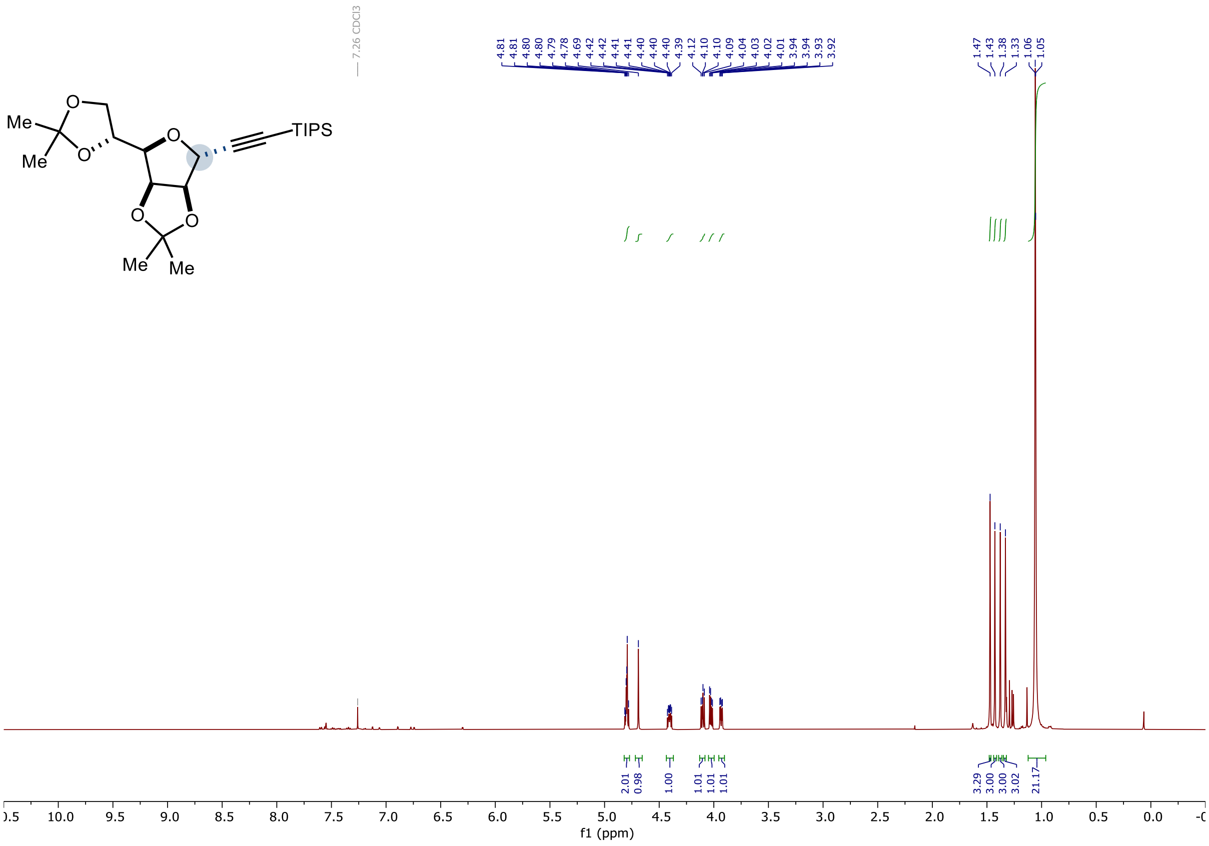
7.26 CDCl3
4.81 4.81 4.80 4.80 4.79 4.78 4.69 4.42 4.42 4.41 4.41 4.40 4.40 4.40 4.39 4.12 4.10 4.10 4.09 4.04 4.03 4.02 4.01 3.94 3.94 3.93 3.92
1.47 1.43 1.38 1.33 1.06 1.05
Me
Me
O
O
TIPS
O
O
Me
Me
2.01 0.98 1.00 1.01 1.01 1.01
3.29 3.00 3.00 3.02 21.17
f1 (ppm)

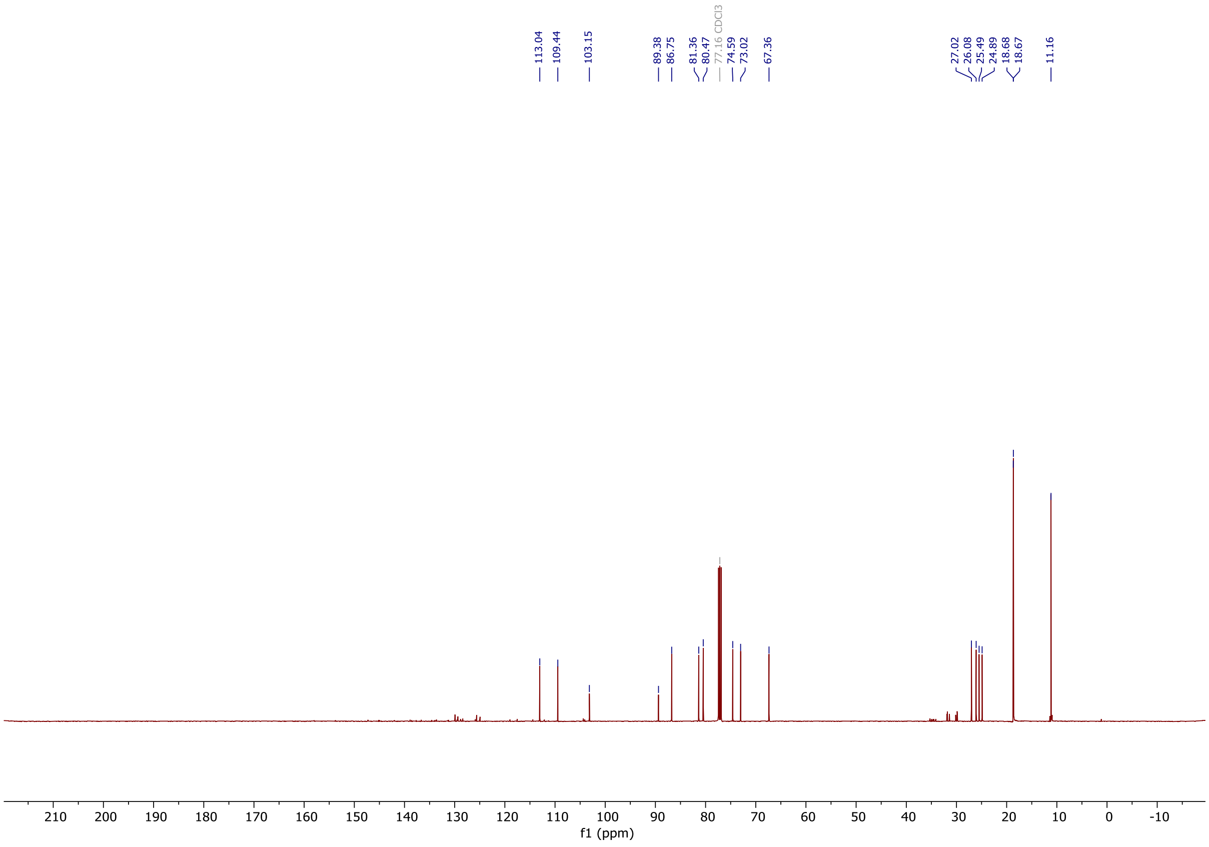
113.04 109.44 103.15 89.38 86.75 81.36 80.47 77.16 CDCl3 74.59 73.02 67.36
27.02 26.08 25.49 24.89 18.68 18.67 11.16
f1 (ppm)

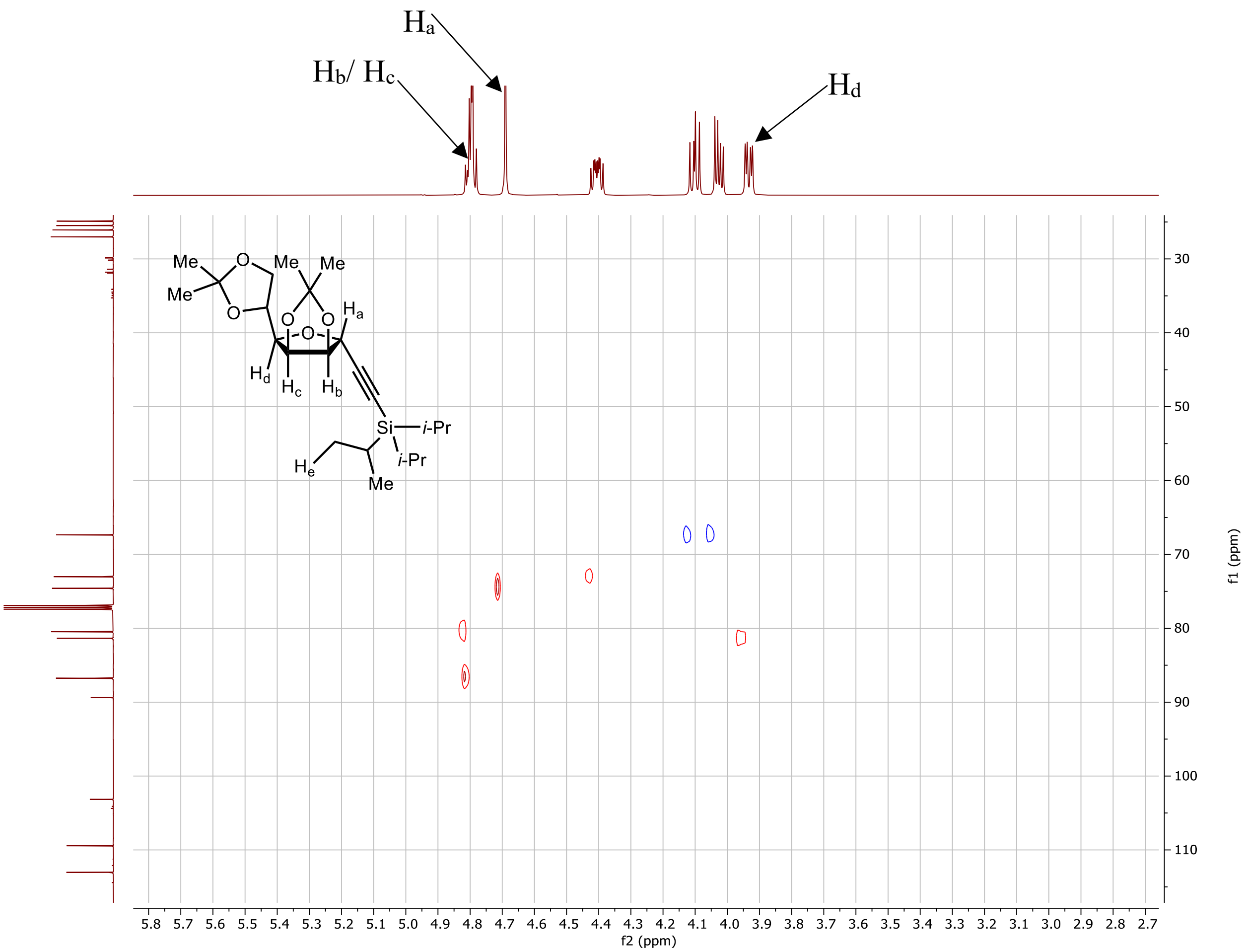

Ha
Hb/ Hc
Hd
Me
O
Me
Me
Me
O
O
O
O
Ha
Hd
Hc
Hb
Si
i-Pr
i-Pr
He
Me
f1 (ppm)
f2 (ppm)

C-H HSQC of **34**

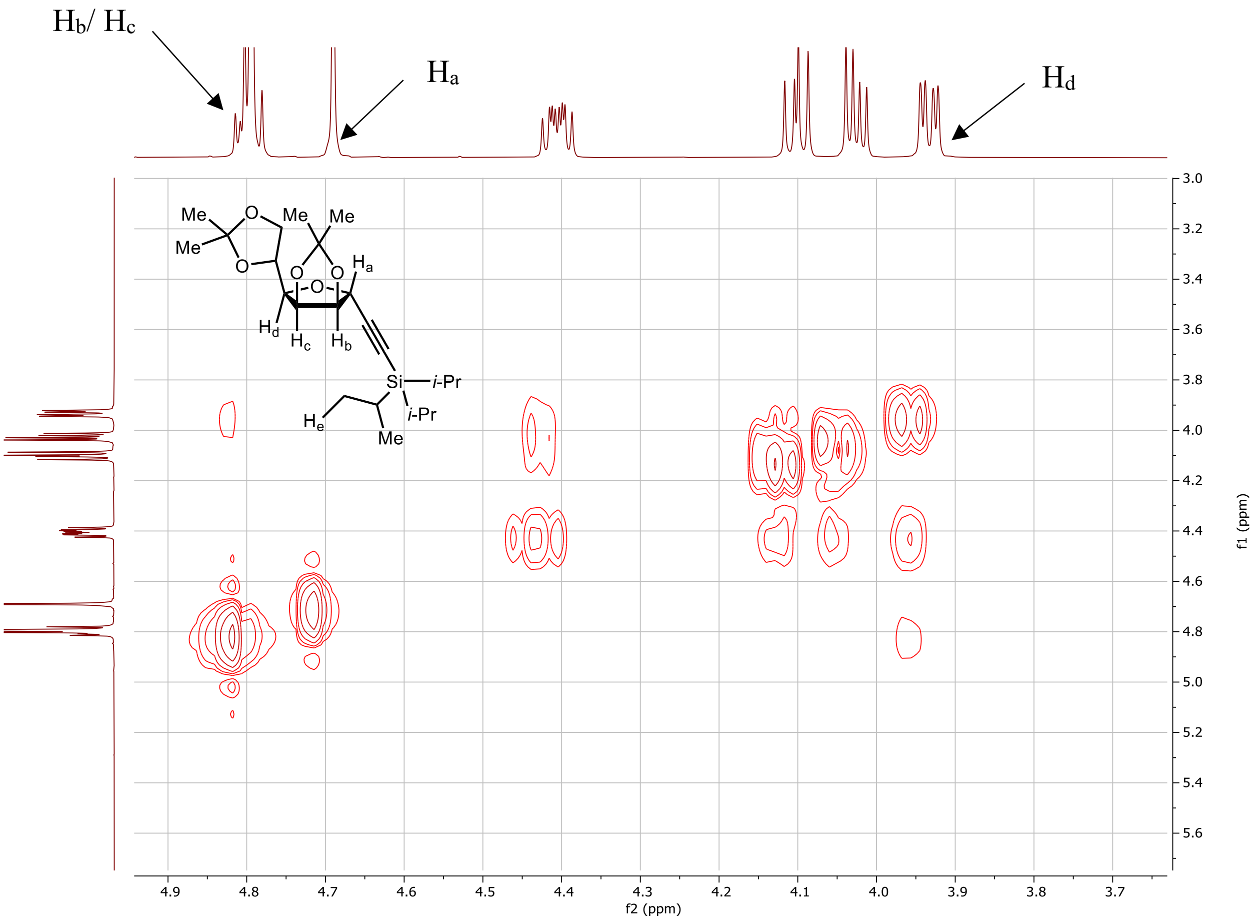
Hb/ Hc
Ha
Hd
Me
Me
O
O
Me
Me
O
O
O
Ha
Hd
Hc
Hb
Si
i-Pr
i-Pr
He
Me
f2 (ppm)
f1 (ppm)

H-H HSQC of **34**

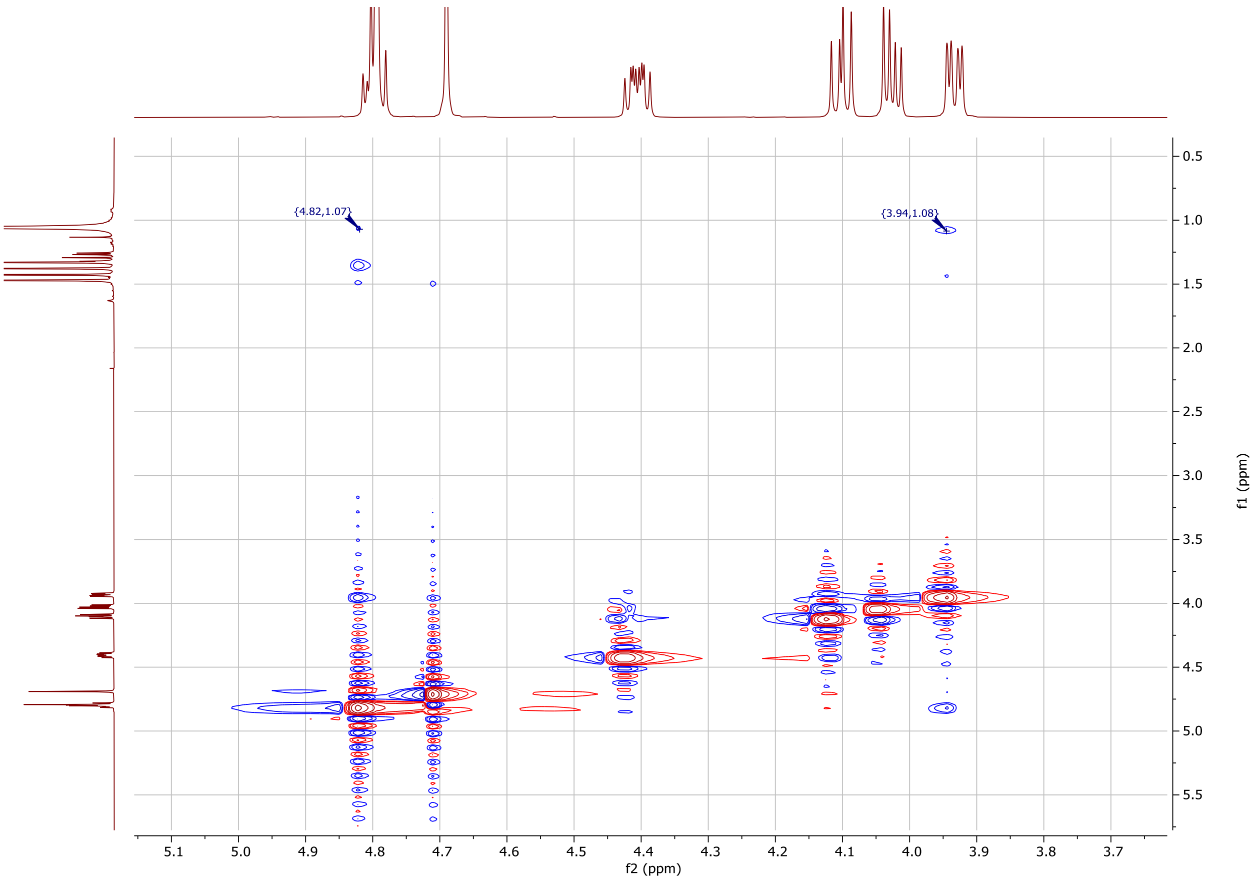

H-H NOESY of **34**

H_e and H_d, with NOE;

H_e and H_b/H_c, with NOE;

H_a and H_d, no NOE;

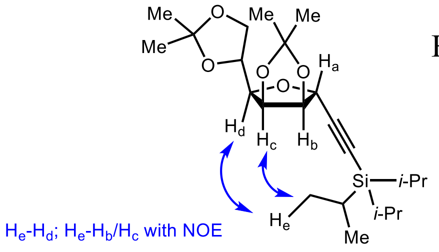

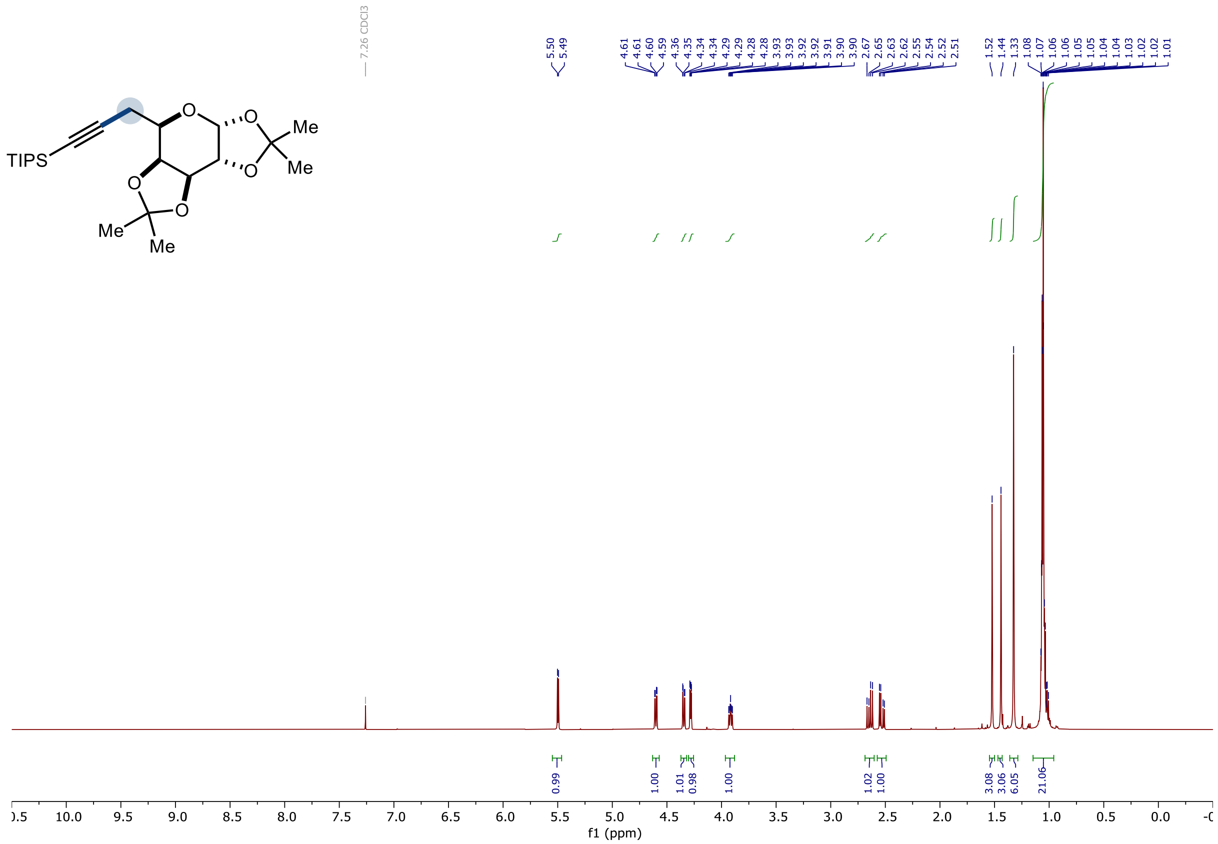
TIPS
O
Me
Me
O
O
O
Me
Me
O
7.26 CDCl3
5.50
5.49
4.61
4.61
4.60
4.59
4.36
4.35
4.34
4.34
4.29
4.29
4.28
4.28
3.93
3.93
3.92
3.92
3.91
3.90
3.90
2.67
2.65
2.63
2.62
2.55
2.54
2.52
2.51
1.52
1.44
1.33
1.08
1.07
1.06
1.06
1.05
1.05
1.04
1.04
1.03
1.02
1.02
1.01
0.99
1.00
1.01
0.98
1.00
1.02
1.00
3.08
3.06
6.05
21.06
f1 (ppm)

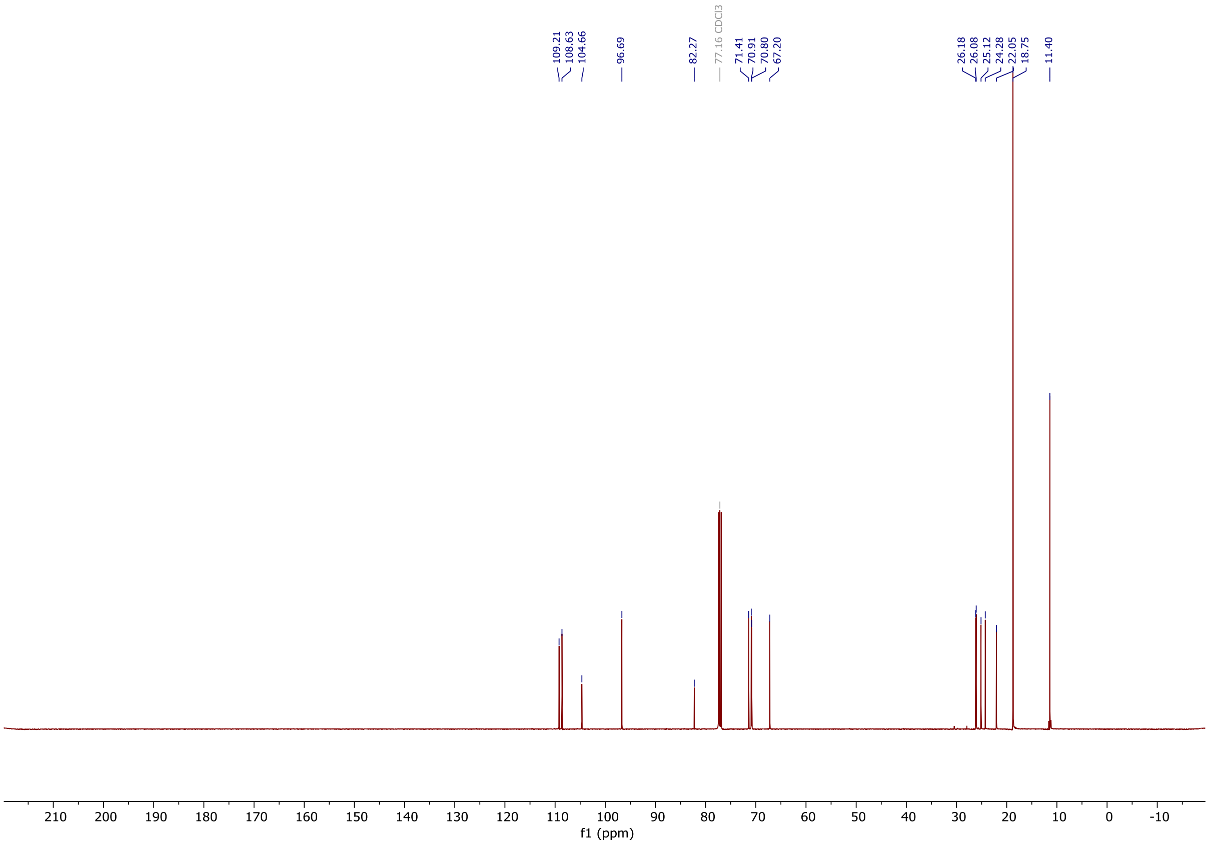
109.21
108.63
104.66
96.69
82.27
77.16 CDCl3
71.41
70.91
70.80
67.20
26.18
26.08
25.12
24.28
22.05
18.75
11.40
f1 (ppm)

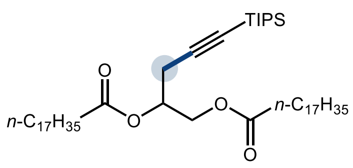
TIPS
O
O
O
O
n-C17H35
n-C17H35

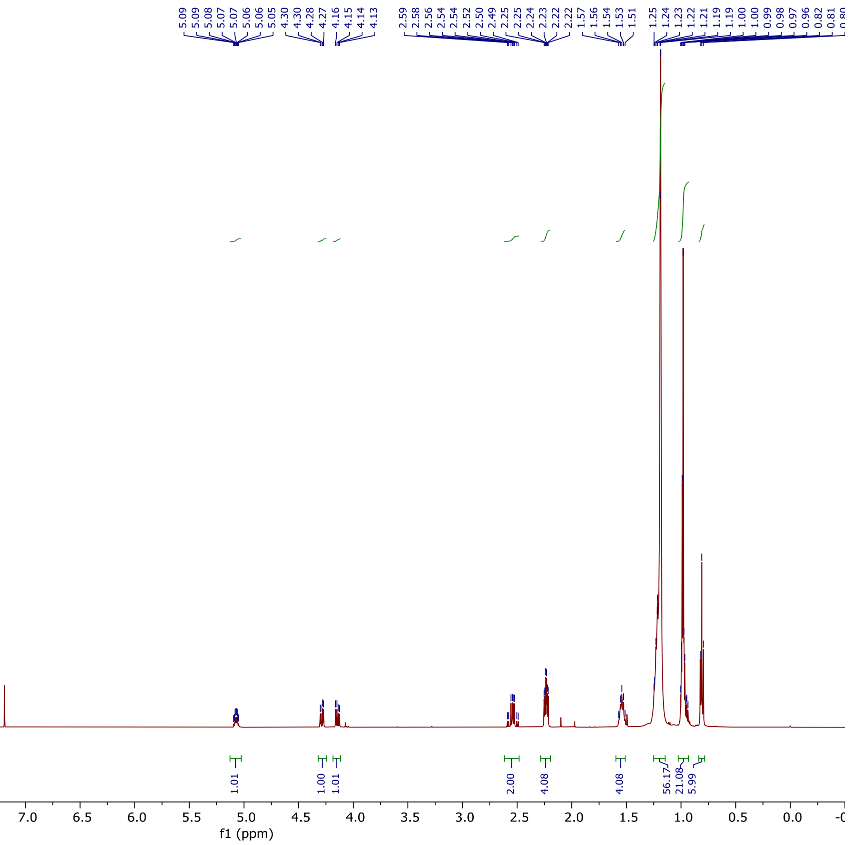
5.09 5.09 5.08 5.07 5.07 5.06 5.06 5.05 4.30 4.30 4.28 4.27 4.16 4.15 4.14 4.13
2.59 2.58 2.56 2.54 2.54 2.52 2.50 2.49 2.25 2.25 2.24 2.23 2.22 2.22 1.57 1.56 1.54 1.53 1.51
1.25 1.24 1.23 1.22 1.21 1.19 1.19 1.00 1.00 0.99 0.98 0.97 0.96 0.82 0.81
1.01 1.00 1.01 2.00 4.08 4.08 56.17 21.08 5.99
10.0 9.5 9.0 8.5 8.0 7.5 7.0 6.5 6.0 5.5 5.0 4.5 4.0 3.5 3.0 2.5 2.0 1.5 1.0 0.5 0.0
f1 (ppm)

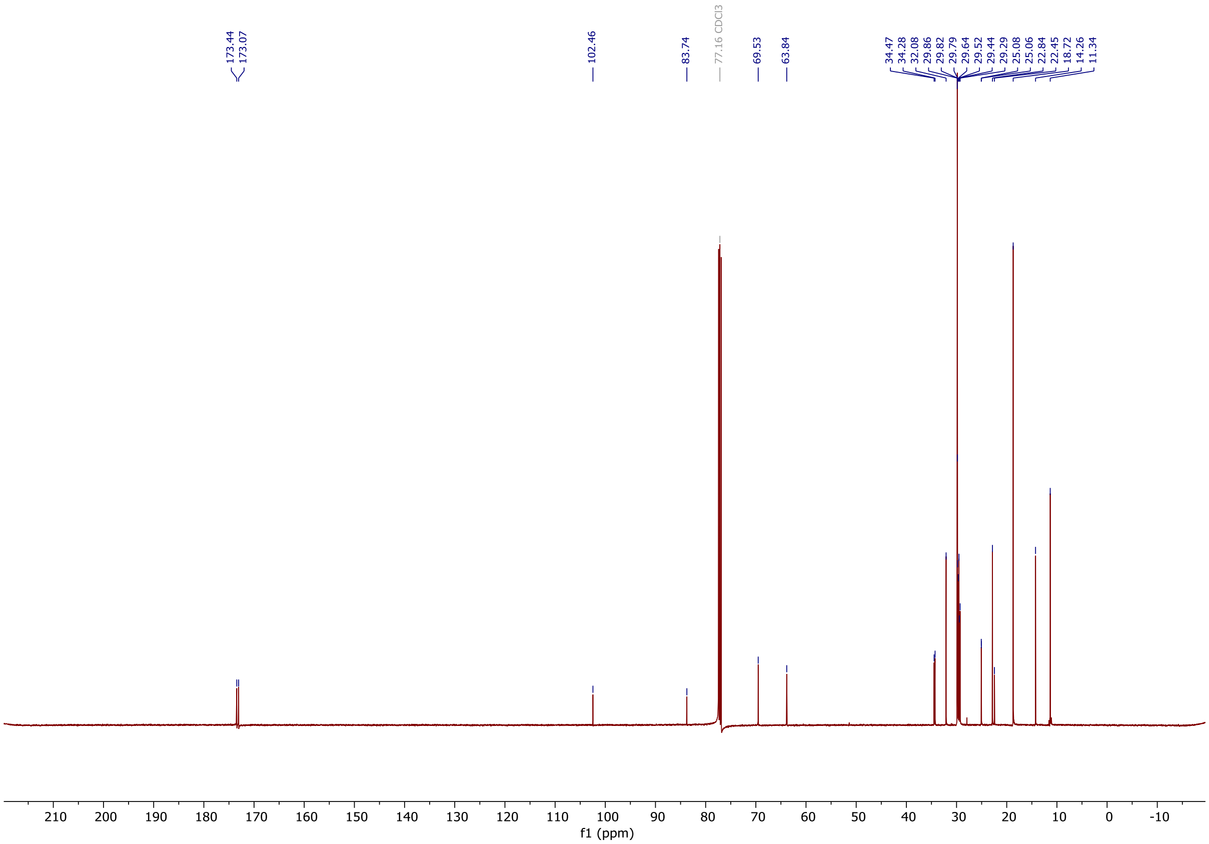
173.44 173.07
102.46
83.74
77.16 CDCl3
69.53
63.84
34.47 34.28 32.08 29.86 29.82 29.79 29.64 29.52 29.44 29.29 25.08 25.06 22.84 22.45 18.72 14.26 11.34
210 200 190 180 170 160 150 140 130 120 110 100 90 80 70 60 50 40 30 20 10 0 -10
f1 (ppm)

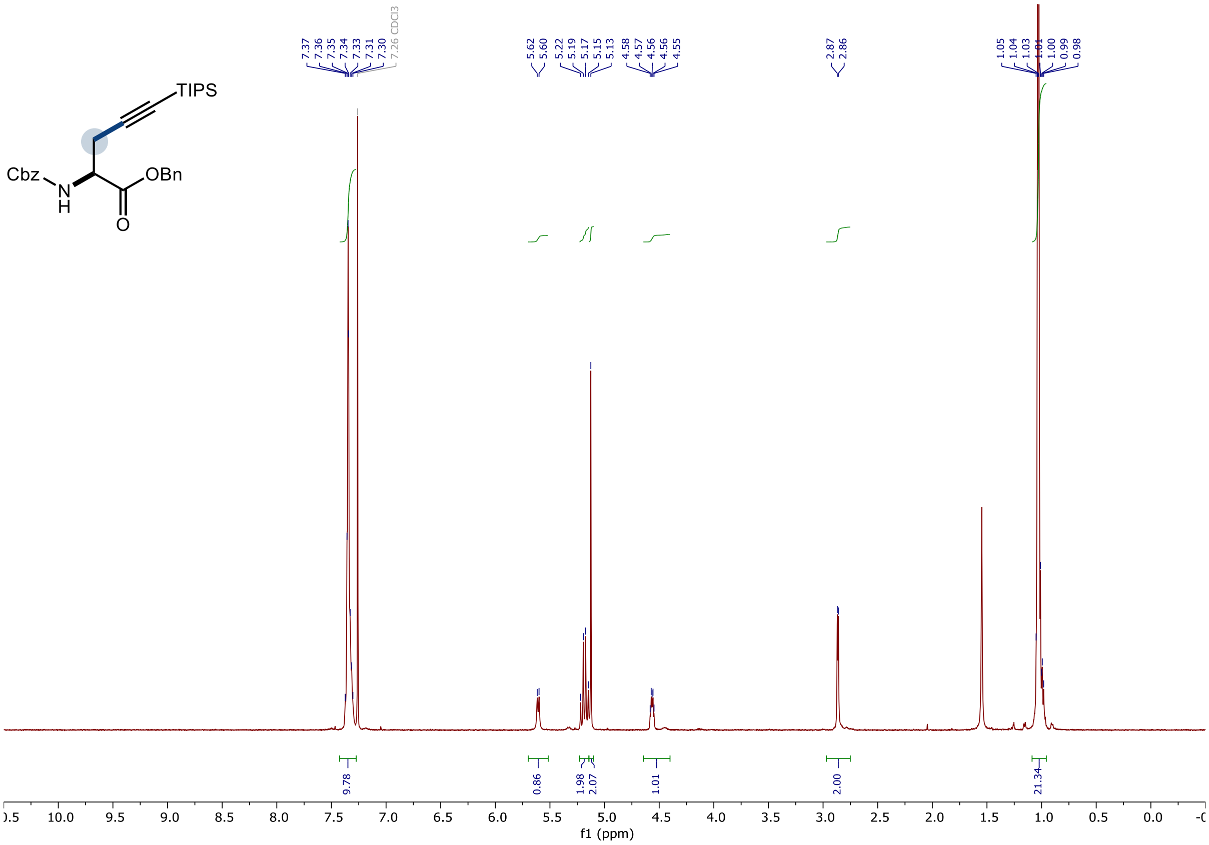
TIPS
Cbz
N
H
OBn
O

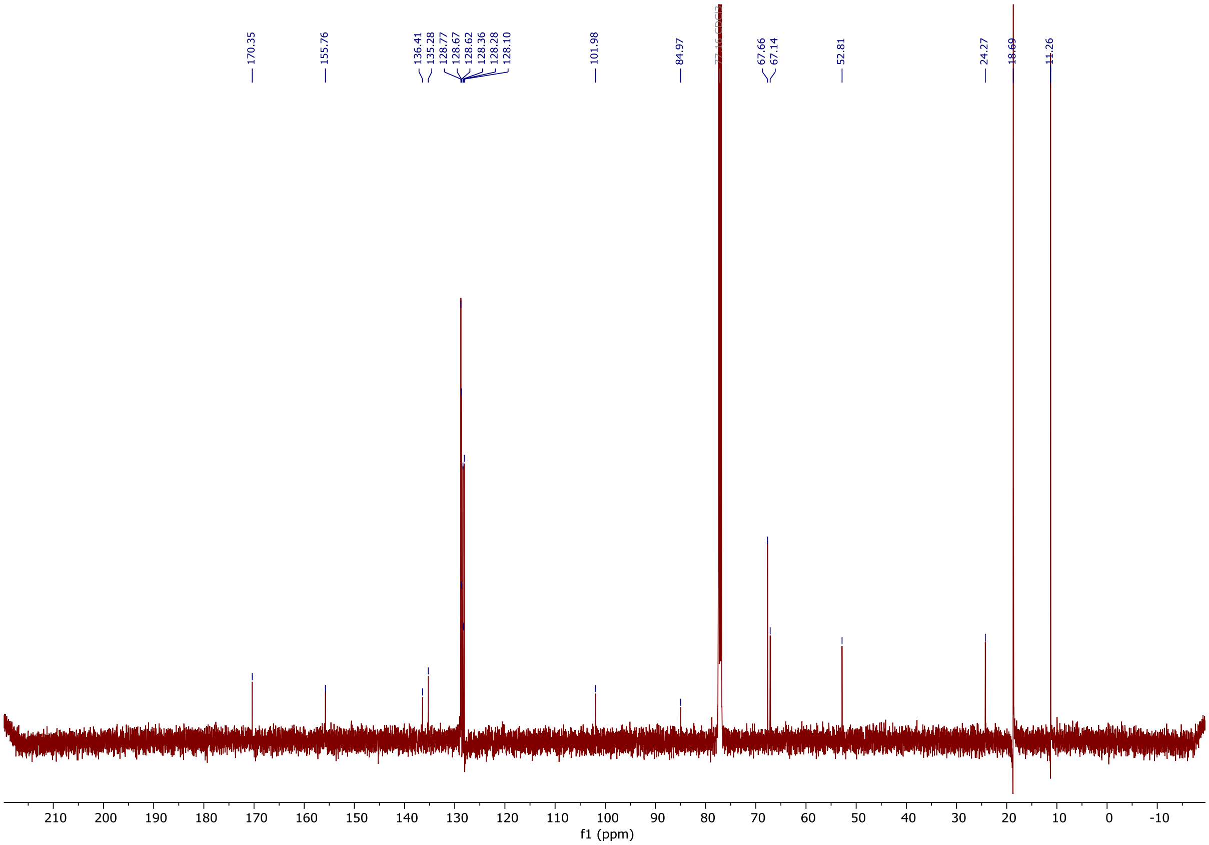

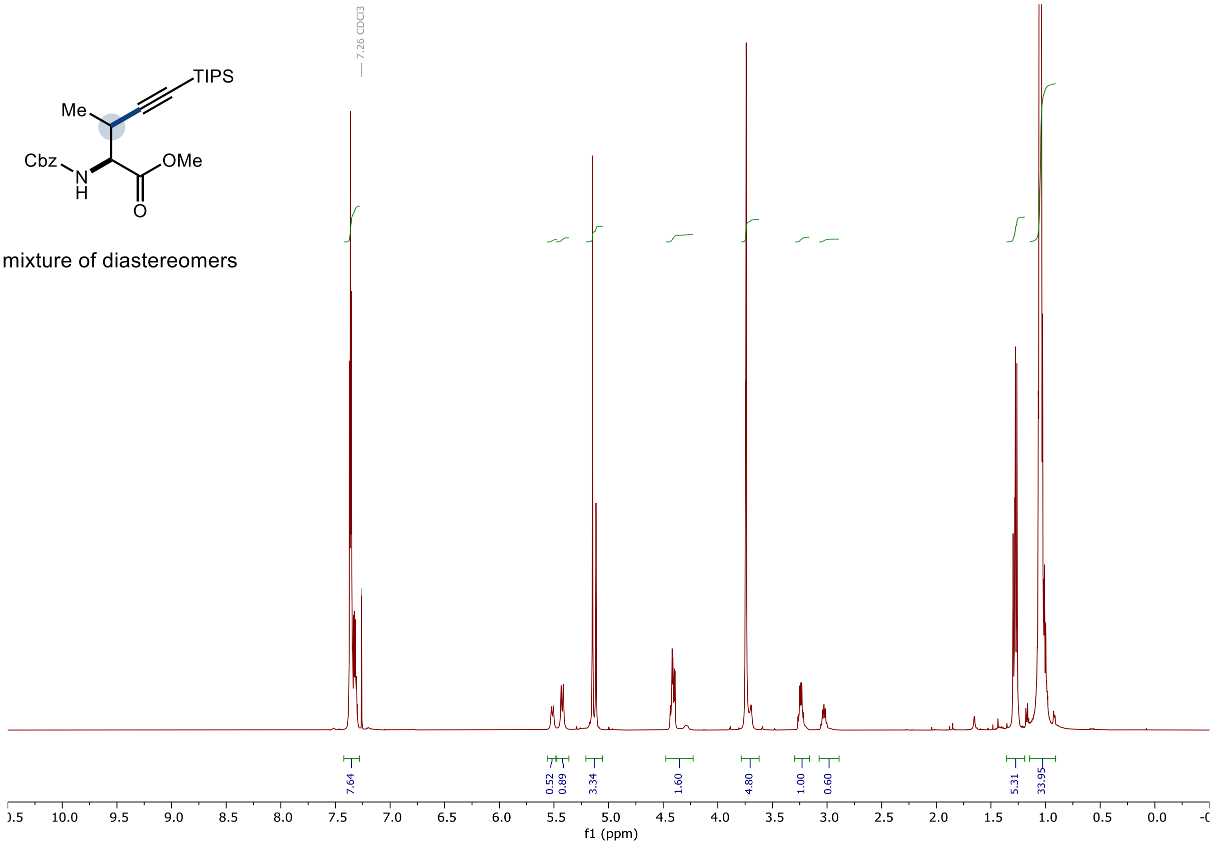
TIPS
Me
Cbz
N
H
OMe
O
mixture of diastereomers
7.26 CDCl3
7.64
0.52
0.89
3.34
1.60
4.80
1.00
0.60
5.31
33.95
f1 (ppm)

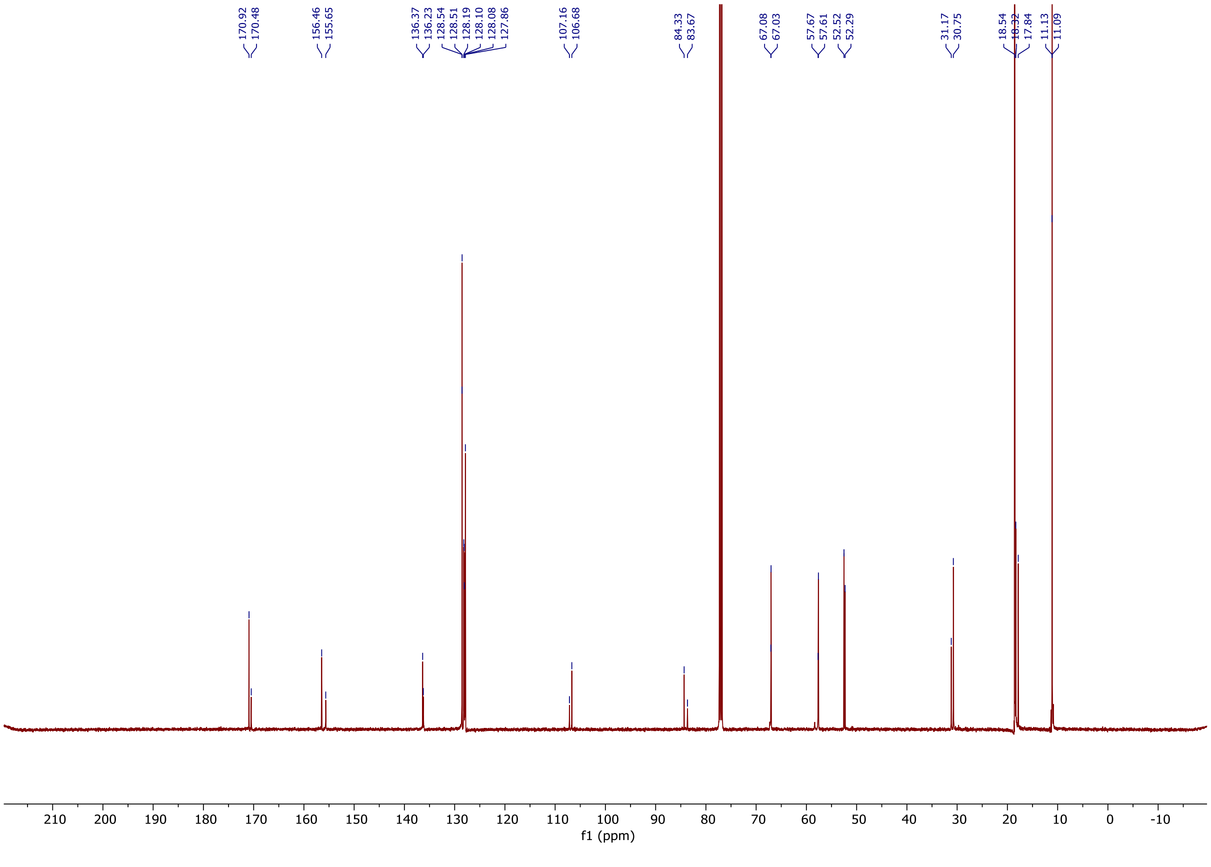
170.92
170.48
156.46
155.65
136.37
136.23
128.54
128.51
128.19
128.10
128.08
127.86
107.16
106.68
84.33
83.67
67.08
67.03
57.67
57.61
52.52
52.29
31.17
30.75
18.54
18.32
17.84
11.13
11.09
f1 (ppm)

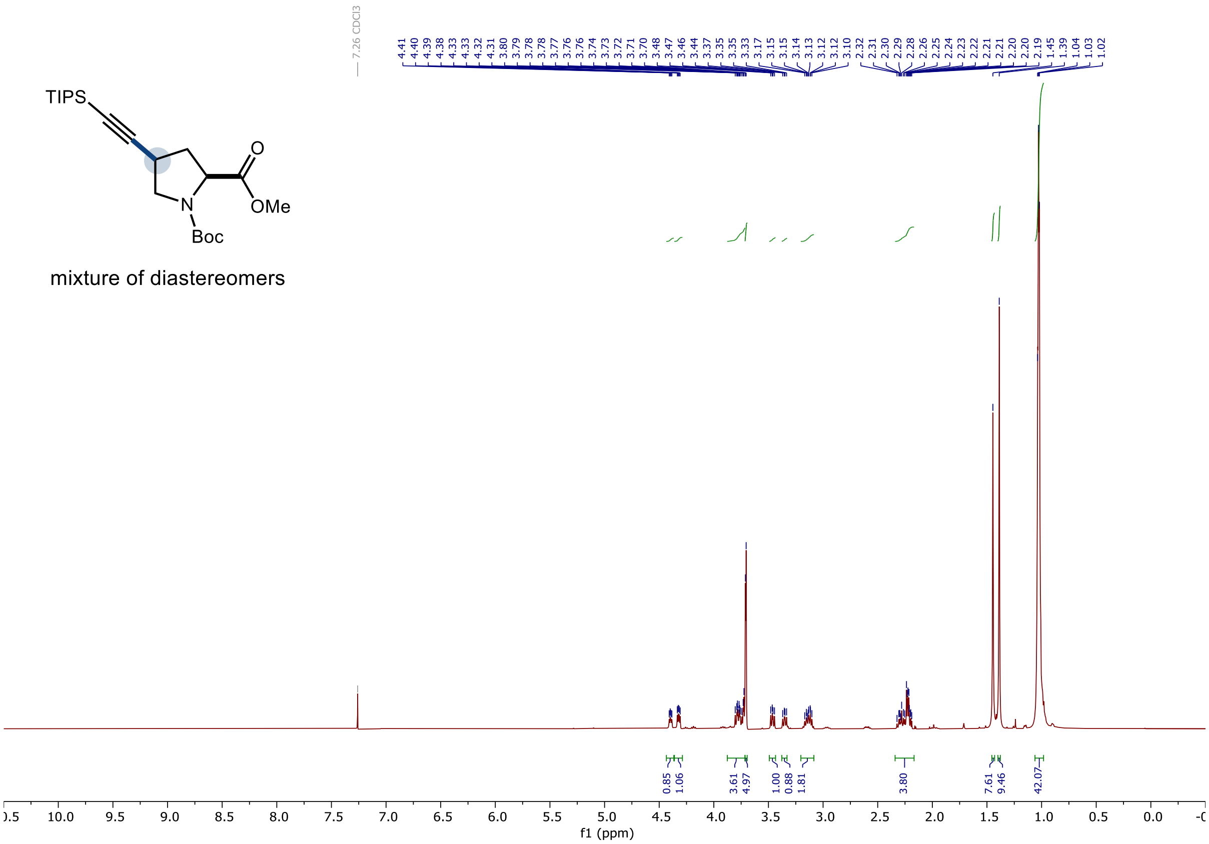
TIPS
O
OMe
N
Boc
mixture of diastereomers
7.26 CDCl3
4.41 4.40 4.39 4.38 4.33 4.33 4.32 4.31 3.80 3.79 3.78 3.78 3.77 3.76 3.76 3.74 3.73 3.72 3.71 3.70 3.48 3.47 3.46 3.44 3.37 3.35 3.35 3.33 3.17 3.15 3.15 3.14 3.13 3.12 3.12 3.10 2.32 2.31 2.30 2.29 2.28 2.26 2.25 2.24 2.23 2.22 2.21 2.21 2.20 2.20 2.19 1.45 1.39 1.04 1.03 1.02
0.85 1.06 3.61 4.97 1.00 0.88 1.81 3.80 7.61 9.46 42.07
10.0 9.5 9.0 8.5 8.0 7.5 7.0 6.5 6.0 5.5 5.0 4.5 4.0 3.5 3.0 2.5 2.0 1.5 1.0 0.5 0.0
f1 (ppm)

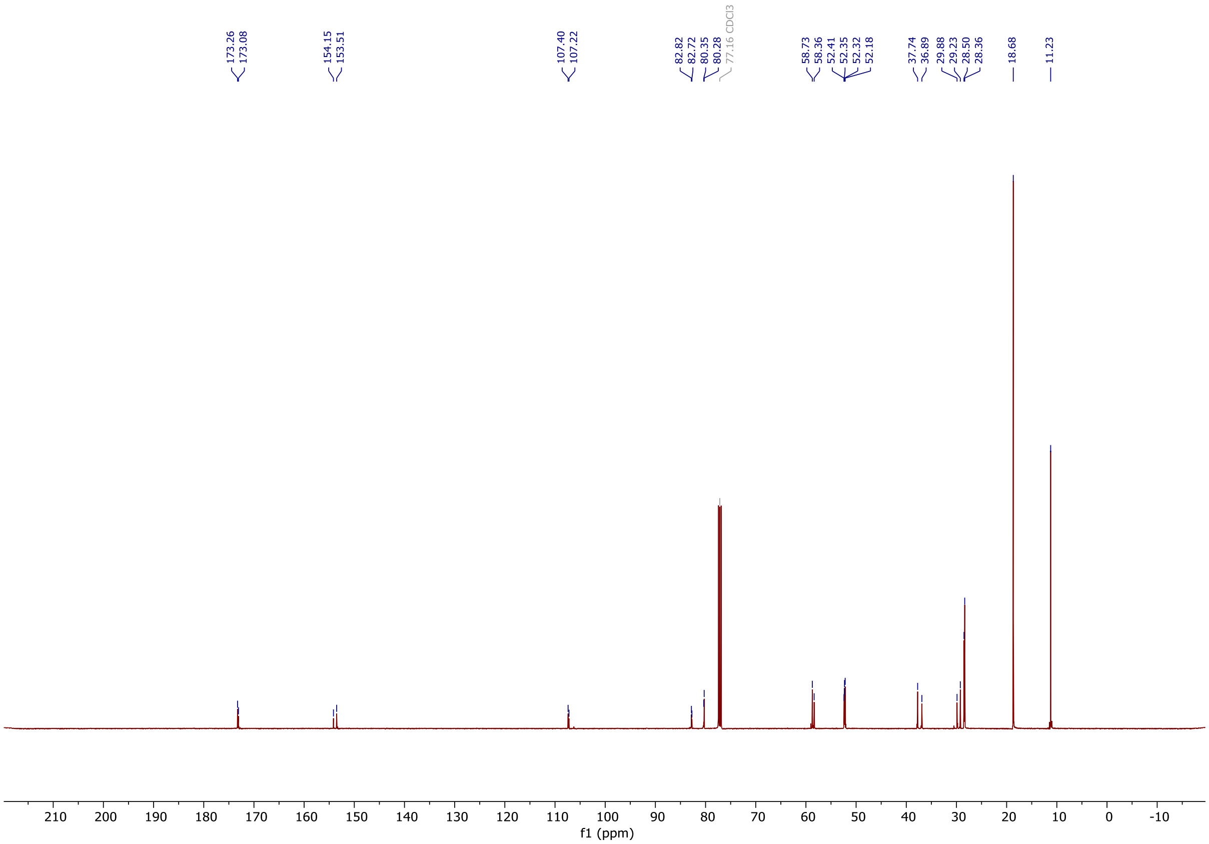
173.26 173.08 154.15 153.51 107.40 107.22 82.82 82.72 80.35 80.28 77.16 CDCl3 58.73 58.36 52.41 52.35 52.32 52.18 37.74 36.89 29.88 29.23 28.50 28.36 18.68 11.23
210 200 190 180 170 160 150 140 130 120 110 100 90 80 70 60 50 40 30 20 10 0 -10
f1 (ppm)

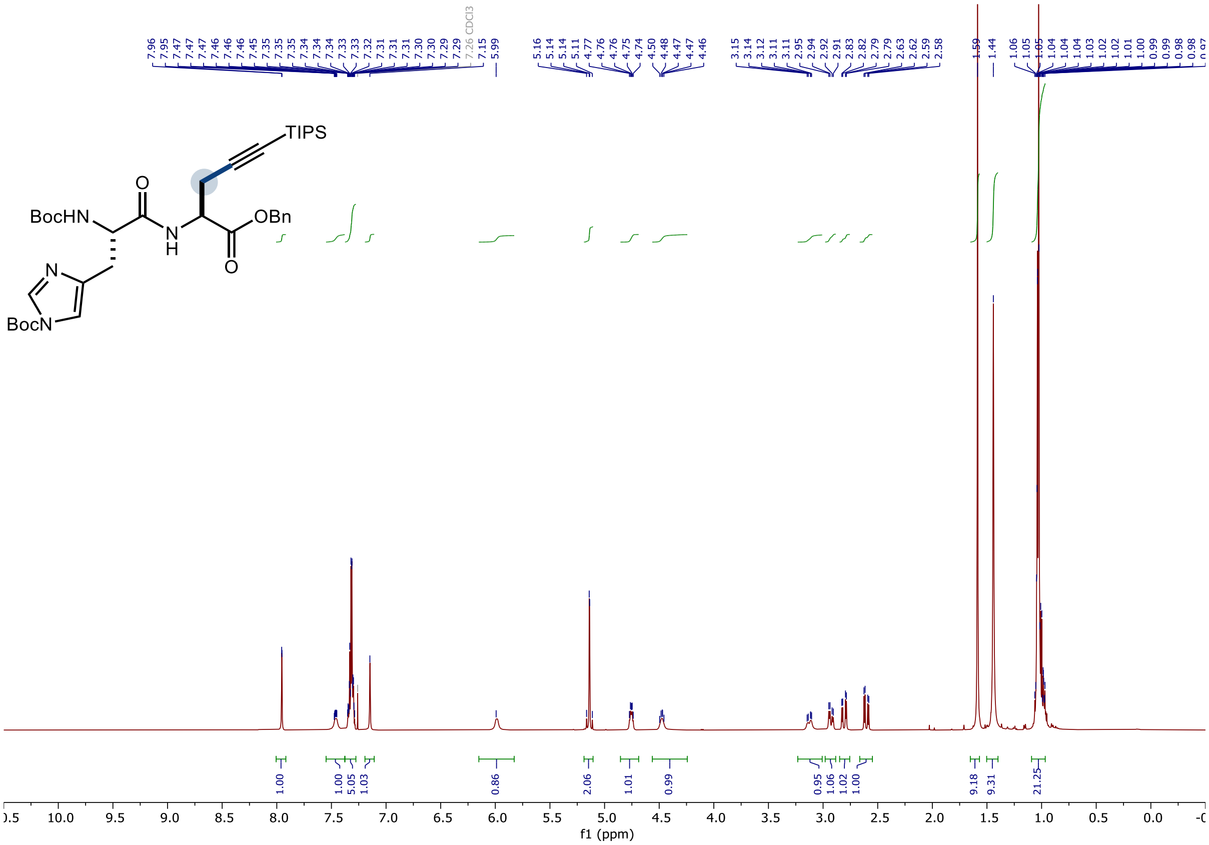
TIPS
O
BocHN
N
H
OBn
O
N
BocN

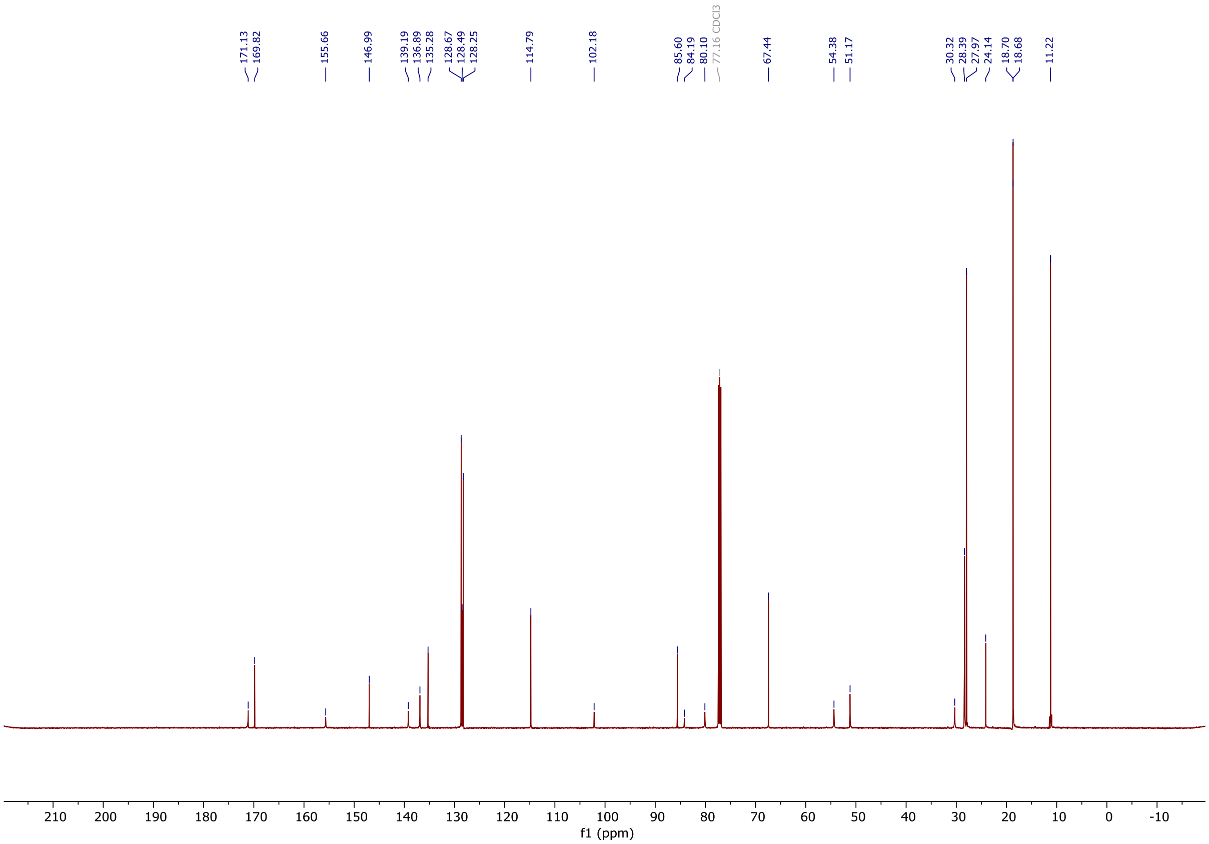

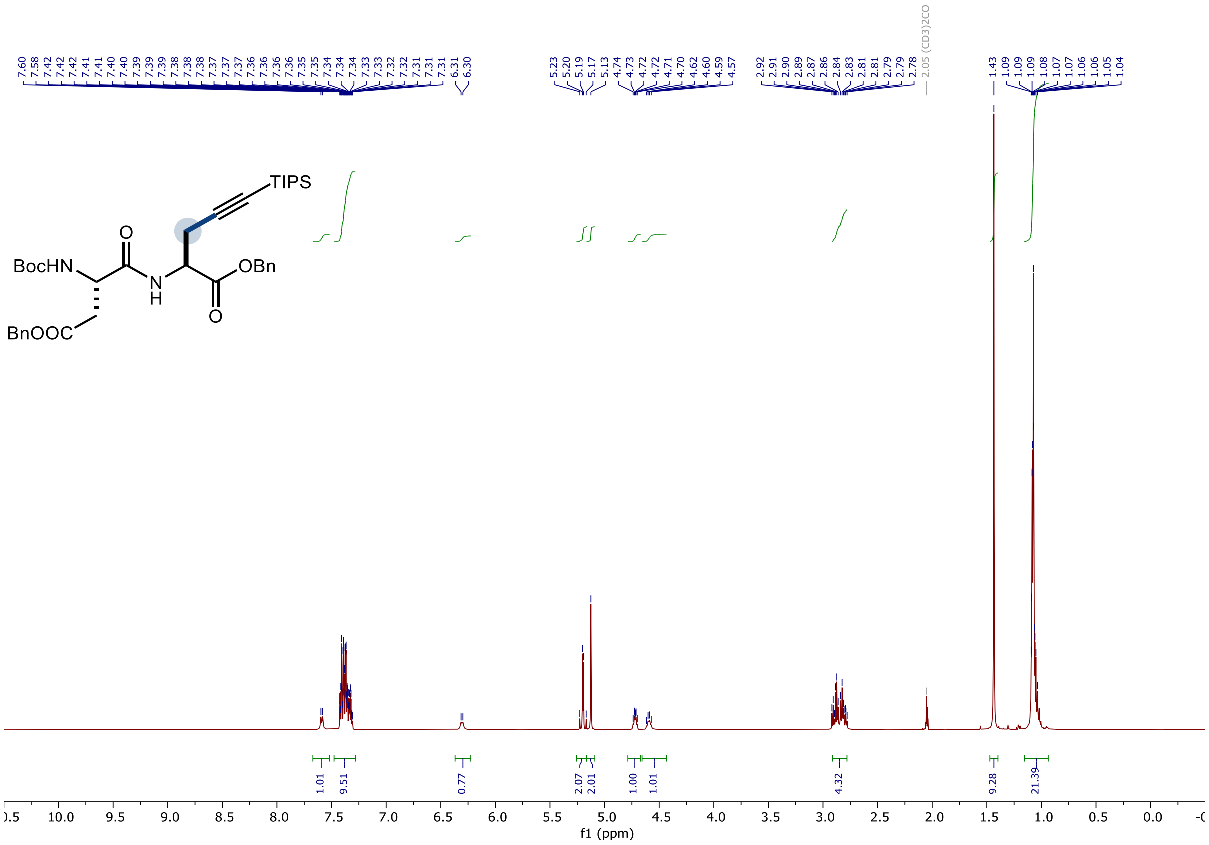

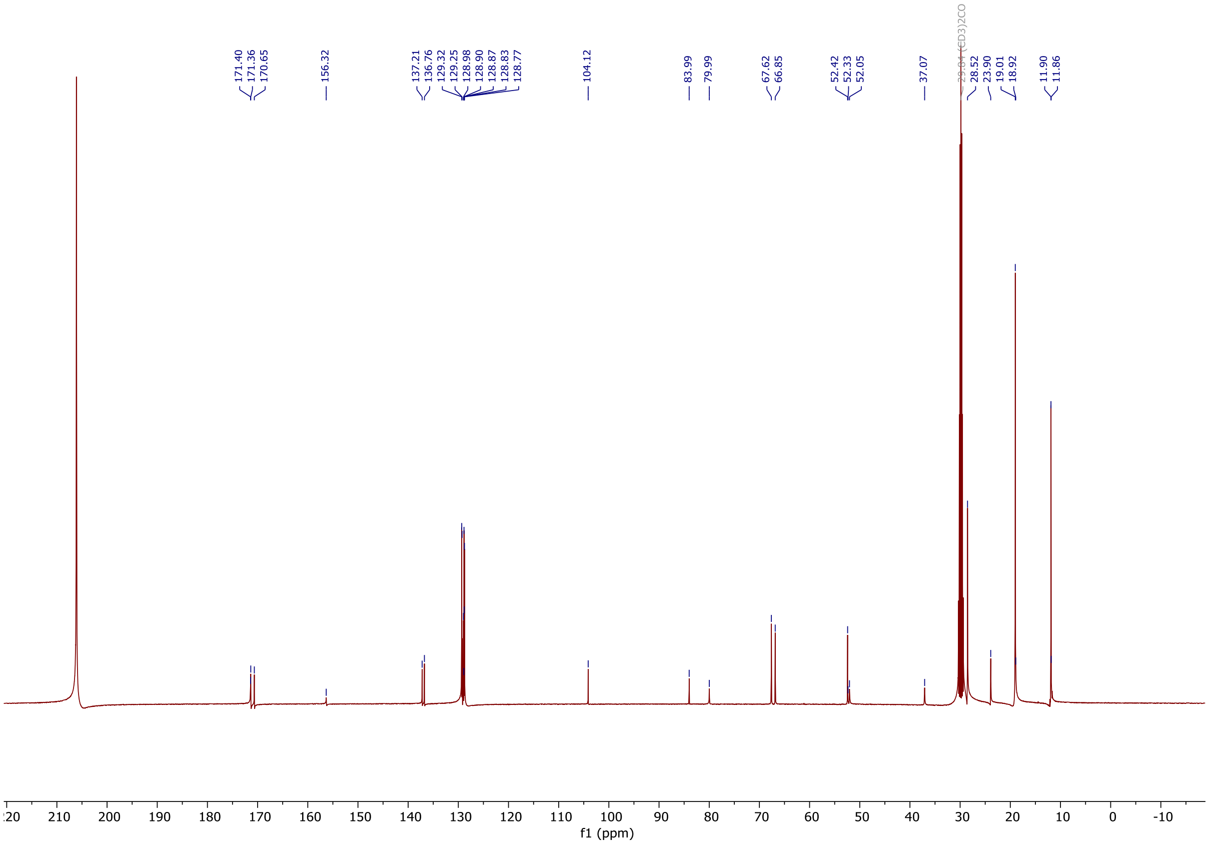

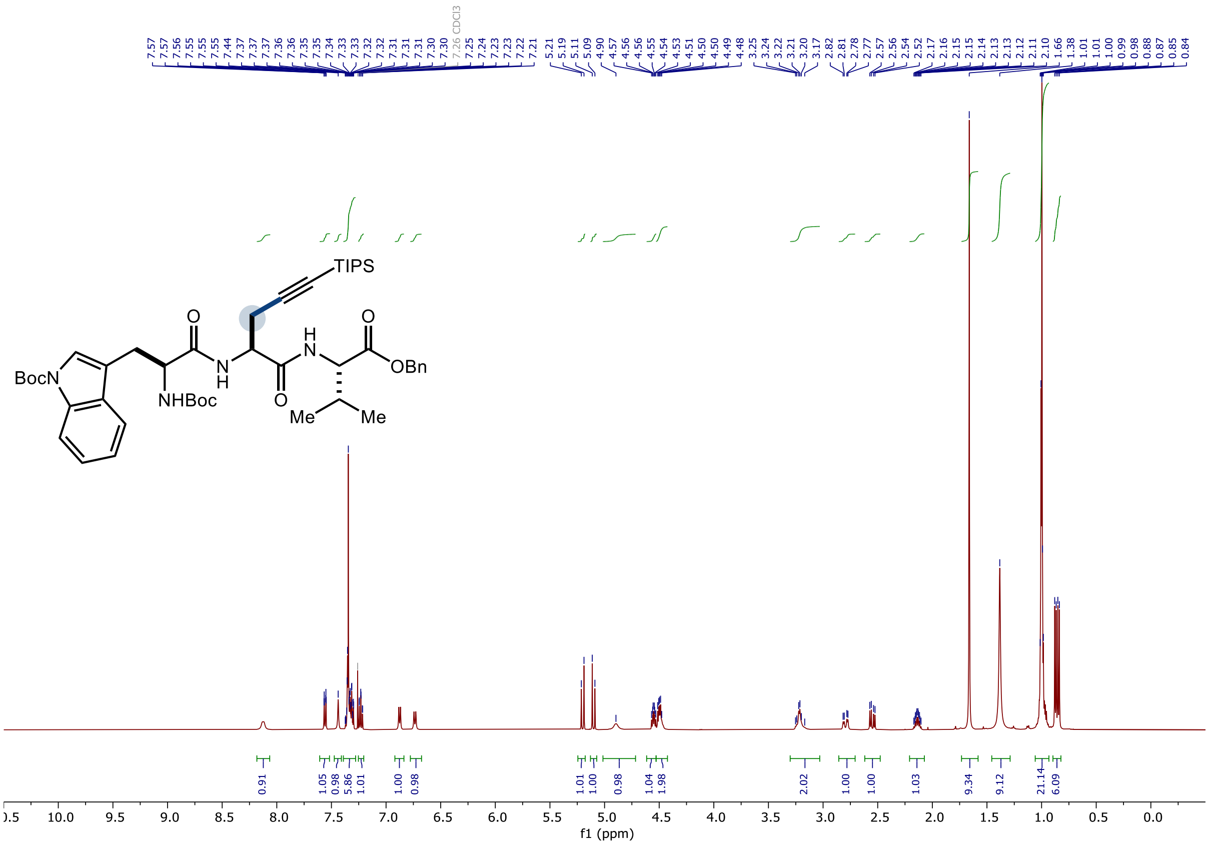
7.57 7.57 7.56 7.55 7.55 7.44 7.37 7.37 7.37 7.36 7.36 7.35 7.35 7.34 7.33 7.33 7.32 7.32 7.31 7.31 7.30 7.26 CDCl3 7.25 7.24 7.23 7.23 7.22 7.21 5.21 5.19 5.11 5.09 4.90 4.57 4.56 4.56 4.55 4.54 4.53 4.51 4.50 4.50 4.49 4.48 3.25 3.24 3.22 3.21 3.20 3.17 2.82 2.81 2.78 2.77 2.57 2.56 2.54 2.52 2.17 2.16 2.15 2.15 2.14 2.13 2.13 2.13 2.12 2.11 2.10 1.66 1.38 1.01 1.01 1.00 0.99 0.98 0.88 0.87 0.85 0.84
TIPS
O
BocN
NHBoc
N
H
H
N
O
O
OBn
Me
Me
0.91 1.05 0.98 5.86 1.01 1.00 0.98 1.01 1.00 0.98 1.04 1.98 2.02 1.00 1.00 1.03 9.34 9.12 21.14 6.09
0.5 10.0 9.5 9.0 8.5 8.0 7.5 7.0 6.5 6.0 5.5 5.0 4.5 4.0 3.5 3.0 2.5 2.0 1.5 1.0 0.5 0.0
f1 (ppm)

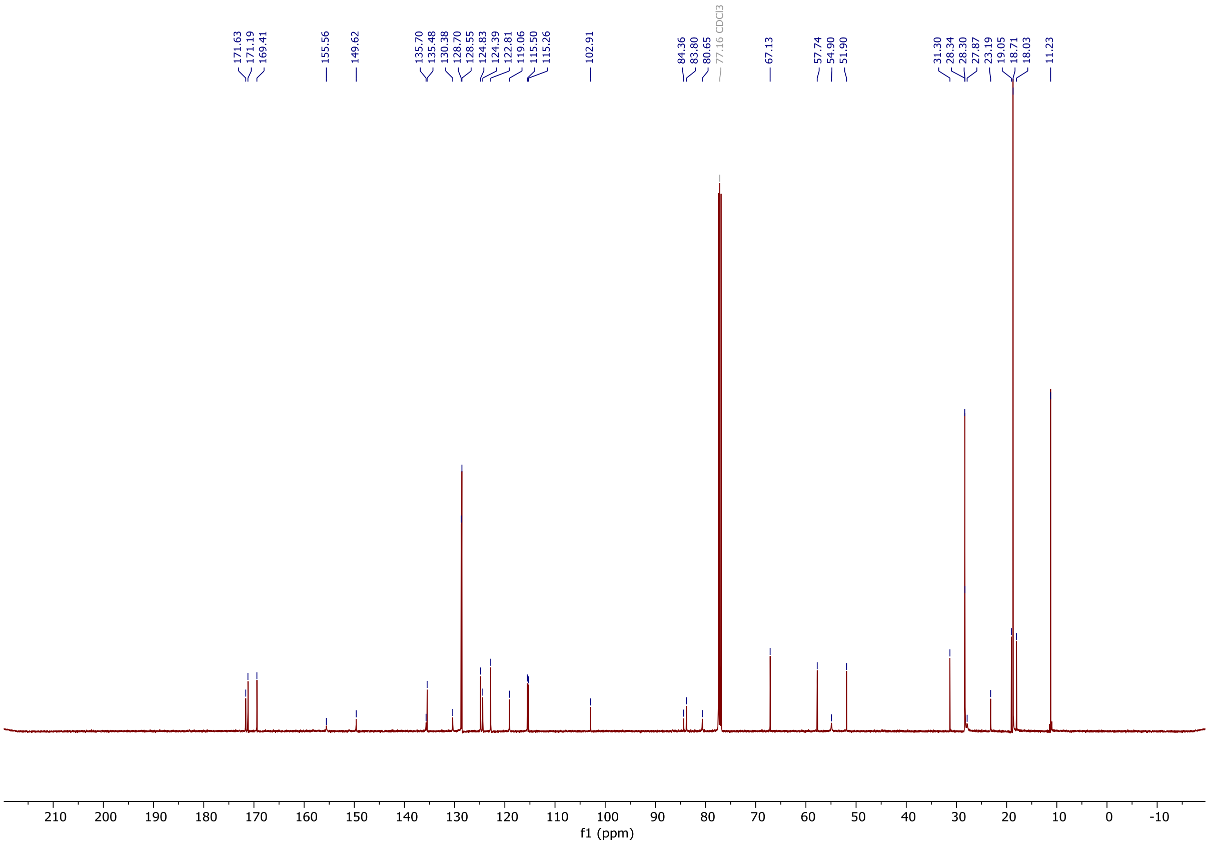
171.63 171.19 169.41 155.56 149.62 135.70 135.48 130.38 128.70 128.55 124.83 124.39 122.81 119.06 115.50 115.26 102.91 84.36 83.80 80.65 77.16 CDCl3 67.13 57.74 54.90 51.90 31.30 28.34 28.30 27.87 23.19 19.05 18.71 18.03 11.23
210 200 190 180 170 160 150 140 130 120 110 100 90 80 70 60 50 40 30 20 10 0 -10
f1 (ppm)

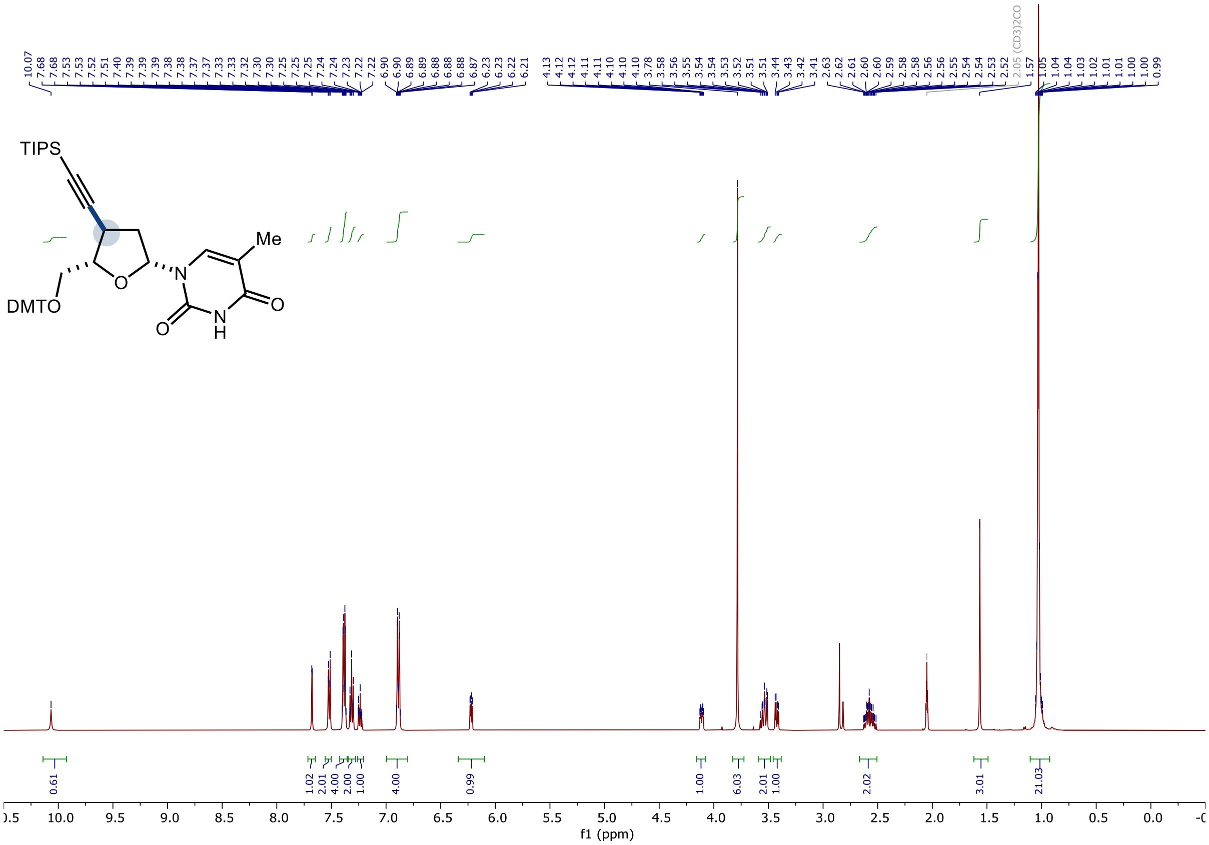

TIPS
Me
DMTO
O
N
N
H
O
O
f1 (ppm)

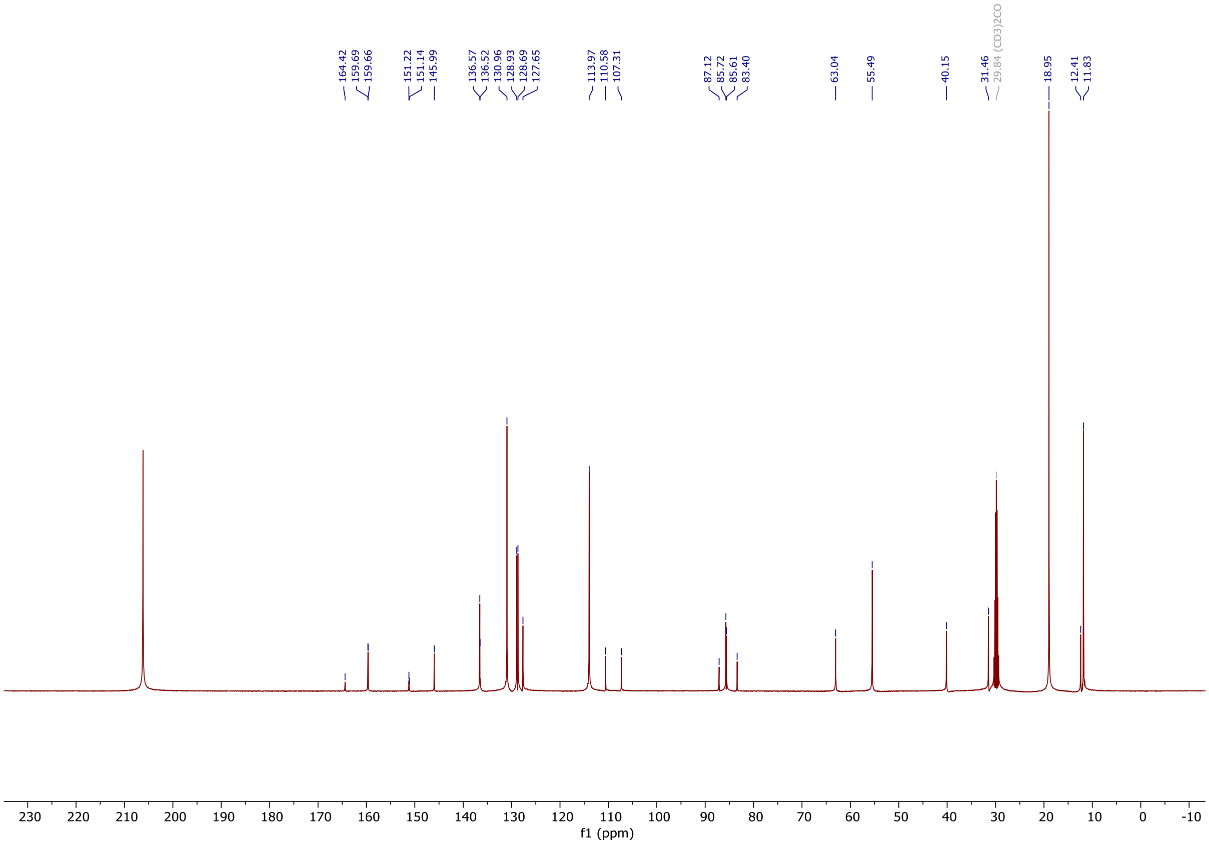

f1 (ppm)

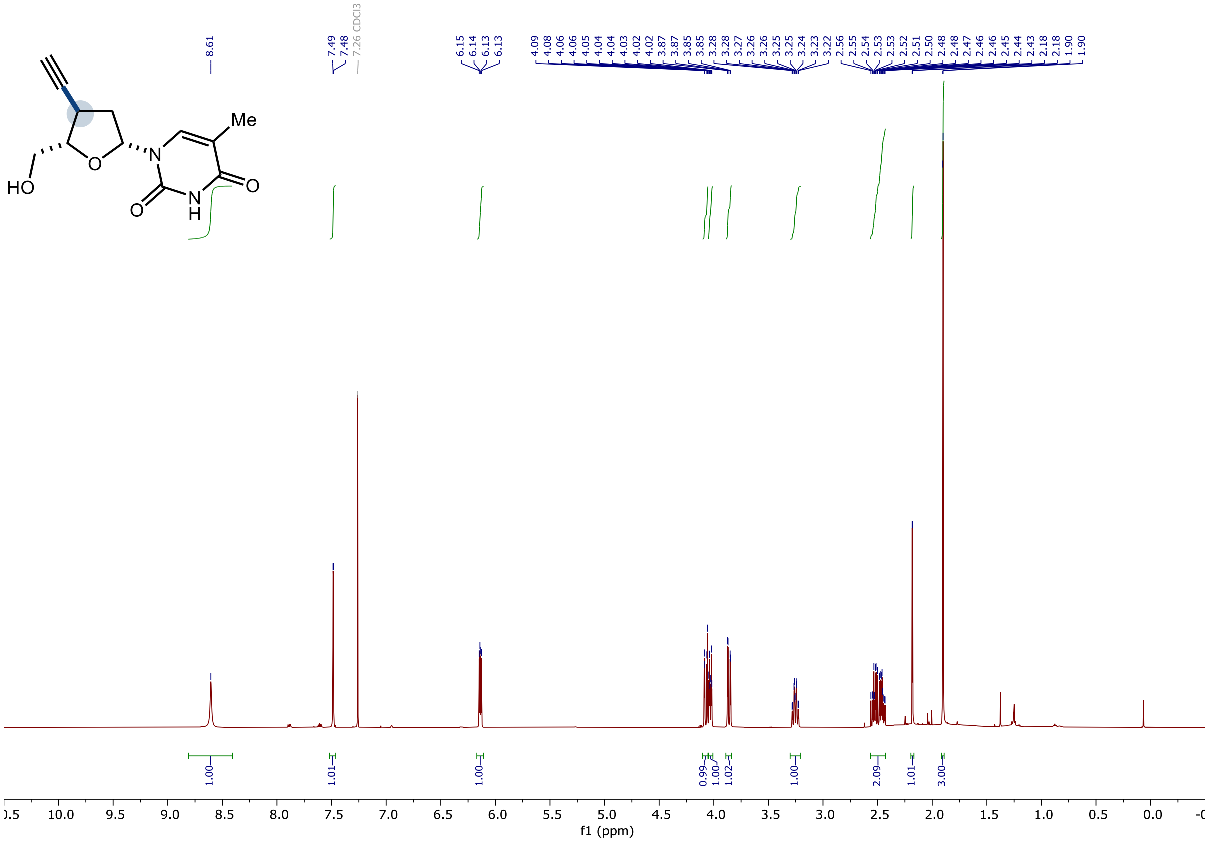
Me
N
O
HO
O
N
H
O
8.61
7.49
7.48
7.26 CDCl3
6.15
6.14
6.13
6.13
4.09 4.08 4.06 4.06 4.05 4.04 4.04 4.03 4.02 4.02 3.87 3.87 3.85 3.85 3.28 3.28 3.27 3.26 3.26 3.25 3.25 3.24 3.23 3.22 2.56 2.55 2.54 2.53 2.53 2.52 2.51 2.50 2.48 2.48 2.47 2.46 2.46 2.45 2.44 2.43 2.18 2.18 1.90 1.90
1.00
1.01
1.00
0.99
1.00
1.02
1.00
2.09
1.01
3.00
f1 (ppm)

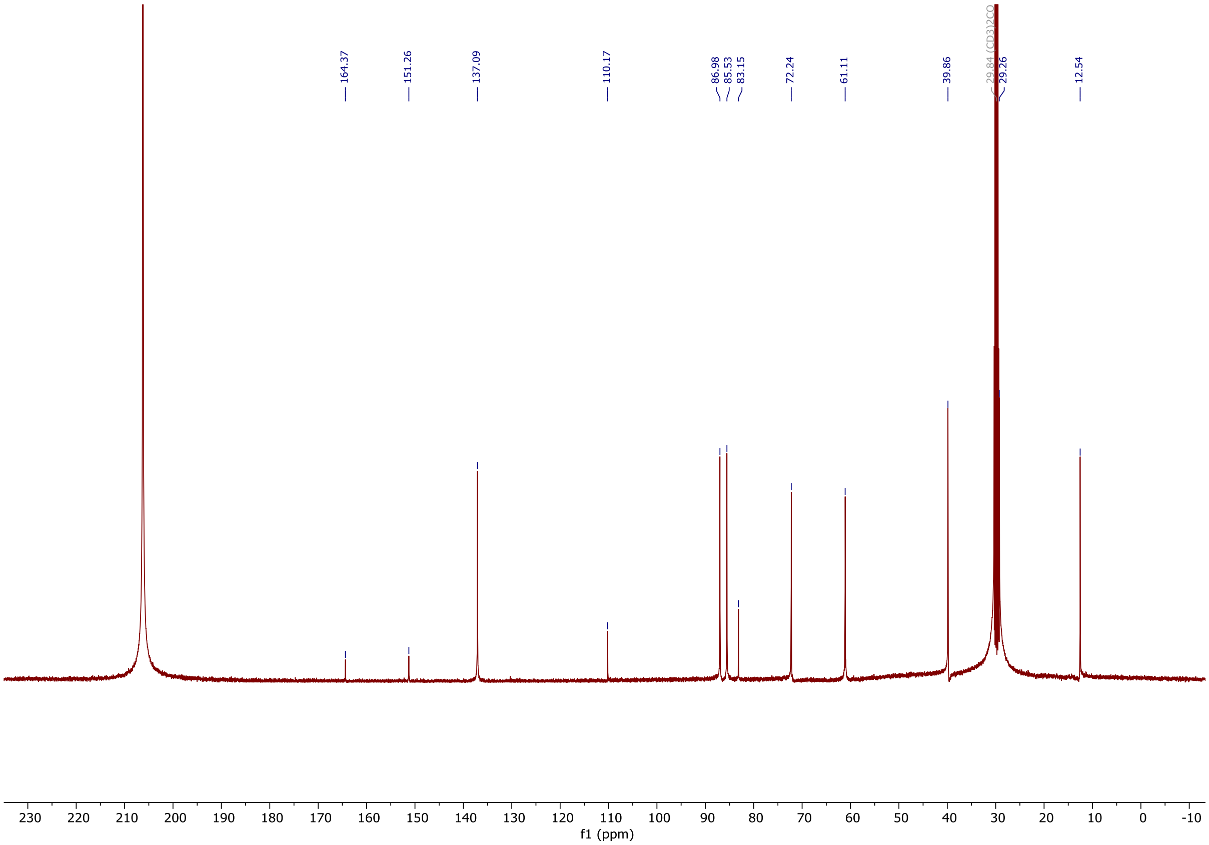
164.37
151.26
137.09
110.17
86.98
85.53
83.15
72.24
61.11
39.86
29.84 (CD3)2CO
29.26
12.54
f1 (ppm)

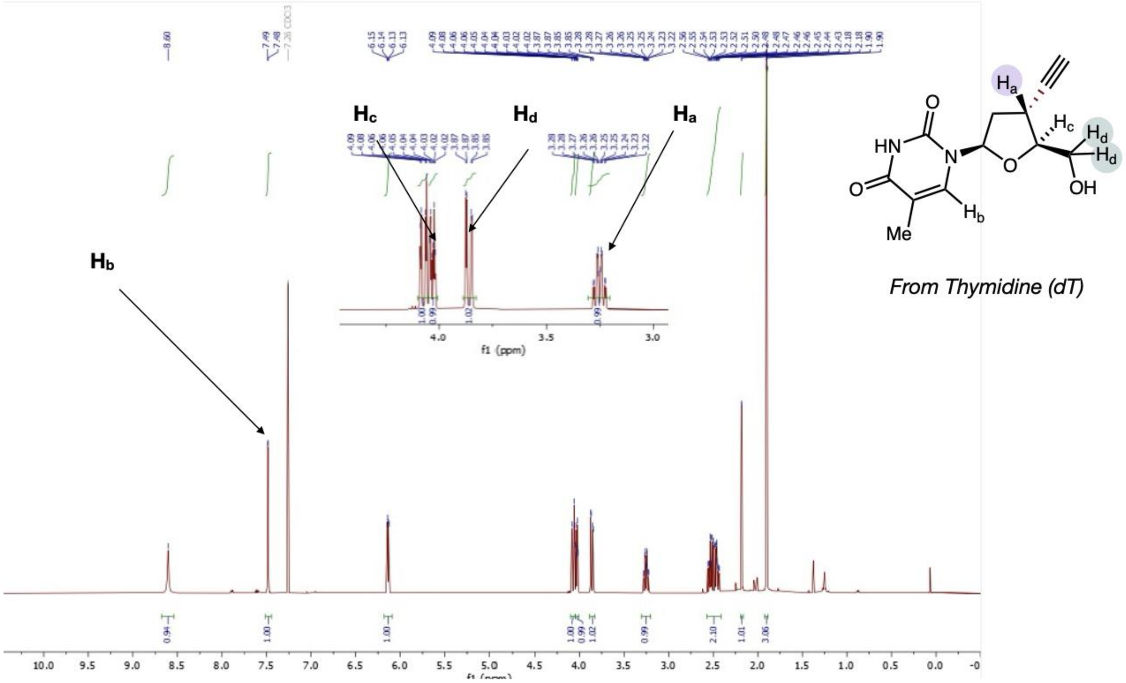

Hb
Hc
Hd
Ha
From Thymidine (dT)
f1 (ppm)

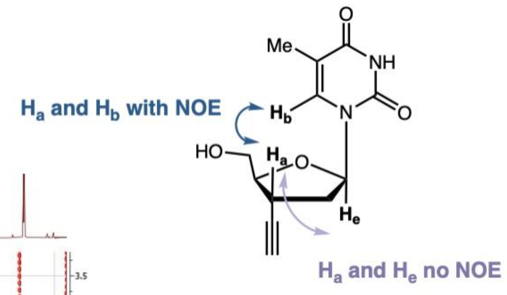

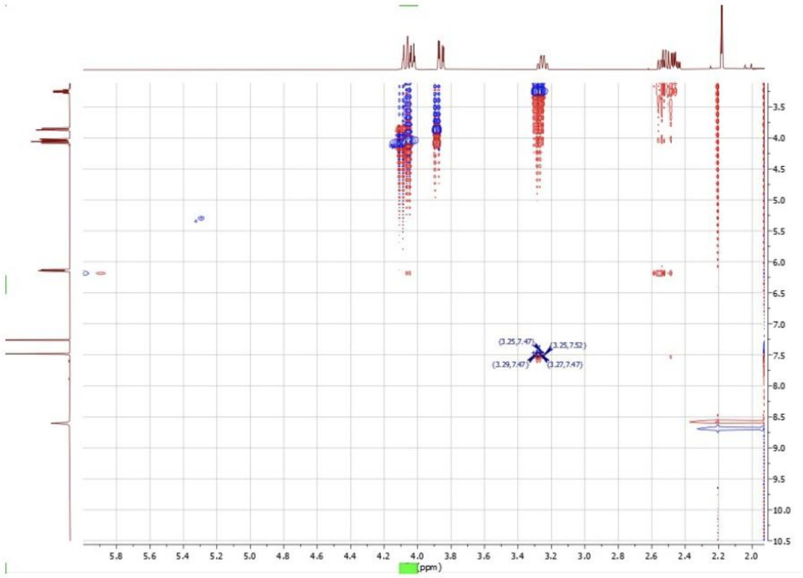

H-H NOESY of **44**

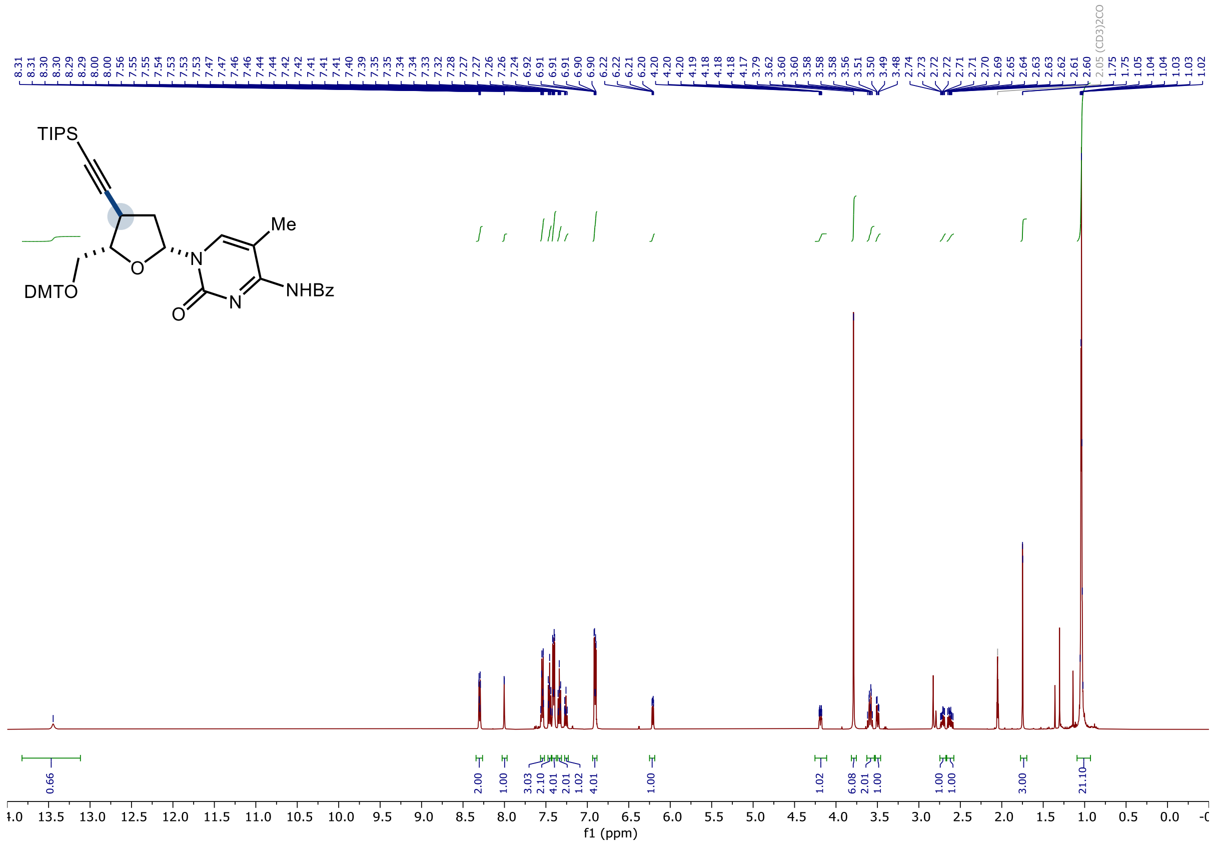
8.31 8.31 8.30 8.30 8.29 8.29 8.00 7.56 7.55 7.55 7.54 7.53 7.53 7.53 7.47 7.47 7.46 7.44 7.44 7.42 7.42 7.41 7.41 7.40 7.39 7.35 7.35 7.34 7.34 7.33 7.32 7.28 7.27 7.27 7.26 7.24 6.92 6.91 6.91 6.91 6.90 6.22 6.21 6.20 4.20 4.20 4.19 4.18 4.18 4.17 3.79 3.62 3.60 3.58 3.58 3.58 3.56 3.51 3.50 3.49 3.48 2.74 2.73 2.72 2.72 2.71 2.71 2.70 2.69 2.65 2.64 2.63 2.63 2.62 2.61 2.60 2.05 (CD3)2CO 1.75 1.75 1.05 1.04 1.04 1.03 1.03 1.02
TIPS
Me
N
O
DMTO
NHBz
N
O
0.66 2.00 1.00 3.03 2.10 4.01 2.01 1.02 4.01 1.00 1.02 6.08 2.01 1.00 1.00 1.00 3.00 21.10
f1 (ppm)

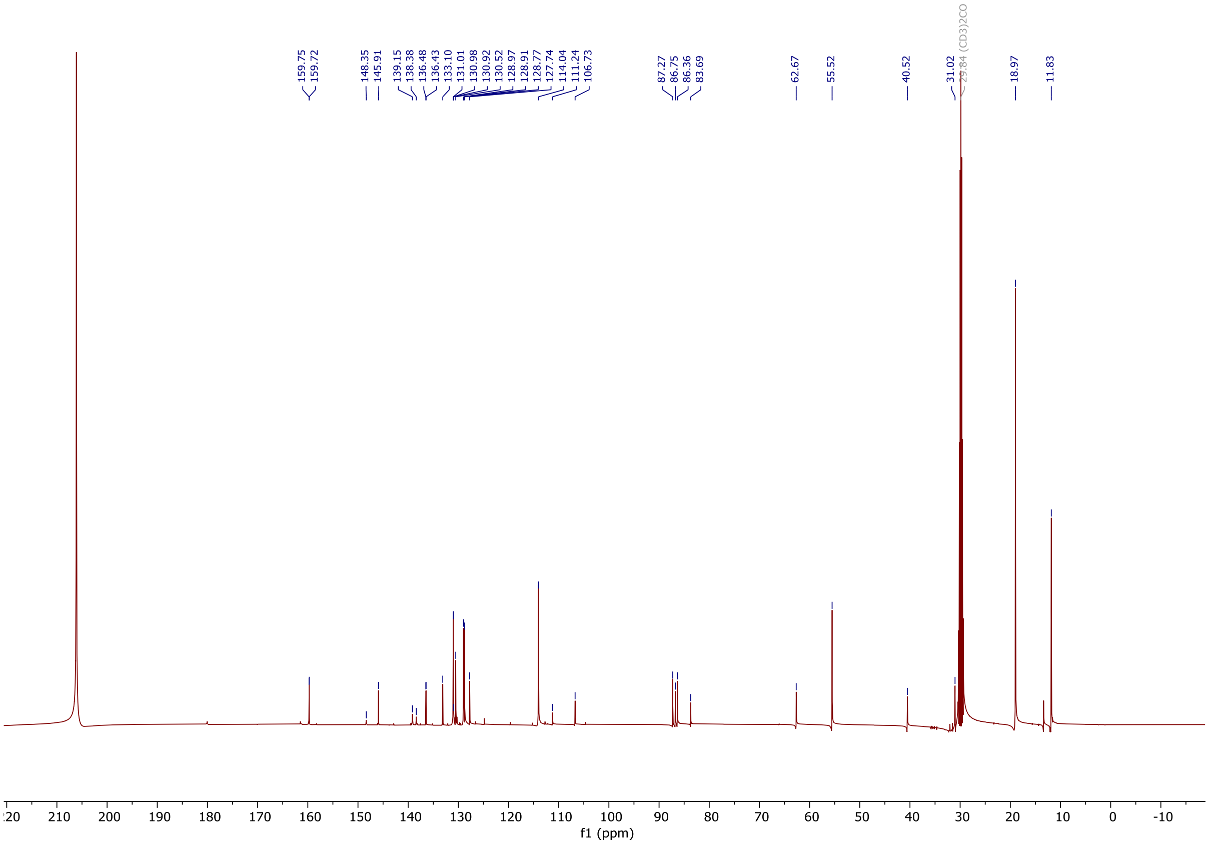
159.75 159.72 148.35 145.91 139.15 138.38 136.48 136.43 133.10 131.01 130.98 130.92 130.52 128.97 128.91 128.77 127.74 114.04 111.24 106.73 87.27 86.75 86.36 83.69 62.67 55.52 40.52 31.02 29.84 (CD3)2CO 18.97 11.83
f1 (ppm)

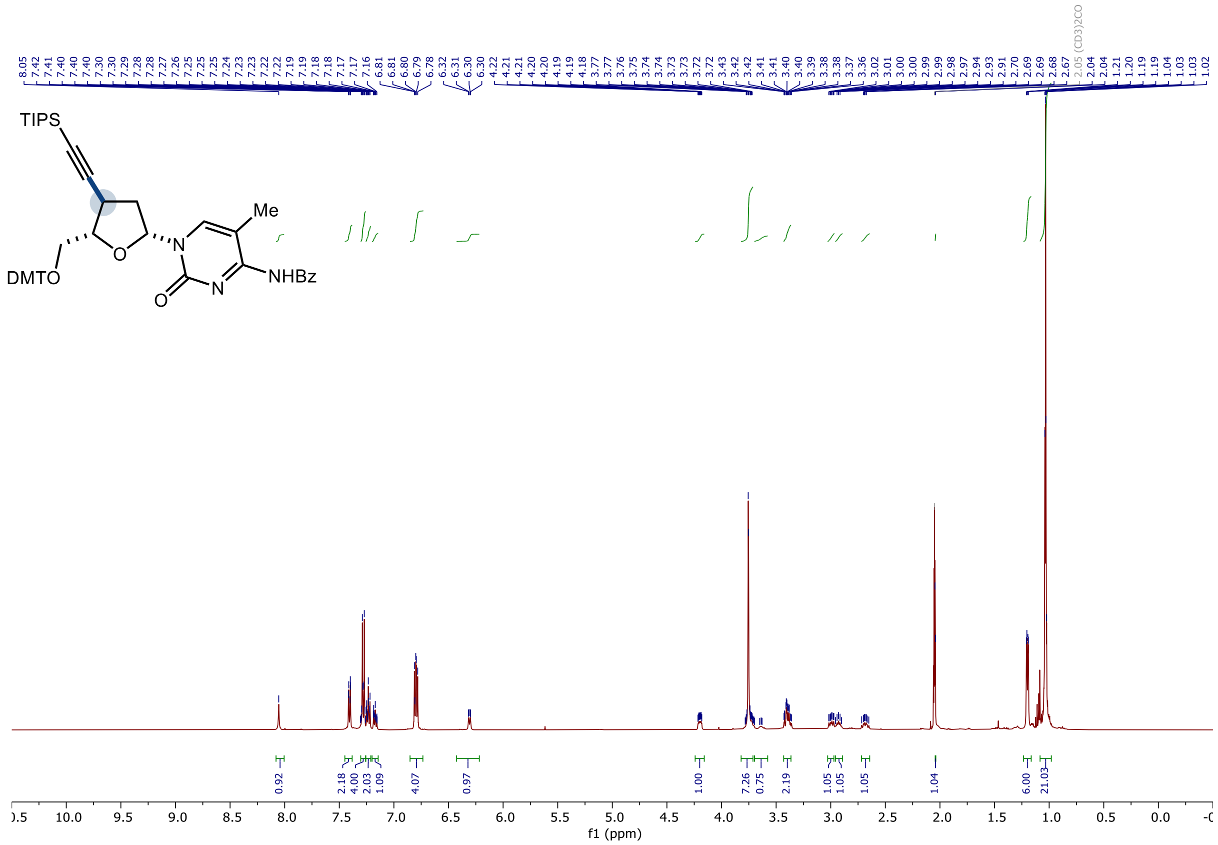

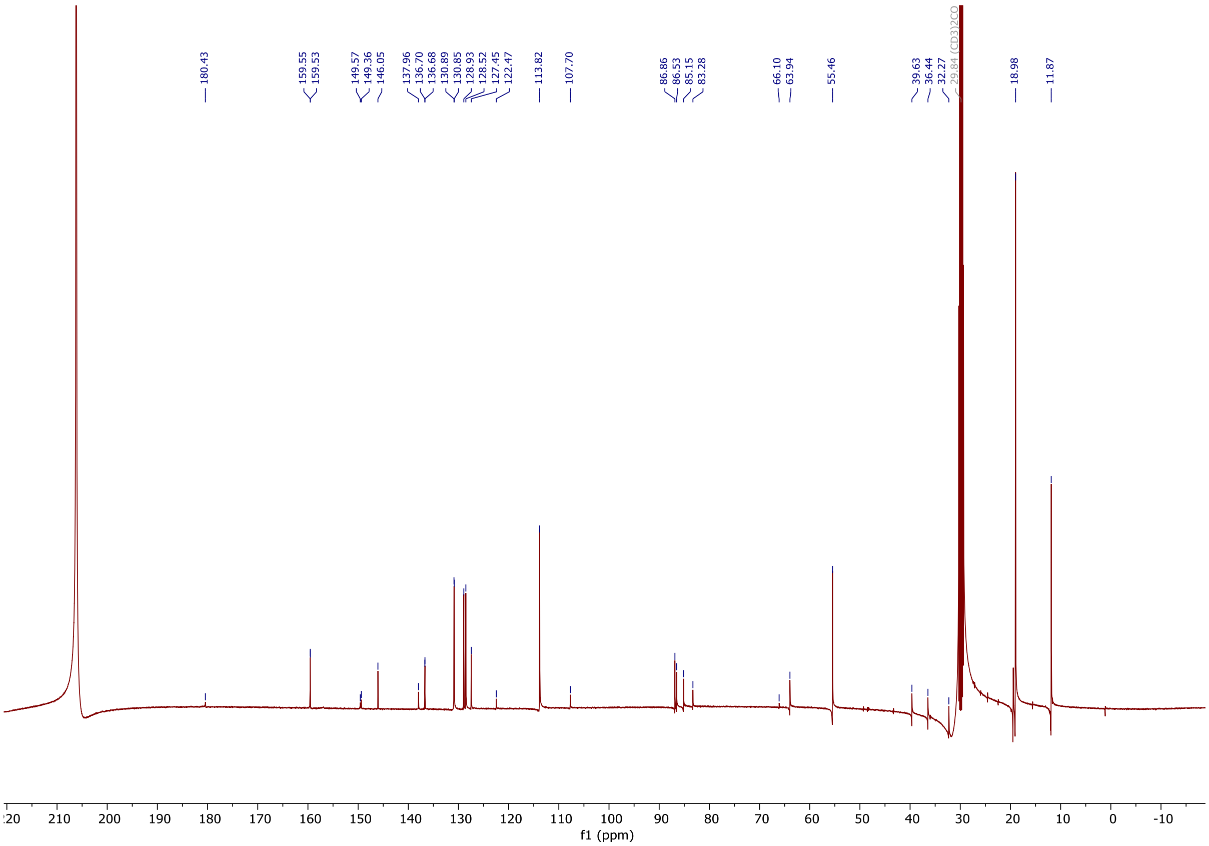

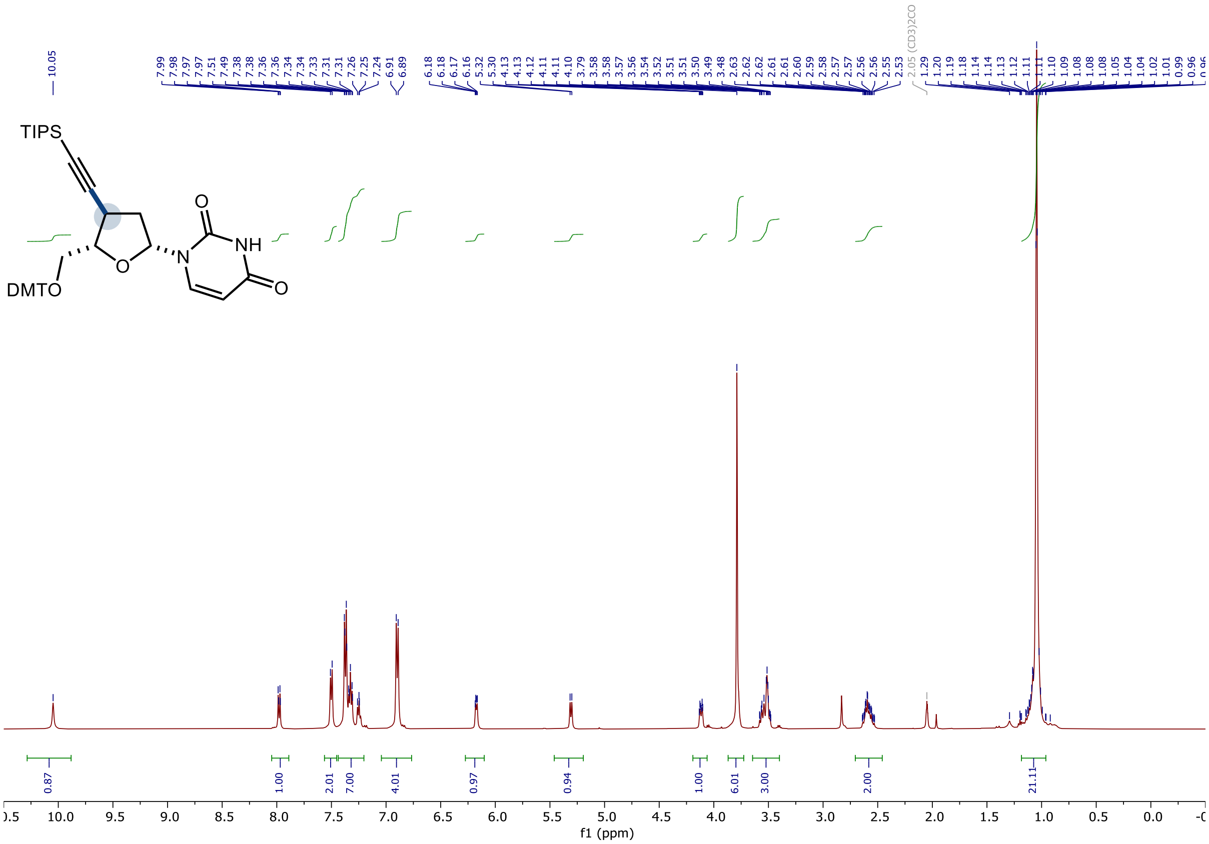
TIPS
O
NH
N
O
O
DMTO
10.05
7.99 7.98 7.97 7.97 7.51 7.49 7.38 7.38 7.36 7.36 7.34 7.34 7.33 7.31 7.31 7.26 7.25 7.24 6.91 6.89
6.18 6.18 6.17 6.16 5.32 5.30 4.13 4.13 4.12 4.11 4.11 4.10 3.79 3.58 3.58 3.57 3.56 3.54 3.52 3.51 3.51 3.50 3.49 3.48 2.63 2.62 2.62 2.61 2.61 2.60 2.59 2.58 2.57 2.57 2.56 2.56 2.55 2.53
2.05 (CD3)2CO
1.29 1.20 1.19 1.18 1.14 1.13 1.12 1.11 1.11 1.10 1.09 1.08 1.08 1.08 1.05 1.04 1.04 1.02 1.01 0.99
0.87 1.00 2.01 7.00 4.01 0.97 0.94 1.00 6.01 3.00 2.00 21.11
10.0 9.5 9.0 8.5 8.0 7.5 7.0 6.5 6.0 5.5 5.0 4.5 4.0 3.5 3.0 2.5 2.0 1.5 1.0 0.5 0.0
f1 (ppm)

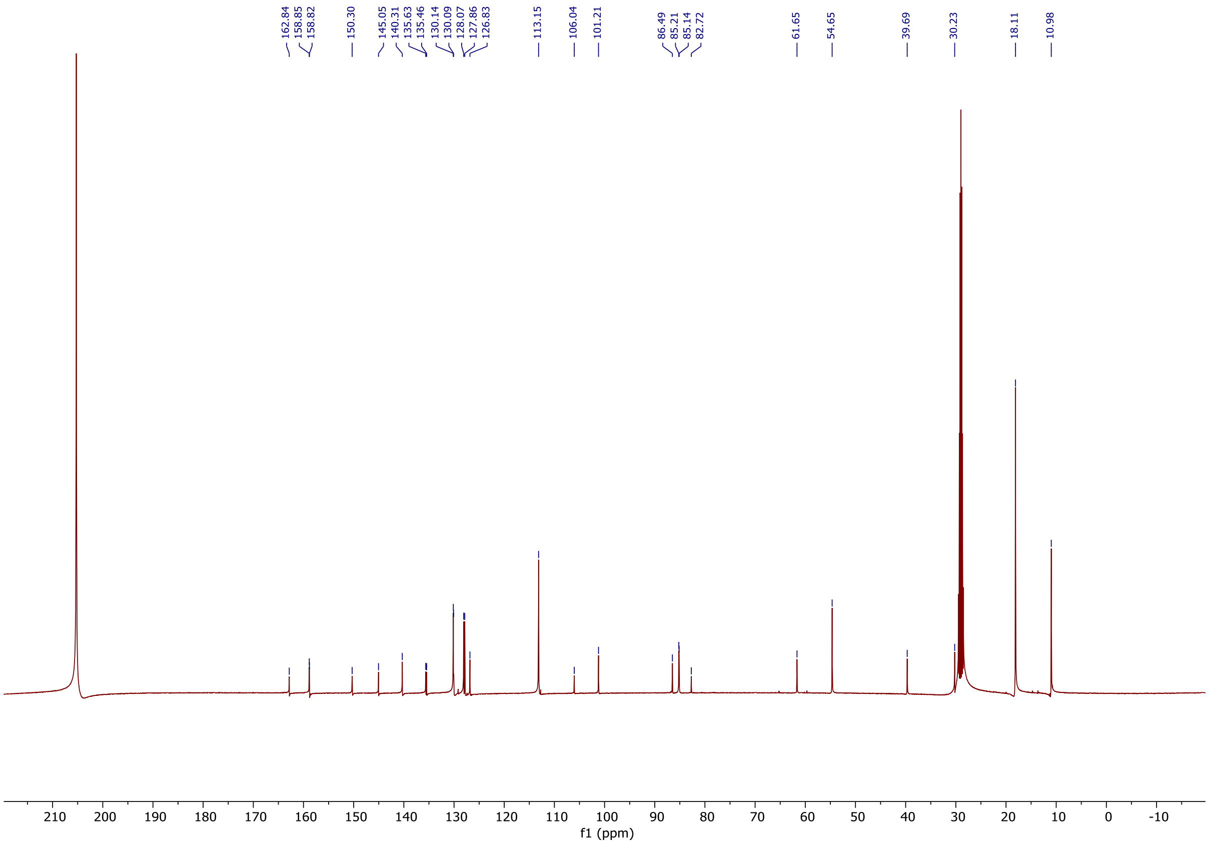
162.84 158.85 158.82 150.30 145.05 140.31 135.63 135.46 130.14 130.09 128.07 127.86 126.83 113.15 106.04 101.21 86.49 85.21 85.14 82.72 61.65 54.65 39.69 30.23 18.11 10.98
210 200 190 180 170 160 150 140 130 120 110 100 90 80 70 60 50 40 30 20 10 0 -10
f1 (ppm)

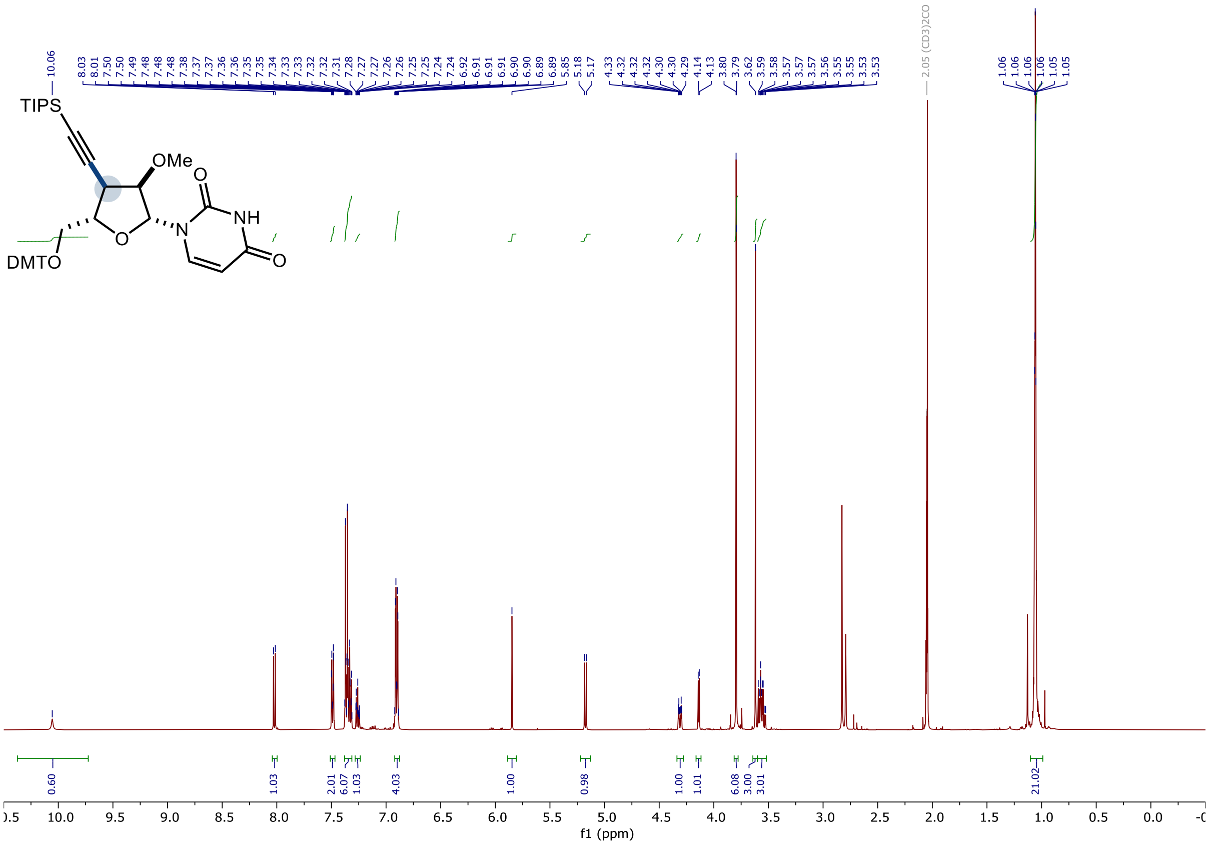

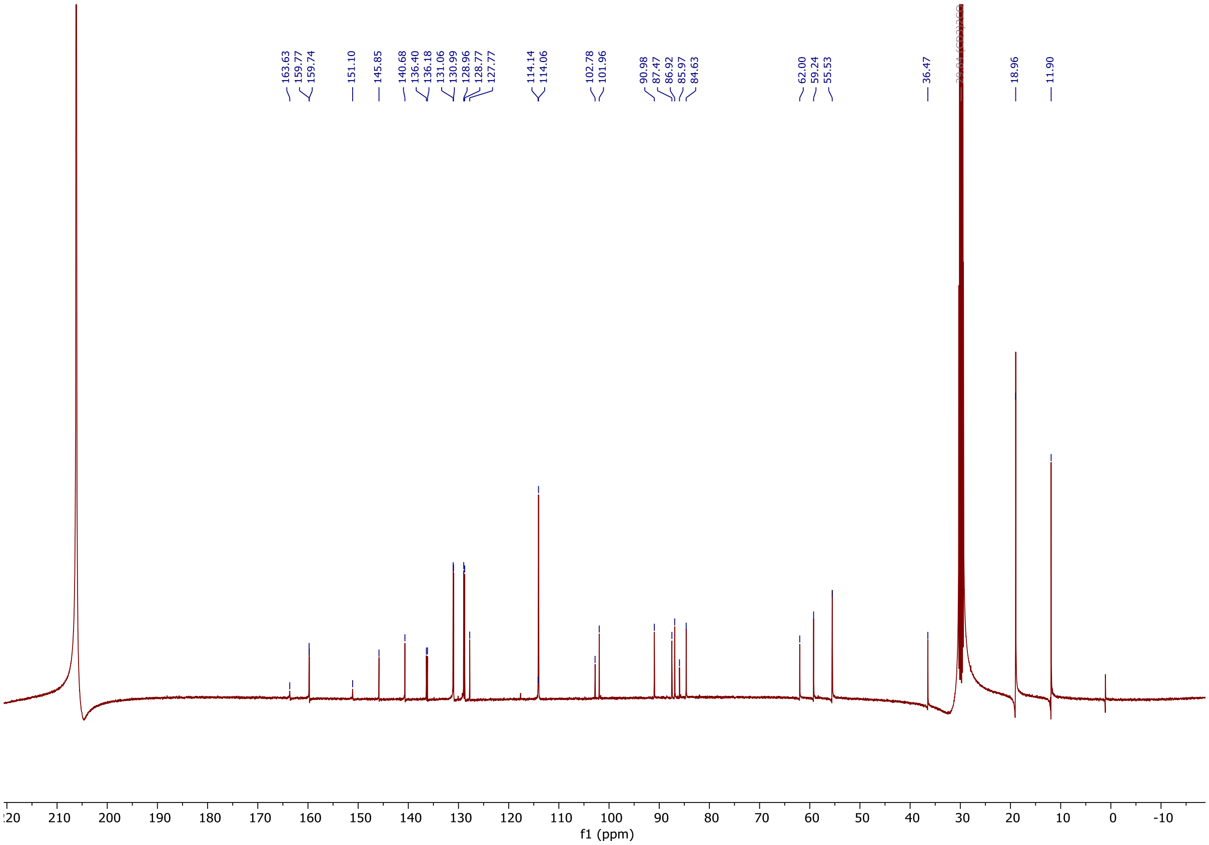

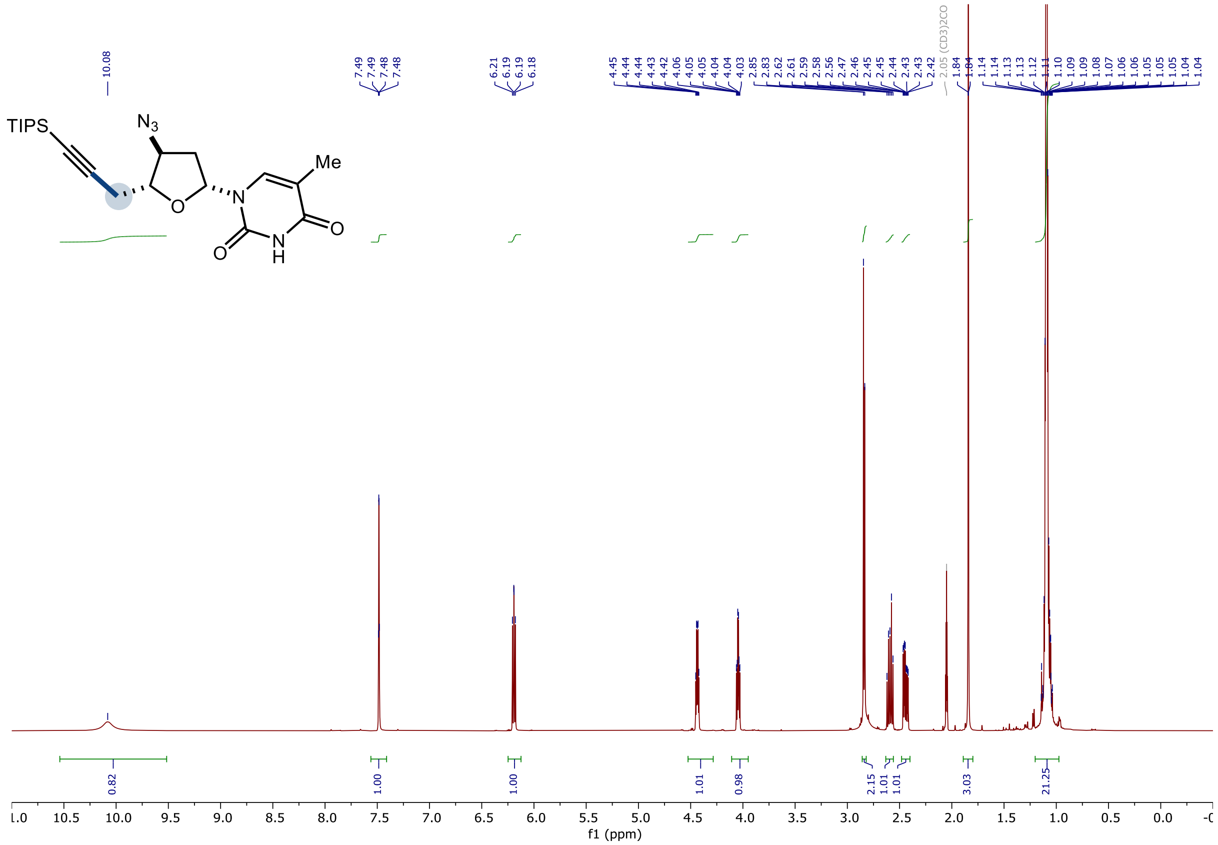
TIPS
N3
Me
O
N
NH
H
10.08
7.49 7.49 7.48 7.48
6.21 6.19 6.19 6.18
4.45 4.44 4.44 4.43 4.42 4.06 4.05 4.05 4.04 4.04 4.03
2.85 2.83 2.62 2.61 2.59 2.58 2.56 2.47 2.46 2.45 2.45 2.44 2.43 2.43 2.42
2.05 (CD3)2CO
1.84 1.84
1.14 1.14 1.13 1.13 1.12 1.11 1.10 1.09 1.09 1.08 1.07 1.06 1.06 1.05 1.05 1.05 1.04 1.04
0.82
1.00
1.00
1.01
0.98
2.15
1.01
1.01
3.03
21.25
f1 (ppm)

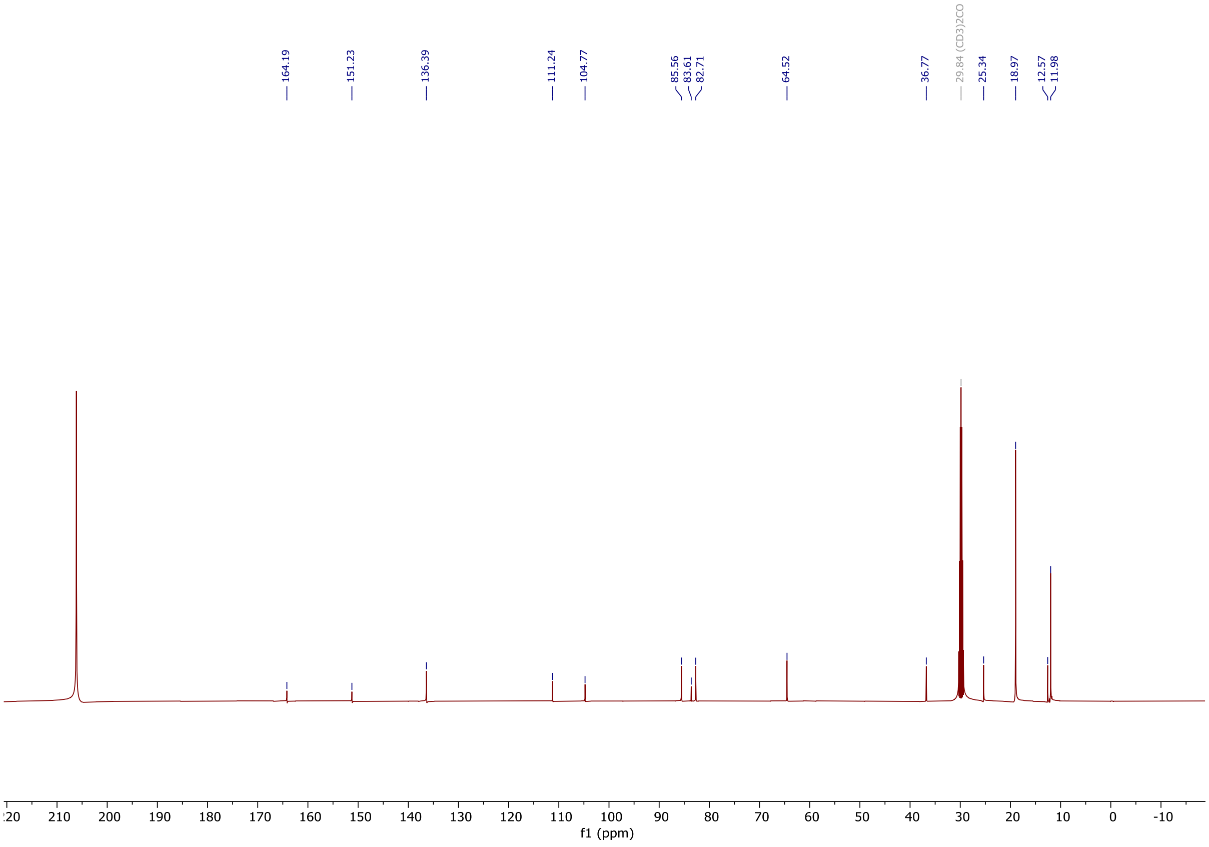
164.19
151.23
136.39
111.24
104.77
85.56 83.61 82.71
64.52
36.77
29.84 (CD3)2CO
25.34
18.97
12.57 11.98
f1 (ppm)

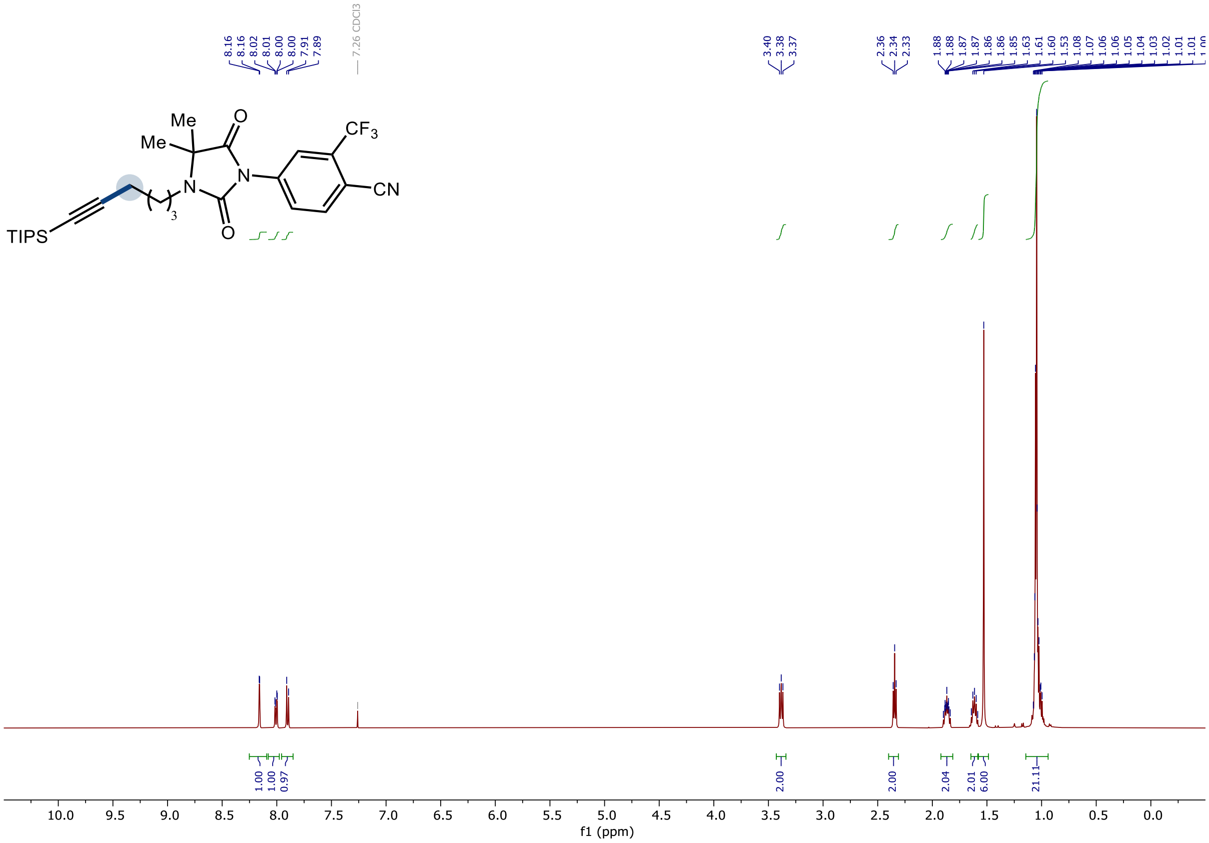
8.16
8.16
8.02
8.01
8.00
8.00
7.91
7.89
7.26 CDCl3
3.40
3.38
3.37
2.36
2.34
2.33
1.88
1.88
1.87
1.87
1.86
1.86
1.85
1.63
1.61
1.60
1.53
1.08
1.07
1.06
1.06
1.05
1.04
1.03
1.02
1.01
1.01
Me
Me
O
CF3
N
N
CN
TIPS
3
O
1.00
1.00
0.97
2.00
2.00
2.04
2.01
6.00
21.11
f1 (ppm)

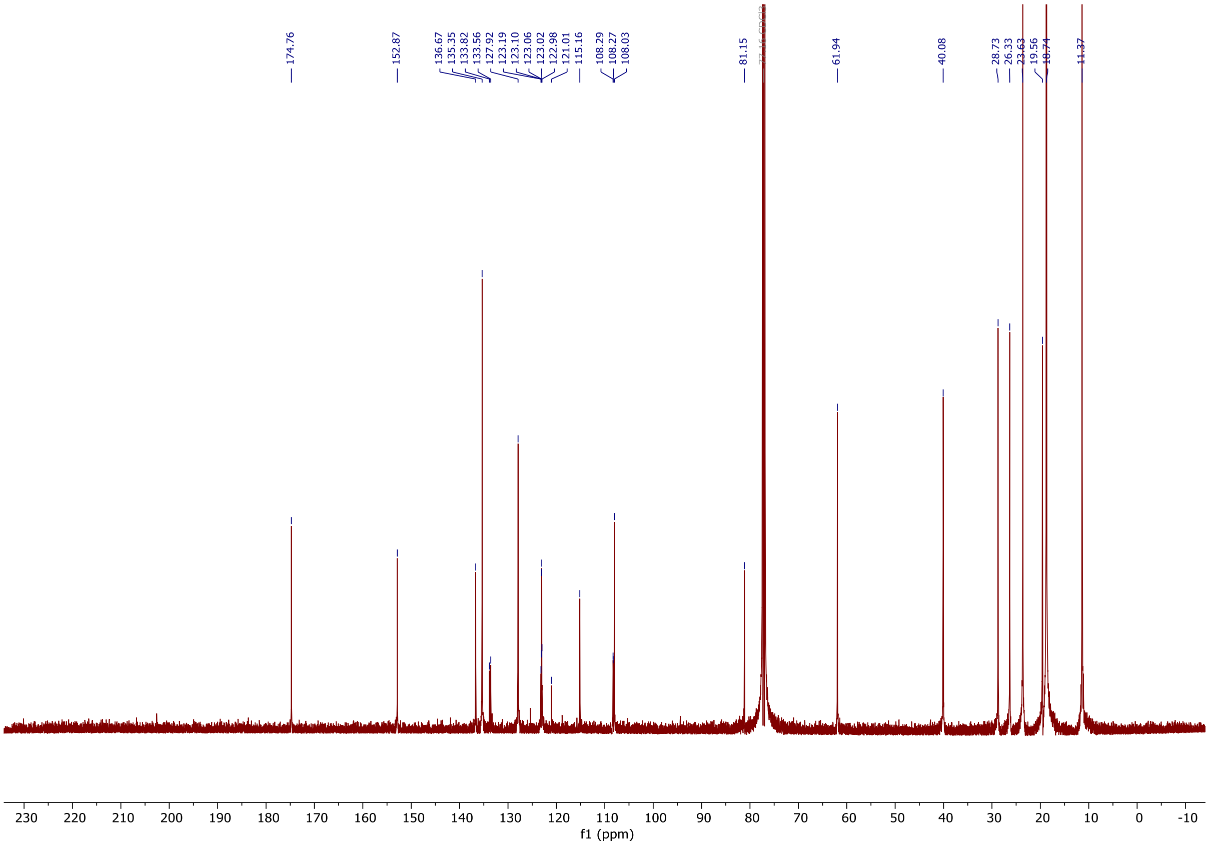
174.76
152.87
136.67
135.35
133.82
133.56
127.92
123.19
123.10
123.06
123.02
122.98
121.01
115.16
108.29
108.27
108.03
81.15
61.94
40.08
28.73
26.33
23.63
19.56
18.74
11.37
f1 (ppm)

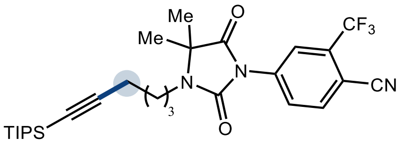

Me
Me
O
N
N
O
CF3
CN
TIPS
3

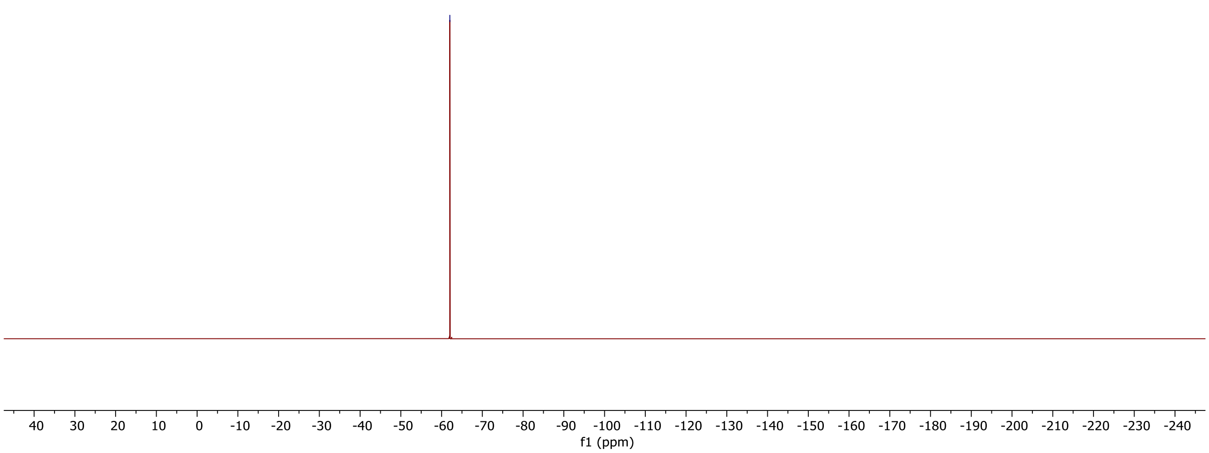

-62.03
40
30
20
10
0
-10
-20
-30
-40
-50
-60
-70
-80
-90
-100
-110
-120
-130
-140
-150
-160
-170
-180
-190
-200
-210
-220
-230
-240
f1 (ppm)

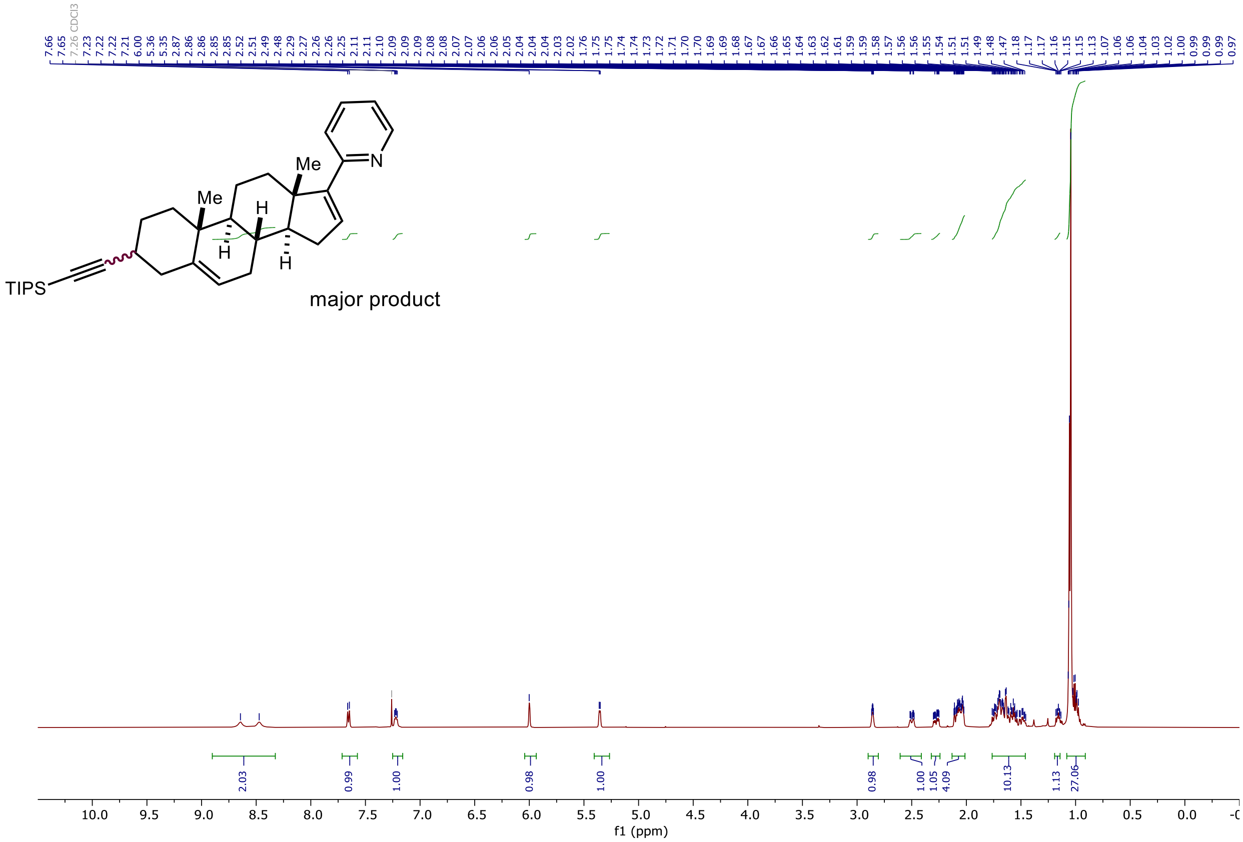
7.66 7.65 7.26 CDCl3 7.23 7.22 7.22 7.21 6.00 5.36 5.35 2.87 2.86 2.86 2.85 2.85 2.52 2.51 2.49 2.48 2.29 2.27 2.26 2.25 2.11 2.11 2.10 2.09 2.09 2.09 2.08 2.08 2.07 2.07 2.06 2.06 2.05 2.04 2.04 2.04 2.03 2.02 1.76 1.75 1.75 1.74 1.74 1.73 1.72 1.71 1.70 1.70 1.69 1.69 1.68 1.67 1.67 1.66 1.65 1.64 1.63 1.62 1.61 1.59 1.59 1.58 1.57 1.56 1.56 1.55 1.54 1.51 1.51 1.49 1.48 1.47 1.18 1.17 1.17 1.16 1.15 1.15 1.13 1.07 1.06 1.06 1.04 1.03 1.02 1.00 0.99 0.99 0.97
Me
Me
H
H
H
N
TIPS
major product
2.03 0.99 1.00 0.98 1.00 0.98 1.00 1.05 4.09 10.13 1.13 27.06
f1 (ppm)

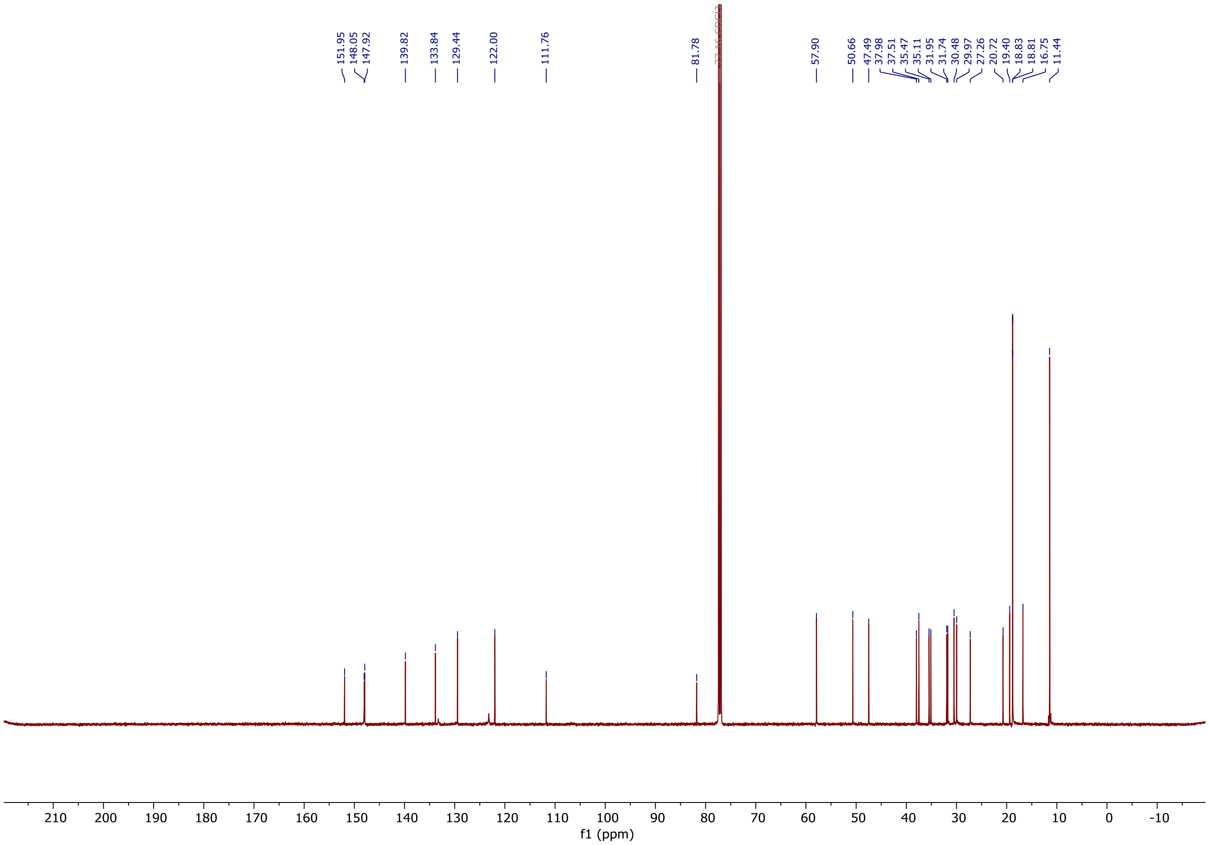
151.95 148.05 147.92 139.82 133.84 129.44 122.00 111.76 81.78 57.90 50.66 47.49 37.98 37.51 35.47 35.11 31.95 31.74 30.48 29.97 27.26 20.72 19.40 18.83 18.81 16.75 11.44
f1 (ppm)

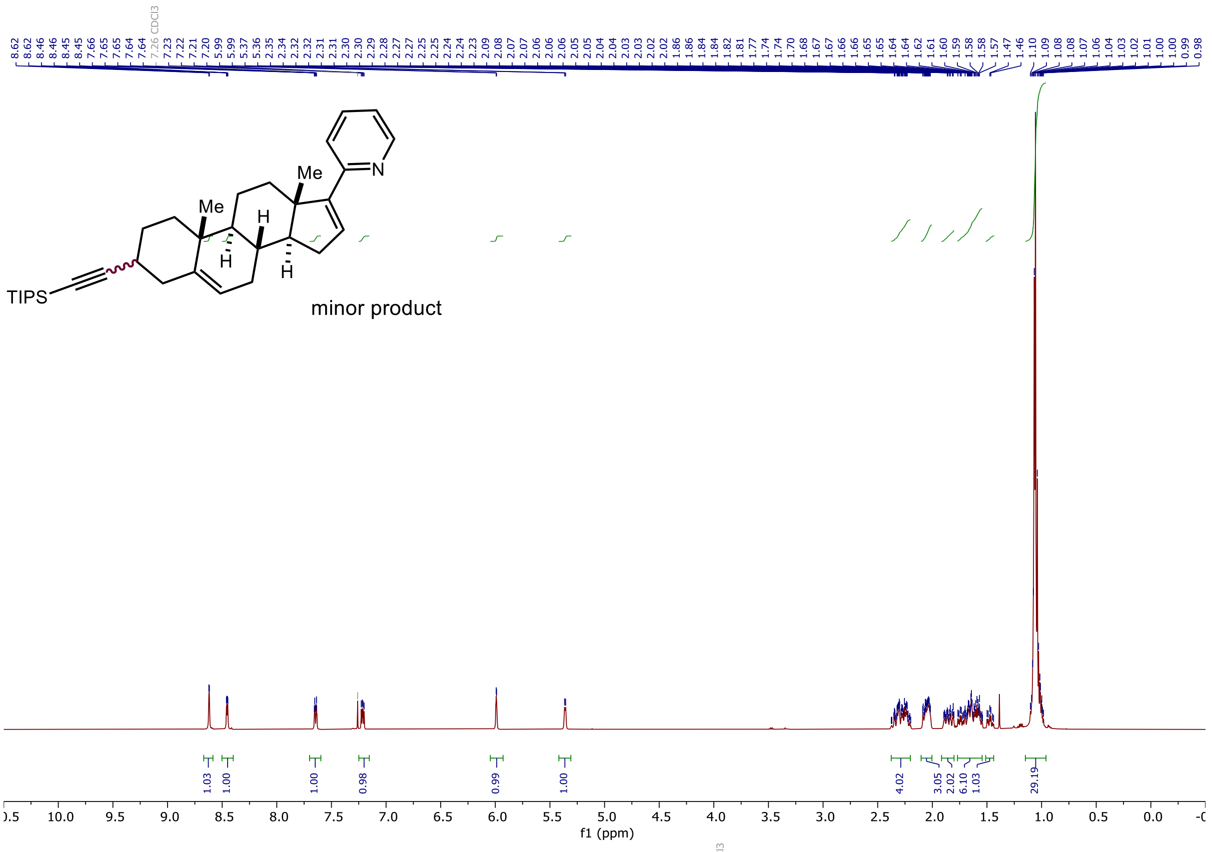
minor product
TIPS
Me
Me
H
H
H
N
7.26 CDCl3
f1 (ppm)

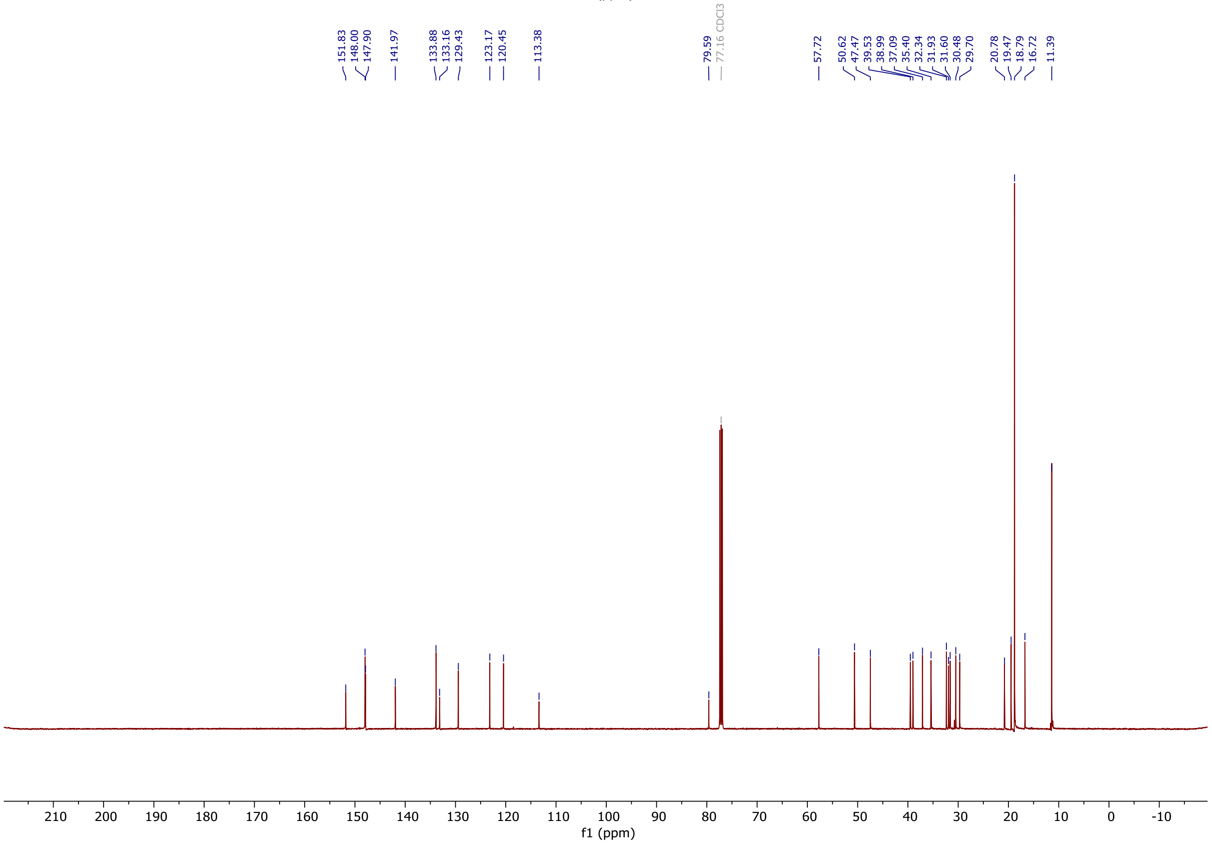
151.83
148.00
147.90
141.97
133.88
133.16
129.43
123.17
120.45
113.38
79.59
77.16 CDCl3
57.72
50.62
47.47
39.53
38.99
37.09
35.40
32.34
31.93
31.60
30.48
29.70
20.78
19.47
18.79
16.72
11.39
f1 (ppm)

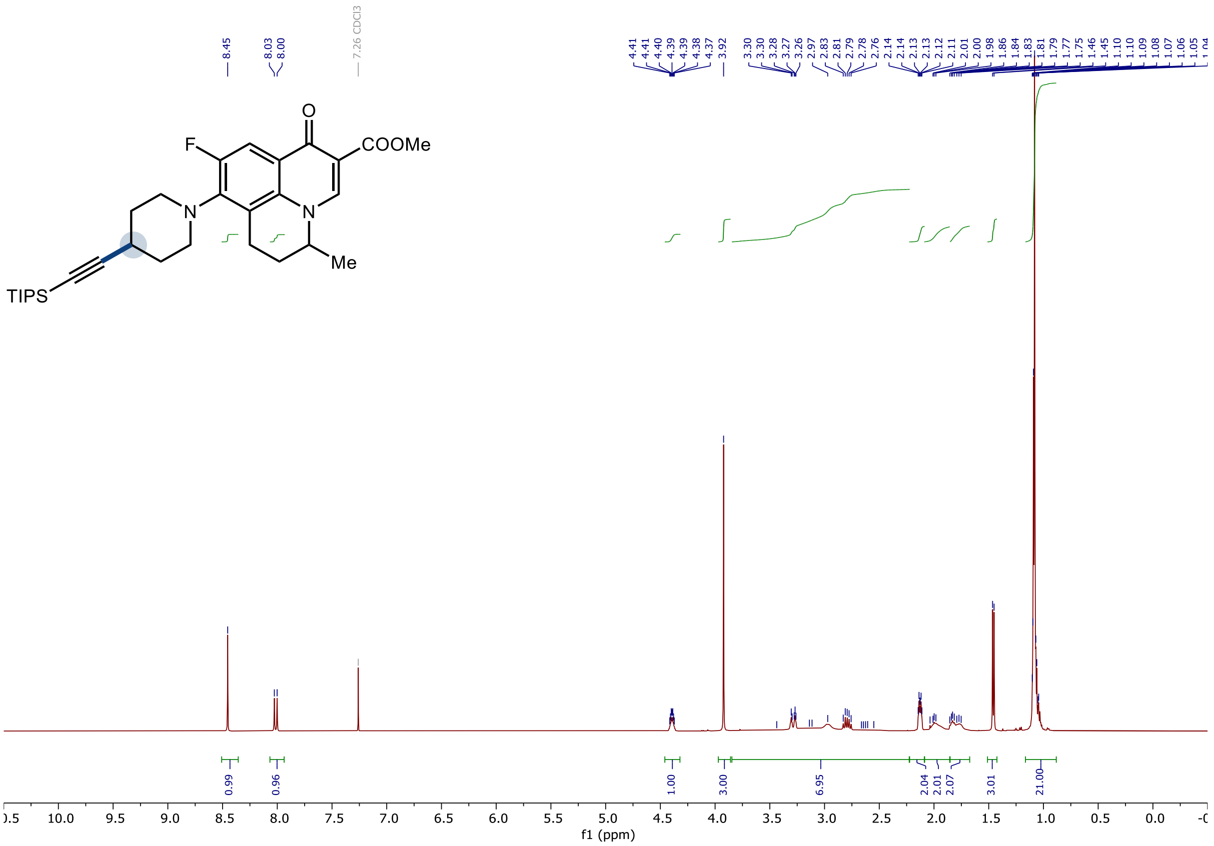
F
O
COOMe
N
N
Me
TIPS
8.45
8.03
8.00
7.26 CDCl3
3.92
0.99
0.96
1.00
3.00
6.95
2.04
2.01
2.07
3.01
21.00
f1 (ppm)

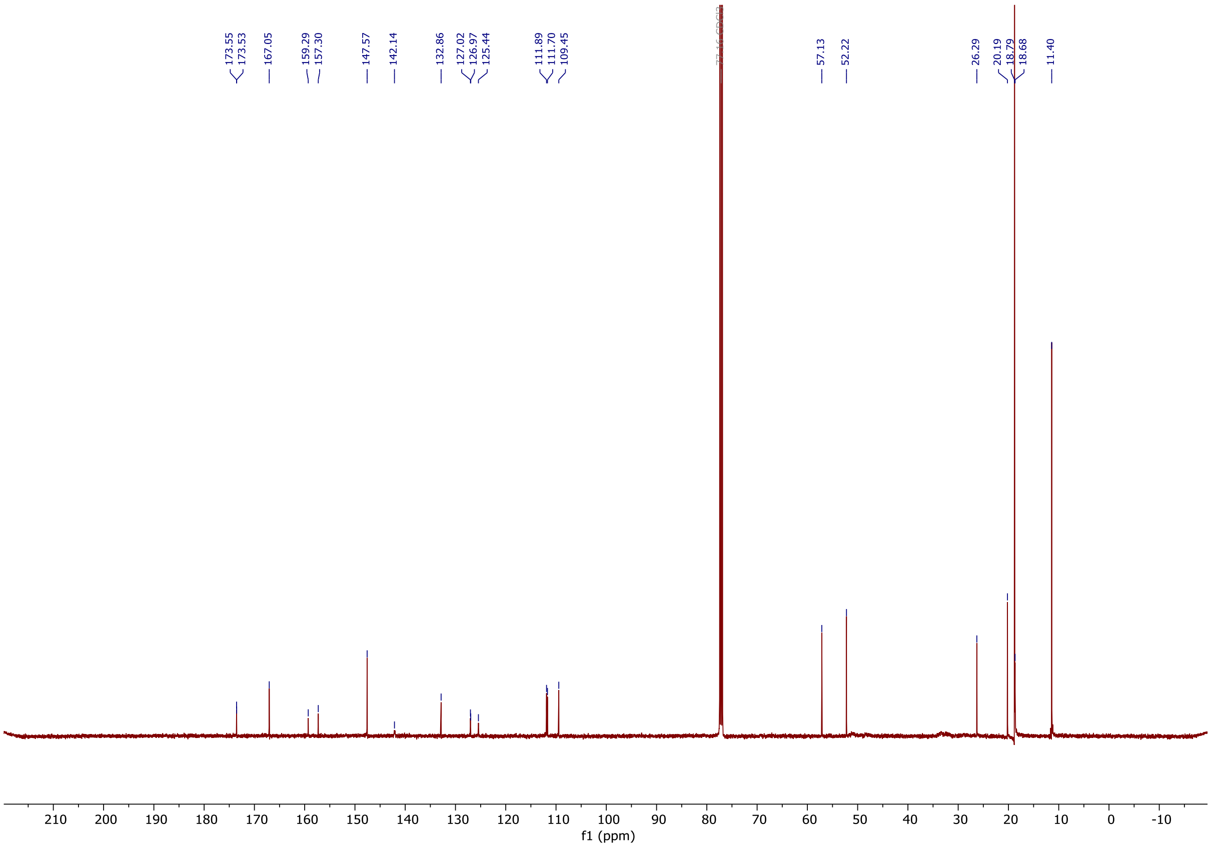
173.55
173.53
167.05
159.29
157.30
147.57
142.14
132.86
127.02
126.97
125.44
111.89
111.70
109.45
57.13
52.22
26.29
20.19
18.79
18.68
11.40
f1 (ppm)

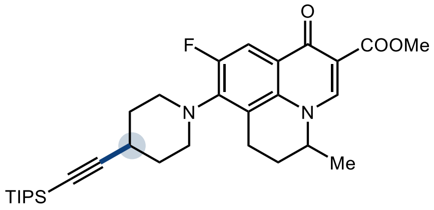

O
F
COOMe
N
N
Me
TIPS

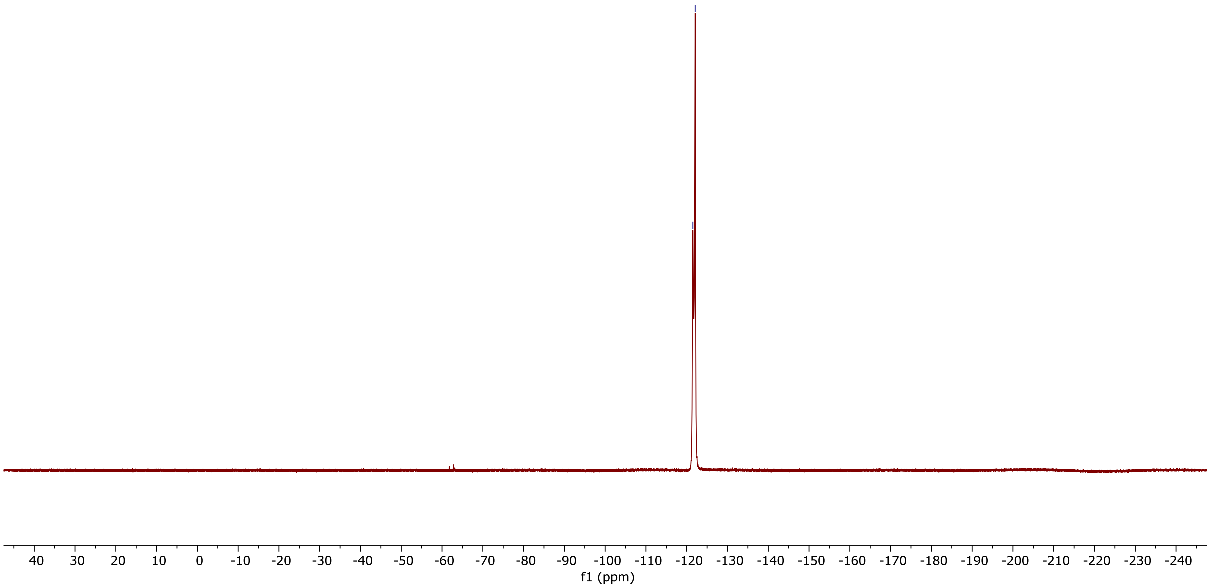

-121.52
-122.07
40 30 20 10 0 -10 -20 -30 -40 -50 -60 -70 -80 -90 -100 -110 -120 -130 -140 -150 -160 -170 -180 -190 -200 -210 -220 -230 -240
f1 (ppm)

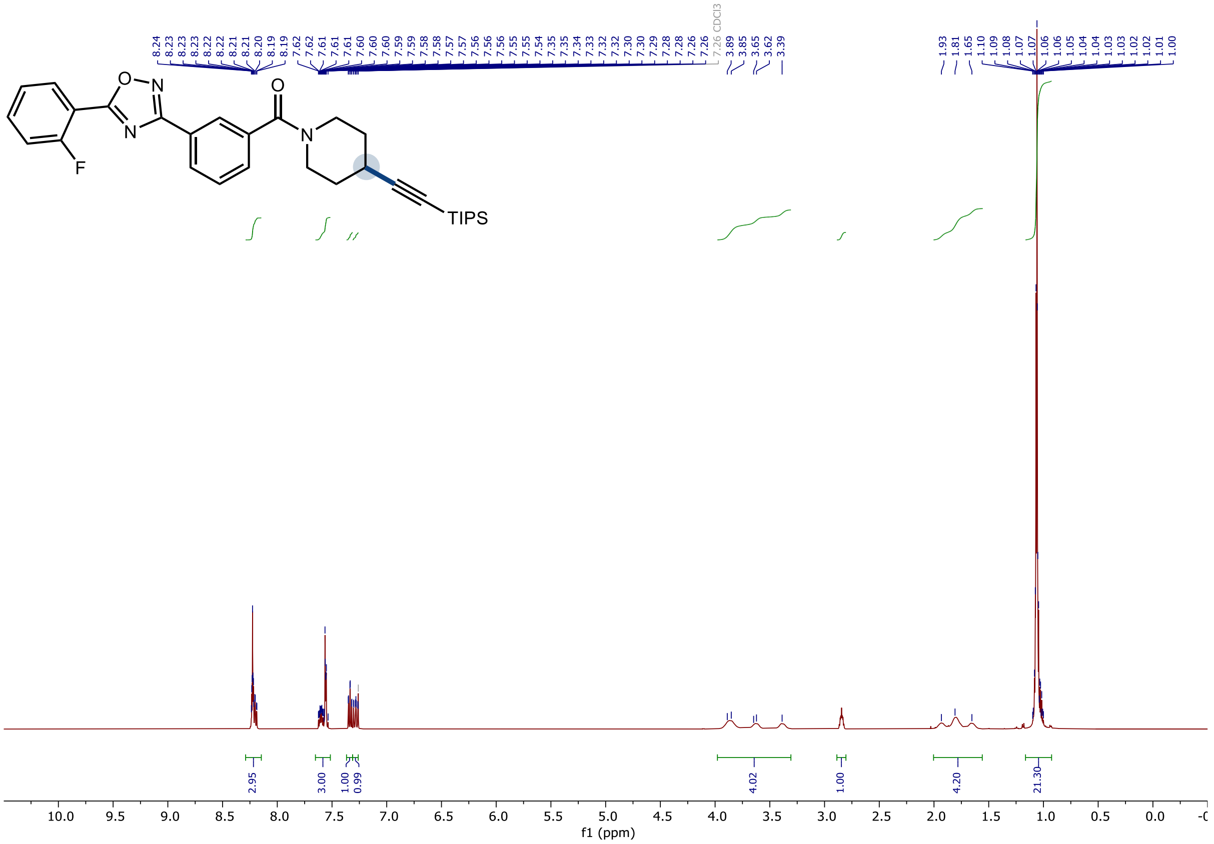
TIPS
f1 (ppm)

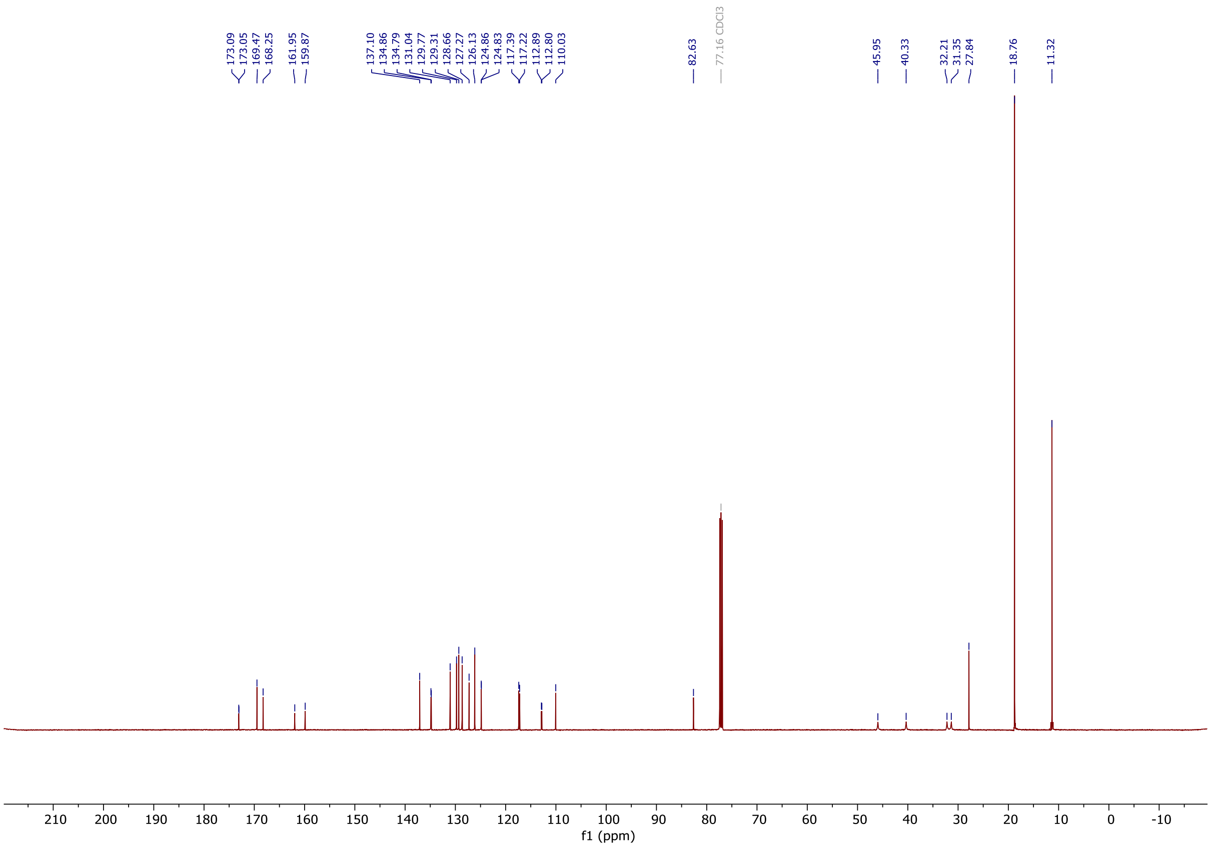
f1 (ppm)

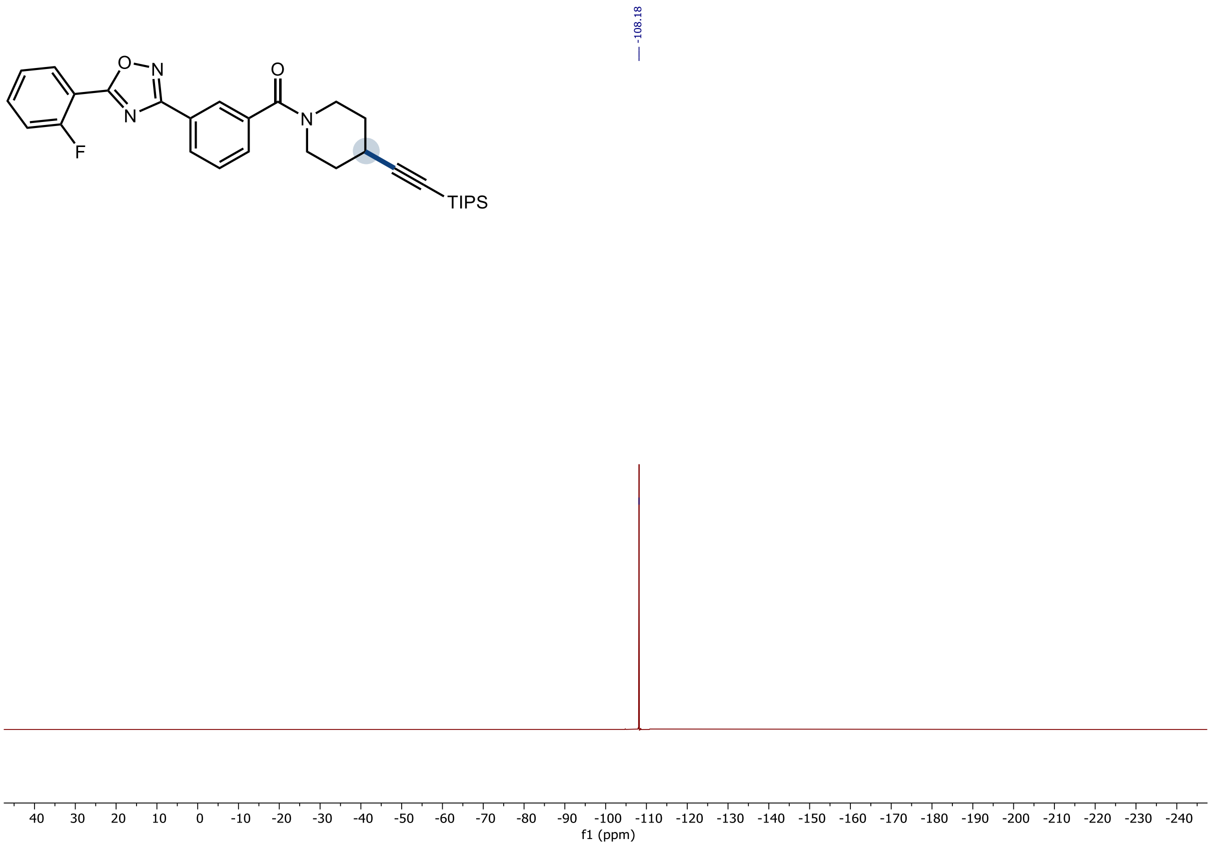

−108.18
O
N
F
TIPS
40 30 20 10 0 -10 -20 -30 -40 -50 -60 -70 -80 -90 -100 -110 -120 -130 -140 -150 -160 -170 -180 -190 -200 -210 -220 -230 -240
f1 (ppm)

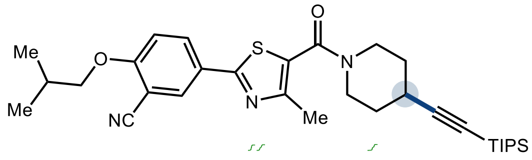

Me
O
Me
NC
S
N
Me
O
N
TIPS

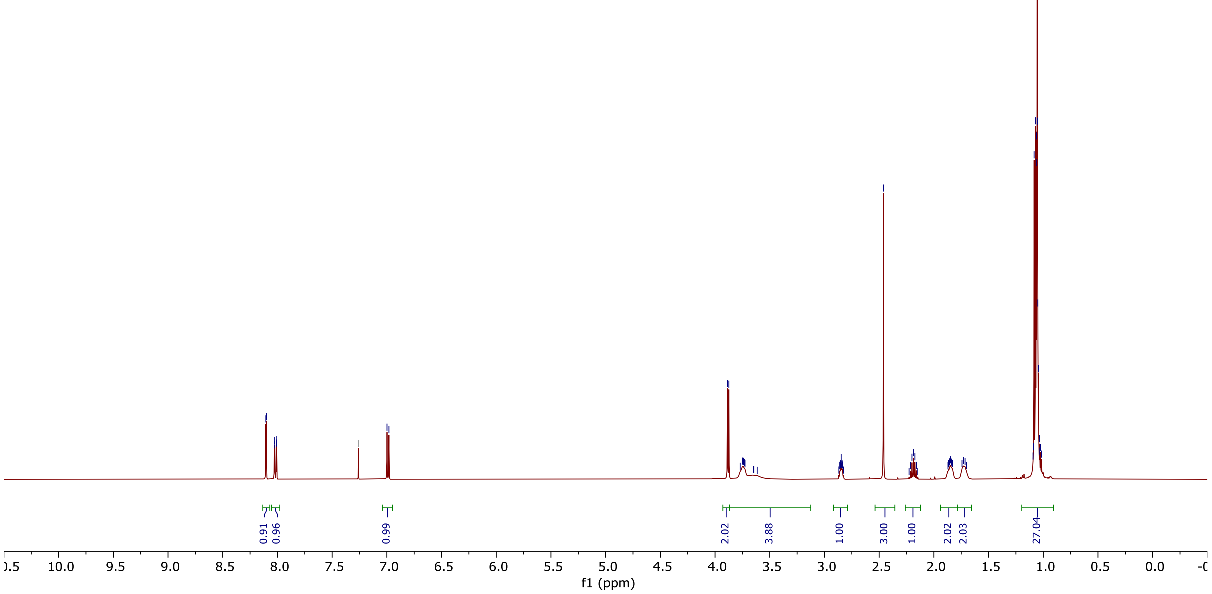

8.11
8.10
8.03
8.02
8.01
8.01
7.26 CDCl3
7.00
6.98
3.89
3.87
2.46
0.91
0.96
0.99
2.02
3.88
1.00
3.00
1.00
2.02
2.03
27.04
f1 (ppm)

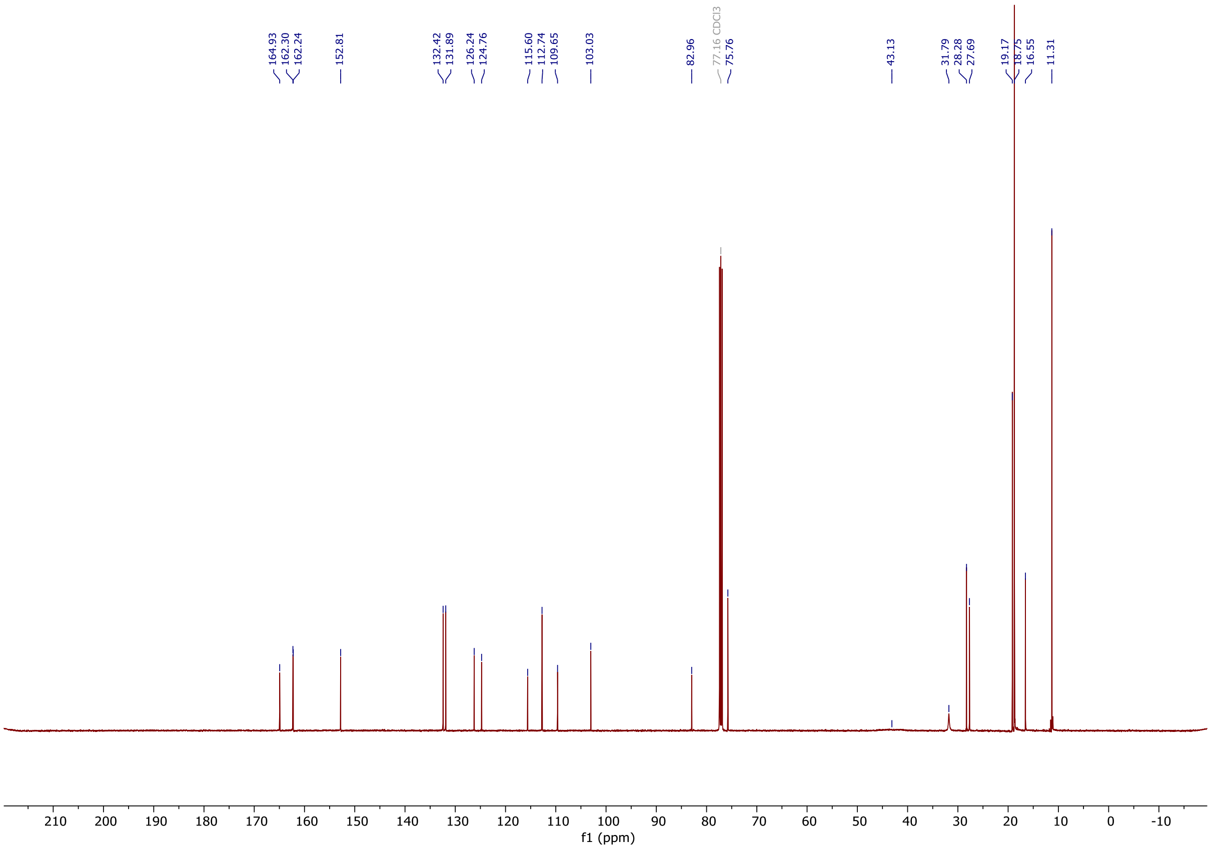

164.93
162.30
162.24
152.81
132.42
131.89
126.24
124.76
115.60
112.74
109.65
103.03
82.96
77.16 CDCl3
75.76
43.13
31.79
28.28
27.69
19.17
18.75
16.55
11.31
f1 (ppm)

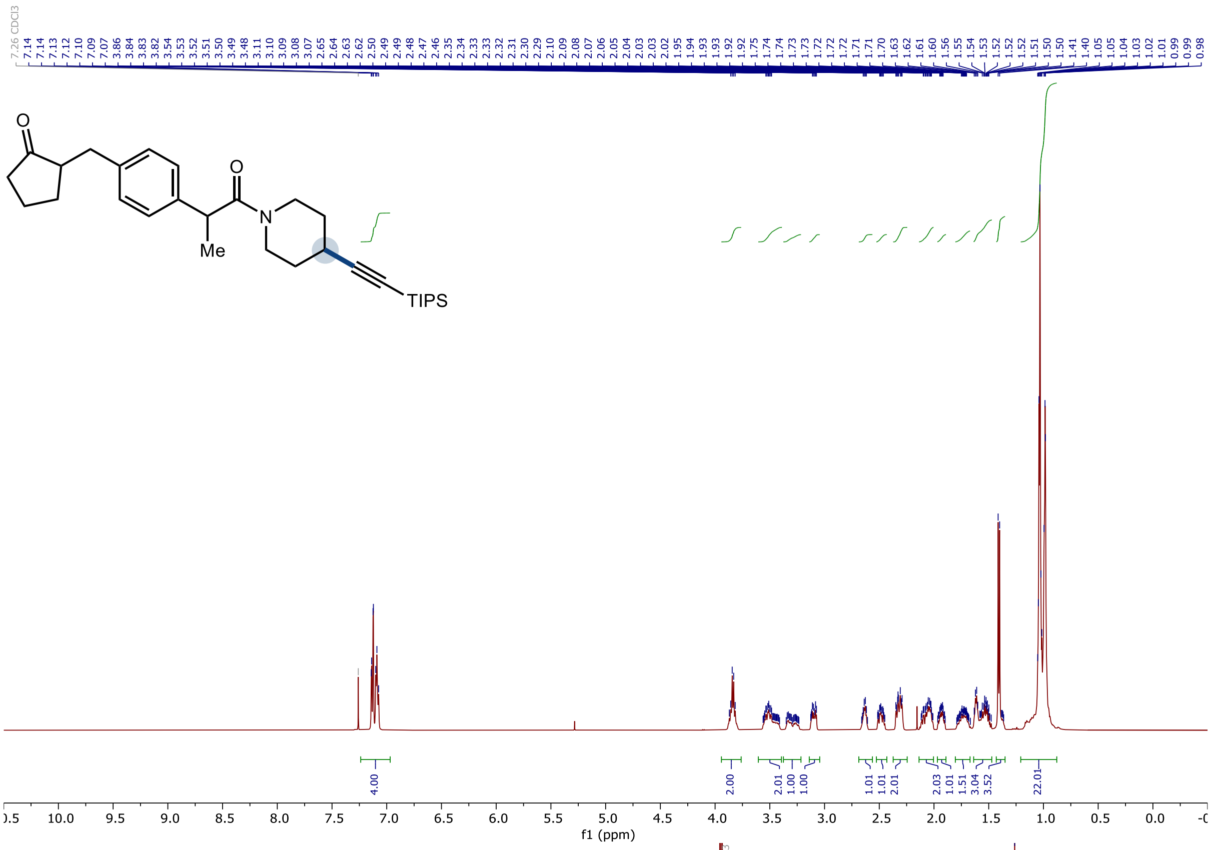
O
O
N
Me
TIPS
7.26 CDCl3
7.14 7.14 7.13 7.12 7.10 7.09 7.07 3.86 3.84 3.83 3.82 3.54 3.53 3.52 3.51 3.50 3.49 3.48 3.11 3.10 3.09 3.08 3.07 2.65 2.64 2.63 2.62 2.50 2.49 2.49 2.48 2.47 2.46 2.35 2.34 2.33 2.33 2.32 2.31 2.30 2.29 2.10 2.09 2.08 2.07 2.06 2.05 2.04 2.03 2.03 2.02 1.95 1.94 1.93 1.93 1.92 1.92 1.75 1.74 1.74 1.73 1.73 1.72 1.72 1.72 1.71 1.71 1.70 1.63 1.62 1.61 1.60 1.56 1.55 1.54 1.53 1.52 1.52 1.52 1.51 1.50 1.50 1.41 1.40 1.05 1.05 1.04 1.03 1.02 1.01 0.99 0.98
4.00
2.00
2.01
1.00
1.00
1.01
1.01
2.01
2.03
1.01
1.51
3.04
3.52
22.01
10.0 9.5 9.0 8.5 8.0 7.5 7.0 6.5 6.0 5.5 5.0 4.5 4.0 3.5 3.0 2.5 2.0 1.5 1.0 0.5 0.0
f1 (ppm)

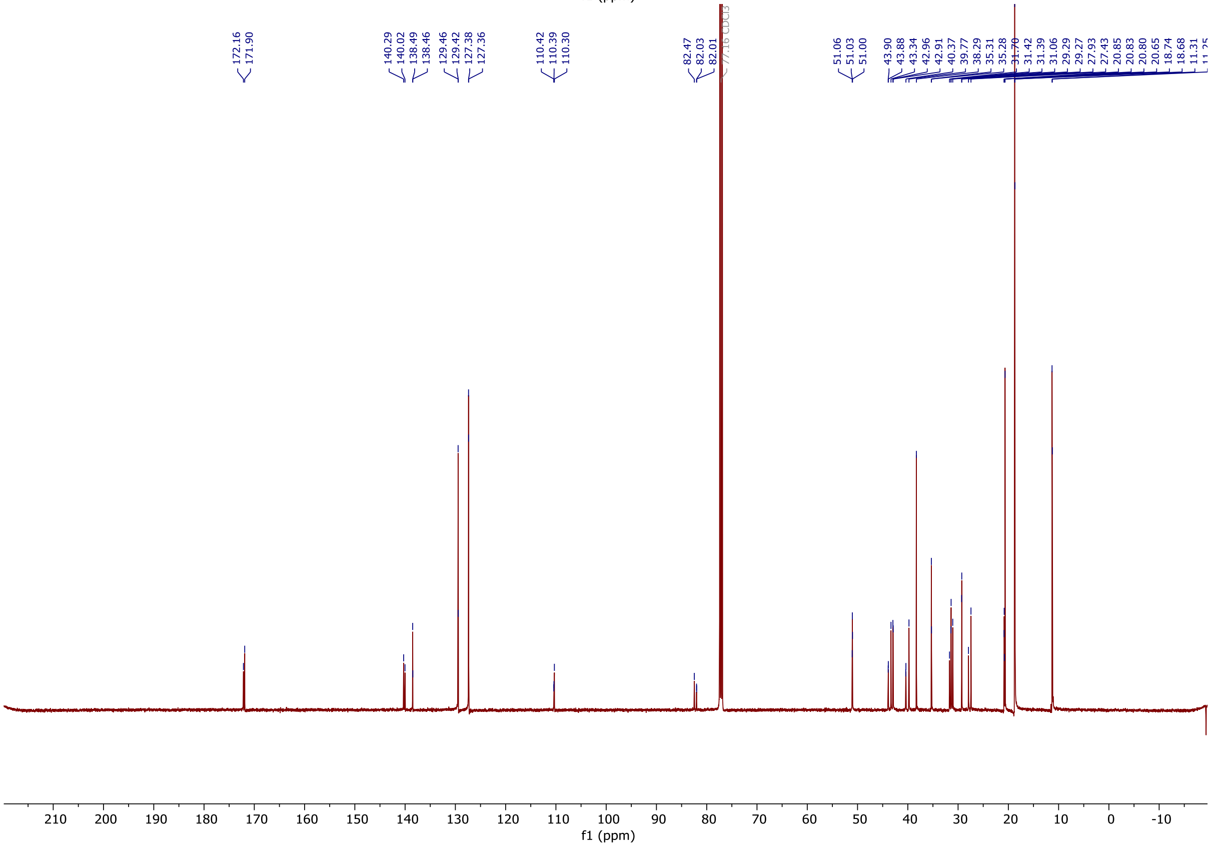
172.16
171.90
140.29
140.02
138.49
138.46
129.46
129.42
127.38
127.36
110.42
110.39
110.30
82.47
82.03
82.01
77.16 CDCl3
51.06
51.03
51.00
43.90
43.88
43.34
42.96
42.91
40.37
39.77
38.29
35.31
35.28
31.70
31.42
31.39
31.06
29.29
29.27
27.93
27.43
20.85
20.83
20.80
20.65
18.74
18.68
11.31
210 200 190 180 170 160 150 140 130 120 110 100 90 80 70 60 50 40 30 20 10 0 -10
f1 (ppm)

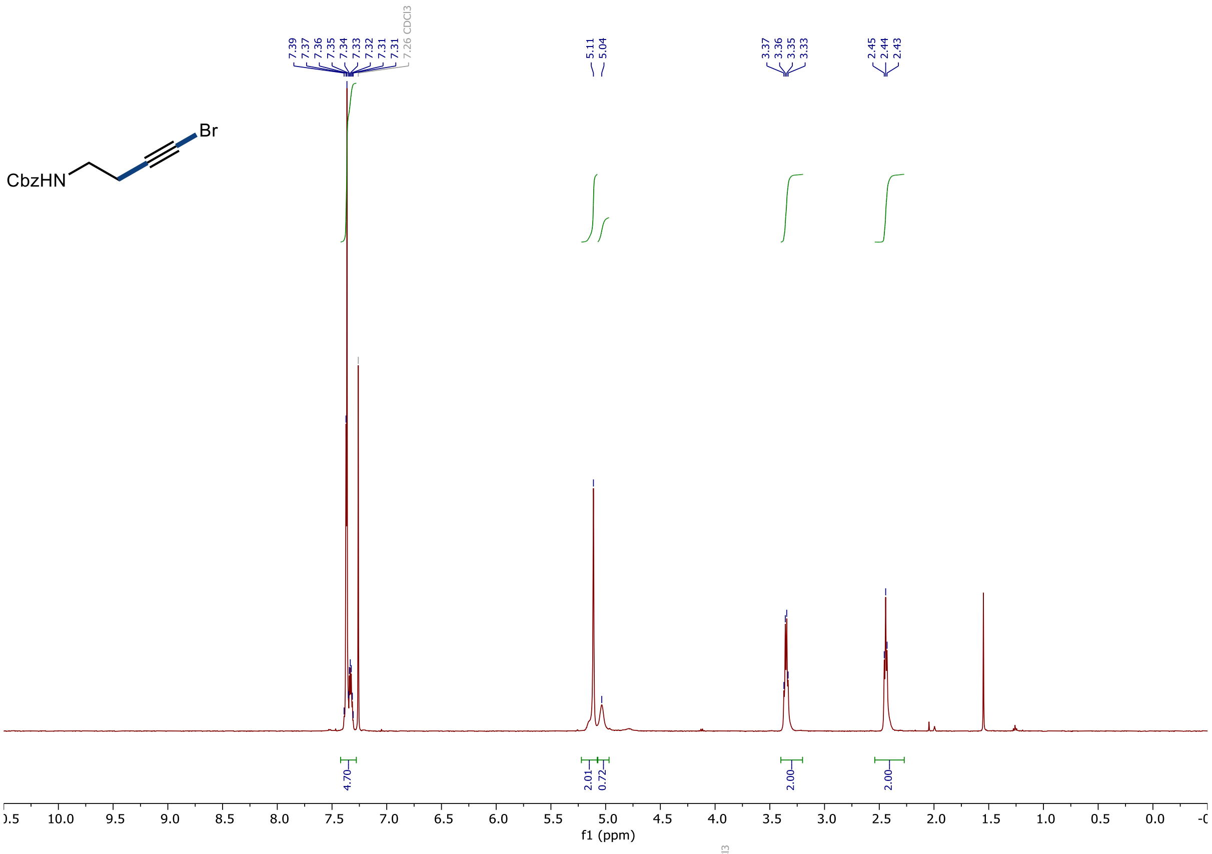

CbzHN
Br
7.39
7.37
7.36
7.35
7.34
7.33
7.32
7.31
7.31
7.26 CDCl3
5.11
5.04
3.37
3.36
3.35
3.33
2.45
2.44
2.43
4.70
2.01
0.72
2.00
2.00
f1 (ppm)

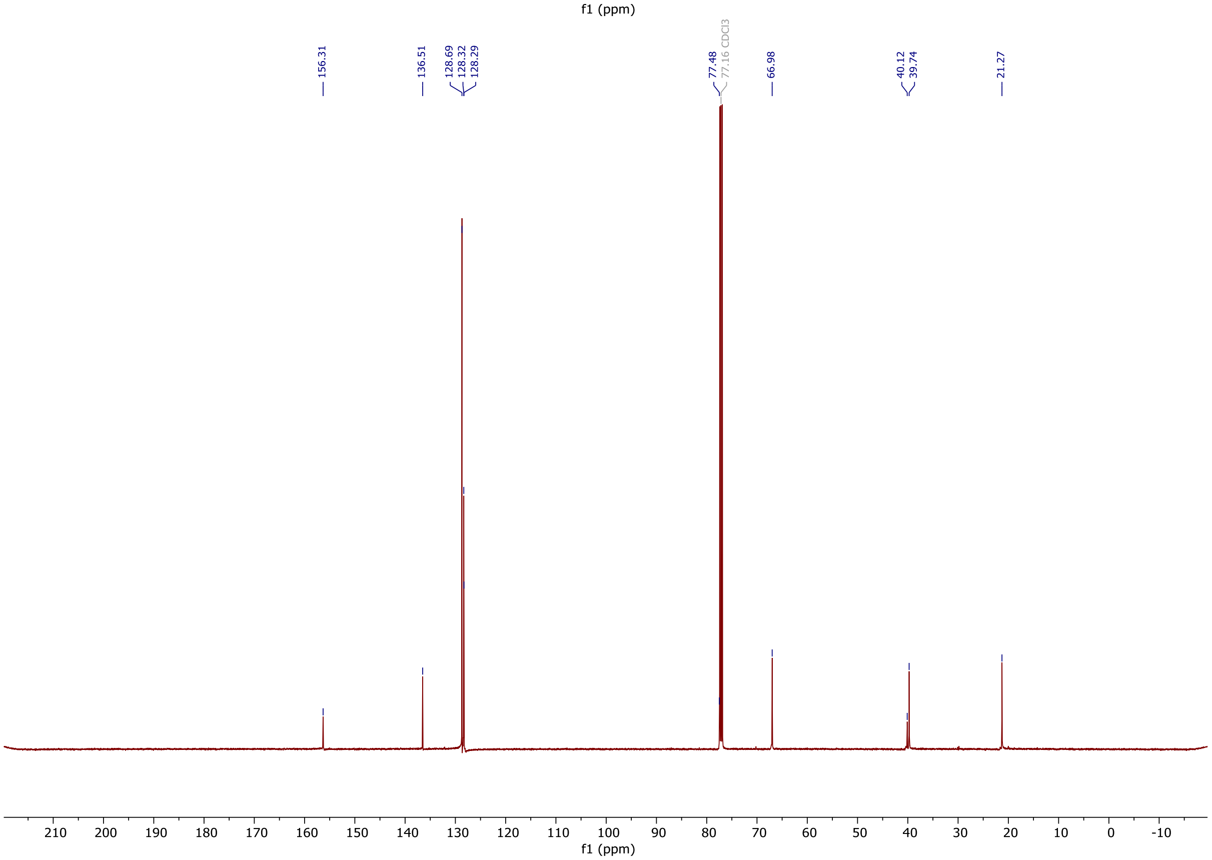

156.31
136.51
128.69
128.32
128.29
77.48
77.16 CDCl3
66.98
40.12
39.74
21.27
f1 (ppm)

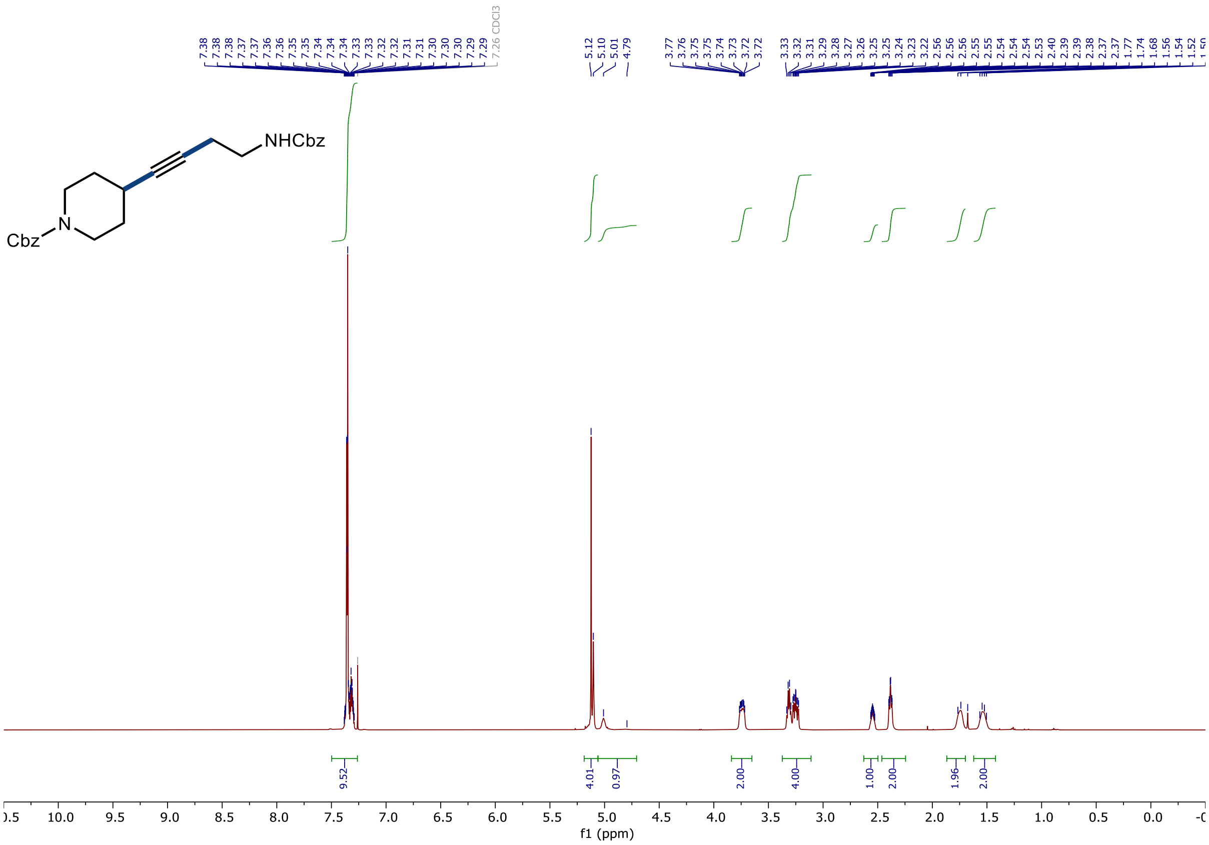
NHCbz
Cbz
N
7.26 CDCl3
f1 (ppm)

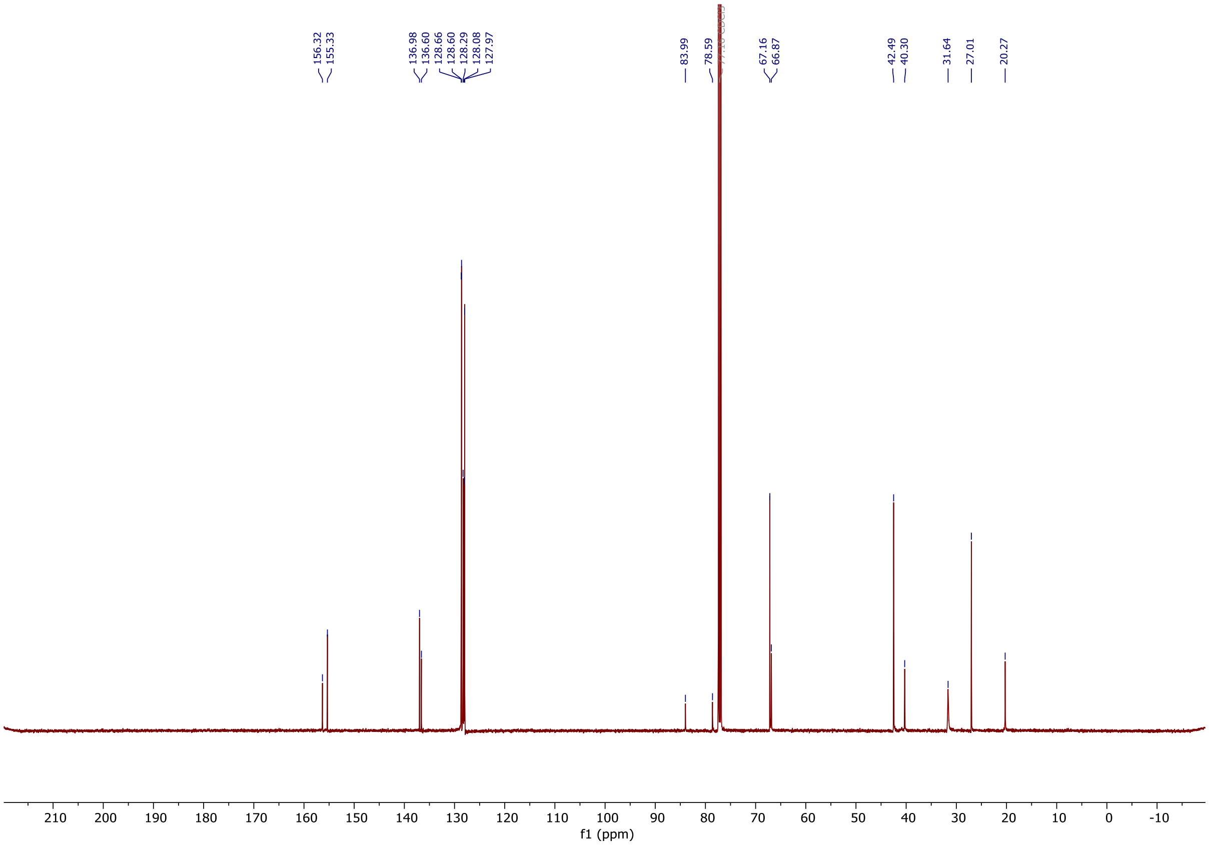
f1 (ppm)

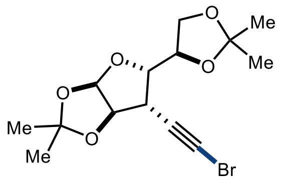
Me
Me
Me
Me
O
O
O
O
O
Br

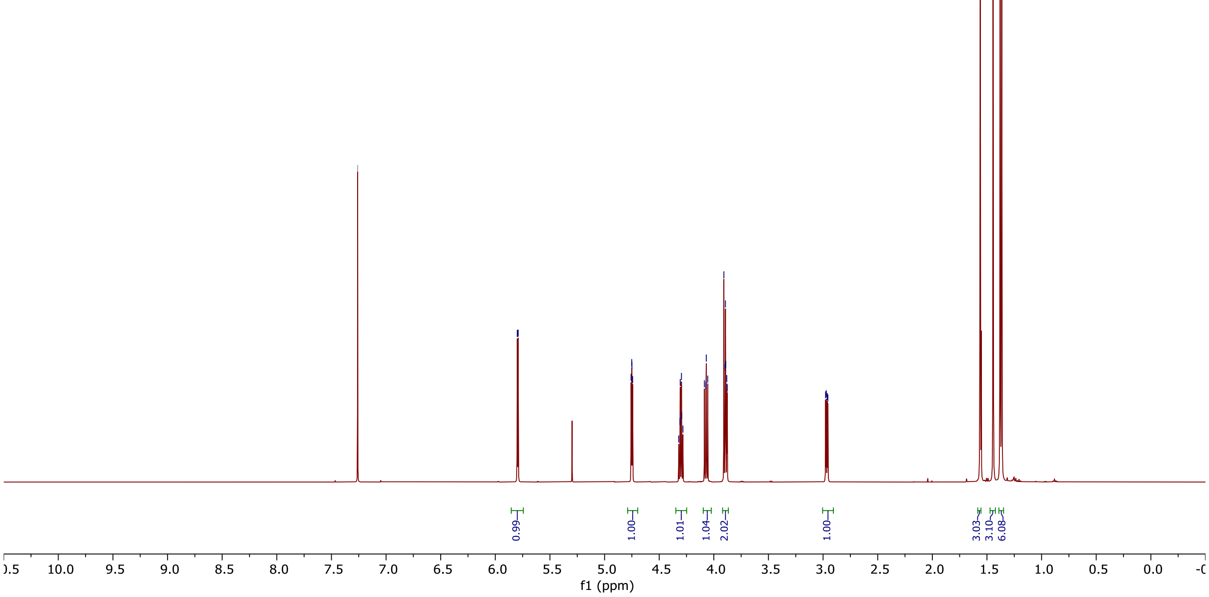
7.26 CDCl3
5.80
5.79
4.76
4.75
4.75
4.74
4.32
4.31
4.30
4.29
4.28
4.09
4.07
4.07
4.06
3.91
3.90
3.89
3.89
3.88
3.88
2.98
2.97
2.96
2.96
1.56
1.44
1.38
1.36
0.99
1.00
1.01
1.04
2.02
1.00
3.03
3.10
6.08
10.0
9.5
9.0
8.5
8.0
7.5
7.0
6.5
6.0
5.5
5.0
4.5
4.0
3.5
3.0
2.5
2.0
1.5
1.0
0.5
0.0
f1 (ppm)

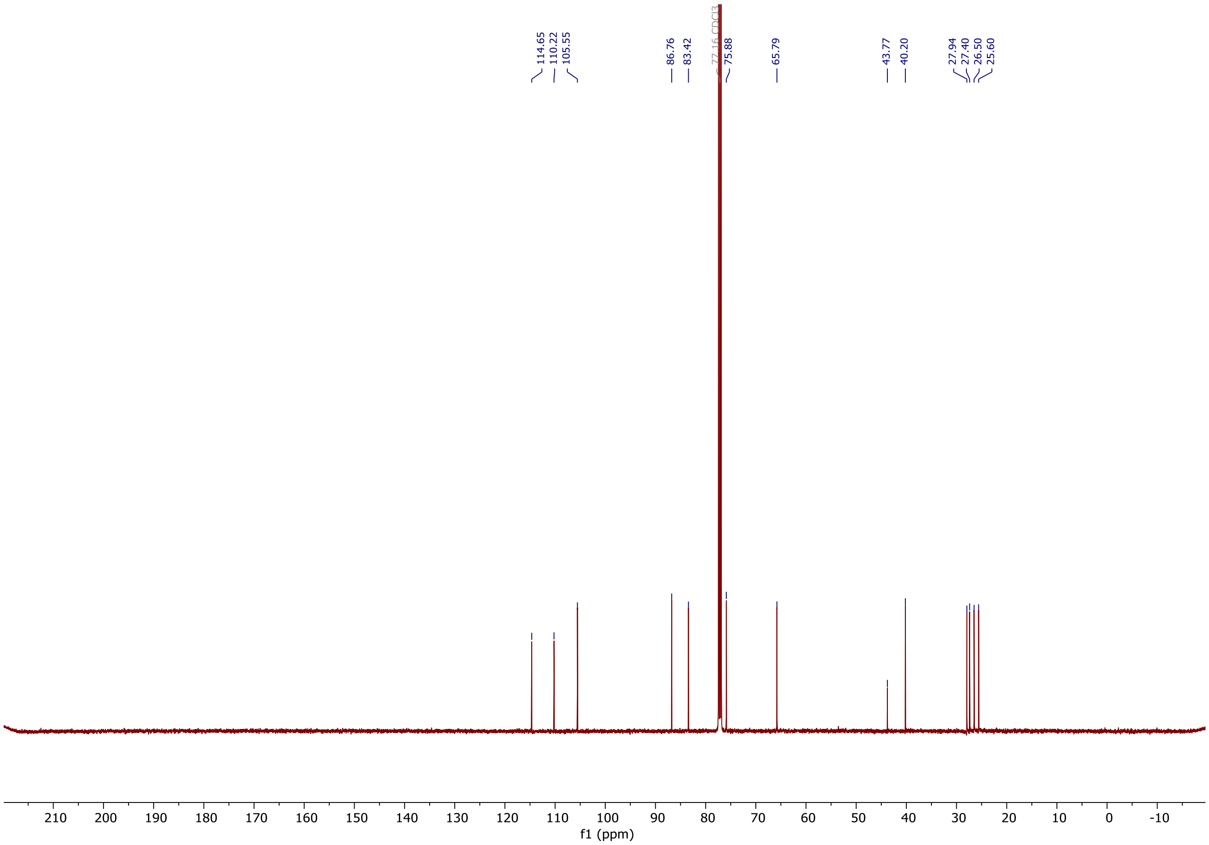
114.65
110.22
105.55
86.76
83.42
77.16 CDCl3
75.88
65.79
43.77
40.20
27.94
27.40
26.50
25.60
210
200
190
180
170
160
150
140
130
120
110
100
90
80
70
60
50
40
30
20
10
0
-10
f1 (ppm)

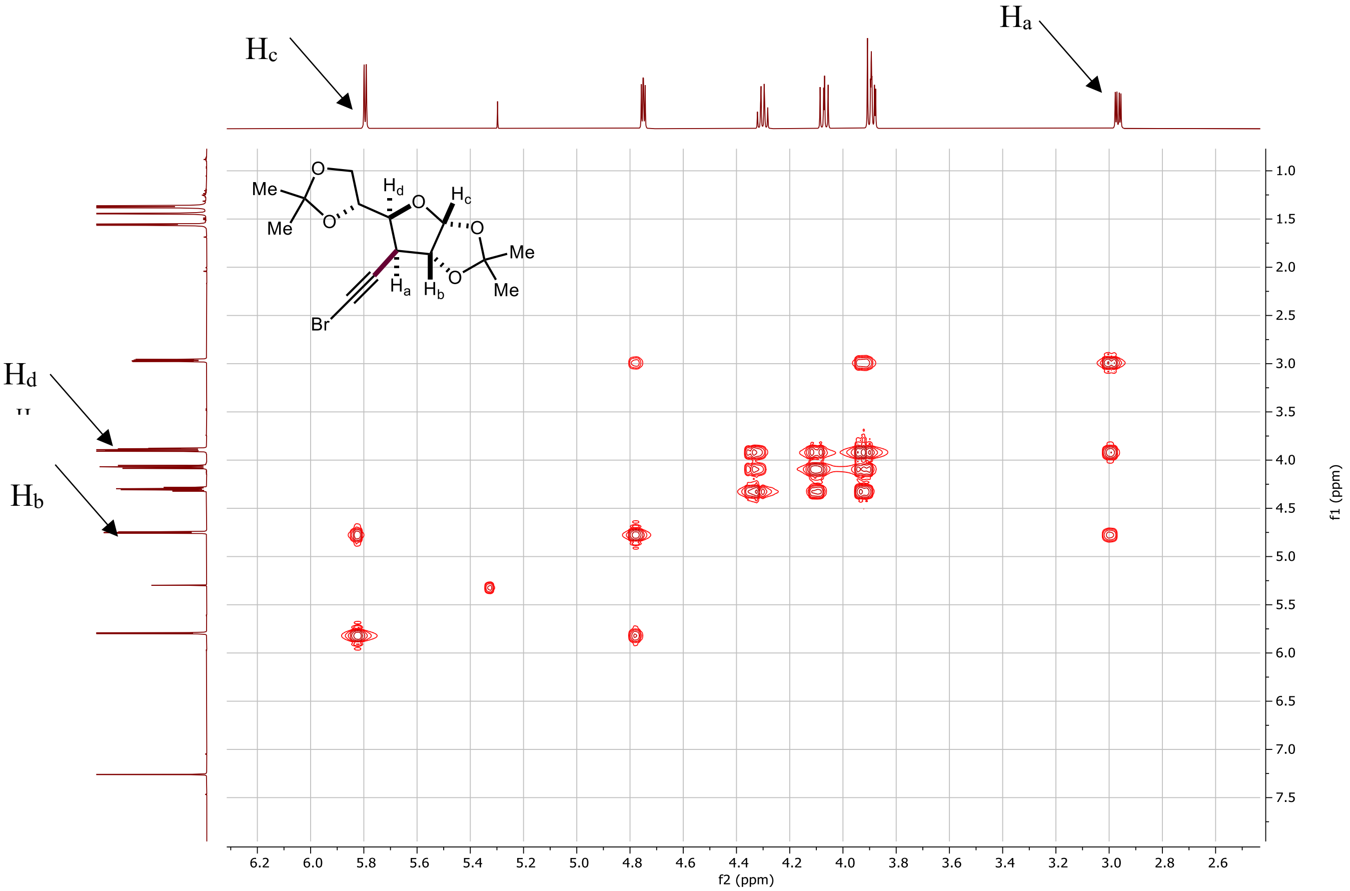

H-H COSY of **58**

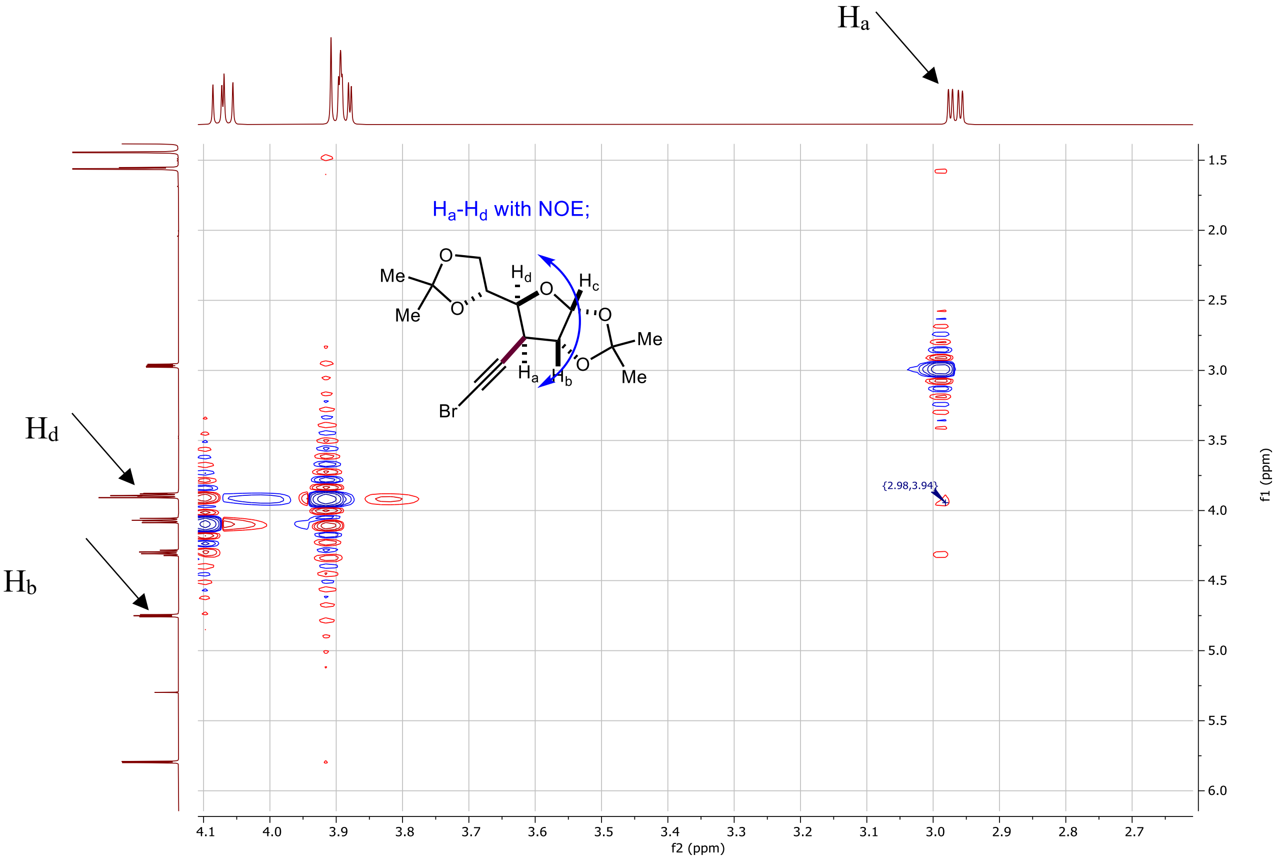

H-H NOESY of **58**

H_a and H_d, with NOE;

H_a and H_b, no NOE

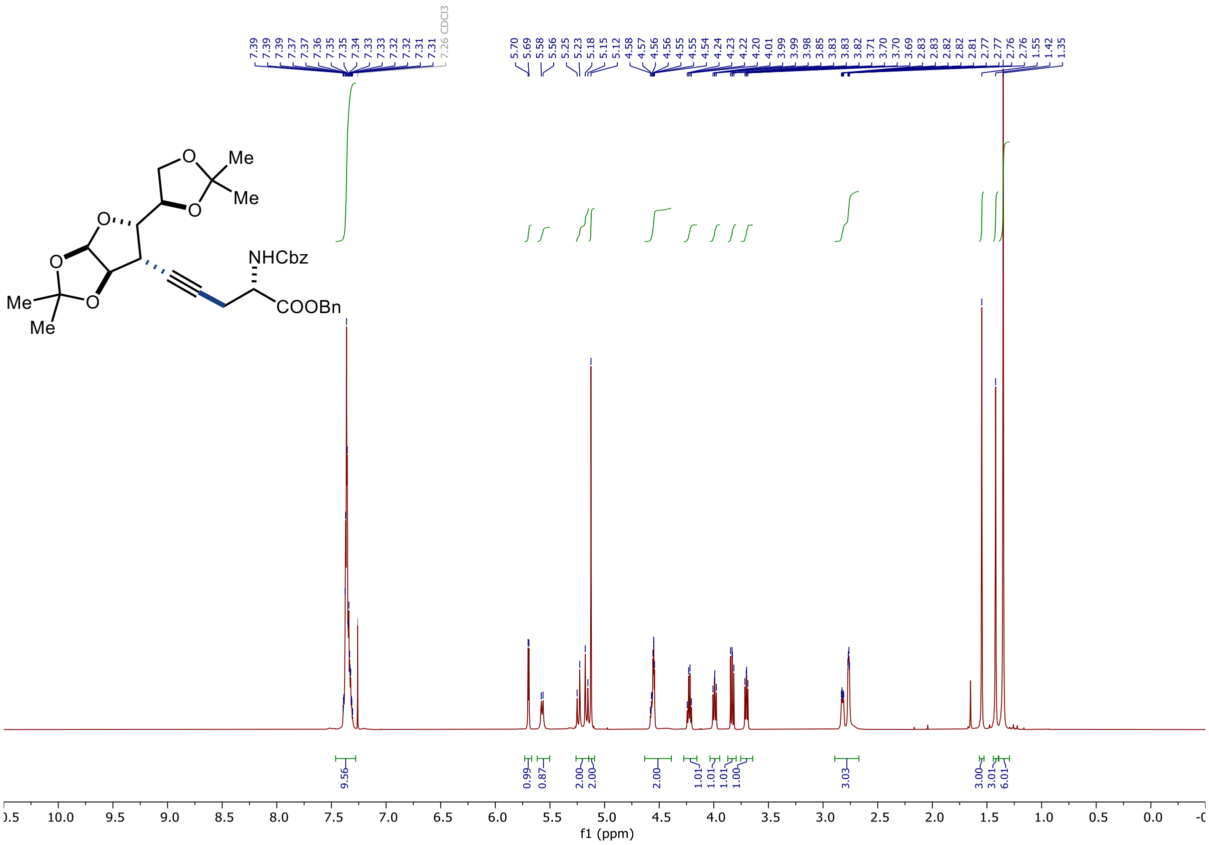
7.39 7.39 7.39 7.37 7.37 7.36 7.35 7.35 7.34 7.33 7.33 7.32 7.32 7.31 7.31 7.26 CDCl3
5.70 5.69 5.58 5.56 5.25 5.23 5.18 5.15 5.12 4.58 4.57 4.56 4.56 4.55 4.55 4.55 4.54 4.24 4.23 4.22 4.20 4.01 3.99 3.99 3.98 3.85 3.85 3.83 3.83 3.82 3.71 3.70 3.70 3.69 2.83 2.83 2.82 2.82 2.81 2.77 2.77 2.76 1.55 1.42 1.35
Me
Me
O
O
NHCbz
COOBn
9.56
0.99
0.87
2.00
2.00
2.00
1.01
1.01
1.01
1.00
3.03
3.00
3.01
6.01
f1 (ppm)

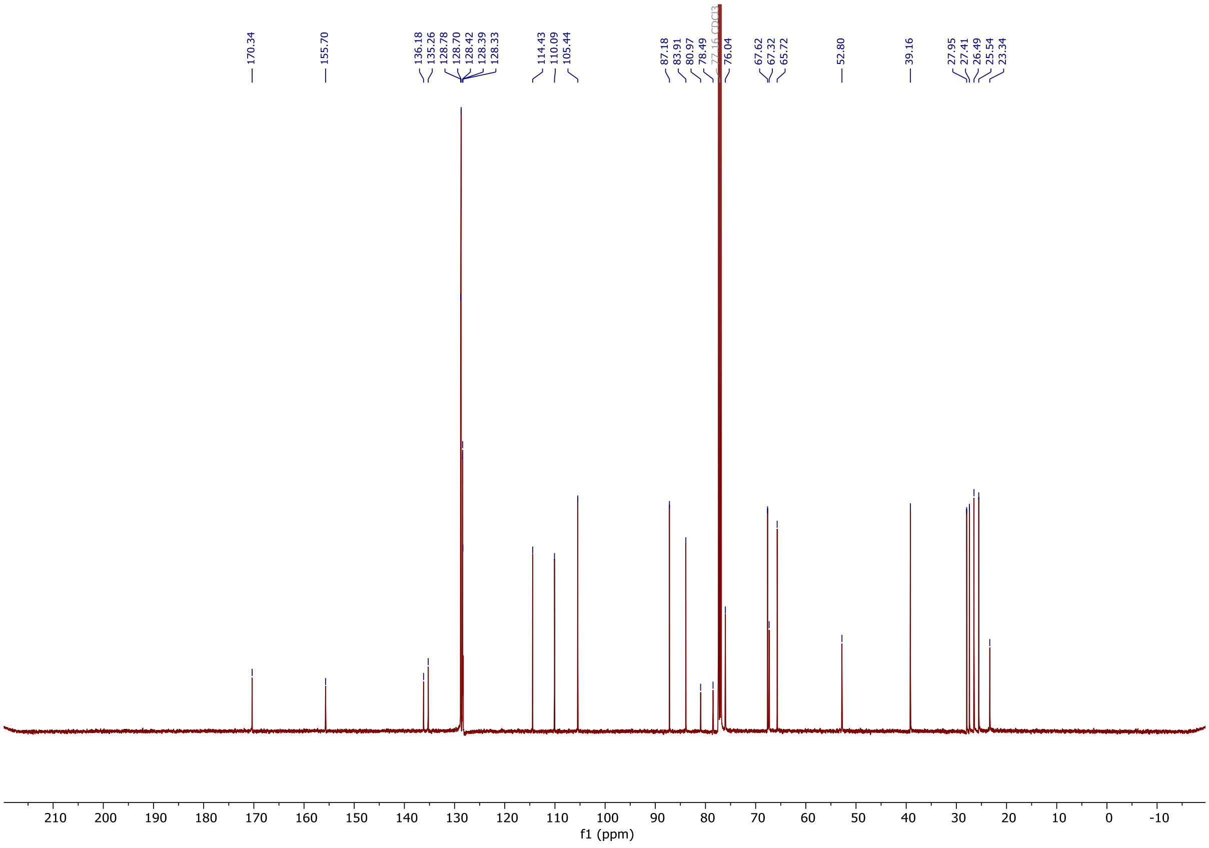
170.34
155.70
136.18 135.26 128.78 128.70 128.42 128.39 128.33
114.43 110.09 105.44
87.18 83.91 80.97 78.49 77.16 CDCl3 76.04
67.62 67.32 65.72
52.80
39.16
27.95 27.41 26.49 25.54 23.34
f1 (ppm)

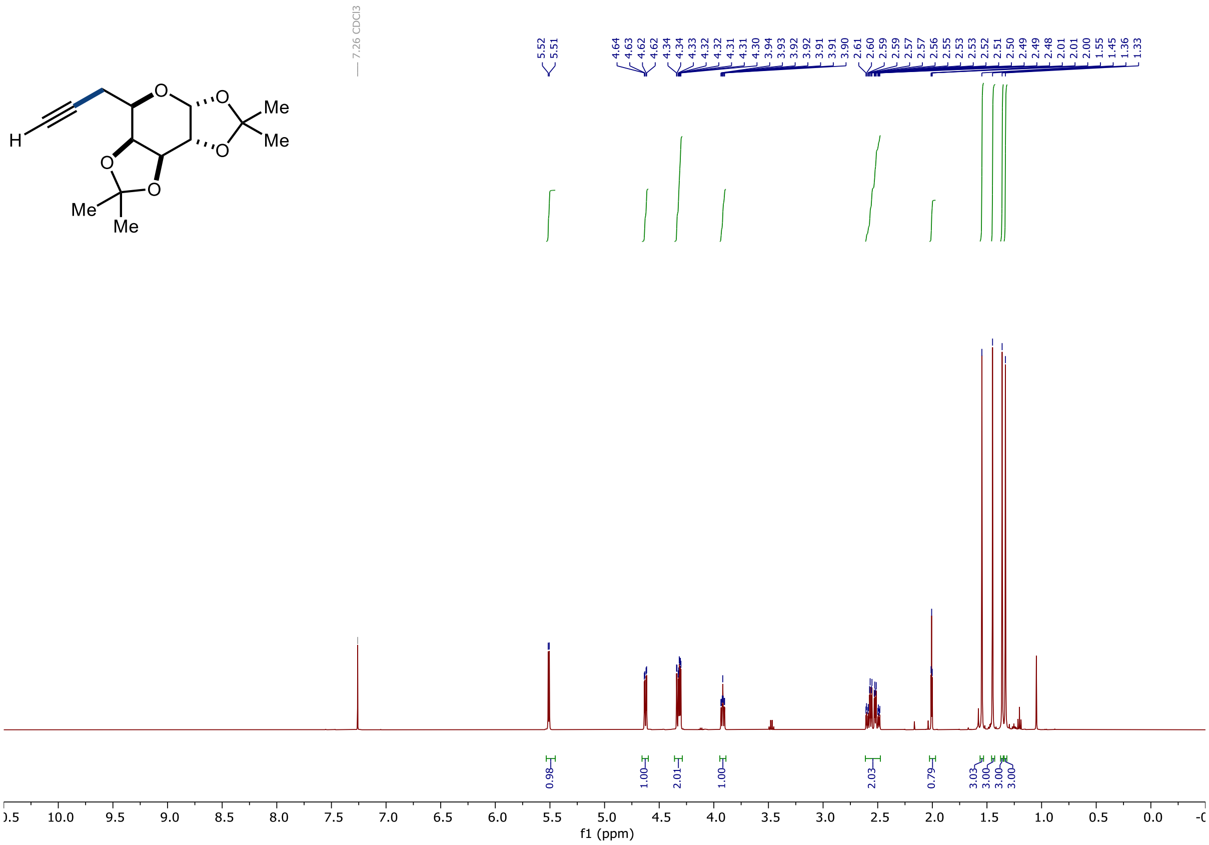

H
O
O
O
Me
Me
O
O
Me
Me
7.26 CDCl3
f1 (ppm)

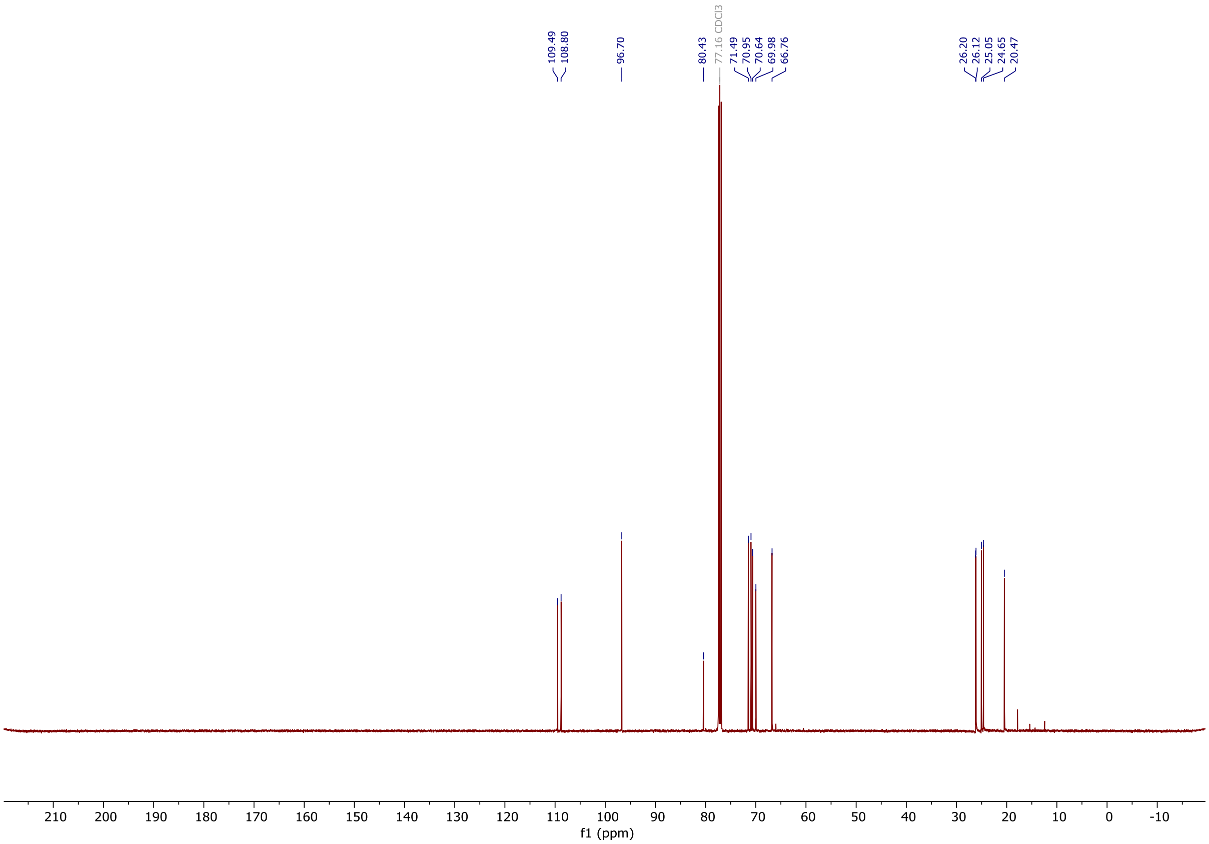

77.16 CDCl3
f1 (ppm)

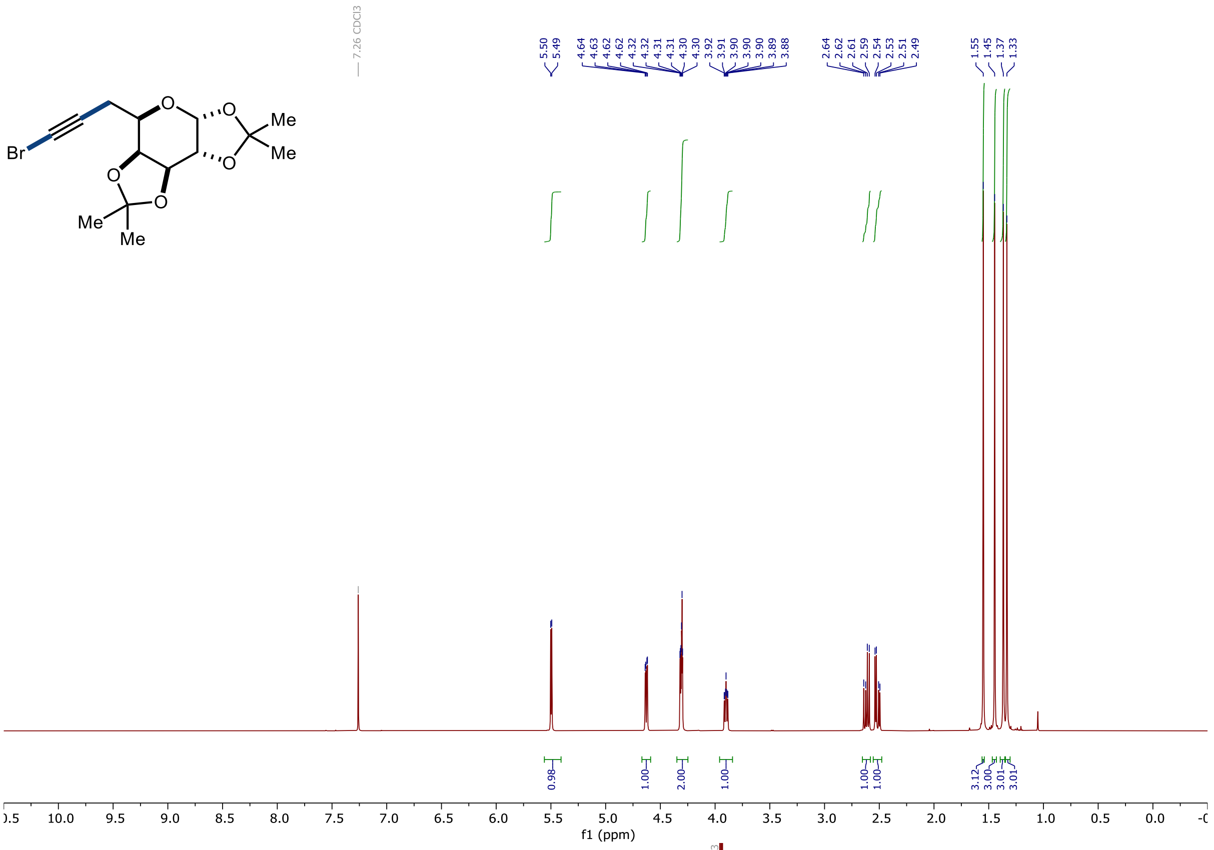
Br
O
O
O
Me
Me
O
O
Me
Me
7.26 CDCl3
5.50
5.49
4.64
4.63
4.62
4.62
4.32
4.32
4.31
4.31
4.30
4.30
3.92
3.91
3.90
3.90
3.90
3.89
3.88
2.64
2.62
2.61
2.59
2.54
2.53
2.51
2.49
1.55
1.45
1.37
1.33
0.98
1.00
2.00
1.00
1.00
1.00
3.12
3.00
3.01
3.01
f1 (ppm)

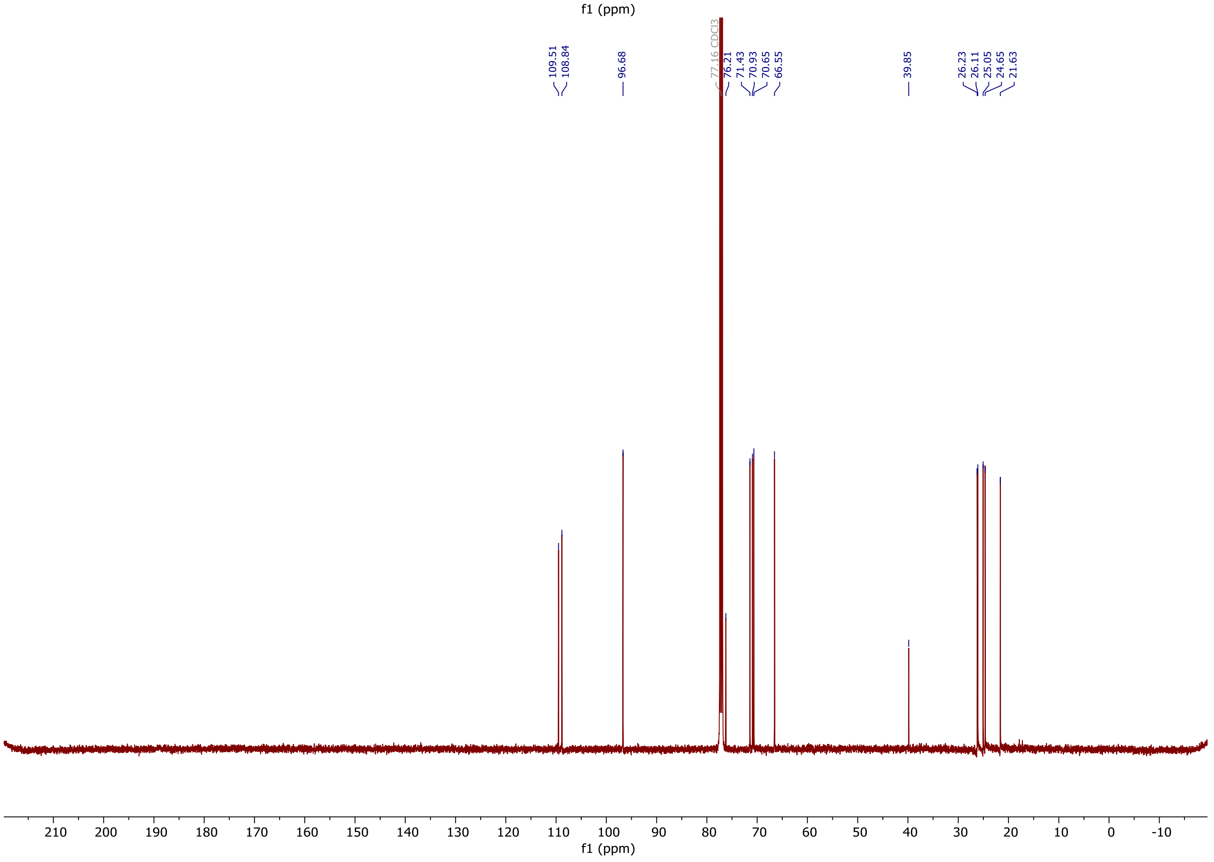
109.51
108.84
96.68
77.16 CDCl3
76.21
71.43
70.93
70.65
66.55
39.85
26.23
26.11
25.05
24.65
21.63
f1 (ppm)

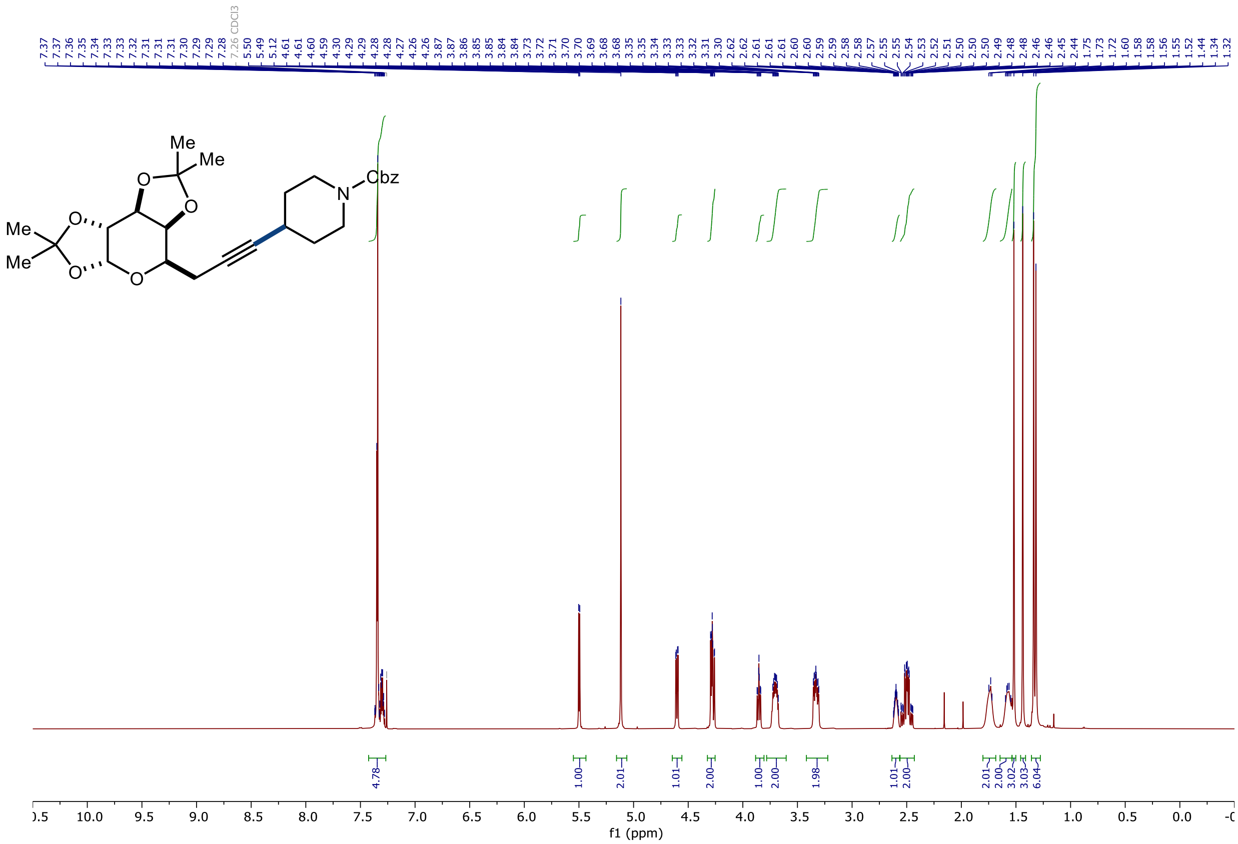
Me
Me
Me
Me
O
O
O
O
O
N
Cbz
4.78
1.00
2.01
1.01
2.00
1.00
2.00
1.98
1.01
2.00
2.01
2.00
3.02
3.03
6.04
f1 (ppm)

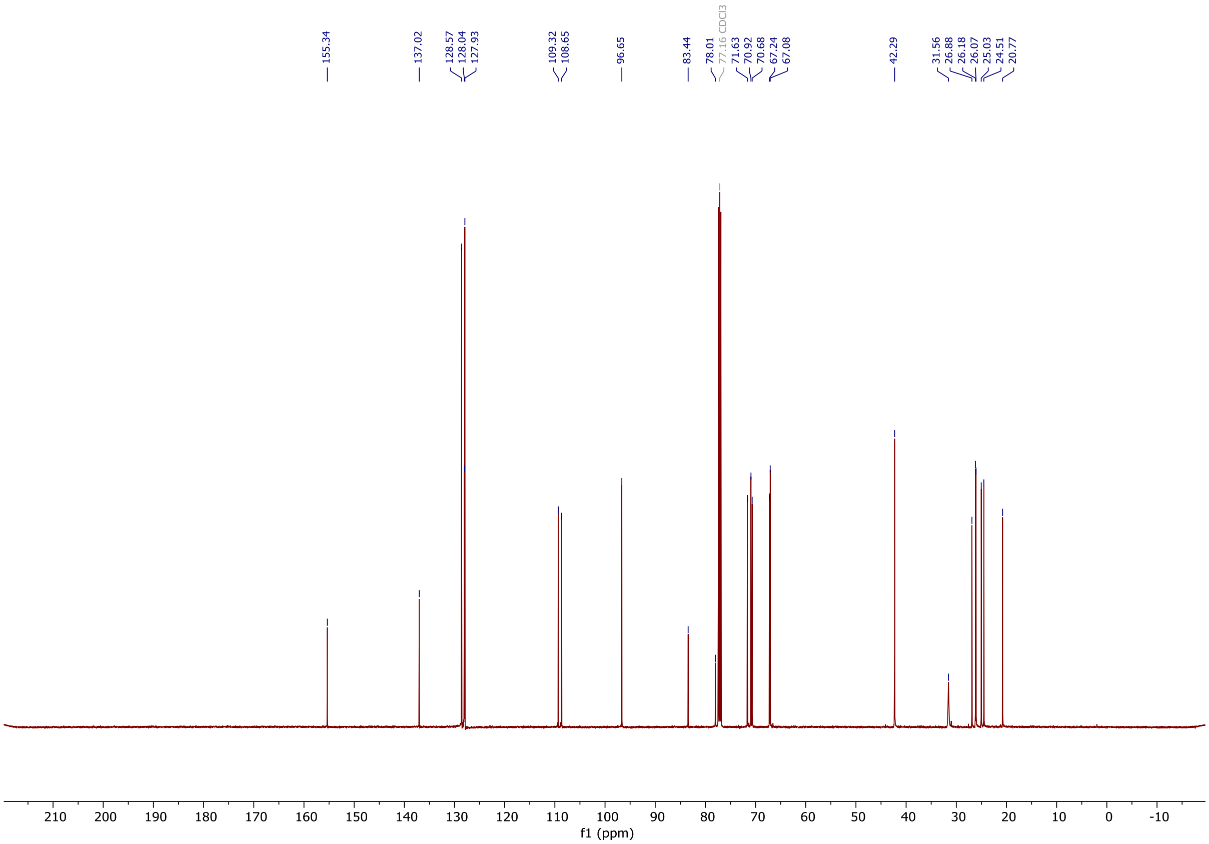
155.34
137.02
128.57
128.04
127.93
109.32
108.65
96.65
83.44
78.01
77.16 CDCl3
71.63
70.92
70.68
67.24
67.08
42.29
31.56
26.88
26.18
26.07
25.03
24.51
20.77
f1 (ppm)

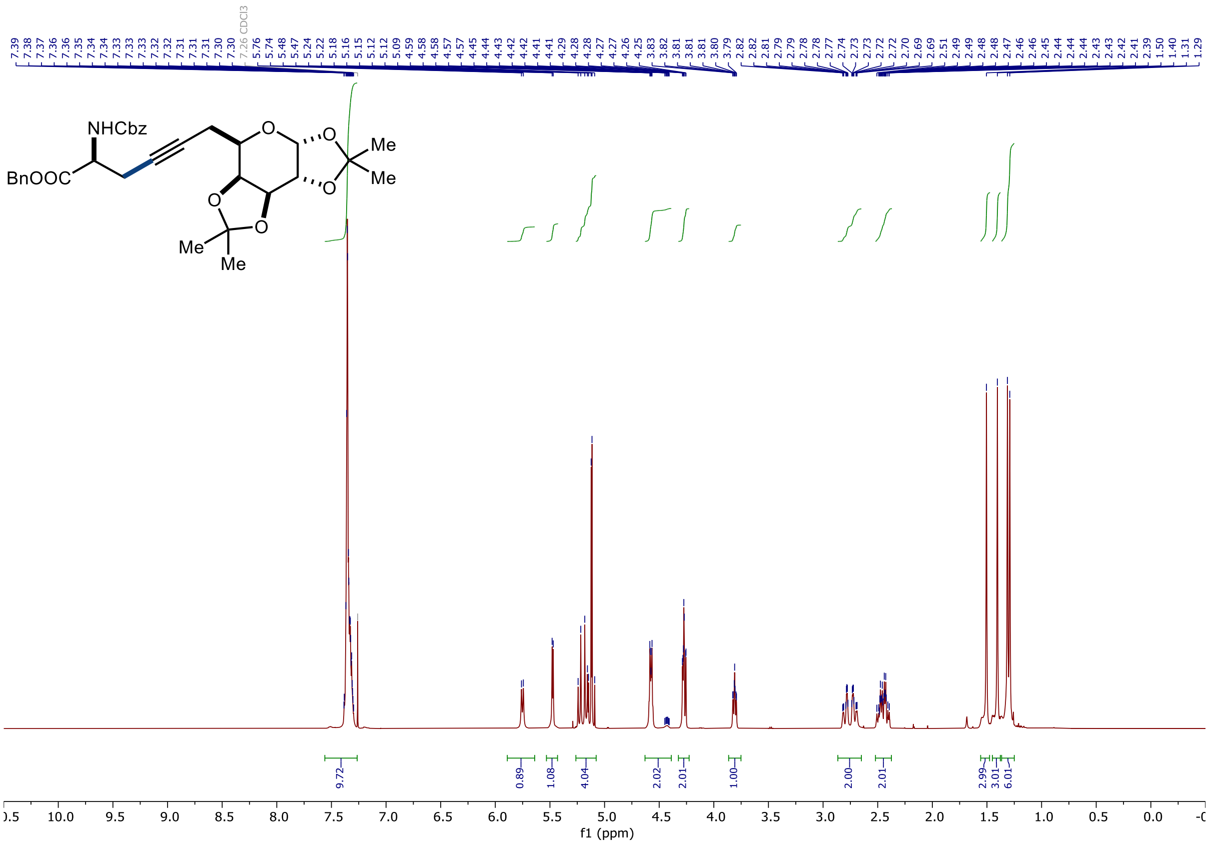

7.39 7.38 7.37 7.36 7.35 7.34 7.33 7.33 7.32 7.32 7.31 7.31 7.31 7.30 7.26 CDCl3 5.76 5.74 5.48 5.47 5.24 5.22 5.18 5.16 5.15 5.12 5.12 5.09 4.59 4.58 4.58 4.57 4.57 4.45 4.44 4.43 4.42 4.42 4.41 4.29 4.28 4.28 4.27 4.27 4.26 4.25 3.83 3.82 3.81 3.81 3.80 3.79 2.82 2.82 2.81 2.79 2.79 2.78 2.78 2.77 2.74 2.73 2.73 2.72 2.72 2.70 2.69 2.69 2.51 2.49 2.49 2.48 2.48 2.47 2.46 2.46 2.45 2.44 2.44 2.44 2.43 2.43 2.42 2.41 2.39 1.50 1.40 1.31 1.29
NHCbz
BnOOC
O
Me
Me
Me
Me
9.72
0.89
1.08
4.04
2.02
2.01
1.00
2.00
2.01
2.99
3.01
6.01
f1 (ppm)

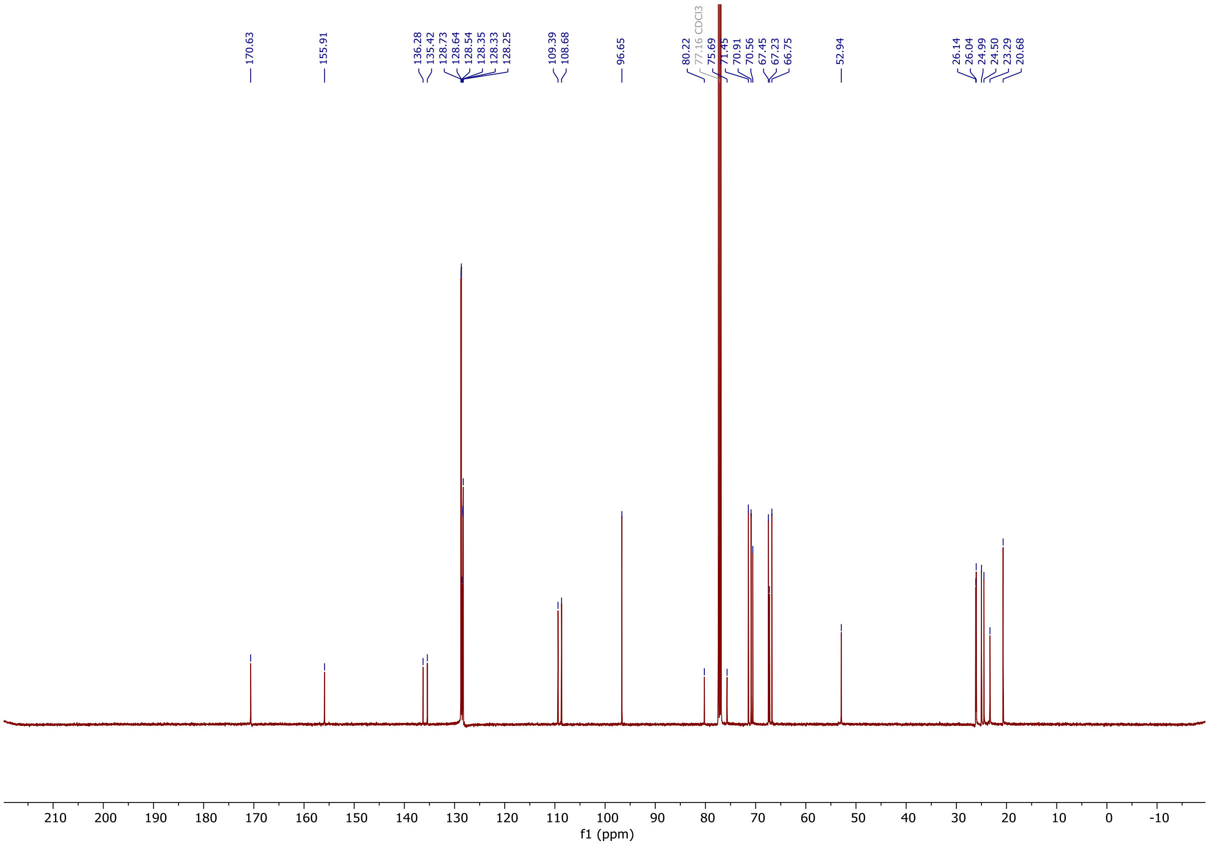

170.63
155.91
136.28 135.42 128.73 128.64 128.54 128.35 128.33 128.25
109.39 108.68
96.65
80.22 77.16 CDCl3 75.69 71.45 70.91 70.56 67.45 67.23 66.75
52.94
26.14 26.04 24.99 24.50 23.29 20.68
f1 (ppm)

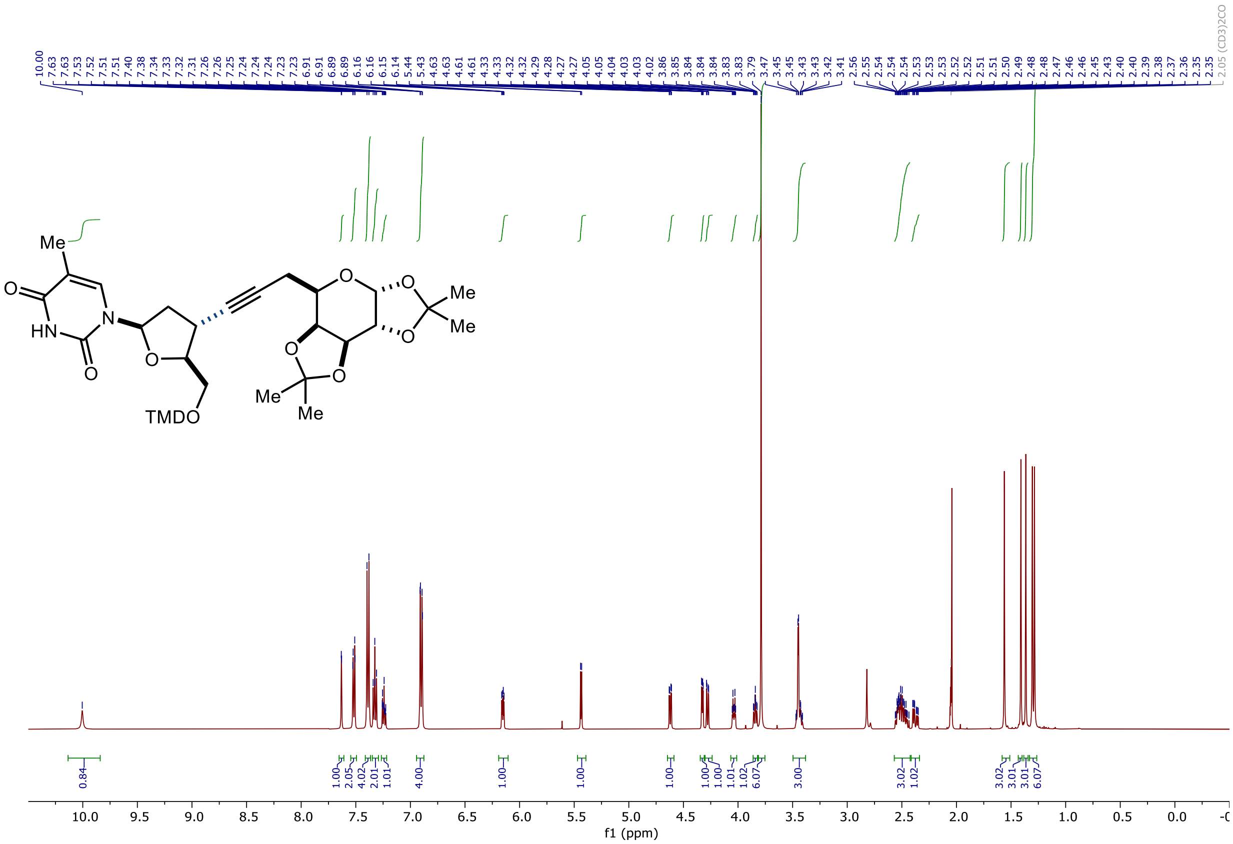

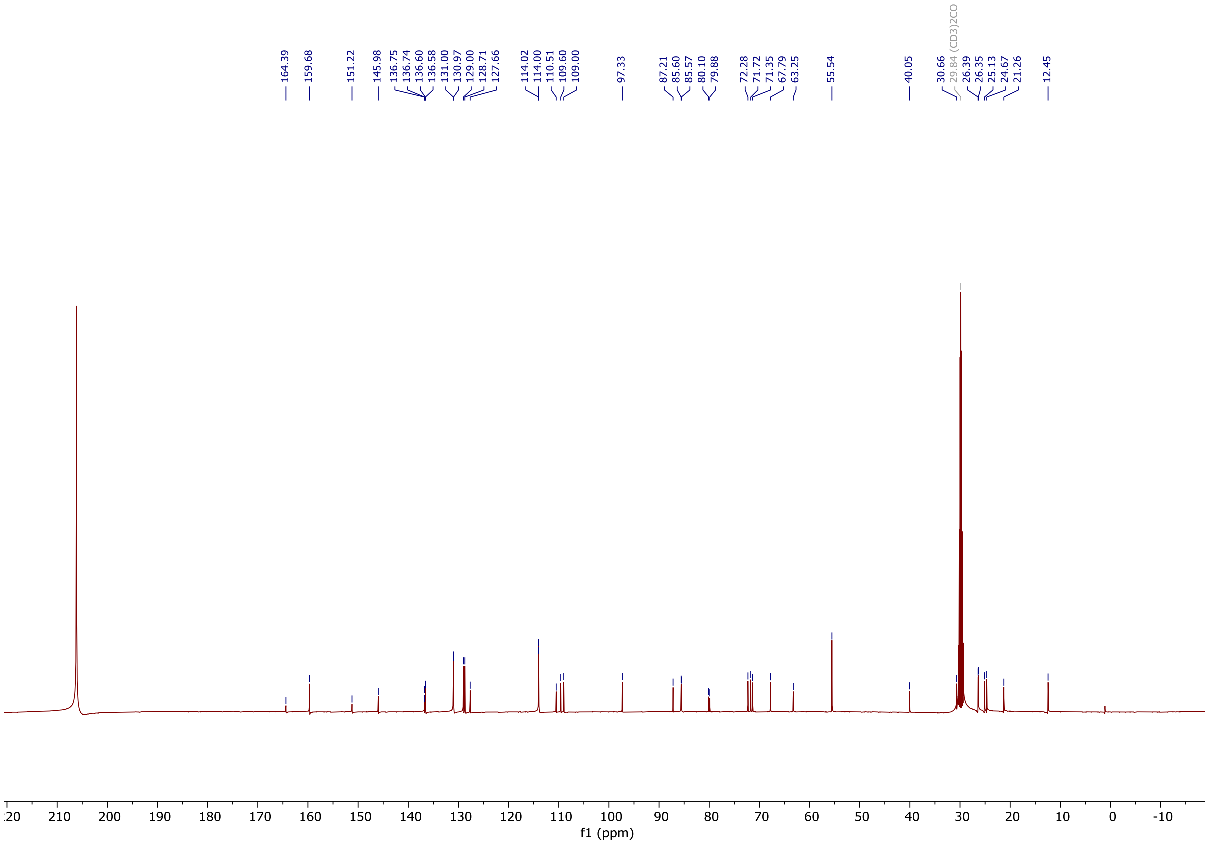

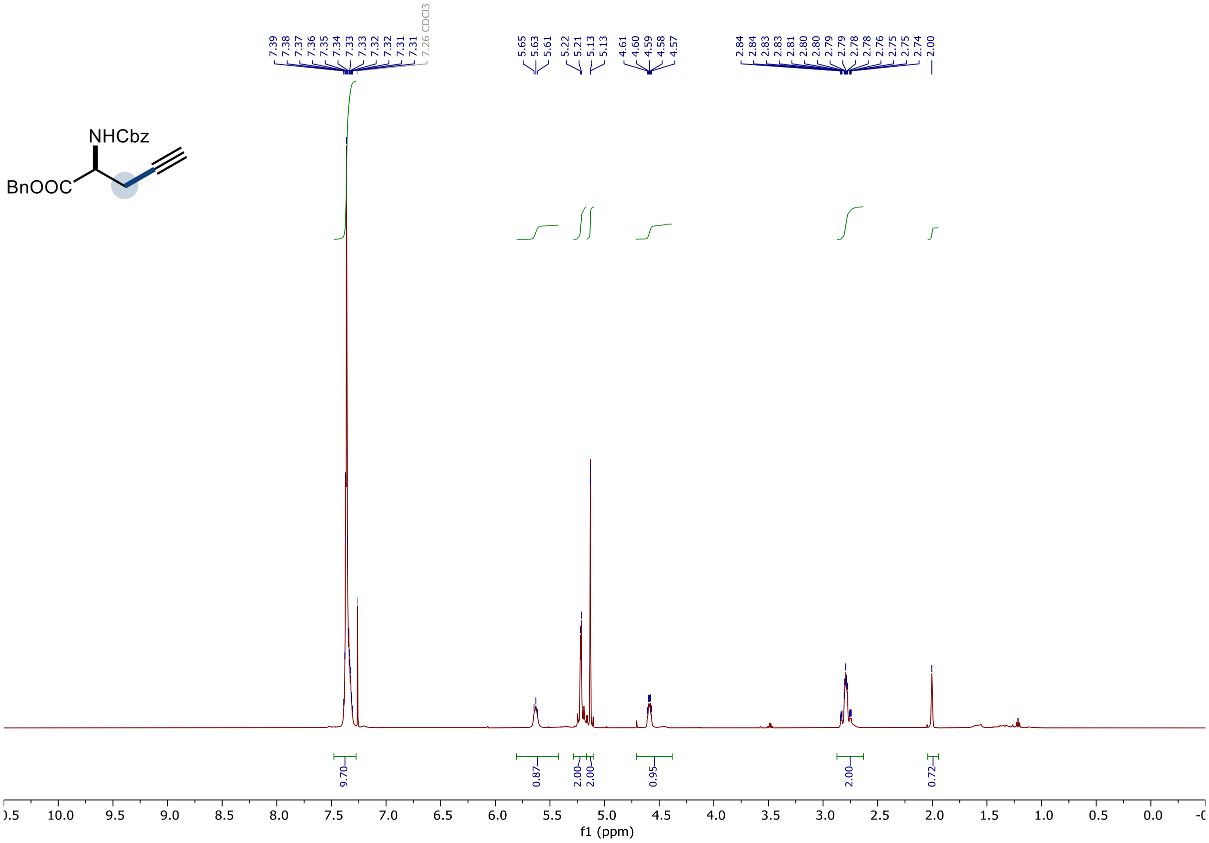
NHCbz
BnOOC
7.39
7.38
7.37
7.36
7.35
7.34
7.33
7.33
7.32
7.32
7.31
7.31
7.26 CDCl3
5.65
5.63
5.61
5.22
5.21
5.13
5.13
4.61
4.60
4.59
4.58
4.57
2.84
2.84
2.83
2.83
2.81
2.80
2.80
2.79
2.79
2.78
2.78
2.76
2.75
2.75
2.74
2.00
9.70
0.87
2.00
2.00
0.95
2.00
0.72
f1 (ppm)

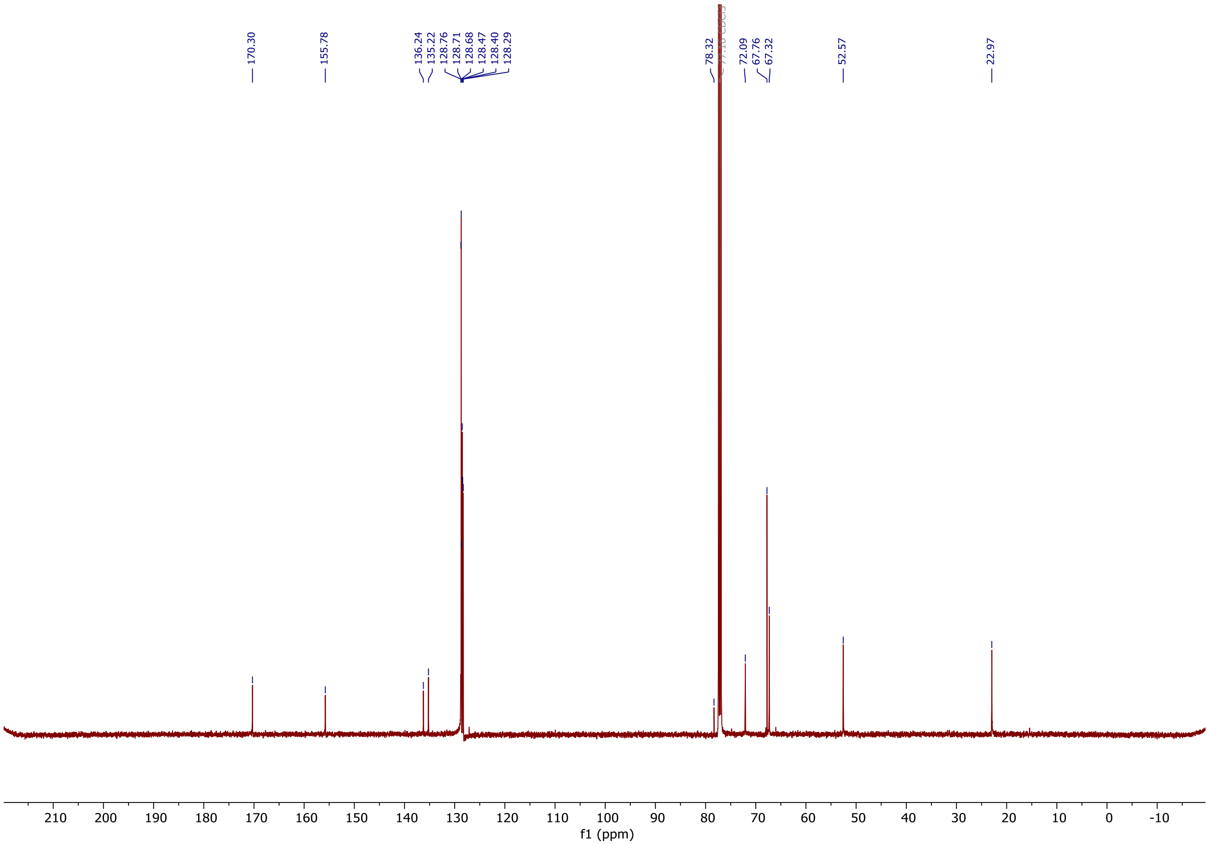
170.30
155.78
136.24
135.22
128.76
128.71
128.68
128.47
128.40
128.29
78.32
72.09
67.76
67.32
52.57
22.97
f1 (ppm)

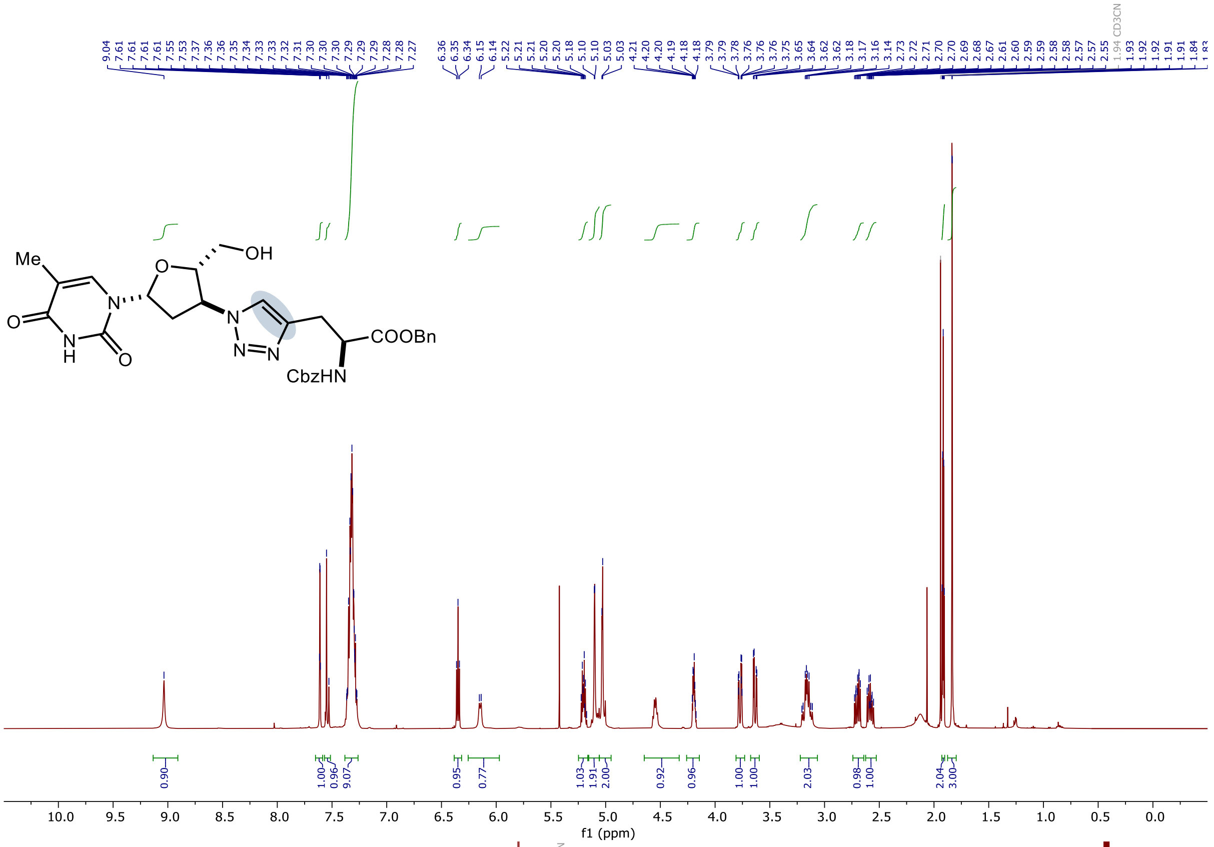
9.04 7.61 7.61 7.61 7.55 7.53 7.37 7.36 7.36 7.35 7.34 7.33 7.33 7.32 7.31 7.30 7.30 7.30 7.29 7.29 7.29 7.28 7.28 7.27
6.36 6.35 6.34 6.15 6.14 5.22 5.21 5.21 5.20 5.20 5.18 5.10 5.10 5.03 4.21 4.20 4.20 4.19 4.18 4.18 3.79 3.79 3.78 3.76 3.76 3.76 3.75 3.65 3.64 3.62 3.62 3.18 3.17 3.16 3.14 2.73 2.72 2.71 2.70 2.70 2.69 2.68 2.67 2.61 2.60 2.59 2.59 2.58 2.58 2.57 2.57 2.55
1.94 CD3CN
1.93 1.92 1.92 1.91 1.91 1.84
Me
O
N
OH
O
N
H
O
N
N=N
COOBn
CbzHN
0.90 1.00 0.96 9.07 0.95 0.77 1.03 1.91 2.00 0.92 0.96 1.00 1.00 2.03 0.98 1.00 2.04 3.00
10.0 9.5 9.0 8.5 8.0 7.5 7.0 6.5 6.0 5.5 5.0 4.5 4.0 3.5 3.0 2.5 2.0 1.5 1.0 0.5 0.0
f1 (ppm)

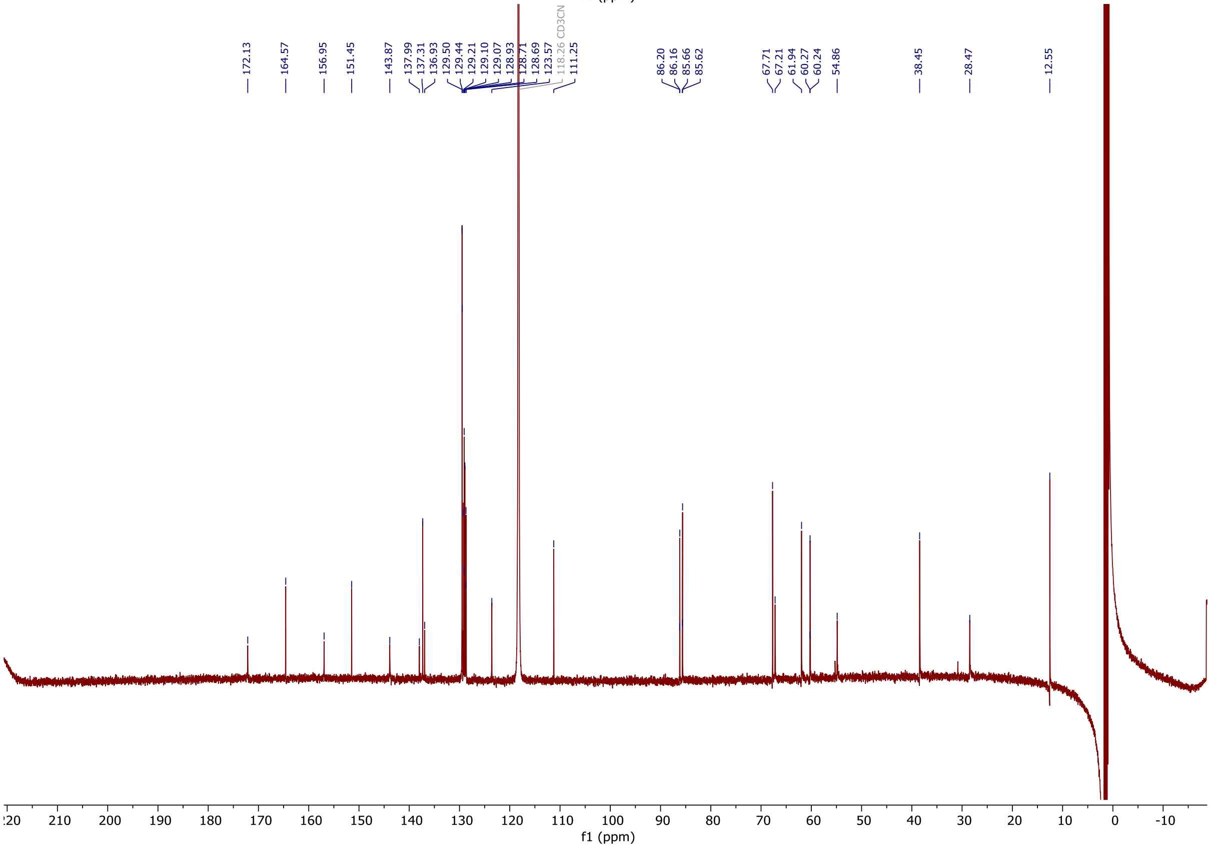
172.13 164.57 156.95 151.45 143.87 137.99 137.31 136.93 129.50 129.44 129.21 129.10 129.07 128.93 128.71 128.69 123.57
118.26 CD3CN
111.25 86.20 86.16 85.66 85.62 67.71 67.21 61.94 60.27 60.24 54.86 38.45 28.47 12.55
220 210 200 190 180 170 160 150 140 130 120 110 100 90 80 70 60 50 40 30 20 10 0 -10
f1 (ppm)

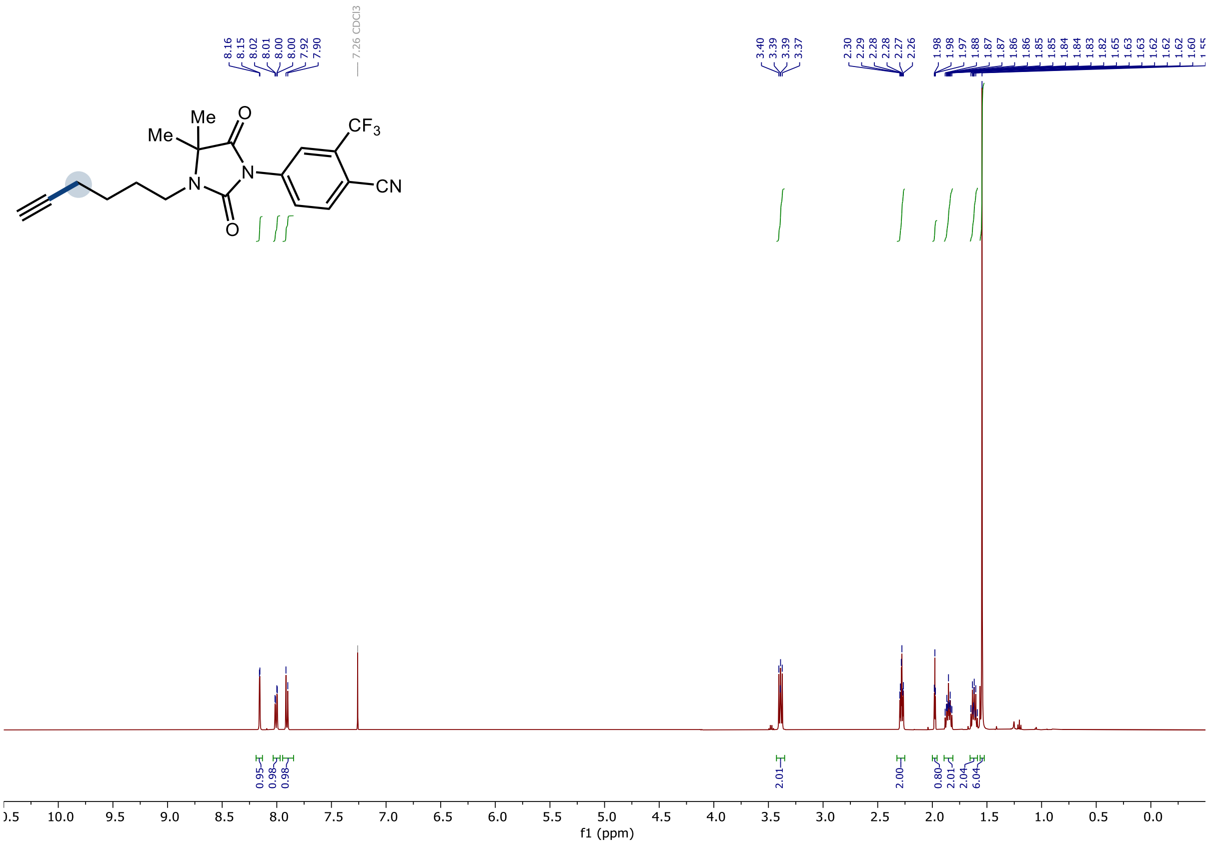
8.16 8.15 8.02 8.01 8.00 8.00 7.92 7.90
7.26 CDCl3
3.40 3.39 3.39 3.37
2.30 2.29 2.28 2.28 2.27 2.26
1.98 1.98 1.97 1.88 1.87 1.86 1.86 1.85 1.85 1.84 1.84 1.83 1.82 1.65 1.63 1.63 1.62 1.62 1.62 1.60
Me
Me
O
CF3
N
N
CN
O
0.95 0.98 0.98
2.01
2.00
0.80 2.01 2.04 6.04
f1 (ppm)

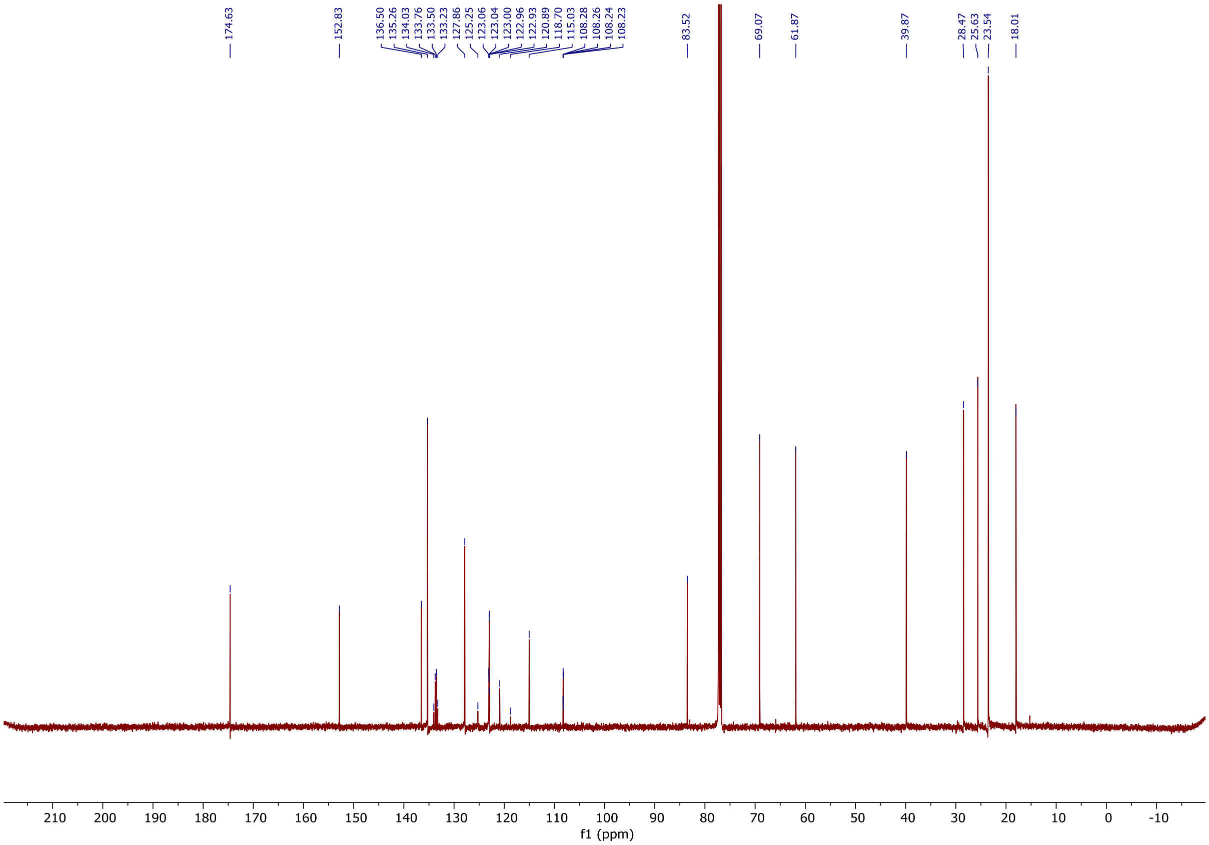
174.63
152.83
136.50 135.26 134.03 133.76 133.50 133.23 127.86 125.25 123.06 123.04 123.00 122.96 122.93 120.89 118.70 115.03 108.28 108.26 108.24 108.23
83.52
69.07
61.87
39.87
28.47 25.63 23.54
18.01
f1 (ppm)

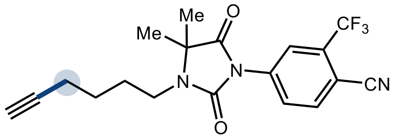

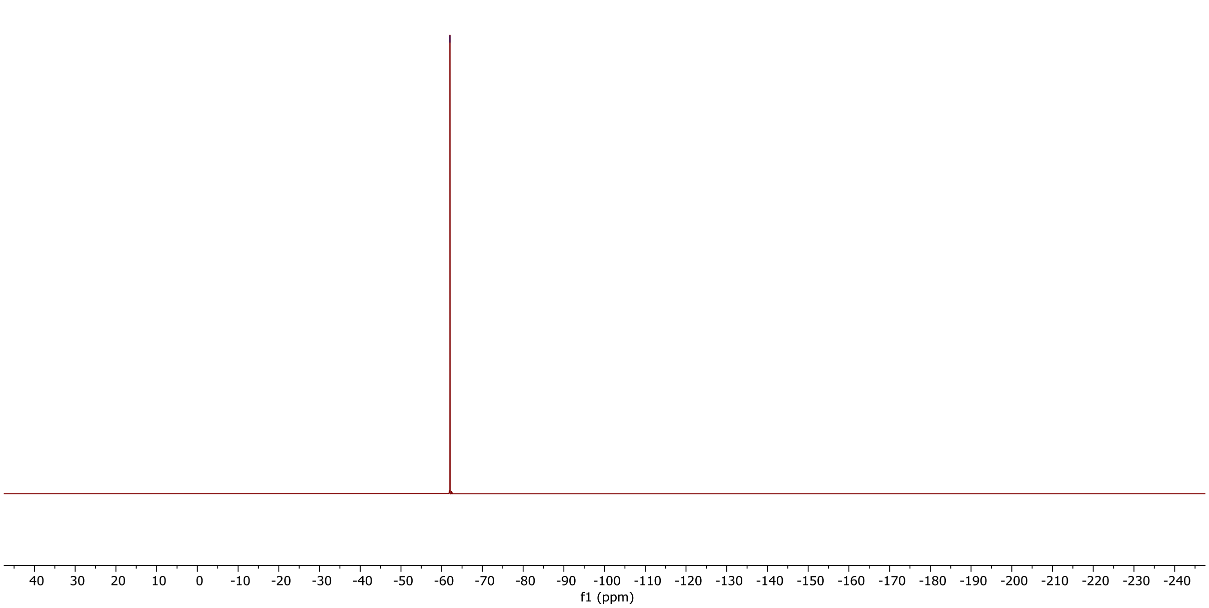

-62.03
40 30 20 10 0 -10 -20 -30 -40 -50 -60 -70 -80 -90 -100 -110 -120 -130 -140 -150 -160 -170 -180 -190 -200 -210 -220 -230 -240
f1 (ppm)

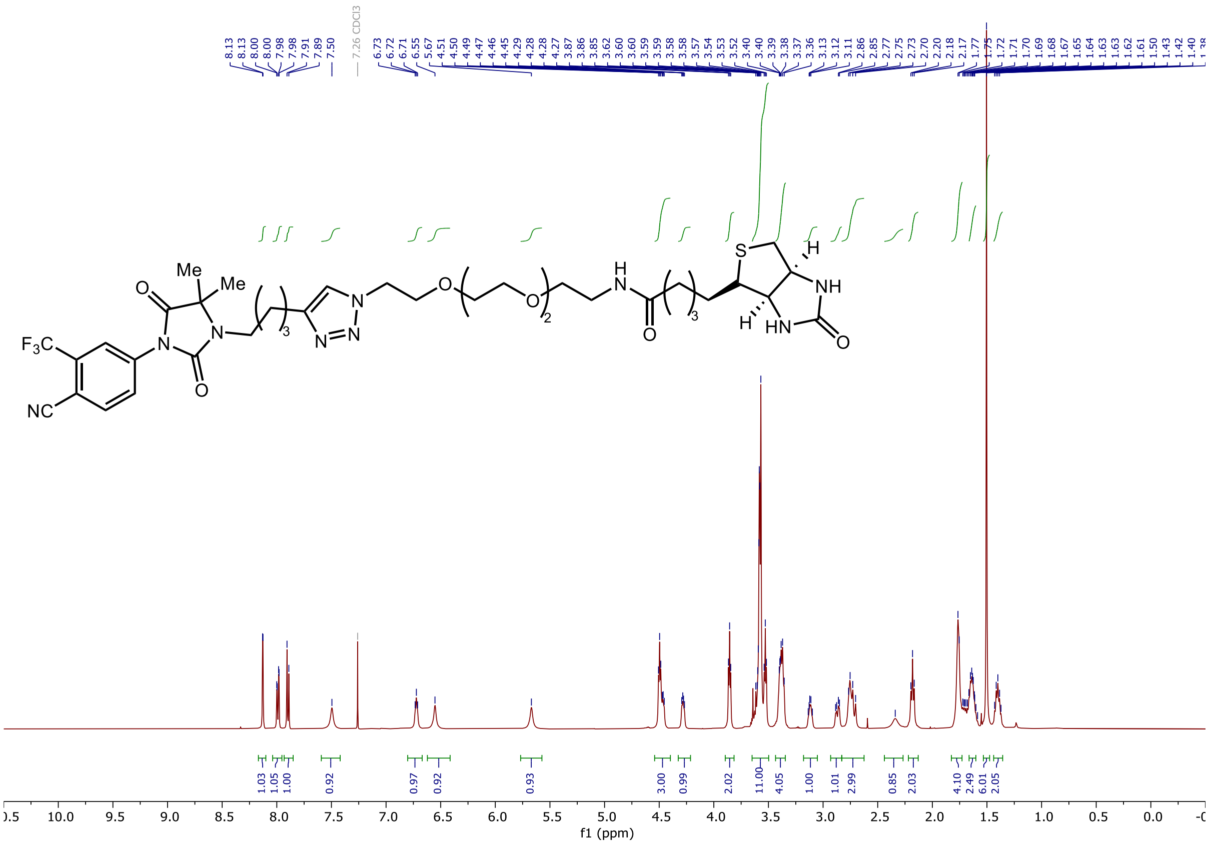
F3C
NC
Me
Me
O
N
N
O
3
N
N=N
O
O
2
H
N
O
3
S
H
H
NH
HN
O
7.26 CDCl3
f1 (ppm)

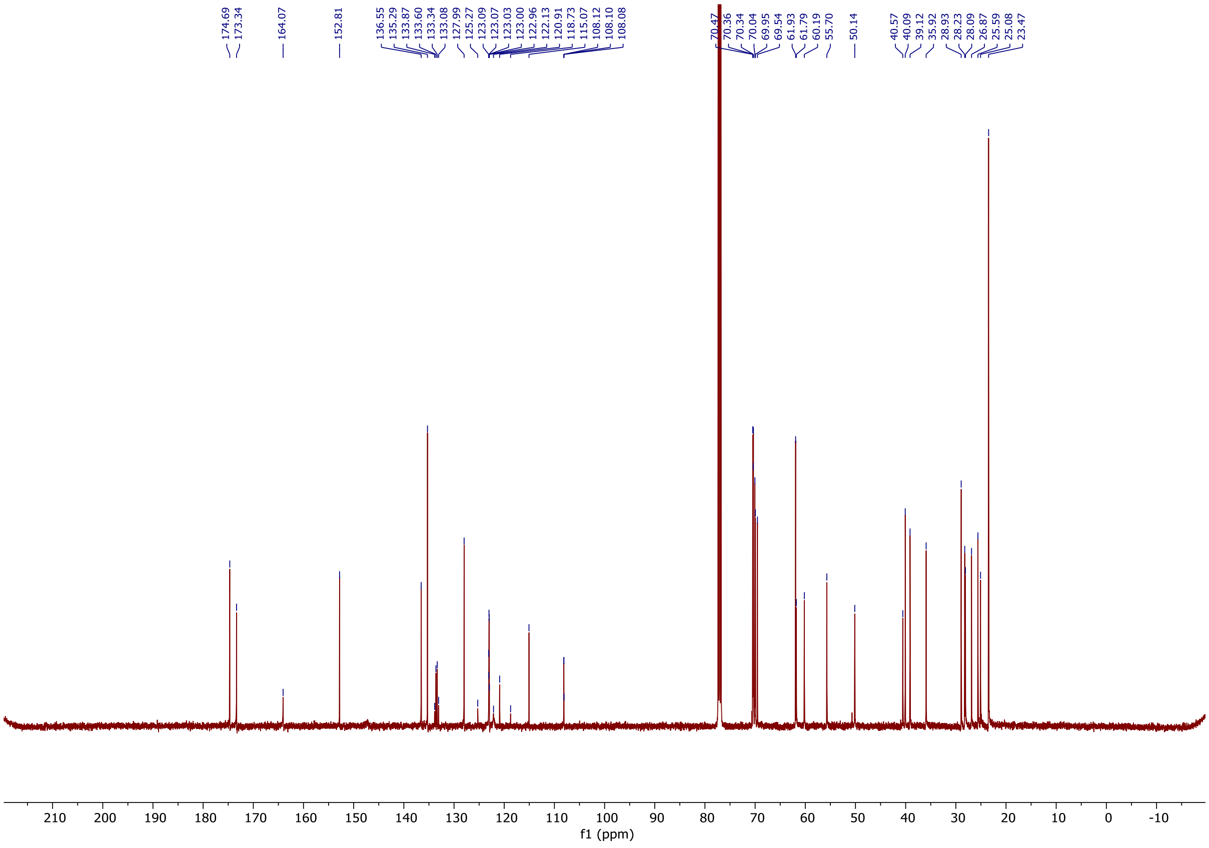
174.69
173.34
164.07
152.81
136.55
135.29
133.87
133.60
133.34
133.08
127.99
125.27
123.09
123.07
123.03
123.00
122.96
122.13
120.91
118.73
115.07
108.12
108.10
108.08
70.47
70.36
70.34
70.04
69.95
69.54
61.93
61.79
60.19
55.70
50.14
40.57
40.09
39.12
35.92
28.93
28.23
28.09
26.87
25.59
25.08
23.47
f1 (ppm)

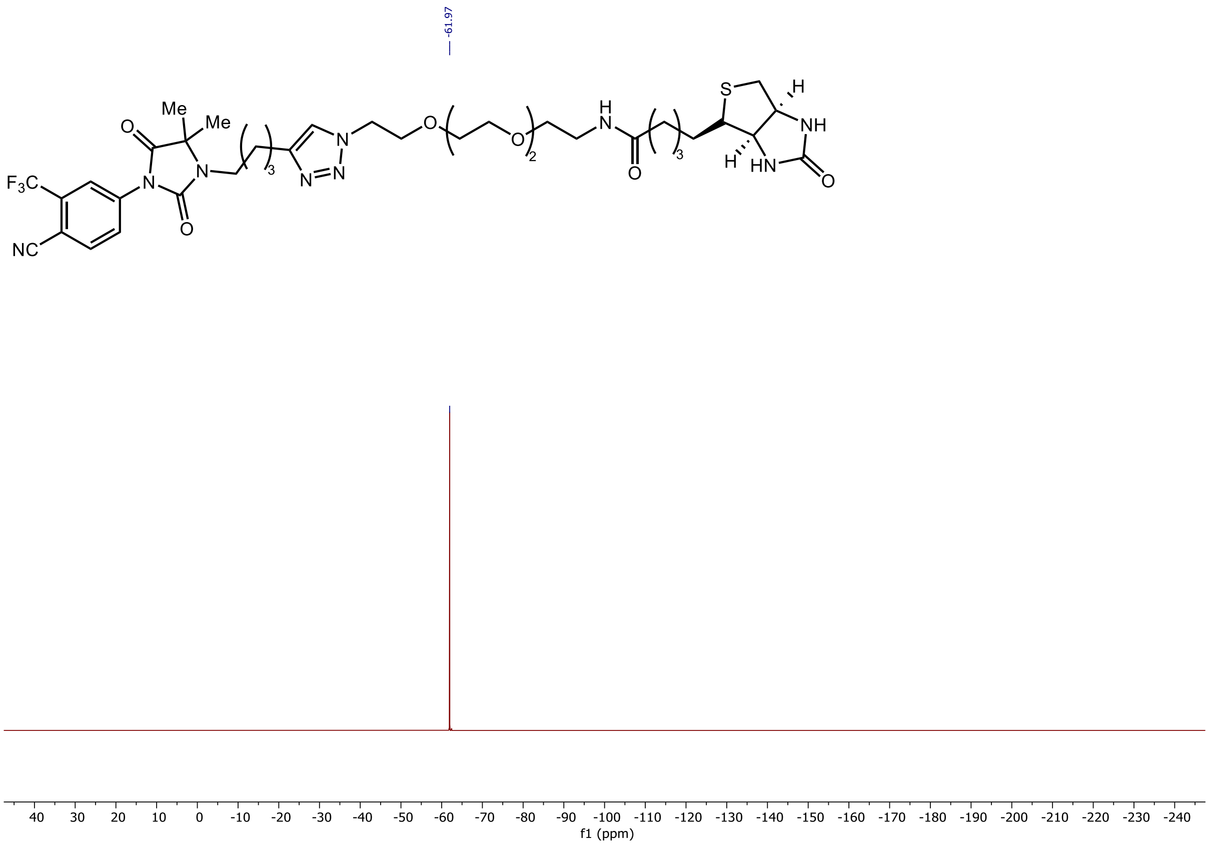

-61.97
Me
Me
F3C
NC
N
O
N
N=N
H
NH
HN
S
H
f1 (ppm)

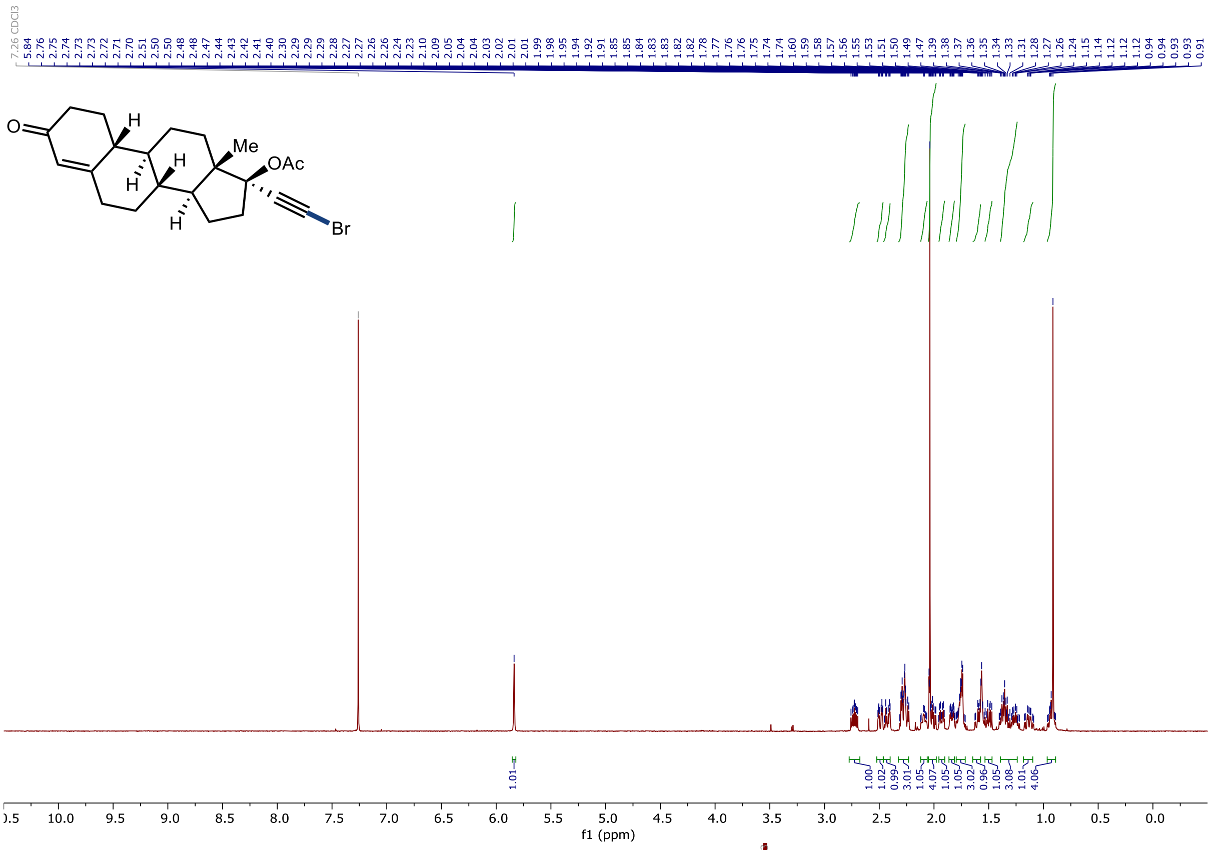

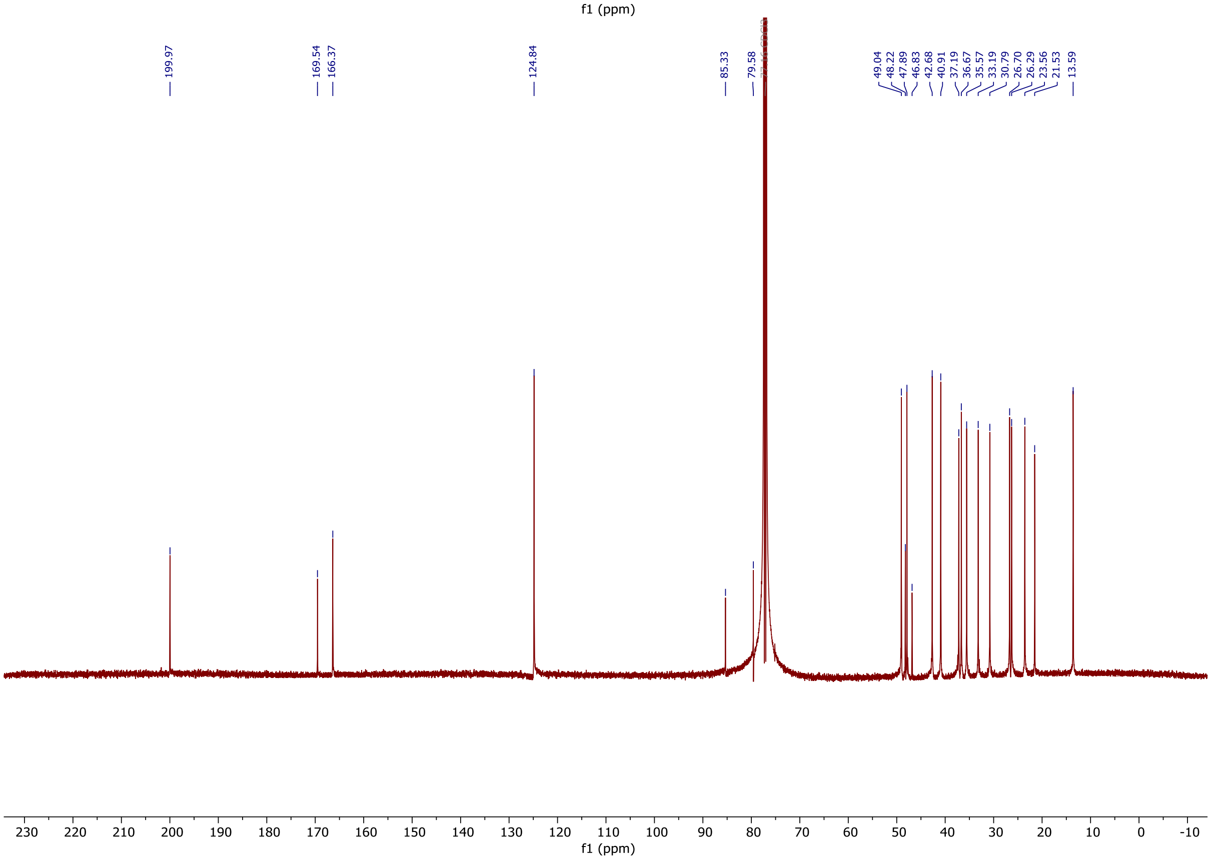

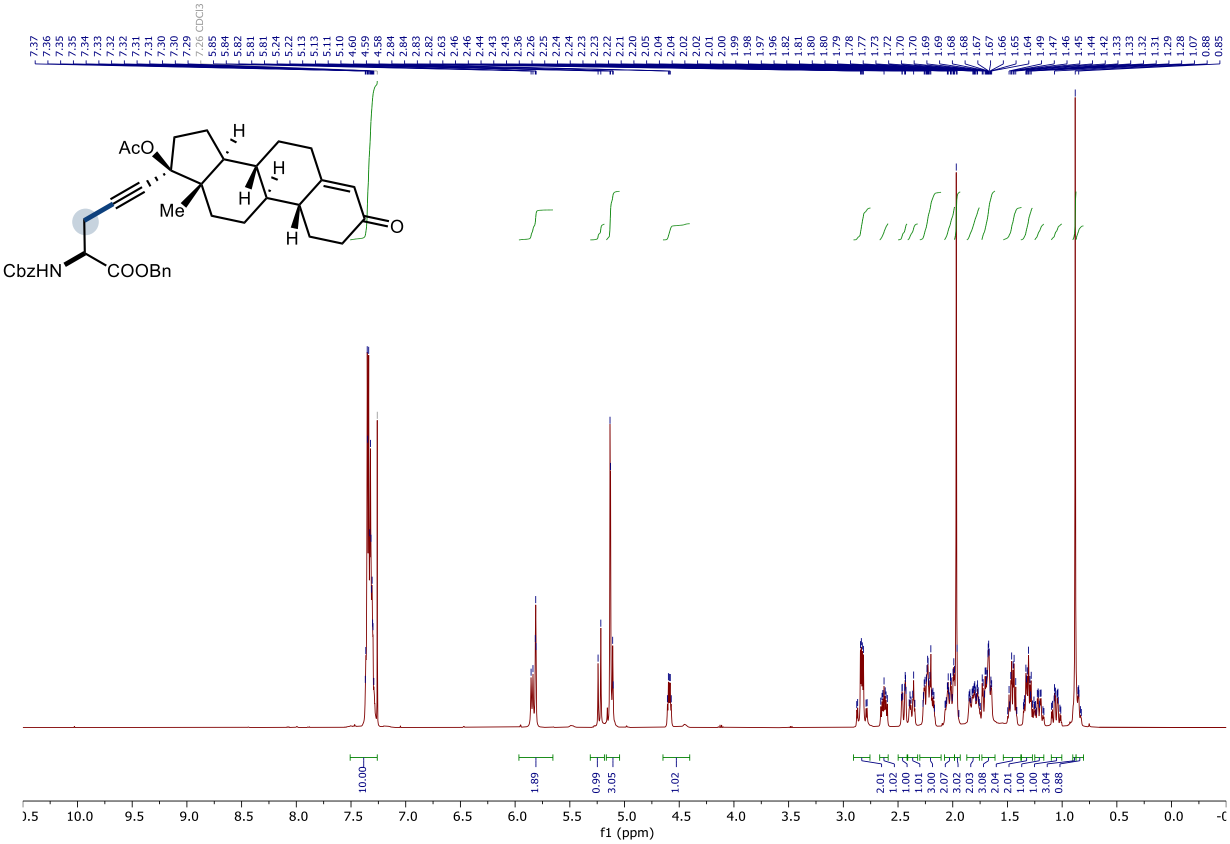
7.37
7.36
7.35
7.34
7.33
7.32
7.32
7.31
7.31
7.30
7.29
7.26 CDCl3
5.85
5.84
5.82
5.81
5.81
5.24
5.22
5.13
5.13
5.11
5.10
4.60
4.59
4.58
2.84
2.84
2.83
2.82
2.63
2.46
2.46
2.44
2.43
2.36
2.26
2.25
2.24
2.24
2.23
2.22
2.21
2.20
2.05
2.04
2.02
2.02
2.01
2.00
1.99
1.98
1.97
1.96
1.82
1.81
1.80
1.80
1.79
1.78
1.77
1.73
1.72
1.70
1.70
1.69
1.69
1.68
1.68
1.67
1.67
1.66
1.65
1.64
1.49
1.47
1.46
1.45
1.44
1.42
1.33
1.33
1.32
1.31
1.29
1.28
1.07
0.88
0.85
AcO
H
H
H
Me
H
O
CbzHN
COOBn
10.00
1.89
0.99
3.05
1.02
2.01
1.02
1.00
1.01
3.00
2.07
3.02
2.03
3.08
2.04
2.01
1.00
1.00
3.04
0.88
f1 (ppm)

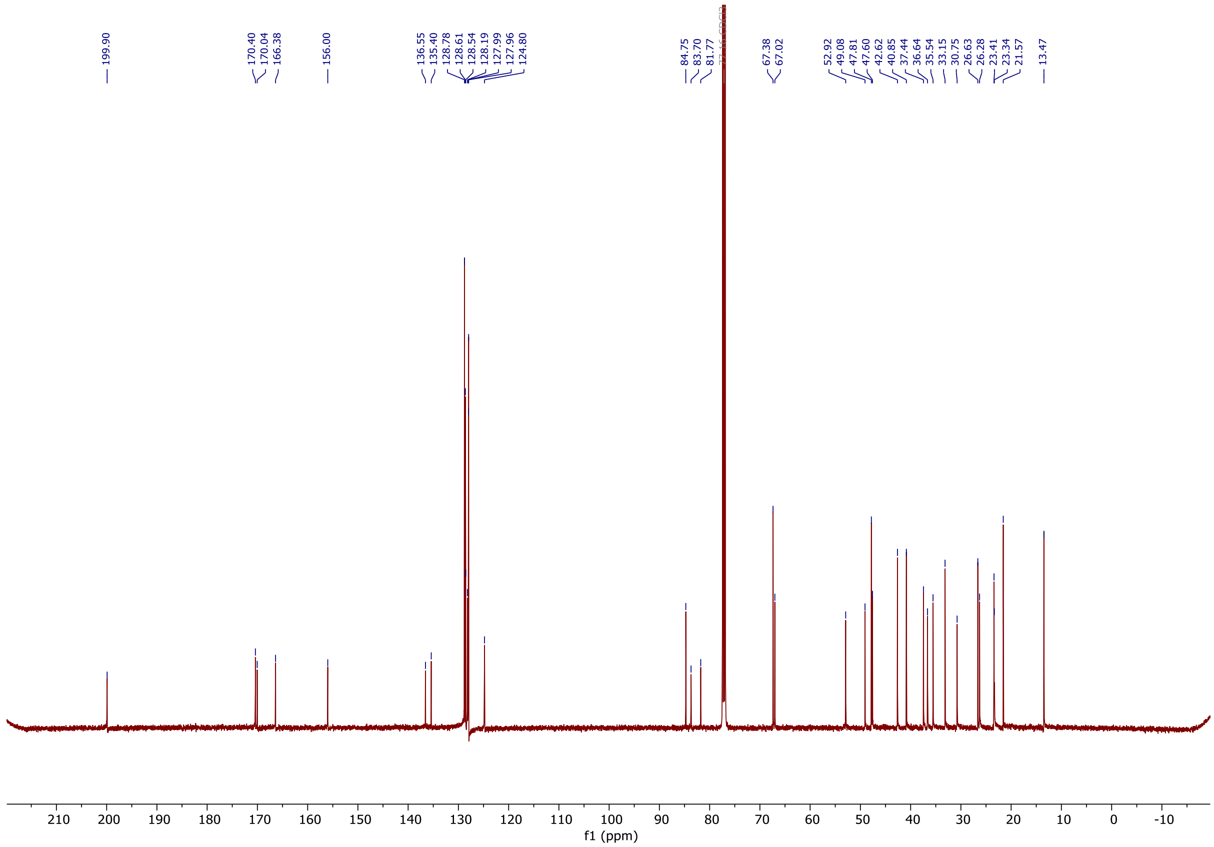
199.90
170.40
170.04
166.38
156.00
136.55
135.40
128.78
128.61
128.54
128.19
127.99
127.96
124.80
84.75
83.70
81.77
77.16 CDCl3
67.38
67.02
52.92
49.08
47.81
47.60
42.62
40.85
37.44
36.64
35.54
33.15
30.75
26.63
26.28
23.41
23.34
21.57
13.47
f1 (ppm)

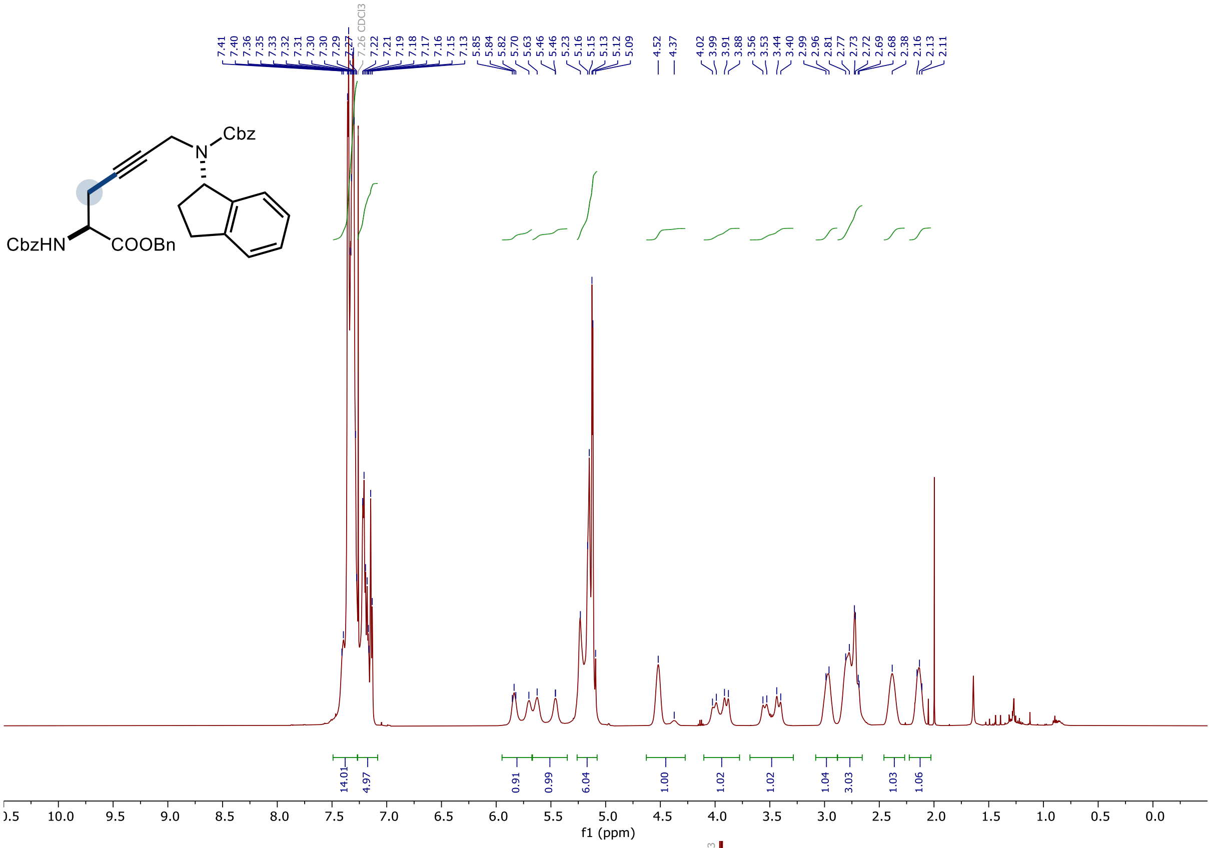
Cbz
N
CbzHN
COOBn
7.41
7.40
7.36
7.35
7.33
7.32
7.31
7.30
7.29
7.27
7.26 CDCl3
7.22
7.21
7.19
7.18
7.17
7.16
7.15
7.13
5.85
5.84
5.82
5.70
5.63
5.46
5.46
5.23
5.16
5.15
5.13
5.12
5.09
4.52
4.37
4.02
3.99
3.91
3.88
3.56
3.53
3.44
3.40
2.99
2.96
2.81
2.77
2.73
2.72
2.69
2.68
2.38
2.16
2.13
2.11
14.01
4.97
0.91
0.99
6.04
1.00
1.02
1.02
1.04
3.03
1.03
1.06
f1 (ppm)

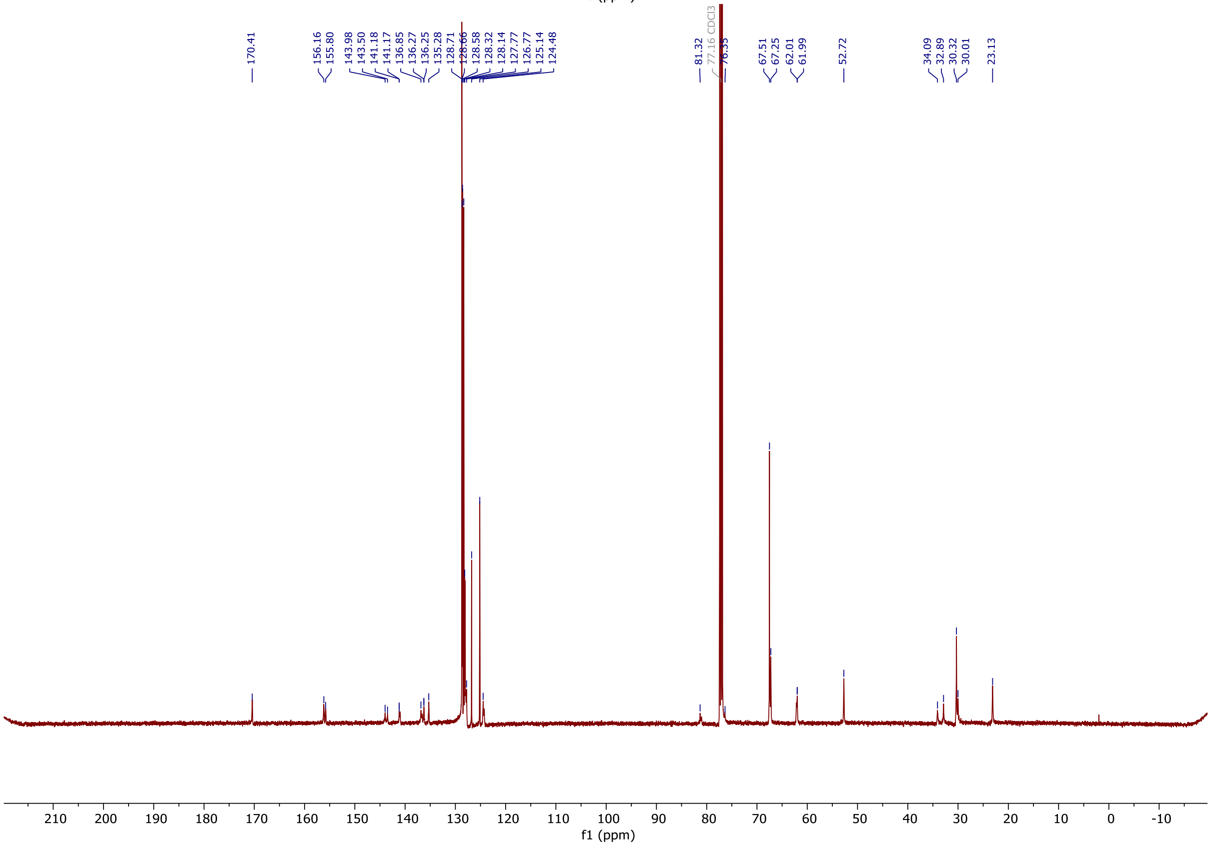
170.41
156.16
155.80
143.98
143.50
141.18
141.17
136.85
136.27
136.25
135.28
128.71
128.66
128.58
128.32
128.14
127.77
126.77
125.14
124.48
81.32
77.16 CDCl3
76.35
67.51
67.25
62.01
61.99
52.72
34.09
32.89
30.32
30.01
23.13
f1 (ppm)

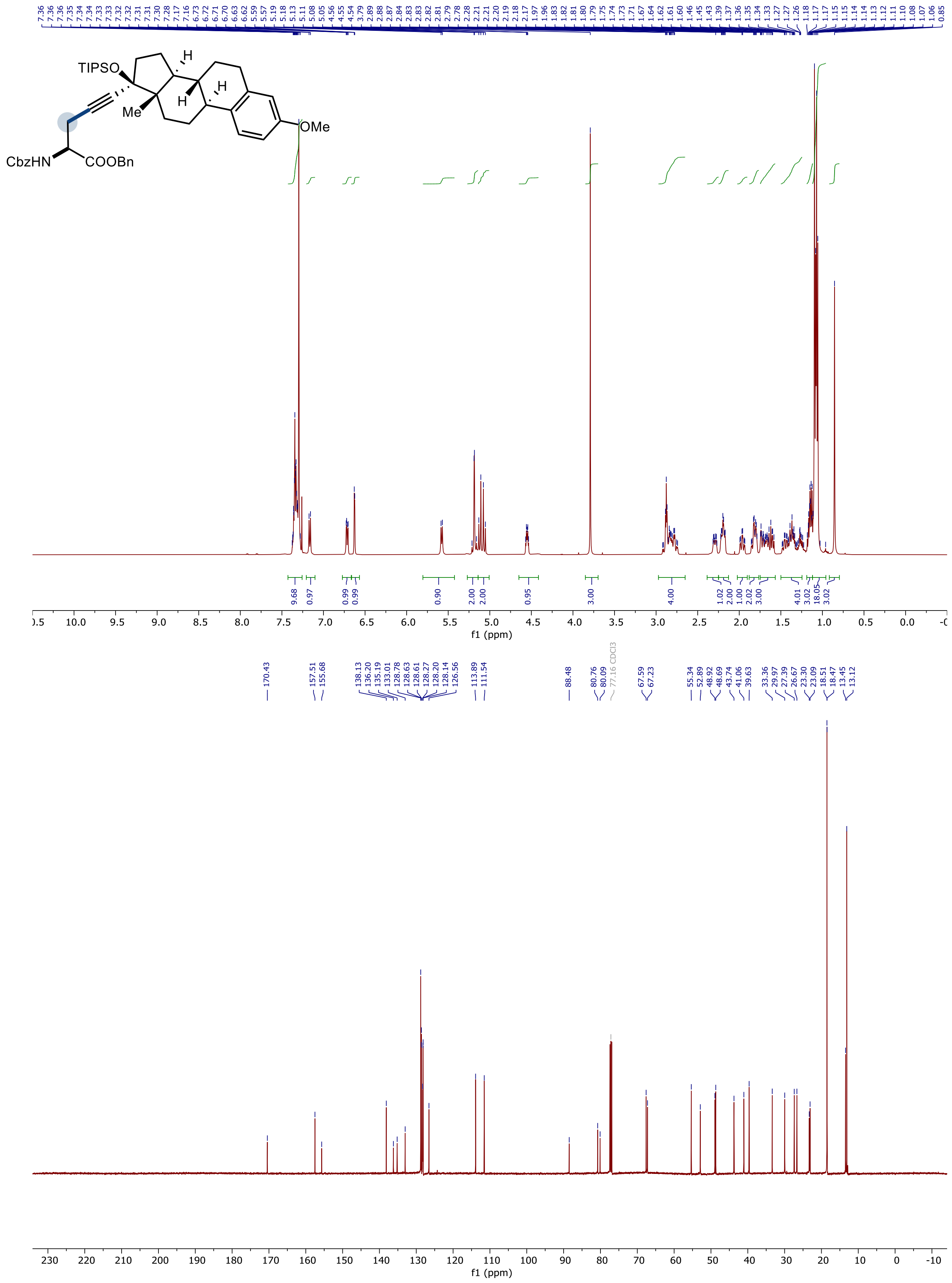

Additional Previously Unreported Compounds

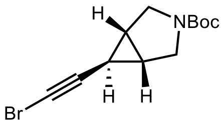

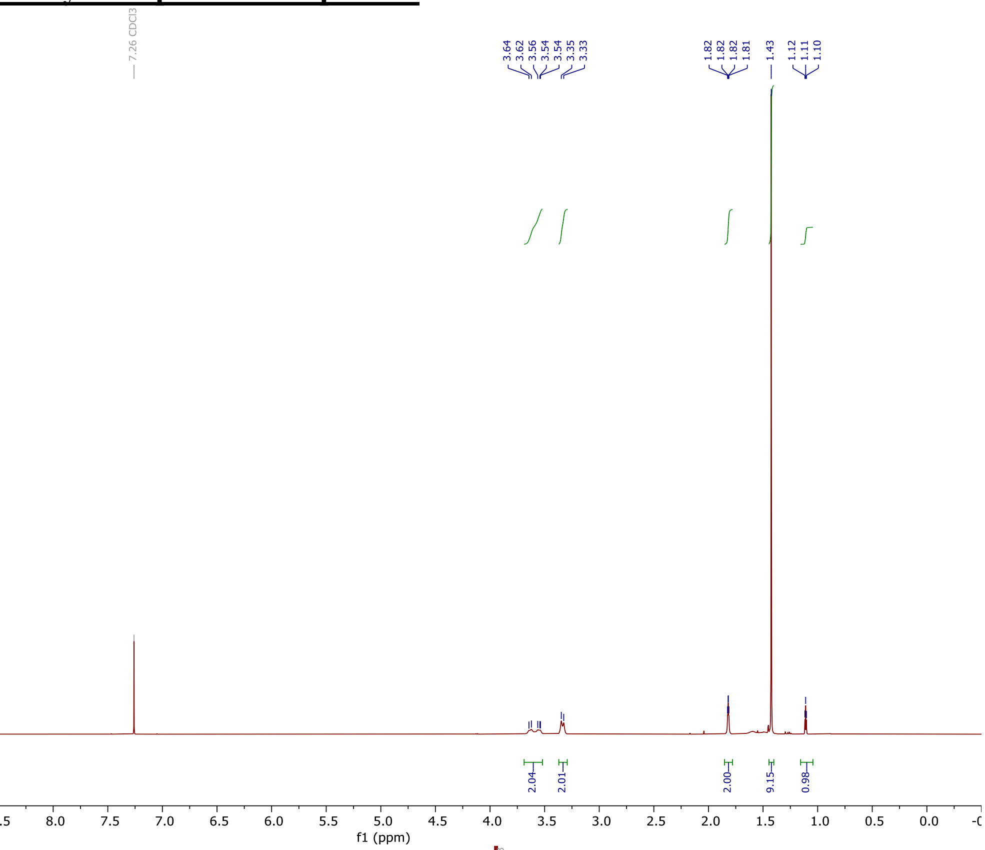

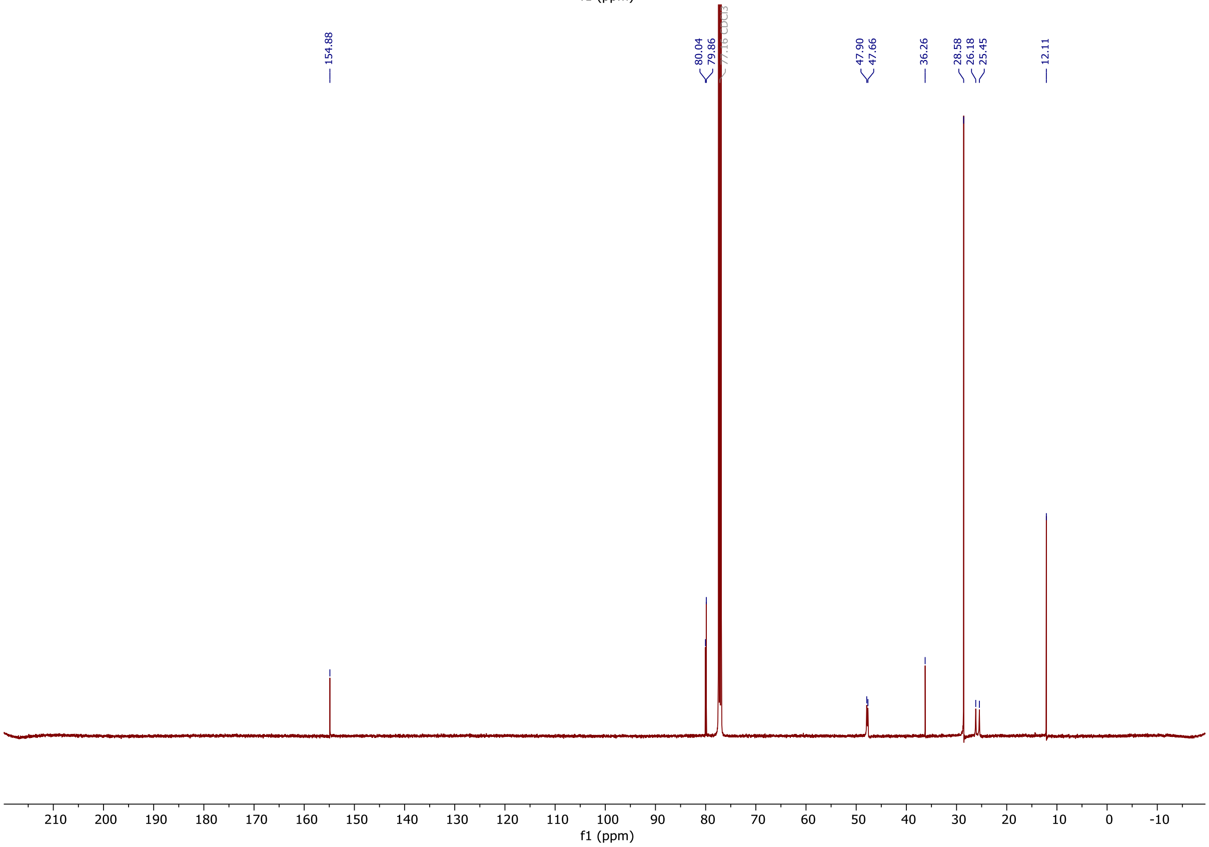

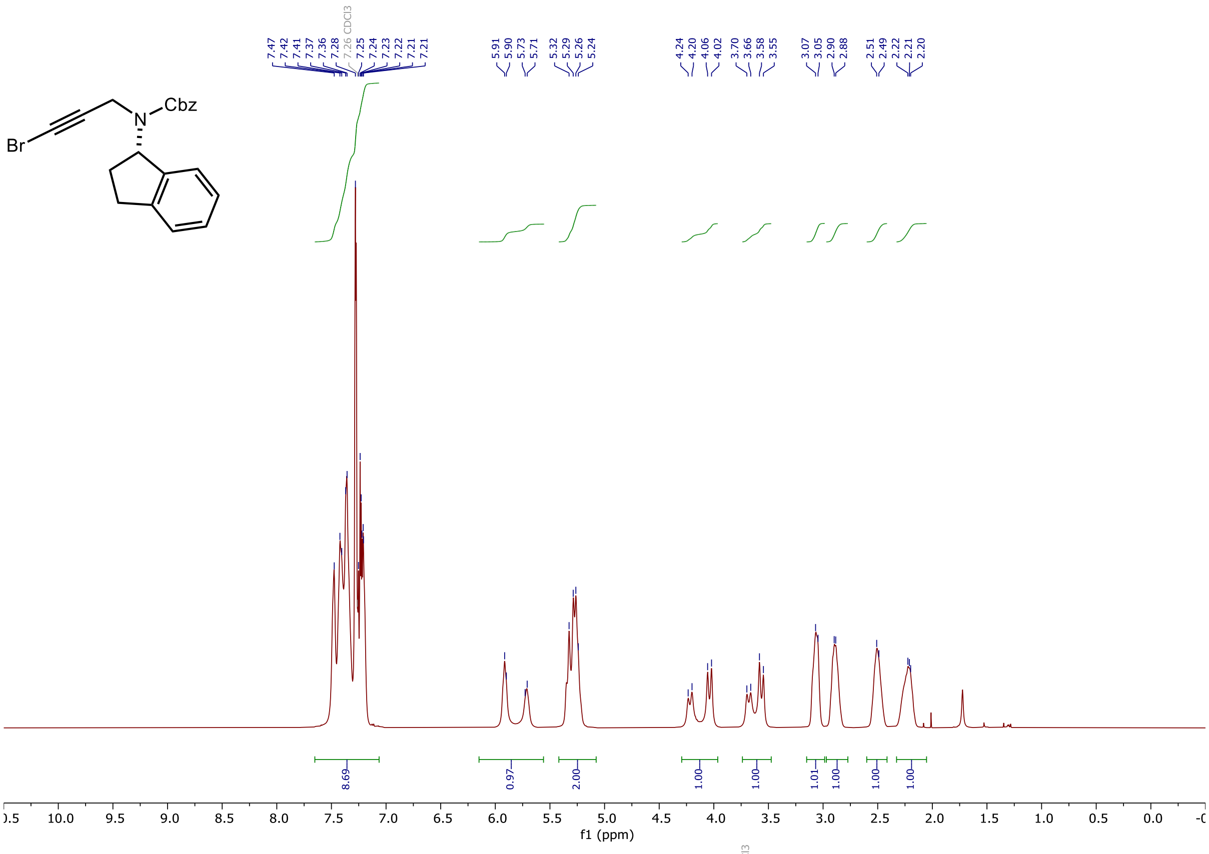
Cbz
N
Br
7.47
7.42
7.41
7.37
7.36
7.28
7.26 CDCl3
7.25
7.24
7.23
7.22
7.21
7.21
5.91
5.90
5.73
5.71
5.32
5.29
5.26
5.24
4.24
4.20
4.06
4.02
3.70
3.66
3.58
3.55
3.07
3.05
2.90
2.88
2.51
2.49
2.22
2.21
2.20
8.69
0.97
2.00
1.00
1.00
1.01
1.00
1.00
1.00
f1 (ppm)

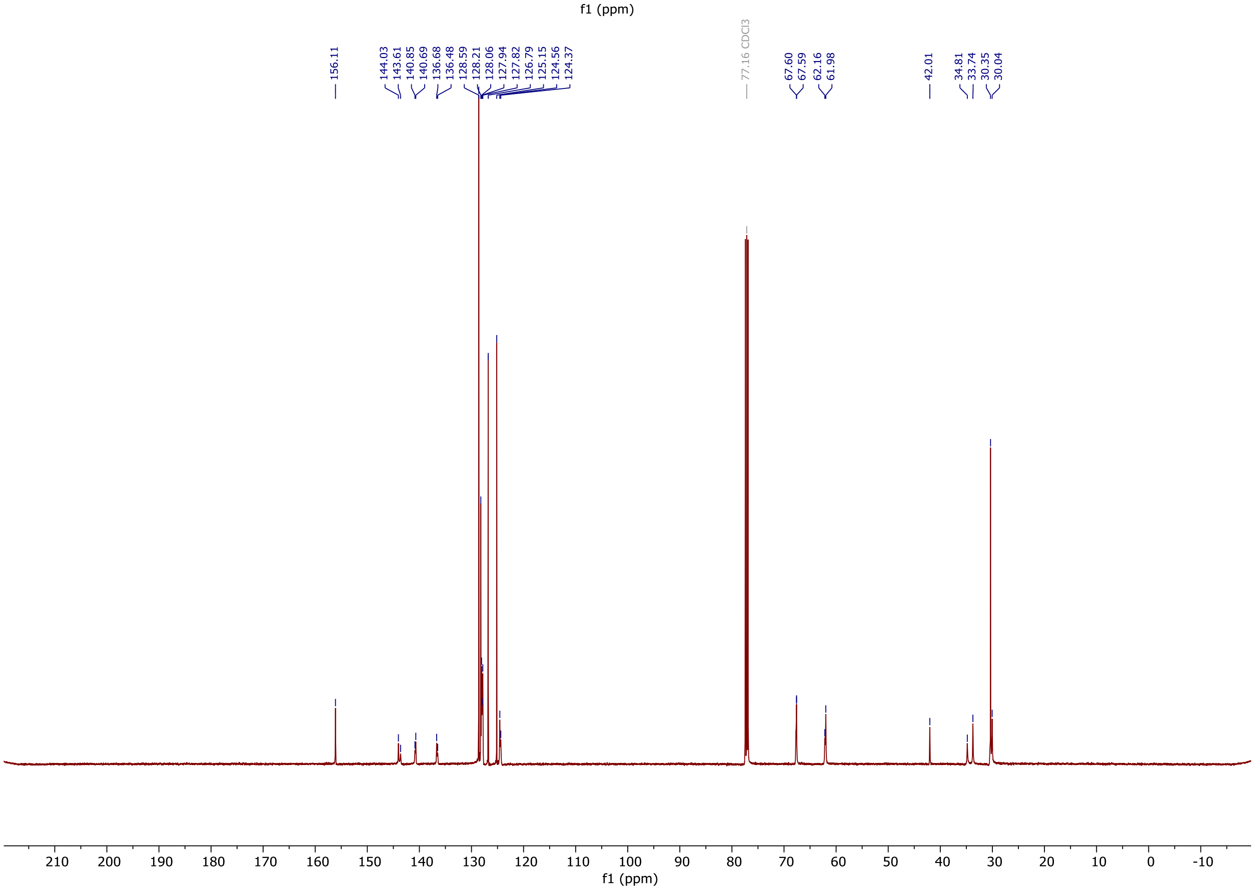
156.11
144.03
143.61
140.85
140.69
136.68
136.48
128.59
128.21
128.06
127.94
127.82
126.79
125.15
124.56
124.37
77.16 CDCl3
67.60
67.59
62.16
61.98
42.01
34.81
33.74
30.35
30.04
f1 (ppm)

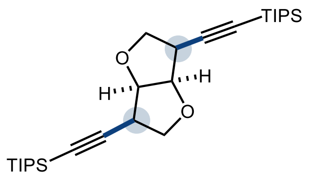
TIPS
H
H
O
O
TIPS

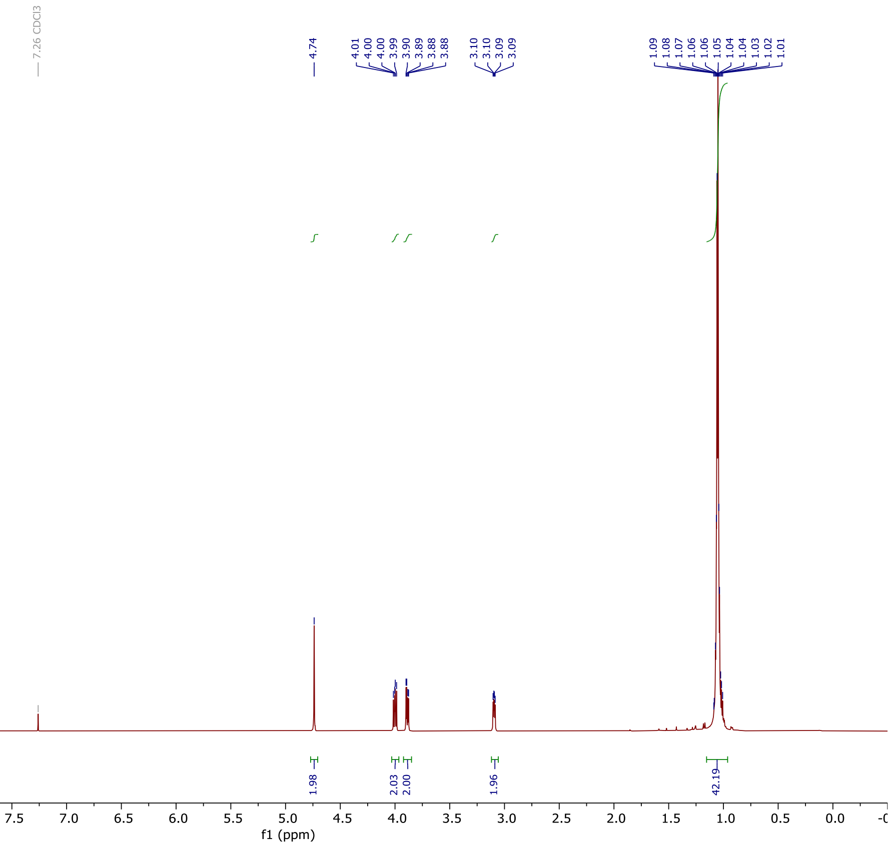
7.26 CDCl3
4.74
4.01
4.00
4.00
3.99
3.90
3.89
3.88
3.88
3.10
3.10
3.09
3.09
1.09
1.08
1.07
1.06
1.06
1.05
1.04
1.04
1.03
1.02
1.01
1.98
2.03
2.00
1.96
42.19
f1 (ppm)

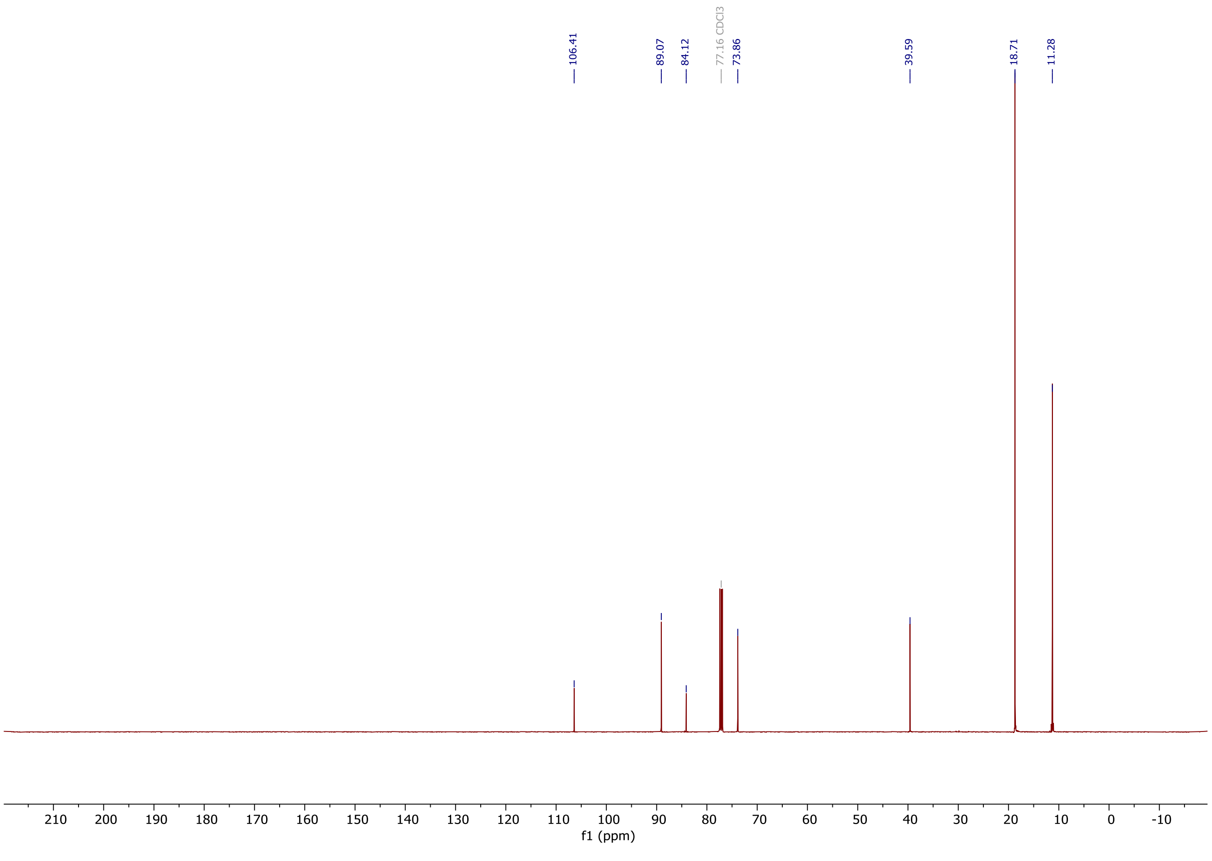
106.41
89.07
84.12
77.16 CDCl3
73.86
39.59
18.71
11.28
f1 (ppm)

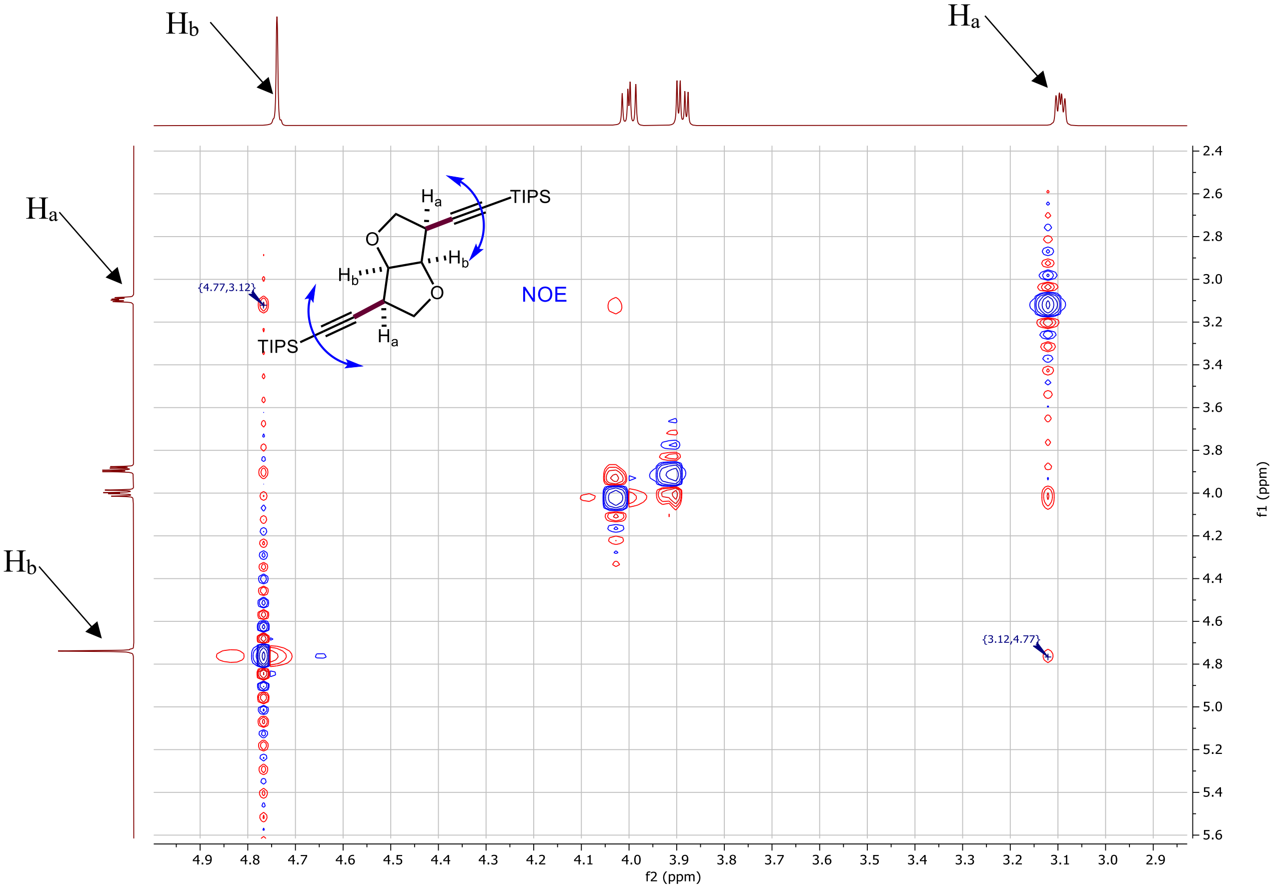

H_a-H_b with NOESY

11) Citations

1. Pangborn, A. B., Giardello, M. A., Grubbs, R.H., Rosen, R. K., Timmers, F. J. Safe and Convenient Procedure for Solvent Purification. *Organometallics*, **1996**, *15*, 1518–1520.
2. Dong, Z. & MacMillan, D. W. C. Metallaphotoredox-enabled deoxygenative arylation of alcohols. *Nature* **2021**, *598*, 451–456.
3. Rana, N.; Huang, S.; Patel, P.; Samuni, U.; Sabatino, D. Synthesis, characterization and anti-cancer activity of a peptide nucleolipid bioconjugate. *Bioorg. Med. Chem. Lett.* **2016**, *26*, 3567– 3571.
4. Liu, T.; Qiao, J. X.; Poss, M. A.; Yu, J.-Q. Palladium(II)-catalyzed site-selective C(sp^3)-H alkynylation of oligopeptides: a linchpin approach for oligopeptide–drug conjugation. *Angew. Chem., Int. Ed.* **2017**, *56*, 10924– 10927.
5. Quinodoz, P.; Quelhas, A.; Wright, K.; Drouillat, B.; Marrot, J.; Couty, F. Iodocarbamation of *N*-Homopropargyl Carbamates: Mild and Stereoselective Entry to Functionalized Oxazinan-2-ones. *Eur. J. Org. Chem.* **2017**, *2017*, 2621– 2626.
